# Amplicon Analysis of Dictean Cave Microbial Communities and Essential Oils as a Mild Biocide

**DOI:** 10.1264/jsme2.ME24115

**Published:** 2025-09-25

**Authors:** Olga Martzoukou, Alexandra Oikonomou, Sotiris Amillis, Dimitris G. Hatzinikolaou

**Affiliations:** 1 Enzyme and Microbial Biotechnology Unit, Department of Biology, National and Kapodistrian University of Athens, Athens, Greece; 2 Ephorate of Palaeoanthropology and Speleology, Hellenic Republic Ministry of Culture, Athens, Greece

**Keywords:** Amplicon, Biodeterioration, Diktaion Andron, Lampenflora, Microbiome

## Abstract

Naturally occurring caves are sites of significant cultural value, while also displaying the unique biodiversity of associated microbiomes that may provide an untapped source of potentially beneficial organisms. However, the touristic exploitation of show caves may ultimately result in the biodeterioration of speleothems, primarily through the introduction and establishment of alien microbiota or the uncontrolled growth of indigenous species, exacerbated by the use of artificial lighting. These habitat characteristics are present in the Dictean cave, also known as “Diktaion Andron”, a highly visited cave in eastern Crete, Greece, which was regarded in ancient Greek mythology as one of the putative sites of the birth of Zeus. Therefore, an efficient approach to controlling these ecological niches without irreversibly disturbing microbial diversity is needed, and essential oils are currently being investigated as a mild cleaning method. The present study exami­ned the microbial diversity of the Dictean cave using 16S and 18S rRNA gene amplicon sequencing and methods for quantitative metabolic activity estimations and also investigated the application of a formulation containing specific essential oils as a mild cleaning method. Amplicon sequencing ana­lyses revealed distinct profiles among the different sample sites, with species of the genera *Pseudomonas*, *Sporosarcina*, *Butiauxella*, *Glutamicibacter*, *Paenibacillus*, *Mortierella*, and *Jenufa* being the most abundant, while uncharacterized microorganisms were also detected. The single simultaneous application of a formulation of 0.2% (v/v) oregano and 0.4% (v/v) cinnamon essential oils was effective at significantly reducing microbial metabolic activity by up to 89.2% within 24 h, without adversely affecting the coloration of speleothems.

Natural caves present unique secluded environments on Earth, and, in most cases, are characterized by a microenvironment of constant low temperatures and high moisture in the absence of light. These habitats allow for the development of ecologically diverse species of flora, fauna, and microorganisms adapted to the limiting conditions of the cave ecosystem (Lamprinou *et al.*, 2013; Rangseekaew and Pathom-aree, 2019; Cardoso *et al.*, 2022; Kosznik-Kwaśnicka *et al.*, 2022; Reboleira *et al.*, 2022; Preedanon *et al.*, 2023; Mohtar *et al.*, 2024). Moreover, speleothems constitute a detailed archive of various natural phenomena recorded over periods of time ranging from thousands to millions of years (Forti, 2009). In addition to their intrinsic ecological and natural value, caves are of high cultural, historical, and archaeological significance because they have served as habitats for humans since prehistoric times. Therefore, it is important to ensure that monuments of natural and cultural heritage are visitable to the public.

Nevertheless, the process of habitat colonization by microorganisms as well as biofilm growth naturally changes the substrate on which growth occurs. The correlation between the processes of deterioration of stone monuments and the uncontrolled biological activity of microorganisms, including bacteria, fungi, algae, mosses, and lichens, has been firmly established (Ortega-Calvo *et al.*, 1991, 1995; Warscheid and Braams, 2000). Regarding show caves and other subterranean environments, the growth of photosynthetic microorganisms has been perceived in recent decades as a significant threat to the preservation of cultural and natural heritage. The presence of non-photosynthetic micro­organisms and/or microflora in these extremely fragile ecosystems may change the resident microbiome. Importantly, show caves may exhibit common microbial characteristics that differ from those found in a natural cave (Biagioli *et al.*, 2023). At an advanced stage, changes are observed in the form of aesthetic changes to the surface of cave walls and speleothems, affecting their natural coloring, and, thus, are regarded as a “bioburden” for the natural cave environment. Additionally, the growth of these microorganisms may induce structural changes, whether physical or chemical, such as erosion, weathering, and degradation of the growth substrate (Warscheid and Braams, 2000; Martino, 2016). Therefore, it is necessary to limit the growth of microorganisms that proliferate in the cave environment in order to address this emerging ecological issue and protect sites of high cultural value (Estévez *et al.*, 2019). However, it is of paramount importance to implement a solution that does not transfer this ecological issue from the cave walls and cave interior to the water table (Kalhor *et al.*, 2019). In recent years, research has focused on the search for ecological, environmentally friendly solutions that are not toxic to neighboring ecosystems (Trinh *et al.*, 2018; Argyri *et al.*, 2021; Mohapatra *et al.*, 2023; Tsouggou *et al.*, 2023).

The term “Lampenflora” (or “lamp flora”) is used to describe autotrophic microorganisms that proliferate in the vicinity of artificial light sources (Dobat, 1963). Collectively, they are referred to as “photosynthetic microflora” and include prokaryotic microorganisms, such as cyanobacteria, as well as eukaryotic microalgae, including chlorophytes, phaeophytes, diatoms, and certain plants. The most common manifestation of photosynthetic microorganisms is the formation of green or dark (black or brown) deposits on cave walls or the ground. This is particularly evident in areas where artificial light is prevalent. Photosynthetic microflora gain access to the cave interior via several different mechanisms, including air currents, gravitational settling through small cavities or cracks in the rock surface, flowing water, and the mobility of cave fauna and visitors (Cigna, 2012; Mulec, 2012). As a consequence, show caves face the particular challenge of the appearance and propagation of complex communities composed of invading microorganisms that displace the original cave biota (Tomczyk-Żak and Zielenkiewicz, 2016). The proliferation of these microorganisms facilitates the degradation of the substrates they colonize, ultimately leading to the destruction of the speleothems and a consequent reduction in the cave’s viewing value. Apart from the cave entrance area, photosynthetic microflora exploit artificial light to grow, exhibiting a marked preference for specific wavelengths (430–490 and 640–690‍ ‍nm) (Olson, 2006; Cigna, 2011; Mulec, 2012). This issue is compounded by the high intensity and prolonged duration of lighting as well as elevated humidity. Furthermore, periods of high visitor traffic represent a significant exacerbating factor. Carbon dioxide (CO_2_) released by visitors’ respiration contributes to an increase in the photosynthetic capacity of lampenflora, which is incorporated into their biomass (Grobbelaar, 2000). The formation of biofilms of autotrophic microorganisms then promotes the growth of heterotrophic microorganisms, mosses, and ferns in close proximity due to the richness of the environment in nutrients and organic matter (Warscheid and Braams, 2000; Cigna, 2011; Martino, 2016). Sources of organic matter include animal excrement (*e.g.*, bats and birds), sediments transported through water flowing inside the cave, and other materials, including fluff, hair, dry skin, and dust from footwear, introduced into tourist caves by visitors (Grobbelaar, 2000). To resolve this issue, two distinct strategies are employed, the first of which involves the prevention of new microorganism establishment, while the second concerns the elimination of pre-established microorganisms.

In this context, various solutions have been proposed, including both physical and chemical methods. Physical methods include reducing visitor numbers in line with the “carrying capacity” of the cave, defined as the maximum number of visitors the ecosystem may receive without causing permanent environmental damage (Hoyos *et al.*, 1998; Cigna and Burri, 2000). Furthermore, the use of low-power LED lamps in lieu of conventional incandescent lamps is employed in combination with other treatment methods because microflora development is dependent on the intensity and wavelength of the light. Another proposed method is interrupted lighting through the use of motion sensors, which ensures minimal light exposure, particularly in relation to visitor traffic (Cigna, 2011; Havlena *et al.*, 2021; Piano *et al.*, 2021). Mechanical scraping is not recommended because it has the potential to spread lampenflora and, thus, intensify the issue (Hebelka, 2014). Moreover, the utilization of pressurized water for cleaning purposes may inevitably result in the destruction of fragile cave speleothems (Spate and Moses, 1994; Werker and Hildreth-Werker, 2004; Mulec, 2012). Regarding chemical methods for controlling photosynthetic microflora, it is essential that specific environmental standards are met to prevent toxicity and the disruption of neighboring aquatic ecosystems. Concurrently, formulations must exhibit high efficacy and long duration of action. Bromine-containing chemicals have been excluded due to their high toxicity, while certain biocides affect stone coloration despite their satisfactory activity (Nugari *et al.*, 2009). Moreover, mixtures of quaternary ammonium compounds (QACs) may be utilized by bacteria and fungi as the sole source of carbon and nitrogen while also promoting biocide selection, leading to secondary overgrowth (Bastian *et al.*, 2010; Mohapatra *et al.*, 2023). Chemical compounds, including NaOCl and Ca(ClO)_2_, are considered less toxic to the environment, but do not act against endolithic microorganisms, such as the filamentous cyanobacterium *Scytonema julianum* and *Leptolyngbya sp.*, while also damaging cave decorations (Iliopoulou-Georgoudaki *et al.*, 1993; Estévez *et al.*, 2019). Furthermore, hypochlorite solutions may react with ammonia, forming toxic chloramines or even carcinogenic compounds, such as trihalomethanes (THMs) (Faimon *et al.*, 2003). Traditional chemical agents not only disrupt the unique cave biodiversity, but may also lead to speleothem damage or generate pollutant effluents that adversely affect groundwaters (Estévez *et al.*, 2019). Recent findings highlighted the utilization of essential oils (EOs) via spraying and their potential as “natural” biocides, offering an alternative to conventional chemical agents (Barresi *et al.*, 2017). These plant-derived solutions exhibit considerable efficacy at inhibiting the growth of epilithic microorganisms, and present a promising alternative to the highly toxic spray formulations previously employed (*e.g.*, paraquat and chloride-based compounds) (Swamy *et al.*, 2016; Fidanza and Caneva, 2019; Cappitelli *et al.*, 2020; Romani *et al.*, 2022; Mateus *et al.*, 2023; Russo and Palla, 2023). Specific essential oils, including oregano and cinnamon essential oils, are considered less toxic than antibiotics and are used as therapeutical agents for a series of infections and medical conditions (El Atki *et al.*, 2019; Sim *et al.*, 2019; Drioiche *et al.*, 2024; El-Sherbiny *et al.*, 2024; Fernandes *et al.*, 2024; Suljević *et al.*, 2024). In addition, the use of raw oregano and cinnamon essential oils is qualified as ‘Generally Recognized As Safe’ (GRAS) (Es *et al.*, 2017; Leonelli Pires de Campos *et al.*, 2022; Alonso *et al.*, 2024). However, it is important to note that other essential oils and plant extracts have been shown to exert toxic effects on non-target organisms (Zárybnický *et al.*, 2018; Ferraz *et al.*, 2022). Therefore, it is recommended that essential oil formulations are selected carefully and used with caution by specially trained staff and in accordance with the existing regulations when considering large-scale applications for the prevention of biodeterioration (Romani *et al.*, 2022).

Previous studies demonstrated the potent antimicrobial activity of essential oils derived from, among other sources, *Origanum vulgare* (oregano), *Cinnamomum cassia* (cinnamon), *Satureja thymbra* (thyme-leaved savory or Cretan savory), and *Laurus nobilis* (laurel) (El Atki *et al.*, 2019; Argyri *et al.*, 2021; Leonelli Pires de Campos *et al.*, 2022; Tsouggou *et al.*, 2023). Of note, the simultaneous application of oregano and cinnamon essential oils at low concentrations was shown to be promising for the elimination of metabolic activity at the Greek Petralona cave (Tsouggou *et al.*, 2023). The mechanisms of action of carvacrol and cinnamaldehyde are pleiotropic and include impairment of the cell membrane, resulting in the leakage of cellular contents and loss of membrane potential (Ultee *et al.*, 2002; Bouhdid *et al.*, 2010; Chavan and Tupe, 2014; Košćak *et al.*, 2023; Duan *et al.*, 2024), the inhibition of biofilm formation by down-regulating the transcription of motility genes, and other intracellular mechanisms of action that include ROS generation via the Fenton reaction and the inhibition of ATPase, membrane porins, cell division, and bacterial quorum sensing (Lambert *et al.*, 2001; Vasconcelos *et al.*, 2018; Lee *et al.*, 2020; Guo *et al.*, 2024; Liu *et al.*, 2024).

Essential oils exhibit antimicrobial activity against a diverse range of microorganisms, including cyanobacteria, green algae, bacteria (both Gram-positive and -negative), and fungi. The genera *Escherichia*, *Pseudomonas*, *Staphylococcus*, *Bacillus*, *Achromobacter*, *Paenibacillus*, *Rhodococcus*, *Aspergillus*, and *Penicillium* as well as *Fusarium* have been identified as examples of microorganisms susceptible to the antimicrobial effects of various essential oils (Lambert *et al.*, 2001; Nazzaro *et al.*, 2017; Rotolo *et al.*, 2018; El Atki *et al.*, 2019; Sim *et al.*, 2019; Zerrifi *et al.*, 2020; Argyri *et al.*, 2021; Leonelli Pires de Campos *et al.*, 2022). Notably, oregano and cinnamon essential oils exhibit antimicrobial efficacy at low concentrations, with MIC values ranging from 0.020–0.630% (v/v) against bacterial pathogens and 0.004–0.156% (v/v) against fungal species (Sim *et al.*, 2019; Simirgiotis *et al.*, 2020; Argyri *et al.*, 2021).

Naturally occurring caves are important ecosystems that may harbor potentially beneficial microorganisms. Secondary metabolite-producing microorganisms, comprising bacteria and, to a lesser extent, fungi, serve as prominent examples within this category. Actinomycetes, particularly members of the genus *Streptomyces*, are a notable example of this group, even though bacteria from various genera, including *Nonomuraea*, *Agromyces*, *Nocardia*, *Rhodococcus*, *Micrococcus* and *Bacillus*, have also been reported (Rangseekaew and Pathom-aree, 2019; Cyske *et al.*, 2021). Bioactive compounds isolated from the cave microbiota exhibit mostly antibacterial and anticancer bioactivities, with potential applications in the field of medical microbiology (Ghosh *et al.*, 2017; Kosznik-Kwaśnicka *et al.*, 2022).

We herein investigated the microbial bioburden in the highly visited Dictean cave in Crete, Greece and evaluated the effectiveness of the application of oregano and cinnamon essential oil mixtures to reduce microbial metabolic activity. Specifically, we employed 16S and 18S rRNA gene amplicon sequencing to examine the microbial diversity of endogenous and alien species, collected from five sampling sites with visible changes in speleothems in the Dictean cave. Samples were initially exami­ned through optical microscopy, revealing the presence of photosynthetic (green) microorganisms. We also assessed CO_2_ levels, temperature, and relative humidity in various areas of the cave interior and proceeded to a quantitative estimation of microbial metabolic activity through the measurement of adenosine triphosphate (ATP) levels *in situ*. With the aim of reducing the microbial bioburden, we performed a test application of an essential oil formulation containing 0.2% (v/v) oregano oil and 0.4% (v/v) cinnamon oil, estimated residual metabolic activity, and assessed its impact on sampling site coloration. The effects of essential oils are promising for large-scale applications, with the objective of the complete restoration of Dictean cave speleothems using this mild cleaning method.

## Materials and Methods

### Study site

Diktaion Andron, also known as “Psychro cave” and “Dictean cave”, is situated on the northern slope of Mount Dikti in Crete, within the western region of the Lassithi prefecture in the Lassithi Plateau municipality, 600‍ ‍m from the village of Psychro, at an altitude of 1,025 m. The geographical coordinates of Psychro cave are 35°09′46.4″N 25°26′42.4″E. Ancient Greek mythology stated that the birth (genesis) of Zeus by his mother Rhea took place in the Dictean cave, whereas it was also claimed that his whole childhood was spent there, raised by the Dictae Curetes, under the care of the goat Amalthea and the nymph Melissa (Hogarth, 1900). Therefore, Psychro Cave was considered a sacred grotto during antiquity, particularly in the Minoan era and until the Roman times. Of note, archeologists recovered various ritual objects, such as bronze statuettes, jewelry, pottery, and ancient weapons (Hogarth, 1900; Watrous, 1996). The Dictean Cave has been converted into a tourist site, and the tourist path comprises a total length of 250‍ ‍m with an estimated total area of 2,200 m^2^ and is visited by more than 200,000 visitors annually. The cave contains numerous stalactites, stalagmites, and several pillars, as well as a subterranean lake. However, the lake located in the lower part of the cave has largely receded (current water depth ~15‍ ‍cm) following an earthquake with intense seismic activity of magnitude 5.9 that occurred in the area on September 27, 2021 and was centered in Arkalochori Minoa Pediados (Ganas *et al.*, 2022). The cave is divided into upper and lower levels. The latter comprises five chambers and is of particular interest because the majority of speleothems are situated there and objects of archaeological and cultural value have been recovered from its chambers (Hogarth, 1900) (Fig. S1).

### Sample & data collection

Microbial samples were collected using cotton swabs immersed in buffered peptone water (CompactDry SWABS for surface testing; Shimadzu Diagnostics Europe), which was also used as a preculture medium. Two samples were collected from each site and stored at 4°C for up to 24 h. A map of the cave is presented in Fig. S1, showing the five sampling sites and cave chambers. Additionally, the position of the lake is indicated.

Air conditions were monitored through measurements of CO_2_, temperature, and relative humidity using the CO_2_ monitor Air CO_2_ntrol (TFA).

### ATP measurements, colorimetry, and microscopy

In *in situ* metabolic activity estimations, the ATP measurement method was applied using the sampling system LuciPac Pen ATP swabs and the luminometer lumitester PD-20 (Kikkoman Biochemifa). ATP is the energy source of living cells and is also required for the oxidation of luciferin by the enzyme luciferase in a reaction that releases light (bioluminescence). Adenosine monophosphate (AMP) produced from this reaction is converted back to ATP using orthophosphate dikinase (PPDK) to obtain a large and stable amount of luminescence. The use of appropriate equipment allows for the direct and rapid assessment of microbial metabolic activity on inanimate surfaces *via* the quantification of ATP and/or AMP. This method offers a high degree of sensitivity, making it a suitable approach for monitoring microbial activity *in situ*. It is important for both the substrate, luciferin, and the enzyme, luciferase, to be present in excess in order to establish a linear relationship between the concentration of ATP/AMP and emitted bioluminescence (Mulec and Oarga-Mulec, 2016; van Arkel *et al.*, 2021). Emitted light is proportional to the amount of ATP and/or AMP present in the sample (10^–11^ mol L^–1^ to 10^–6^ mol L^–1^) and is expressed in relative luminosity units (RLU), where 1 pmol ATP corresponds to 1,000 RLU (Kikkoman Biochemifa).

The LC 100 Spectrocolorimeter (Lovibond) was used in color measurements, and results are expressed as numeric values describing lightness, L*, and the chromaticity coordinates, a* and b*. To assess color variations, the parameters Δa=a_After_–a_Before_, Δb=b_After_–b_Before_, and ΔL=L_After_–L_Before_ were calculated, when necessary.

Microscopic observations of the samples collected (see the “Sample & Data collection” section) were conducted by applying a small volume (1–2 drops) of each sample directly to glass microscope slides, placing a coverslip, and using a Bresser Researcher Trino microscope equipped with a 100× oil immersion objective lens and 10× ocular lens.

### DNA extraction

Total DNA was extracted from 250–500‍ ‍mg wet cell weight per sample using the Nucleospin Soil Kit (Macherey-Nagel, LabSupplies Scientific S.A., Hellas). Samples were vigorously vortexed and transferred from swab collectors to 1.5-mL Eppendorf tubes. In brief, samples were treated in Lysis buffer SL2 and Enhancer SX, followed by mechanical disruption using ceramic beads (Type A; size 0.6–0.8‍ ‍mm), the precipitation of debris, and the removal of potential inhibitors, according to the manufacturer’s instructions. The concentration (10–38‍ ‍ng μL^–1^) and purity of DNA (OD_260_/OD_280_ ~1.8) were estimated photometrically using the μDrop module coupled with the Multiskan GO microplate spectrophotometer (Thermo Fisher Scientific).

### 16S and 18S rDNA amplicon sequencing

Microbial diversity was analyzed in DNA samples from the five sampling sites by 16S and 18S rRNA gene sequencing for bacteria and eukaryotes, respectively. In the sequencing of 16S rRNA genes, we used the primers S-D-Bact-0341-b-S-17 (5′-CCTACGGGNGGCWGCAG-3′) and S-D-Bact-0785-a-A-24 (5′-GACTACHVGGGTATCTAATCC-3′), which target the V3 and V4 variable regions of the 16S rRNA genes (Herlemann *et al.*, 2011; Klindworth *et al.*, 2013), providing a good balance between taxonomic resolution and the broad coverage of diversity, encompassing a range of cyanobacteria and other bacterial groups (Klindworth *et al.*, 2013; Khomutovska *et al.*, 2020; Brown *et al.*, 2021; He *et al.*, 2022; Mezzasoma *et al.*, 2022). The primers E572F (5′-CYGCGGTAATTCCAGCTC-3′) and E1009R (5′-AYGGTATCTRATCRTCTTYG-3′) were selected to sequence 18S rRNA genes (Comeau *et al.*, 2011). Amplified sequences were sequenced on a MiSeq Illumina instrument (2×300 bp) at the MRDNA sequencing facilities. A polymerase chain reaction (PCR) was performed with the HotStarTaq Plus Master Mix Kit (Qiagen) for 30 cycles under the following conditions: at 94°C (3‍ ‍min), 94°C (30 s), 53°C (40 s), and 72°C (1‍ ‍min) and a final extension at 72°C (5‍ ‍min). Unprocessed DNA sequences are available in the Sequence Read Archive (https://www.ncbi.nlm.nih.gov/sra/) under BioSamples SAMN45948990–SAMN45948999 of the BioProject PRJNA1202040. All processing of raw 16S and 18S rRNA gene sequences was performed by Smallomics using the MOTHUR standard operating procedure (SOP) (v.1.46.1) (Schloss *et al.*, 2009). More specifically, after trimming barcodes and primer sequences, quality control was performed through the ‘screen.seqs’ command and sequences were removed according to the following filtering parameters: length <423 bp, ambiguous bases, average quality score <25, and homopolymers longer than eight nucleotides. The remaining sequences were aligned against the SILVA 138.1 database (Quast *et al.*, 2012; Yilmaz *et al.*, 2014). The VSEARCH algorithm (Rognes *et al.*, 2016) was used to detect and remove chimeric reads. Sequences were clustered into operational taxonomic units (OTUs) based on the average neighbor algorithm at a 97% sequence identity threshold followed by a calculation of diversity indices. High-quality OTU sequences were classified to different taxa according to the SILVA 138.1 database (Quast *et al.*, 2012; Yilmaz *et al.*, 2014) with a confidence value >80%. The taxonomic ana­lysis of OTUs was performed using the SILVA 138 database (Pruesse *et al.*, 2007). Bray-Curtis similarities between samples as well as alpha diversity indices were calculated using OTU abundance for all samples with Palaeontological Studies (PAST) software (Hammer *et al.*, 2001). Figures were created in GraphPad Prism 6.01.

### Formulation preparation & application

The small-scale application of essential oils was conducted at sampling sites 1–5 with the objective of eliminating metabolically active microorganisms from the walls and speleothems of the cave. A formulation comprising a 0.2% (v/v) solution of oregano essential oil and a 0.4% (v/v) solution of cinnamon essential oil was applied to the five sampling sites. This formulation was selected because the simultaneous application of oregano and cinnamon essential oils at similar concentrations was previously shown to reduce metabolic activity at the Greek Petralona cave (Tsouggou *et al.*, 2023). The solution was prepared in distilled water and contained 0.1% (v/v) Polyethylene Glycol (PEG) 400 Food grade (FCC) as an emulsifier. Oregano oil (e0070; Platon S.A., Hellas) contains carvacrol as the main active ingredient at ~70% and, thus, the final concentration of the substance in the formulation was ~0.14% (v/v). Similarly, the main active ingredient in cinnamon essential oil (e0035; Platon S.A.) is cinnamaldehyde, present in the formulation at a final concentration of 0.4% (v/v). The application of the formulation was conducted by spraying one selected surface of 10×10‍ ‍cm^2^ within each sampling site without brushing. The volume used was 100–120‍ ‍mL m^–2^. Metabolic activity was estimated by measuring RLU, as described in the section “ATP measurements, colorimetry, and microscopy”. RLU measurements for the selected colonized areas were conducted prior to formulation application as well as 5‍ ‍min and 24‍ ‍h after its application.

### Statistical ana­lysis

The results of measurements of metabolic activity (RLU) and colorimetric parameters (L, a, and b) at the five sampling sites prior to and after application of the formulation were statistically analyzed using GraphPad Prism 6.01. Regarding metabolic activity results, a one-sample *t*-test was initially performed to confirm the significance of differences within samples under the same condition (prior to or 5‍ ‍min or 24‍ ‍h post-application). To compare differences between conditions, a one-way ana­lysis of variance (ANOVA) with Tukey’s multiple comparison test was conducted. Concerning colorimetric parameters, a paired *t*-test was used to compare pre- and post-application values for each parameter (L, a, and b) and to assess the significance of ΔL (brightness), Δa (green-red), and Δb (blue-yellow). In all ana­lyses, the confidence interval was set to 95% (alpha=0.05).

## Results

### Sampling site selection & conditions monitored

**Fig. 1.**
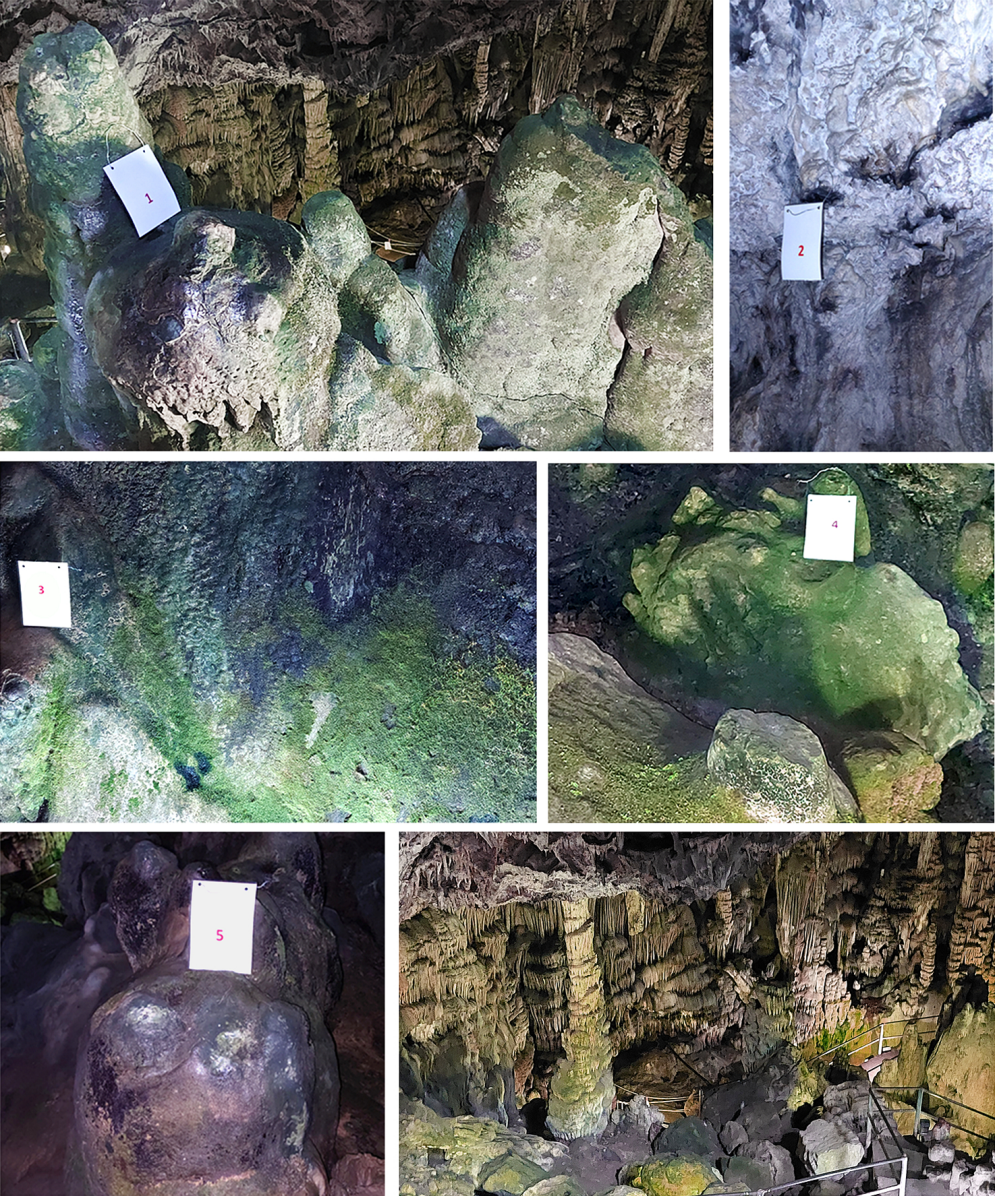
Sampling sites 1–5 and panoramic view of the cave interior, as seen from site 1 (bottom right). Note the predominance of green photosynthetic microflora at sites 1, 3, and 4 and the presence of black biofilms at sites 2, 3, and 5 (see also Table 1).

The selection of sampling points was based on the visible development of microflora on the cave walls and on the representation of areas located at different altitudes inside the cave. Fig. 1 shows photographs of the five sampling sites, while Table 1 summarizes macroscopic observations and shows measurements of CO_2_, temperature, and humidity at various locations in the cave, including the five sampling sites. The highest CO_2_ concentration was documented at the narrowest points of the cave, *i.e.*, the orifice and site 5. Temperature was relatively stable throughout the cave interior, at 16°C on average. Humidity was elevated at the entrance of the cave, and the lowest value was recorded at site 5.

### Microbial diversity in the Dictean cave

**Fig. 2.**
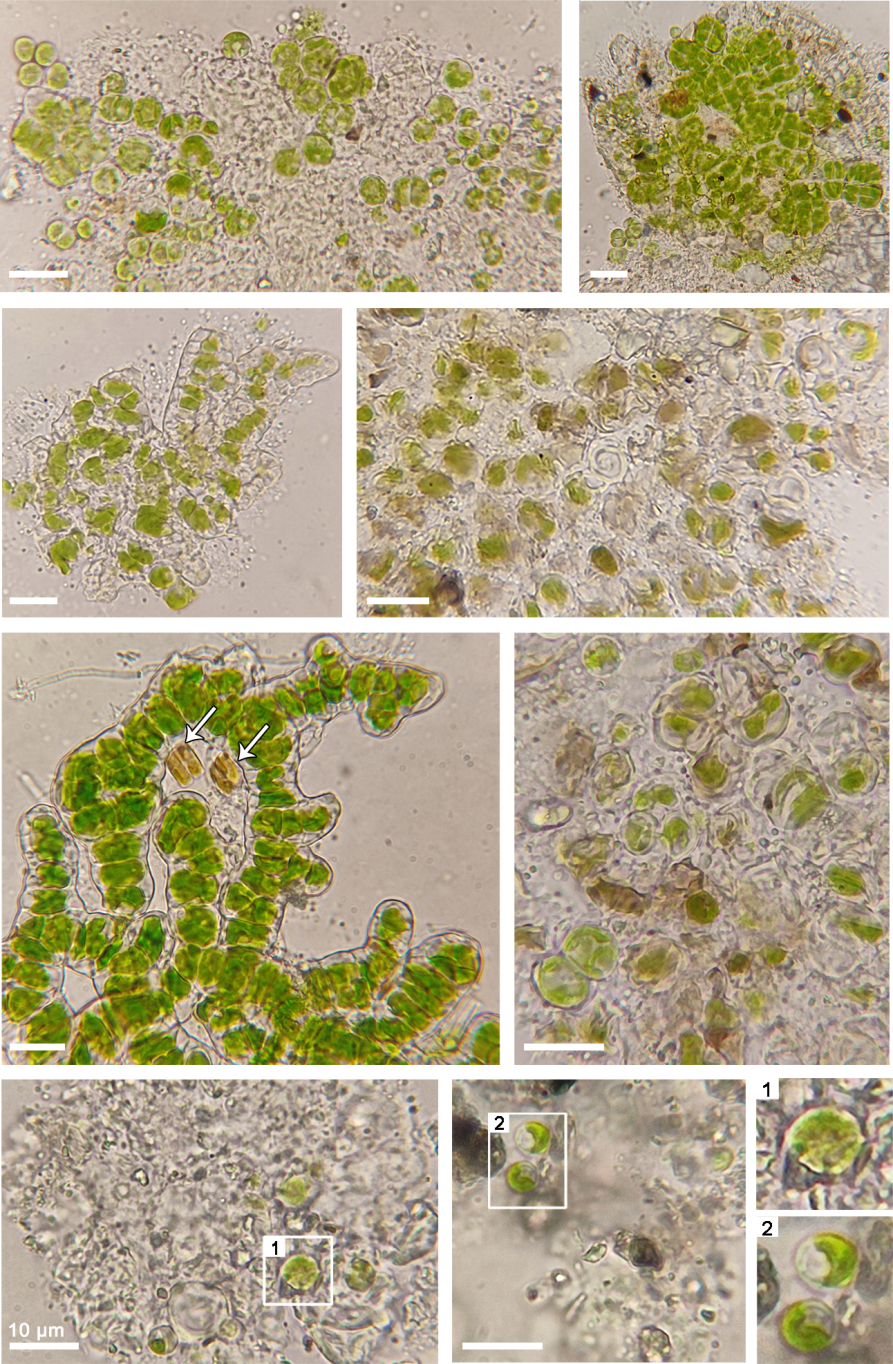
Representative microscopy images of photosynthetic microflora samples. Diatoms are marked with white arrows. Insets 1–2, bottom right, provide magnified views of the indicated areas on the left, presenting photosynthetic eukaryotes possibly belonging to *Jenufa* spp., and to the class *Trebouxiophyceae*, respectively. All scale bars represent 10‍ ‍μm (Images are derived from sites 3, 4, 1, 3, 1, 3, 4, and 1, respectively; left to right, top to bottom).

Initial microscopic observations of the samples revealed the presence of both green- and brown-colored microorganisms (Fig. 2). Photosynthetic microorganisms with large cell sizes, resembling chlorophytes, were observed, and magnified views are shown in Fig. 2. Representatives of *Diatomea* are also indicated with arrows (Fig. 2), and colorless, non-photosynthetic microorganisms, including rod-shaped bacteria, were present in all samples. Taxonomic identification was conducted through amplicon ana­lyses. Specifically, microbial diversity was analyzed by 16S and 18S amplicon sequencing for bacteria and eukaryotes, respectively. Results are presented separately for the two domains. A complete list of the microorganisms identified is provided in Table S1.

### Bacterial biodiversity

The majority of the microorganisms present in each of the five samples were detected, as indicated by the coverage index, which shows the percentage of species (defined here as OTUs) identified relative to the total number of species (Table 2). The Shannon diversity index is indicative of abundance (number of different species) and of the equal distribution of species. Site 2 had a lower Shannon index, which implied few species with very high relative abundance. In contrast, site 3 harbored the highest diversity of species among the five sampling sites.

**Fig. 3.**
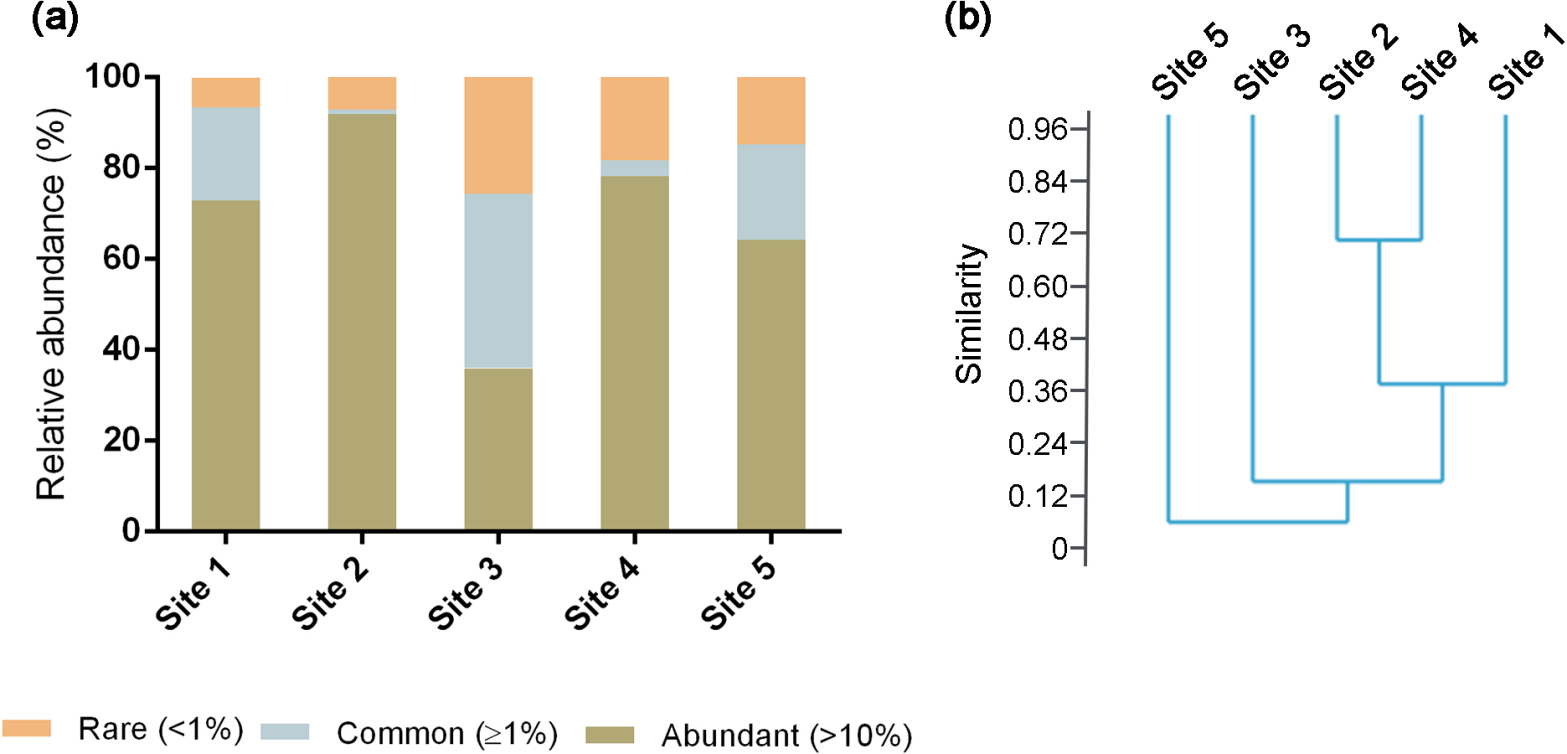
(a) Relative abundance of bacterial sequences belonging to abundant (>10%), common (>1%), and rare OTUs. (b) Dendrogram from a cluster ana­lysis using the Bray-Curtis similarity index.

Based on the relative abundance of the species detected, metabolically active members of the microbiological community in each sample, at a specific time point, mostly comprised abundant species (≥10%) and common species (1–10%). In all samples, 2,396 species were detected, of which only 34 were classified as abundant or common OTUs (see Table S1). However, the cumulative abundance of these species was >70% of the total bacterial sequences in all samples, consistent with the low overall values of the Shannon index (Fig. 3a, Table 2). A clustering ana­lysis using the Bray-Curtis similarity index revealed low similarities (<40%) between most samples, except Sites 2 and 4, where similarity was >70% (Fig. 3b).

**Fig. 4.**
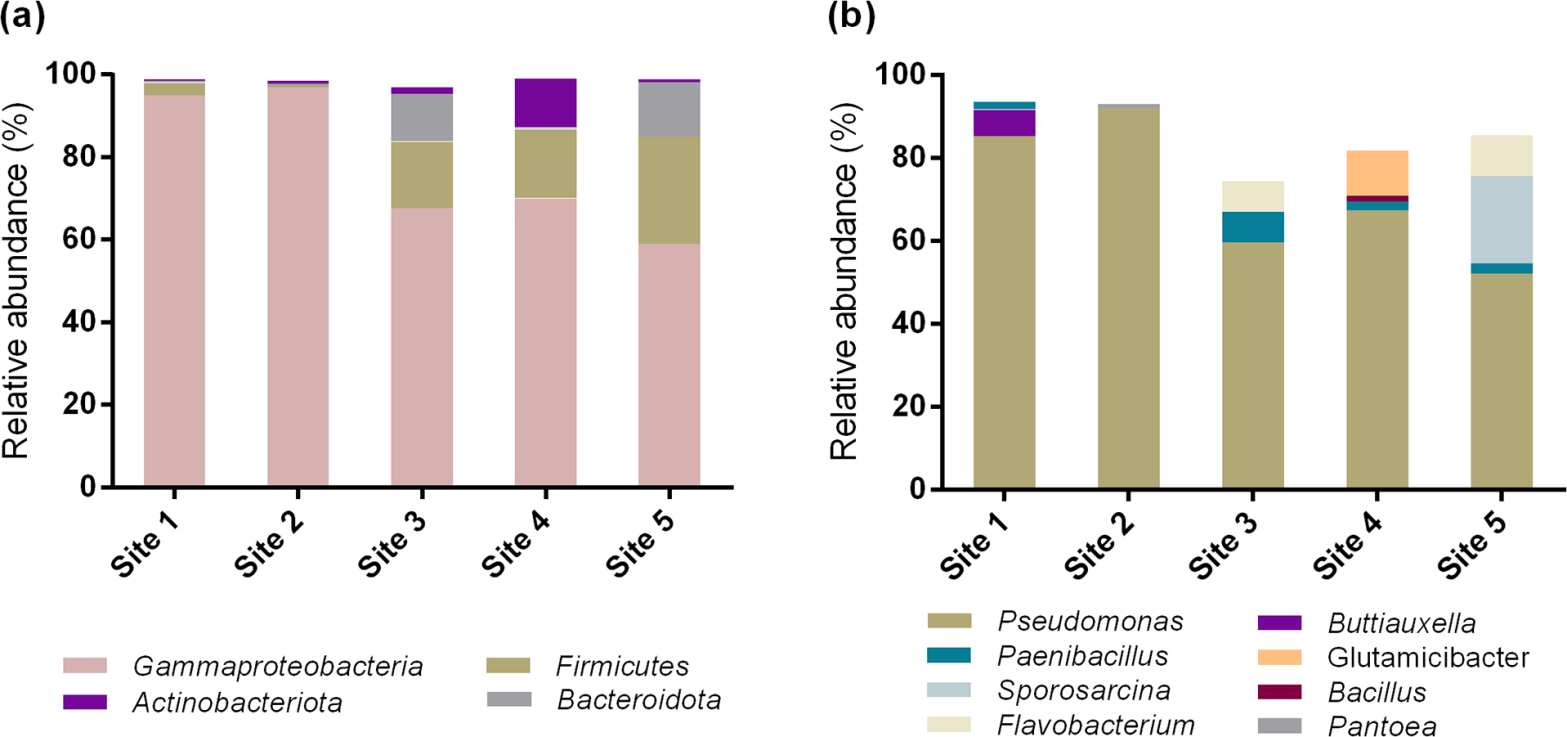
(a) Relative abundance (%) of the most abundant bacterial phyla. (b) Relative abundance (%) of common and abundant bacterial OTUs grouped at the genus level.

The taxonomic ana­lysis revealed that four phyla were the most prevalent, collectively representing more than 97% of the total abundance in all samples (Fig. 4a). Consistent with these results and diversity indices, the taxonomic ana­lysis of abundant and common OTUs identified eight genera in total across all samples, originating from the respective phyla (Fig. 4b). The genus *Pseudomonas* was the most prevalent in all samples, although the ana­lysis of OTUs revealed that different *Pseudomonas* species (Mulet *et al.*, 2010) were the most abundant in each sampling site (Table 3 and S1).

In addition to the three *Pseudomonas* species that were the most prevalent in site 1, the next most common genera were *Butiauxella* and *Paenibacillus* (Ferragut *et al.*, 1981; Ash *et al.*, 1994). Furthermore, the latter genus was present in sites 3–5 (Fig. 4b). Sites 2 and 4 exhibited increased similarities, likely attributable to the dominance of the same‍ ‍*Pseudomonas* species in both samples (>67%; *P. fluorescens*, GenBank accession number: AB680972). Additionally, the relative abundance of common OTUs was similar between the two sites (1.1 and 3.6%, respectively; Table S1). Among the common OTUs identified were the gammaproteobacterium *Pantoea* in Site 2 as well as the actinobacterium *Glutamicibacter* and bacteria of the genera *Bacillus* and *Paenibacillus* in site 4 (Fig. 4). Site 5 exhibited the most pronounced differences from the other sampling sites in terms of bacterial diversity (Fig. 3). This was also evident in abundant and common species, with the most predominant OTU belonging to *P. reinekei* (Table 3) and the‍ ‍remainder belonging to the genera *Sporosarcina*, *Flavobacterium*, and *Paenibacillus* (Stackebrandt *et al.*, 1987; Ash *et al.*, 1994; Barbier *et al.*, 2013) (Fig. 4).

### Eukaryotic biodiversity

**Fig. 5.**
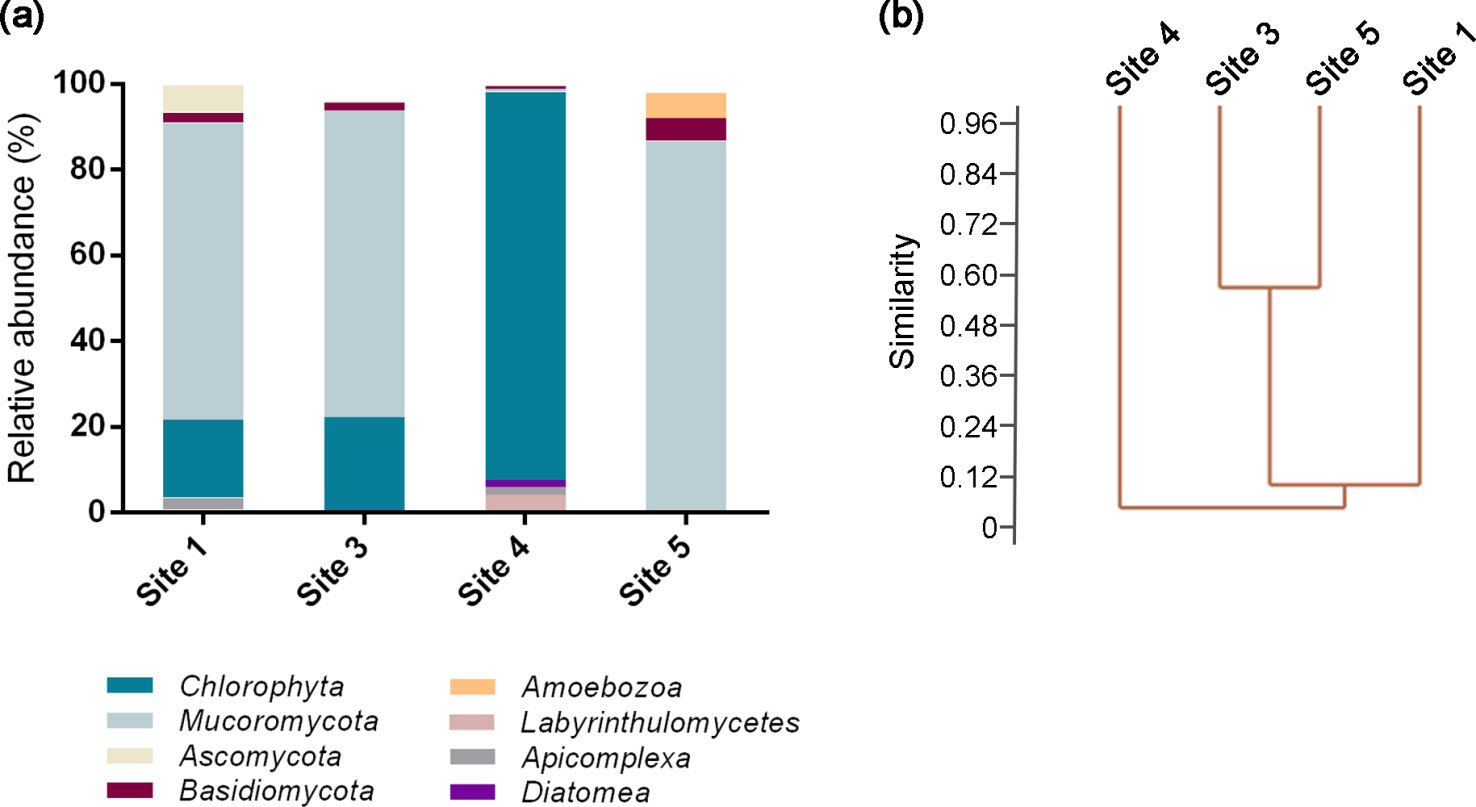
(a) Relative abundance of eukaryotic sequences belonging to abundant (>10%) OTUs grouped at the phylum level. (b) Dendrogram from a cluster ana­lysis using the Bray-Curtis similarity index.

Based on the results of 18S amplicon sequencing, we focused on unicellular organisms. The majority of unicellular eukaryotes were identified in four of the five samples analyzed (Sites 1 and 3–5). The number of sequences obtained in site 2 was very low (40% coverage; see Table 4), which precluded the identification of unicellular eukaryotes therein. The Shannon diversity index ranged between 0.9 and 2.4, suggesting limited eukaryotic diversity across the five sites. The clustering ana­lysis using the Bray-Curtis similarity index showed that similarities between the samples were generally low (<15%), apart from sites 3 and 5, which were more than 60% similar (Fig. 5). The taxonomic ana­lysis indicated that eukaryotic communities included representatives of various autotrophic, heterotrophic, saprophytic, and parasitic organisms. The most abundant groups were *Mucoromycota* (fungi) and *Chlorophyta* (Fig. 5).

At the genus level, site 1 was dominated by fungi of the genus *Mortierella* (Telagathoti *et al.*, 2022) (*Mucoromycota*) and phylogenetically close genera as well as green algae of the genus *Jenufa* (*Chlorophyceae*) and related genera. Site 3 was also predominantly composed of species belonging to the genus *Mortierella* and algae of the genus *Jenufa* (Němcová *et al.*, 2011), as well as uncultured *Chlorophyta* belonging to the family *Trebouxiophyceae* (Friedl, 1995; Guiry and Guiry, 2024). In site 4, chlorophytes were the most abundant phylum (90.53%), with representatives from species closely related to those identified in sites 1 and 3. Site 5 mostly harbored representatives of the genus *Mortierella*, while the presence of chlorophytes was negligible (Table S1).

In all sampling sites, numerous uncultured OTUs were identified through amplicon sequencing ana­lyses. Specifically, the relative abundance of uncultured bacteria in sites 1–5 were 3.93, 3.18, 14.60, 2.48, and 32.44%, respectively (2395 bacterial OTUs in total), whereas uncultured eukaryotes in sites 1 and 3–5 exhibited relative abundance equal to 48.42, 79.46, 44.16, and 85.05%, respectively (58 eukaryotic OTUs in total).

### Test application of the essential oil formulation

An initial estimation of metabolic activity was conducted through measurements of ATP (RLU) in the five sampling sites, and the results obtained are presented in Table 5 (‘Pre-application’). Sites 2 and 5 presented the highest metabolic activities, followed by sites 3, 1, and 4 in decreasing order. Notably, sites 2 and 5 also exhibited a predominance of black biofilms, in contrast to the other three sites where photosynthetic (green) microflora was prevailing. The application of the essential oil formulation was conducted by spraying of the same sites, and samples were collected for metabolic activity estimations 5‍ ‍min and 24‍ ‍h post-application (Table 5). Statistical ana­lyses indicated a significant reduction in bioluminescence following the application of essential oils (Prior *vs* 5‍ ‍min: P=0.0024; Prior *vs* 24 h: P=0.0004; 5‍ ‍min *vs* 24 h: not significant). Notably, sites 2 and 5 (black biofilms), which previously presented the highest metabolic activities, exhibited a rapid decrease 5‍ ‍min after the application of the essential oil formulation, whereas a milder effect was documented for site 1. In sites 3 and 4, where photosynthetic microflora was prevailing, a marked decrease in metabolic activity was documented 24‍ ‍h after the application of the essential oil formulation, indicating that a longer period of time (h instead of min) was required for the formulation to completely eliminate resistant photosynthetic microorganisms from cave surfaces (Table 5).

To assess the effects of essential oils on the coloration of colonized cave surfaces located in sampling sites 1–5, the colorimetric parameters L, a, and b were measured prior to and after the application of the essential oil formulation. “L” corresponds to the brightness of the color, “a” indicates the shades of red (+a) and green (–a), and “b” indicates the shades of yellow (+b) and blue (–b) (Table 6).

Minor changes were observed in coloration post-application, potentially signifying an enhancement of natural cave wall colors. The level of the green color was significantly lower across all sites (mean Δα=1.92; P=0.027) after the essential oil application, particularly in site 3 where photosynthetic microorganisms were initially prevailing (Δa_3_=3.8). Moreover, surfaces became more yellow in sites 1–4 (Δb positive; range: 0.8–4.7, P=0.05). Site 3 exhibited the highest Δb value (equal to 4.7) among the five sprayed surfaces. Furthermore, the surfaces became darker in sites 1 and 3–5 (ΔL negative; range: –6.1 to –0.6, P=0.09); however, this change was not significant. The most pronounced differences in brightness were documented in site 5 (ΔL_5_=–‍6.1), followed by site 3 (ΔL_3_=–3.2).

## Discussion

Despite the ecological and cultural importance of caves, human activities, particularly the influx of visitors, may have detrimental effects on these sensitive environments. The presence of a large number of visitors may change cave walls and speleothems, particularly when there is a significant increase in CO_2_ levels within the confined space of a cave. This, combined with high humidity and artificial lighting, may facilitate the spread and development of “alien” microorganisms, resulting in losses in natural cave diversity, speleothem integrity, and appearance. Specifically, natural caves are characterized by microorganisms adapted to low-light and limited nutrient availability conditions, such as bacteria, archaea, fungi, and other micro-eukaryotes, even though variations are observed not only between caves, but also within them (Tomczyk-Żak and Zielenkiewicz, 2016; Aldemaro, 2019). A microbial biofilm may include sulfur- and nitrite-oxidizing species as well as organotrophic bacteria (Aldemaro, 2019). In contrast, show caves typically harbor light-dependent and human-related microbial communities, such as algae and skin bacteria or fungi, respectively (Aldemaro, 2019; Biagioli *et al.*, 2023).

In the Dictean cave, the presence of photosynthetic microflora and black biofilms was evident prior to sample collection and ana­lyses. Amplicon sequencing further revealed the presence of multiple prokaryotic (16S) and eukaryotic (18S) phyla including, but not limited to, *Gammaproteobacteria*, *Firmicutes*, *Actinobacteriota*, *Bacteroidota*, *Chlorophyta*, *Muromycota*, *Ascomycota*, *Basidiomycota*, *Amoebozoa*, *Labyrinthulomycetes*, *Apicomplexa*, and *Diatomea*. Optical microscopy observations of samples also confirmed the presence of photosynthetic microorganisms, such as *Jenufa* sp. (Němcová *et al.*, 2011), whereas diatoms were also detected. Based on the amplicon ana­lyses conducted herein, the different sampling sites exhibited slightly different microbial diversity profiles and different Bray-Curtis similarity indices depending on whether 16S or 18S rRNA genes were analyzed. Microenvironmental factors, such as the substrate type, microclimatic differences, water availability, and other abiotic conditions affect cave microflora (Kosznik-Kwaśnicka *et al.*, 2022). In addition, variations in the microalgal community composition of show caves may be linked to artificial lighting (Biagioli *et al.*, 2023). However, the genus *Pseudomonas* was the most abundant bacterium in the 16S amplicon sequencing results of the five sampling sites. At the species level, microhabitat variability likely shaped the abundance of different *Pseudomonas* species, with factors such as localized nutrient availability, humidity, temperature fluctuations, oxygen levels, and microbial competition, which collectively create distinct ecological niches within a cave. Differences in rock surfaces, water flow, organic matter, or external factors (*e.g.*, human or animal activity) may further contribute to site-specific species distribution. The present results align with previous findings showing that even within seemingly uniform cave ecosystems, microbial communities exhibited spatial heterogeneity due to microenvironmental conditions (Northup and Lavoie, 2001; Ortiz *et al.*, 2013). Notably, Proteobacteria and Actinobacteria are the most abundant bacterial groups identified in cave ecosystems (Ikner *et al.*, 2007; Tomova *et al.*, 2013). *Pseudomonas* spp. have been detected in subterranean environments and particularly cave speleothems (Švec *et al.*, 2020; Zhu *et al.*, 2021), and their presence may be attributed to increased organic matter availability in high impact areas along cave trails, while microbial metabolism plays an active role in biogeochemical cycling in cave environments (Ikner *et al.*, 2007; Zhu *et al.*, 2021). Show caves are dominated by microbial taxa capable of degrading human-derived organic matter (*i.e.*, bacteria of the genus *Pseudomonas*) (Tomczyk-Żak and Zielenkiewicz, 2016; Biagioli *et al.*, 2023). Other human-associated taxa that are mainly or uniquely present in show caves (Biagioli *et al.*, 2023) were also found in the Dictean cave, including *Malasseziomycetes*, *Staphylococcus*, *Streptococcus*, *Lactococcus*, and *Legionella*. In addition, amplicon sequencing ana­lyses revealed the presence of numerous uncultured bacteria and eukaryotes, reaching relative abundance of up to 32 and 85%, respectively, some of which may represent novel and potentially beneficial taxa that offer sources for novel compound or enzyme discovery, such as *Massilia*, *Rhodobacter*, *Micrococcus,* *Bacillus*, and other *Actinobacteriota* as well as members of *Mortierella* and *Chlorophyta* (Wang *et al.*, 2011; Ghosh *et al.*, 2017; Kosznik-Kwaśnicka *et al.*, 2022). Of note, site 2 was macroscopically dominated by black biofilms and exhibited the highest metabolic activity among the five sampling sites, followed by site 5. The absence of observable photo­synthetic microflora in site 2 was in line with the results of‍ ‍the‍ ‍16S amplicon sequencing ana­lysis because *Gammaproteobacteria* and specifically *Pseudomonas* exhibited an abundance of 91.89%, the highest documented among the sites tested. Despite the observed high similarity between sites 2 and 4 based on the clustering ana­lysis of prokaryotes, it is not possible to draw definitive conclusions about actual differences in biodiversity due to the limited number of eukaryotic sequences obtained from site 2 (coverage 40%). Based on the present results, it is possible that the black biofilms observed at site 2 were resistant to lysis, composed of eukaryotic microorganisms, and that better coverage may have revealed the true biodiversity of site 2 via a clustering ana­lysis of eukaryotes. In addition, based on the Bray-Curtis ana­lysis of bacteria, site 5 exhibited lower similarity (<12%) to the other sites, indicating the highest level of divergence. In contrast, the amplicon ana­lysis for eukaryotes revealed a high degree of similarity between sites 3 and 5, both of which were dominated by *Mucoromycota* (black fungi). This result suggests that black biofilms were also abundant at site 3, even though green microflora predominated macroscopically. Overall, it is important to ensure the preservation of potentially beneficial microorganisms and their metagenomes, particularly in the case of unculturable or difficult-to-cultivate species, and, thus, regular sampling and amplicon ana­lyses need to be conducted to monitor biodiversity in caves.

Essential oils have been exami­ned as a mild cleaning method and are very promising for the efficient removal of lampenflora (Zerrifi *et al.*, 2020; Tsouggou *et al.*, 2023); however, their potential for use in the highly-visited Dictean cave had not previously been assessed. In the present study, the simultaneous application of oregano 0.2% (v/v) and cinnamon 0.4% (v/v) essential oils resulted in the efficient reduction of microbial metabolic activity, as evidenced by measurements of ATP *in situ*. This method is simple, fast, and sensitive and offers an estimation of the bioburden with a strong degree of linear predictability for the majority of cell types (Turner *et al.*, 2010). However, results may vary based on the properties of the sampled surface, which, in turn, affect the adhesion and detachment of microorganisms (Shimoda *et al.*, 2015). ATP is generally unstable because it is quickly degraded by cellular enzymes and has a short half-life; therefore, testing must be conducted shortly after sampling. Moreover, ATP residuals from non-viable microorganisms or organic contaminants, such as disinfectants, may adversely impact the efficacy of the method, whereas the assay is not very sensitive for spore detection and may provide false-negative results (Turner *et al.*, 2010; Alfa *et al.*, 2015). In addition, Gram-negative bacteria are not efficiently detected because of incomplete cell lysis (Turner *et al.*, 2010). Importantly, a reduction of up to 94.36% was documented in the Dictean cave within 5‍ ‍min post-application, whereas the lowest residual metabolic activity equal to 10.80% was noted 24‍ ‍h post-application. Sites 2 and 5 showed slightly higher ATP levels 24‍ ‍h after the essential oil application than at the first time point (5‍ ‍min). This may be explained by the high initial (prior to application) levels of metabolic activity at these specific sites as well as the presence of recalcitrant black biofilms, and underscores the necessity for repeated applications to effectively eliminate the resistant bioburden. Importantly, differences in the sampled surfaces and variability in sample biodiversity may contribute to the observed differences in metabolic activity levels within the different sites belonging to the same cave ecosystem. This variability may also play a significant role in how effectively the applied essential oil formulation interacts with the specific species of microbes present in each sample, as in sites 2 and 5. Following essential oil application, the speleothems exhibited significant changes in their colorimetric properties, including a reduction in green hue and an increase in yellow tones. Additionally, darkening was observed, with the most notable changes occurring in sites 5 and 3. These observations, when considered alongside reductions in the estimated biomass, indicate the effective removal of photosynthetic microflora from the treated sites and suggest that the biocidal application induced measurable shifts in coloration, enhancing natural cave wall colors by reducing green tones and increasing yellow tones, with site 3 showing the most significant changes. The present results are in line with those of previous studies utilizing essential oils in caves or against cave microbial isolates (Argyri *et al.*, 2021; Tsouggou *et al.*, 2023). Specifically, a similar essential oil formulation (a mixture of 0.15% oregano essential oil and 0.4% cinnamon essential oil) has been employed in Petralona cave, resulting in reductions in ATP levels of up to 96% for black and green spots (Tsouggou *et al.*, 2023). However, in that study, the application of the essential oil mixture was preceded by the separate application of oregano and cinnamon essential oils in two successive steps. In addition, measurements were conducted after 3‍ ‍min, which differs from the time points used in the present study (5‍ ‍min and 24‍ ‍h). In another study, oregano essential oil at a concentration of 0.1% (v/v) exhibited biocidal activity *in vitro* against isolates derived from Petralona cave walls (Argyri *et al.*, 2021). Although the concentration of the mixture and the types of essential oils were similar, the present results are not directly comparable due to differences in sampling sites, application protocols, and measurement time points. These variations highlight the importance of site-specific conditions and methodologies when evaluating the efficacy of biocidal treatments. Nevertheless, as a general consensus, metabolic activity was markedly reduced, underscoring the potential efficacy of essential oil-based formulations as biocidal agents.

While the essential oil treatment showed promising short-term efficacy at reducing microbial metabolic activity, it is important to consider potential long-term effects. Concerning its impact on native microbial communities, prolonged exposure to essential oils is not advisable without further study because it may lead to the loss of natural microbial communities that participate in nutrient cycling and other natural processes within the cave ecosystem (Barresi *et al.*, 2017; Di Carlo *et al.*, 2017). In addition, the concentrations of applied formulations need to be kept to a minimum, and applications need to be conducted with long intervals in order to eliminate the risk of unintended consequences, such as the development of resistance. Specifically, the development of resistance to aldehyde biocides, such as cinnamaldehyde, has been reported (Dubois *et al.*, 2025). Nevertheless, the utilization of essential oils holds considerable promise for the prevention of bacterial resistance in general. This may be attributed to the multi-component nature of essential oils, whereas many conventional antimicrobials, by comparison, typically have a single target site (Yap *et al.*, 2014). For example, the combination of Palmarosa essential oil with antifungal agents resulted in increased efficacy, lower dosages, and the mitigation of drug resistance development (Yuan *et al.*, 2024). In this context, the application of a combination of two or more essential oils may prove more efficient at lowering the risk of resistance development. In consideration of potential implications for cave water systems and interconnected environmental factors, the application of essential oils may have long-term cascading effects. One concern is the accumulation of the degrading microbial biomass, while the essential oils themselves may also be transferred to the cave water system via direct run-off, possibly impacting water quality and other cave organisms. To mitigate these potential risks, additional measures may be necessary, such as the cautious neutralization of residual compounds or of the accumulated biomass using conventional chemical agents that have the least ecological impact. Furthermore, an ecotoxicity study is highly recommended using organisms such as *Artemia salina* (brine shrimp) to assess the potential toxicity of essential oils on aquatic life and identify safe application levels. Future research needs to include periodic monitoring to track microbial community shifts, the emergence of resistance, and potential impacts at the ecosystem level. This will allow us to fully comprehend the long-term effects of essential oil application in cave ecosystems.

Even natural biocides will most probably modify the initial microbiota and, thus, a balance between the preservation of original beneficial OTUs and reductions in invading species must be achieved. The careful selection of the biocide type and concentration is important, in conjunction with the preservation of OTUs or their metagenomes in the lab to monitor the short- and long-term effects of biocide application. The promising results of the present study suggest that the applied essential oil mixture may be employed on a broader scale in the Dictean cave as a gentle cleaning agent for the remediation of cave speleothems impacted by microbial colonization. Moreover, this study may serve as a reference point for future research examining microbial diversity after treatment in the Dictean cave. Once the growth of invading microbiota has been restricted, additional measures may be taken to prevent re-colonization of the cave. It is imperative to find a balance between the tourist exploitation of show caves and the preservation of the natural cave environment. This may be achieved by restricting the number of visitors to a level that does not exceed the carrying capacity of the cave, while additional measures, such as limiting natural and/or artificial lighting, may be employed to ensure the sustainability of these unique ecosystems.

## Citation

Martzoukou, O., Oikonomou, A., Amillis, S., and Hatzinikolaou, D. G. (2025) Amplicon Analysis of Dictean Cave Microbial Communities and Essential Oils as a Mild Biocide. *Microbes Environ ***40**: ME24115.

https://doi.org/10.1264/jsme2.ME24115

## Supplementary Material

Supplementary Material

## Figures and Tables

**Table 1.** Macroscopic observation data of sampling sites 1–5, and measurements of CO_2_ concentration, temperature, and relative humidity at nine different locations within the cave.

Sampling site	Macroscopic observation	CO_2_ concentration (ppm)	Temperature (^o^C)	Relative humidity (RH %)
Entrance	—	430	15.3	70
Orifice^1^	—	496	16.1	67
Site 1	Green microflora	445	16.3	65
Site 2	Black biofilms	433	16.4	63
Bridge start	—	438	16.4	64
Bridge end	—	418	16.8	65
Site 3	Green microflora & black biofilms	433	16.9	63
Site 4	Green microflora	481	16.9	61
Site 5	Black biofilms	495	16.9	59
**Average**	—	452±28	16.44±0.49	64±3

^1^ The orifice is defined as the point between the entrance and Site 1.

**Table 2.** Overview of bacterial 16S amplicon ana­lysis results. Abundance: number of different species; Shannon: diversity index; Coverage: percentage of coverage in each sample.

	Site 1	Site 2	Site 3	Site 4	Site 5
Abundance	926	1016	940	864	843
Shannon index	2.17	0.67	3.69	1.79	2.22
Singletons	582	629	464	502	514
Coverage (%)	99.35	99.34	99.36	99.49	99.49

**Table 3.** Relative abundance of abundant (>10%) OTUs belonging to *Pseudomonas* spp. in sites 1–5.

Genbank accession number	Species	Identity (%)	Site 1	Site 2	Site 3	Site 4	Site 5
AB680972	*Pseudomonas fluorescens*	100	35.50	91.89	11.72	67.29	0.55
AB680170	*Pseudomonas synxantha*	100	24.34	0.07	0.16	0.08	0.23
HQ876463	*Pseudomonas fluorescens 2*	100	13.00	0.04	0.05	0.03	0.07
AM293565	*Pseudomonas reinekei*	100	0.36	0.17	1.46	0.18	8.69
AB680483	*Pseudomonas putida*	99.77	0.02	0.03	10.54	0.03	0.02
AB680483	*Pseudomonas putida 2*	100	0.05	0.05	13.73	0.03	0.03

**Table 4.** Overview of eukaryotic (18S) amplicon ana­lysis results. Abundance: number of different species; Shannon: diversity index; Coverage: percentage of coverage in each sample.

	Site 1	Site 2	Site 3	Site 4	Site 5
Abundance	25	4	35	39	12
Shannon index	2.04	1.33	1.6	2.42	0.90
Singletons	549	5	1,548	4,996	2,221
Coverage (%)	98.91	40.00	99.42	99.94	99.91

**Table 5.** Quantitative metabolic activity estimation through ATP measurements prior to and after the application of essential oils at five sampling sites.

Sampling site	Pre-application		Elapsed time: 5‍ ‍min			Elapsed time: 24 h	
	RLU^1^		RLU	% Residual metabolic activity		RLU	% Residual metabolic activity
Site 1	16,209		5,793	35.74		1,827	11.27
Site 2	33,445		1,886	5.64		5,857	17.51
Site 3	19,749		12,843	65.03		2,132	10.80
Site 4	15,741		11,428	72.60		3,161	20.08
Site 5	27,315		6,392	23.40		7,897	28.91
**Average**	22,492		7,668	40.48		4,175	17.71

^1^ Relative luminescence units.

**Table 6.** Assessment of cave surface coloration at five selected sampling sites before and 24‍ ‍h after formulation application based on measured L*a*b chromaticity coordinates.

Sampling site	Before application			After application	
	L	a, b		L	a, b
Site 1	23.7	–0.2, 7.2		23.1	1.5, 9.6
Site 2	29.9	0.6, 5		30.4	2.8, 5.8
Site 3	19.8	–0.7, 3.9		16.6	3.1, 8.6
Site 4	24.9	–0.2, 8.8		23.9	0.1, 9.7
Site 5	49.4	2.7, 9.9		43.3	4.3, 8.7
