# Supplementary material for: Amplicon Analysis of Dictean Cave Microbial Communities and Essential Oils as a Mild Biocide
Source: Microbes Environ. 2025 Sep 25;40(3):ME24115. doi: 10.1264/jsme2.ME24115 (PMC12501871; doi:10.1264/jsme2.ME24115)
Supplement: Supplementary file 1 — Supplementary Material [file 40_24115_s1.pdf]

Amplicon Analysis of Dictean Cave Microbial Communities and Essential Oils as a Mild Biocide

Olga Martzoukou[1]*, Alexandra Oikonomou[2], Sotiris Amillis[1], and Dimitris G. Hatzinikolaou[1]*

[1]*Enzyme and Microbial Biotechnology Unit, Department of Biology, National and Kapodistrian University of Athens, Athens, Greece;* [2]*Ephorate of Palaeoanthropology and Speleology, Hellenic Republic Ministry of Culture, Athens, Greece*

* Corresponding authors: o.martzoukou@biol.uoa.gr , dhatzini@biol.uoa.gr

<u>Supplementary Information</u>

Supplementary Figures

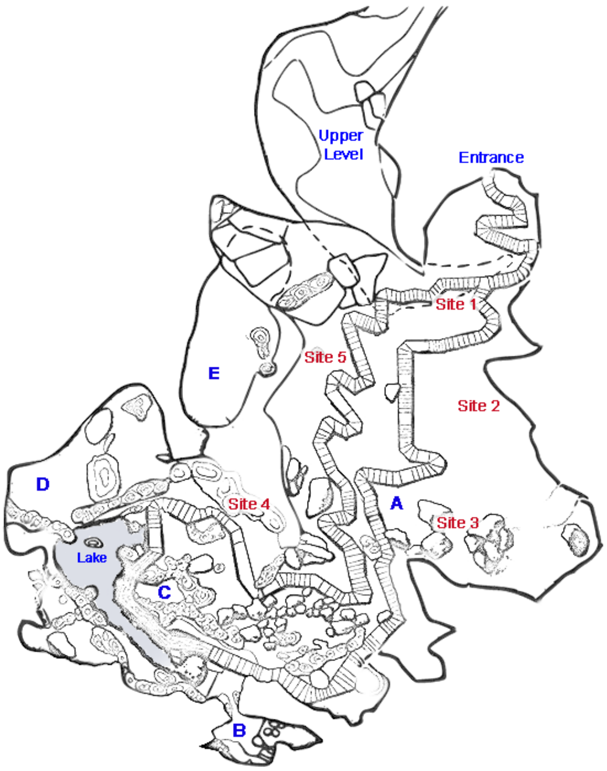

Fig. S1. Schematic representation of Diktaion Andron. Sites 1-5 indicate the sampling sites for this study (red color). Letters A-E indicate the five chambers of the lower level of the cave (blue color). A bridge is situated in Chamber C, in an elevated position above the lake.

Supplementary Tables

Table S1. Relative abundances of OTUs.

Relative Abundances of Unicellular Eukaryotic OTUs (18S)							
**#OTU**	**Genbank Accession Number**	**Taxonomy**	**Site 1**	**Site 2**	**Site 3**	**Site 4**	**Site 5**
OTU444	JQ271779.1.1926	Eukaryota;Amorphea;Amoebozoa;Tubulinea;Tubulinea_group_02_sp._HF3KL	0	0	0	0	5.85321927
OTU744	DQ244015.1.1871	Eukaryota;Amorphea;Obazoa;Opisthokonta;Nucletmycea;Fungi;Blastocladiomycota;Incertae_Sedis;Blastocladiomycetes;Blastocladiales;uncultured_fungus	0	0	3.42377261	0	0
OTU1234	KC841028.1.2436	Eukaryota;Amorphea;Obazoa;Opisthokonta;Nucletmycea;Fungi;Dikarya;Ascomycota;Pezizomycotina;Dothideomycetes;Pleosporales;Phaeosphaeriaceae;Ophiosphaerella;Ophiosphaerella_herpotricha	0	0	0.06459948	0	0
OTU1443	LT558703.1.3546	Eukaryota;Amorphea;Obazoa;Opisthokonta;Nucletmycea;Fungi;Dikarya;Ascomycota;Pezizomycotina;Eurotiomycetes;Chaetothyriales;Trichomeriaceae;Bacillicladium;Bacillicladium_lobatum	4.18943534	0	0.06459948	0	0
OTU1777	LT558703.1.3546	Eukaryota;Amorphea;Obazoa;Opisthokonta;Nucletmycea;Fungi;Dikarya;Ascomycota;Pezizomycotina;Eurotiomycetes;Chaetothyriales;Trichomeriaceae;Bacillicladium;Bacillicladium_lobatum	1.82149362	0	0	0	0
OTU1837	LT558703.1.3546	Eukaryota;Amorphea;Obazoa;Opisthokonta;Nucletmycea;Fungi;Dikarya;Ascomycota;Pezizomycotina;Eurotiomycetes;Chaetothyriales;Trichomeriaceae;Bacillicladium;Bacillicladium_lobatum	0.18214936	0	0	0.18014412	0
OTU816	CACM01000378.6712.8504	Eukaryota;Amorphea;Obazoa;Opisthokonta;Nucletmycea;Fungi;Dikarya;Ascomycota;Pezizomycotina;Leotiomycetes;Erysiphales;Erysiphaceae;Erysiphe;Erysiphe_pisi	0.18214936	0	0	0	0
OTU297	AB013517.1.1784	Eukaryota;Amorphea;Obazoa;Opisthokonta;Nucletmycea;Fungi;Dikarya;Ascomycota;Saccharomycotina;Saccharomycetes;Saccharomycetales;Debaryomycetaceae;Meyerozyma-Candida_clade;[Candida]_carpophila	0	20	0.19379845	0	0
OTU1682	AF026595.1.1777	Eukaryota;Amorphea;Obazoa;Opisthokonta;Nucletmycea;Fungi;Dikarya;Basidiomycota;Agaricomycotina;Agaricomycetes;Agaricales;Pleurotaceae;Pleurotus;Pleurotus_tuber-regium	0	0	0.19379845	0.1601281	0
OTU285	DQ465339.1.1785	Eukaryota;Amorphea;Obazoa;Opisthokonta;Nucletmycea;Fungi;Dikarya;Basidiomycota;Agaricomycotina;Agaricomycetes;Agaricales;Psathyrellaceae;Psathyrella;Psathyrella_candolleana	1.27504554	0	0.90439276	0.34027222	0.63034669
OTU893	DQ465339.1.1785	Eukaryota;Amorphea;Obazoa;Opisthokonta;Nucletmycea;Fungi;Dikarya;Basidiomycota;Agaricomycotina;Agaricomycetes;Agaricales;Psathyrellaceae;Psathyrella;Psathyrella_candolleana	0	0	0.06459948	0	0.04502476
OTU858	AF026629.1.1777	Eukaryota;Amorphea;Obazoa;Opisthokonta;Nucletmycea;Fungi;Dikarya;Basidiomycota;Agaricomycotina;Agaricomycetes;Polyporales;Ganoderma;Ganoderma_australe	0	0	0.06459948	0.02001601	0
OTU488	AB032615.1.1782	Eukaryota;Amorphea;Obazoa;Opisthokonta;Nucletmycea;Fungi;Dikarya;Basidiomycota;Agaricomycotina;Tremellomycetes;Filobasidiales;Filobasidiaceae;Naganishia;Naganishia_albidosimilis	0	0	0.06459948	0	4.09725349
OTU9	AAYY01000016.137081.138848	Eukaryota;Amorphea;Obazoa;Opisthokonta;Nucletmycea;Fungi;Dikarya;Basidiomycota;Tstilaginomycotina;Malasseziomycetes;Malasseziales;Malasseziaceae;Malassezia;Malassezia_globosa_CBS_7966	0.72859745	20	0.12919897	0.02001601	0.40522287
OTU12	AAXK01002636.1452.3220	Eukaryota;Amorphea;Obazoa;Opisthokonta;Nucletmycea;Fungi;Dikarya;Basidiomycota;Tstilaginomycotina;Malasseziomycetes;Malasseziales;Malasseziaceae;Malassezia;Malassezia_restricta_CBS_7877	0.36429872	0	0.19379845	0.02001601	0.13507429
OTU18	MH697739.1.1812	Eukaryota;Amorphea;Obazoa;Opisthokonta;Nucletmycea;Fungi;Dikarya;Basidiomycota;Tstilaginomycotina;Malasseziomycetes;Malasseziales;Malasseziaceae;Malassezia;Polyrhabdina_sp._SR-2018a	0	0	0.19379845	0	0
OTU77	KC670327.1.1784	Eukaryota;Amorphea;Obazoa;Opisthokonta;Nucletmycea;Fungi;Dikarya;Basidiomycota;Tstilaginomycotina;Malasseziomycetes;Malasseziales;Malasseziaceae;Malassezia;uncultured_fungus	0	0	0.06459948	0	0
OTU184	ADAG01001078.1100.2889	Eukaryota;Amorphea;Obazoa;Opisthokonta;Nucletmycea;Fungi;Mucoromycota;Mortierellomycotina;Incertae_Sedis;Mortierellales;Mortierellaceae;Mortierella;Mortierella_alpina_ATCC_32222	19.4899818	40	11.4341085	0.66052842	1.53084196
OTU1006	ADAG01001078.1100.2889	Eukaryota;Amorphea;Obazoa;Opisthokonta;Nucletmycea;Fungi;Mucoromycota;Mortierellomycotina;Incertae_Sedis;Mortierellales;Mortierellaceae;Mortierella;Mortierella_alpina_ATCC_32222	2.73224044	20	1.22739018	0.06004804	0.04502476
OTU249	EF023463.1.1793	Eukaryota;Amorphea;Obazoa;Opisthokonta;Nucletmycea;Fungi;Mucoromycota;Mortierellomycotina;Incertae_Sedis;Mortierellales;Mortierellaceae;Mortierella;uncultured_Eimeriidae	42.2586521	0	0	0	0
OTU1368	EF023463.1.1793	Eukaryota;Amorphea;Obazoa;Opisthokonta;Nucletmycea;Fungi;Mucoromycota;Mortierellomycotina;Incertae_Sedis;Mortierellales;Mortierellaceae;Mortierella;uncultured_Eimeriidae	4.73588342	0	0	0	0
OTU30	EF023210.1.1786	Eukaryota;Amorphea;Obazoa;Opisthokonta;Nucletmycea;Fungi;Mucoromycota;Mortierellomycotina;Incertae_Sedis;Mortierellales;Mortierellaceae;Mortierella;uncultured_Nucleariidae	0	0	53.6175711	0	77.8027915
OTU243	EF023210.1.1786	Eukaryota;Amorphea;Obazoa;Opisthokonta;Nucletmycea;Fungi;Mucoromycota;Mortierellomycotina;Incertae_Sedis;Mortierellales;Mortierellaceae;Mortierella;uncultured_Nucleariidae	0	0	5.23255814	0	7.24898694
OTU1375	FR865548.2.1767	Eukaryota;Archaeplastida;Chloroplastida;Chlorophyta;Chlorophyceae;Chlamydomonadales;Chlamydomonas;Chlamydomonas_sp._CCAP_11/153	0	0	0.90439276	0.18014412	0
OTU449	KR869869.1.1793	Eukaryota;Archaeplastida;Chloroplastida;Chlorophyta;Chlorophyceae;Incertae_Sedis;Jenufa;Jenufa_aeroterrestrica	4.55373406	0	1.74418605	1.14091273	0
OTU288	KR869869.1.1793	Eukaryota;Archaeplastida;Chloroplastida;Chlorophyta;Chlorophyceae;Incertae_Sedis;Jenufa;Jenufa_aeroterrestrica	0.91074681	0	1.80878553	4.10328263	0
OTU128	KR869869.1.1793	Eukaryota;Archaeplastida;Chloroplastida;Chlorophyta;Chlorophyceae;Incertae_Sedis;Jenufa;Jenufa_aeroterrestrica	2.9143898	0	0	9.88791033	0
OTU45	KR869869.1.1793	Eukaryota;Archaeplastida;Chloroplastida;Chlorophyta;Chlorophyceae;Incertae_Sedis;Jenufa;Jenufa_aeroterrestrica	6.92167577	0	0.71059432	34.327462	0
OTU933	KR869869.1.1793	Eukaryota;Archaeplastida;Chloroplastida;Chlorophyta;Chlorophyceae;Incertae_Sedis;Jenufa;Jenufa_aeroterrestrica	1.4571949	0	0.19379845	0.66052842	0
OTU328	AB257663.1.1778	Eukaryota;Archaeplastida;Chloroplastida;Chlorophyta;Chlorophyceae;Incertae_Sedis;uncultured_Chlorophyta	0.54644809	0	0.06459948	3.62289832	0
OTU1678	AB257663.1.1778	Eukaryota;Archaeplastida;Chloroplastida;Chlorophyta;Chlorophyceae;Incertae_Sedis;uncultured_Chlorophyta	0	0	0.12919897	0.28022418	0
OTU1198	AB257663.1.1778	Eukaryota;Archaeplastida;Chloroplastida;Chlorophyta;Chlorophyceae;Incertae_Sedis;uncultured_Chlorophyta	0.18214936	0	0	0.46036829	0
OTU751	AB257663.1.1778	Eukaryota;Archaeplastida;Chloroplastida;Chlorophyta;Chlorophyceae;Incertae_Sedis;uncultured_Chlorophyta	0	0	0	0.98078463	0
OTU455	GQ462996.1.1797	Eukaryota;Archaeplastida;Chloroplastida;Chlorophyta;Chlorophyceae;Incertae_Sedis;uncultured_eukaryote	0.18214936	0	0.7751938	1.78142514	0
OTU890	GQ462996.1.1797	Eukaryota;Archaeplastida;Chloroplastida;Chlorophyta;Chlorophyceae;Incertae_Sedis;uncultured_eukaryote	0	0	0.06459948	0.98078463	0
OTU945	GT067989.1.1789	Eukaryota;Archaeplastida;Chloroplastida;Chlorophyta;Chlorophyceae;uncultured_Atractomorpha	0.18214936	0	0.06459948	0.74059247	0
OTU483	LC109073.1.1820	Eukaryota;Archaeplastida;Chloroplastida;Chlorophyta;Trebouxiophyceae;uncultured_eukaryote	0	0	0.64599483	1.52121697	0
OTU199	LC109073.1.1820	Eukaryota;Archaeplastida;Chloroplastida;Chlorophyta;Trebouxiophyceae;uncultured_eukaryote	0	0	1.9379845	5.90472378	0
OTU137	LC109073.1.1820	Eukaryota;Archaeplastida;Chloroplastida;Chlorophyta;Trebouxiophyceae;uncultured_eukaryote	0	0	4.84496124	7.62610088	0
OTU66	LC109073.1.1820	Eukaryota;Archaeplastida;Chloroplastida;Chlorophyta;Trebouxiophyceae;uncultured_eukaryote	0.36429872	0	7.49354005	14.8919135	0
OTU1051	LC109073.1.1820	Eukaryota;Archaeplastida;Chloroplastida;Chlorophyta;Trebouxiophyceae;uncultured_eukaryote	0	0	0.25839793	0.52041633	0
OTU725	LC109073.1.1820	Eukaryota;Archaeplastida;Chloroplastida;Chlorophyta;Trebouxiophyceae;uncultured_eukaryote	0	0	0.25839793	0.92073659	0
OTU1494	KJ763296.1.1794	Eukaryota;Cryptophyceae;Kathablepharidae;Leucocryptos;uncultured_eukaryote	0	0	0	0.34027222	0
OTU1069	GT320598.1.2010	Eukaryota;Discoba;Discicristata;Heterolobosea;Tetramitia;Naegleria;Naegleria_sp._COHH_64	0	0	0	0	2.11616389
OTU1211	DQ462458.1.1771	Eukaryota;SAR;Alveolata;Apicomplexa;Conoidasida;Gregarinasina;Eugregarinorida;Ascogregarina;Ascogregarina_sp._ex_Ochlerotatus_japonicus	0	0	0	0.70056045	0
OTU782	EF024723.1.1773	Eukaryota;SAR;Alveolata;Apicomplexa;Conoidasida;Gregarinasina;Eugregarinorida;Gregarina;Heterocapsaceae_environmental_sample	0.18214936	0	0	1.14091273	0
OTU1521	EF024723.1.1773	Eukaryota;SAR;Alveolata;Apicomplexa;Conoidasida;Gregarinasina;Eugregarinorida;Gregarina;Heterocapsaceae_environmental_sample	2.55009107	0	0	0.06004804	0
OTU448	HM536152.1.1799	Eukaryota;SAR;Rhizaria;Cercozoa;Cercomonadidae;Eocercomonas;Eocercomonas_sp._HFCC_907	0	0	0	0.04003203	0
OTU474	AB695523.1.1762	Eukaryota;SAR;Rhizaria;Cercozoa;Cercomonadidae;Paracercomonas;uncultured_eukaryote	0.36429872	0	0	0	0
OTU1319	FPLS01025462.9.1376	Eukaryota;SAR;Rhizaria;Cercozoa;Imbricatea;Silicofilosea;Euglyphida;Euglyphidae;Euglypha;metagenome	0	0	0	0	0.09004953
OTU1779	EF023834.1.1804	Eukaryota;SAR;Rhizaria;Cercozoa;Thecofilosea;Cryomonadida;Rhizaspididae;Rhogostoma;uncultured_eukaryote	0	0	0.58139535	0	0
OTU1809	AB022103.1.1787	Eukaryota;SAR;Stramenopiles;Labyrinthulomycetes;Thraustochytriaceae;Aplanochytrium;Aplanochytrium_kerguelense	0	0	0	0.24019215	0
OTU1554	AB022103.1.1787	Eukaryota;SAR;Stramenopiles;Labyrinthulomycetes;Thraustochytriaceae;Aplanochytrium;Aplanochytrium_kerguelense	0.72859745	0	0	0.36028823	0
OTU608	AY916573.1.1691	Eukaryota;SAR;Stramenopiles;Labyrinthulomycetes;Thraustochytriaceae;Aplanochytrium;uncultured_eukaryote	0	0	0	1.54123299	0
OTU558	AB695533.1.1763	Eukaryota;SAR;Stramenopiles;Labyrinthulomycetes;Thraustochytriaceae;Aplanochytrium;uncultured_eukaryote	0	0	0	1.74139311	0
OTU1465	AY916573.1.1691	Eukaryota;SAR;Stramenopiles;Labyrinthulomycetes;Thraustochytriaceae;Aplanochytrium;uncultured_eukaryote	0	0	0	0.30024019	0
OTU1385	KJ961658.1.1764	Eukaryota;SAR;Stramenopiles;Ochrophyta;Diatomea;Bacillariophytina;Bacillariophyceae;Amphora;Amphora_sp._38	0	0	0	0.34027222	0
OTU824	AJ243063.1.1751	Eukaryota;SAR;Stramenopiles;Ochrophyta;Diatomea;Bacillariophytina;Bacillariophyceae;Sellaphoraceae;Eolimna;Eolimna_minima	0	0	0.3875969	1.20096077	0

Relative Abundances of Common and Abundant Bacterial OTUs (> 1%)							
**#OTU**	**Genbank Accession Number**	**Taxonomy**	**Site 1**	**Site 2**	**Site 3**	**Site 4**	**Site 5**
OTU20	AJ609630.1.1468	Bacteria;Actinobacteriota;Actinobacteria;Micrococcales;Micrococcaceae;Glutamicibacter;Glutamicibacter_bergerei	0.02216607	0.08237754	0.01663917	10.9056716	0.01184799
OTU84	AF493635.1.1300	Bacteria;Bacteroidota;Bacteroidia;Flavobacteriales;Flavobacteriaceae;Flavobacterium;Flavobacterium_sp._EP030	0.00221661	0	3.64675051	0.00102209	0
OTU72	AM934647.1.1486	Bacteria;Bacteroidota;Bacteroidia;Flavobacteriales;Flavobacteriaceae;Flavobacterium;Flavobacterium_sp._WB2.3-46	0.00221661	0.02534694	0.00554639	0.00204417	3.0538195
OTU36	HQ120546.1.1496	Bacteria;Bacteroidota;Bacteroidia;Flavobacteriales;Flavobacteriaceae;Flavobacterium;uncultured_bacterium	0.01108303	0.00422449	0.10538138	0.00613252	6.73459514
OTU75	FJ535179.1.1478	Bacteria;Bacteroidota;Bacteroidia;Flavobacteriales;Flavobacteriaceae;Flavobacterium;uncultured_Flavobacteriia_bacterium	0	0.00105612	3.67864224	0.00204417	0.02369598
OTU186	KT719559.1.1407	Bacteria;Firmicutes;Bacilli;Bacillales;Bacillaceae;Bacillus;Bacillus_soli	0.0011083	0	0.00693299	1.44625354	0
OTU13	KF911258.1.1474	Bacteria;Firmicutes;Bacilli;Bacillales;Planococcaceae;Sporosarcina;uncultured_bacterium	0.07979785	0.01056122	0.34387609	0.29844949	15.6018285
OTU50	KF911258.1.1474	Bacteria;Firmicutes;Bacilli;Bacillales;Planococcaceae;Sporosarcina;uncultured_bacterium	0.00443321	0.00422449	0.00831958	0.01022087	5.37800026
OTU56	AB360546.1.1550	Bacteria;Firmicutes;Bacilli;Paenibacillales;Paenibacillaceae;Paenibacillus;Paenibacillus_macquariensis_subsp._defensor	0.00775812	0.02640306	4.37748721	0.81971402	2.46833131
OTU174	AB360546.1.1550	Bacteria;Firmicutes;Bacilli;Paenibacillales;Paenibacillaceae;Paenibacillus;Paenibacillus_macquariensis_subsp._defensor	0.0011083	0.00105612	1.46840638	0.29231697	0.00197467
OTU130	DQ444978.1.1416	Bacteria;Firmicutes;Bacilli;Paenibacillales;Paenibacillaceae;Paenibacillus;Paenibacillus_sp._Eur1_9.9	0.00886643	0.00422449	0.38131422	2.15660422	0.00394933
OTU151	JQ396606.1.1540	Bacteria;Firmicutes;Bacilli;Paenibacillales;Paenibacillaceae;Paenibacillus;Paenibacillus_sp._MN8-13	1.83756705	0.00105612	0.00970618	0.24427887	0.00394933
OTU192	DQ177465.1.1519	Bacteria;Firmicutes;Bacilli;Paenibacillales;Paenibacillaceae;Paenibacillus;Paenibacillus_sp._Tibet-IB15	0.0011083	0.00105612	1.64589082	0.08585533	0
OTU70	KT179367.1.1485	Bacteria;Proteobacteria;Gammaproteobacteria;Enterobacterales;Enterobacteriaceae;Buttiauxella;Buttiauxella_brennerae	4.5518021	0.01056122	0.00831958	0.00919879	0.00493666
OTU137	KT179367.1.1485	Bacteria;Proteobacteria;Gammaproteobacteria;Enterobacterales;Enterobacteriaceae;Buttiauxella;Buttiauxella_brennerae	1.9195815	0.00739286	0.00554639	0.00408835	0.005924
OTU184	CP016889.1470665.1472216	Bacteria;Proteobacteria;Gammaproteobacteria;Enterobacterales;Erwiniaceae;Pantoea;Pantoea_agglomerans	0.01884116	1.09942336	0.0013866	0.00102209	0
OTU1	AB680972.1.1462	Bacteria;Proteobacteria;Gammaproteobacteria;Pseudomonadales;Pseudomonadaceae;Pseudomonas;Pseudomonas_fluorescens	35.5044997	91.8932049	11.7181325	67.2860516	0.54599489
OTU16	HQ876463.1.1508	Bacteria;Proteobacteria;Gammaproteobacteria;Pseudomonadales;Pseudomonadaceae;Pseudomonas;Pseudomonas_fluorescens	13.000399	0.03696428	0.0499175	0.03168471	0.06516395
OTU60	DQ981457.1.1418	Bacteria;Proteobacteria;Gammaproteobacteria;Pseudomonadales;Pseudomonadaceae;Pseudomonas;Pseudomonas_fluorescens	0.01440794	0.01161735	1.76513817	0.02044175	3.40827187
OTU53	AB680165.1.1462	Bacteria;Proteobacteria;Gammaproteobacteria;Pseudomonadales;Pseudomonadaceae;Pseudomonas;Pseudomonas_fluorescens	5.18242674	0.01795408	0.18719062	0.01022087	0.25275713
OTU86	ET594555.1.1445	Bacteria;Proteobacteria;Gammaproteobacteria;Pseudomonadales;Pseudomonadaceae;Pseudomonas;Pseudomonas_fulva	0.04322383	0.09399489	5.41604848	0.10629708	0.01382266
OTU90	AB681347.1.1462	Bacteria;Proteobacteria;Gammaproteobacteria;Pseudomonadales;Pseudomonadaceae;Pseudomonas;Pseudomonas_gessardii	0.02881589	0.03062755	4.41631193	0.04599393	0.01382266
OTU22	AB680483.1.1462	Bacteria;Proteobacteria;Gammaproteobacteria;Pseudomonadales;Pseudomonadaceae;Pseudomonas;Pseudomonas_putida	0.04654874	0.04646938	13.7328582	0.03168471	0.03159464
OTU31	AB680483.1.1462	Bacteria;Proteobacteria;Gammaproteobacteria;Pseudomonadales;Pseudomonadaceae;Pseudomonas;Pseudomonas_putida	0.02105776	0.02957143	10.5367518	0.02861844	0.02073398
OTU3	AM293565.1.1492	Bacteria;Proteobacteria;Gammaproteobacteria;Pseudomonadales;Pseudomonadaceae;Pseudomonas;Pseudomonas_reinekei	0.36463182	0.1700357	1.4587002	0.17579902	48.6883287
OTU80	AB051697.1.1445	Bacteria;Proteobacteria;Gammaproteobacteria;Pseudomonadales;Pseudomonadaceae;Pseudomonas;Pseudomonas_sp._LAB-20	0.12302168	0.17426019	4.83367767	0.12980509	0.01678465
OTU279	DQ453810.1.1496	Bacteria;Proteobacteria;Gammaproteobacteria;Pseudomonadales;Pseudomonadaceae;Pseudomonas;Pseudomonas_sp._m1(2006)	0.00554152	0.00316837	1.37550437	0.00511044	0.00197467
OTU357	DQ453810.1.1496	Bacteria;Proteobacteria;Gammaproteobacteria;Pseudomonadales;Pseudomonadaceae;Pseudomonas;Pseudomonas_sp._m1(2006)	0.00443321	0.00211224	1.00944273	0.00204417	0.00098733
OTU5603	MK038867.1.1438	Bacteria;Proteobacteria;Gammaproteobacteria;Pseudomonadales;Pseudomonadaceae;Pseudomonas;Pseudomonas_stutzeri	1.79766813	0.00211224	0.00277319	0.00102209	0.00197467
OTU5	AB680170.1.1462	Bacteria;Proteobacteria;Gammaproteobacteria;Pseudomonadales;Pseudomonadaceae;Pseudomonas;Pseudomonas_synxantha	24.3383429	0.06759183	0.15945867	0.07563446	0.23498514
OTU131	AF320989.1.1452	Bacteria;Proteobacteria;Gammaproteobacteria;Pseudomonadales;Pseudomonadaceae;Pseudomonas;Pseudomonas_tolaasii	4.18052046	0.10138774	0.04021132	0.11038543	0.01086066
OTU522	ET512011.1.1361	Bacteria;Proteobacteria;Gammaproteobacteria;Pseudomonadales;Pseudomonadaceae;Pseudomonas;uncultured_bacterium	1.16704349	0.00316837	0.00277319	0.00102209	0.02270865
OTU202	KX363701.1.1459	Bacteria;Proteobacteria;Gammaproteobacteria;Pseudomonadales;Pseudomonadaceae;Pseudomonas;uncultured_Pseudomonas_sp.	0.00886643	0.00633673	1.92598344	0.00408835	0.00493666
OTU270	KX363701.1.1459	Bacteria;Proteobacteria;Gammaproteobacteria;Pseudomonadales;Pseudomonadaceae;Pseudomonas;uncultured_Pseudomonas_sp.	0.00997473	0.00211224	1.39491673	0.00306626	0.00197467

Relative Abundances of all Bacterial OTUs (16S)								
#OTU	Genbank Accession Number	Taxonomy	%identity	Site 1	Site 2	Site 3	Site 4	Site 5
OTU1	AB680972.1.1462	Bacteria;Proteobacteria;Gammaproteobacteria;Pseudomonadales;Pseudomonadaceae;Pseudomonas;Pseudomonas_fluorescens	100	35.5044997	91.8932049	11.71927	67.2860516	0.54599489
OTU10	MF040478.1.1461	Bacteria;Proteobacteria;Alphaproteobacteria;Acetobacterales;Acetobacteraceae;Roseomonas;uncultured_bacterium	99.25	0.0332491	0.05069387	0.04160195	0.0480381	0.03258197
OTU100	AJ863252.1.1485	Bacteria;Nitrospirota;Nitrospiria;Nitrospirales;Nitrospiraceae;Nitrospira;uncultured_bacterium	86.21	0.0011083	0.00528061	0	0.00408835	0.00098733
OTU1001	AJ440995.1.1474	Bacteria;Proteobacteria;Gammaproteobacteria;Burkholderiales;Comamonadaceae;Hydrogenophaga;Antarctic_bacterium_R-8890	98.36	0	0.00211224	0	0.00102209	0.00098733
OTU1004	HM187074.1.1485	Bacteria;Nitrospirota;Leptospirillia;Leptospirillales;Leptospirillaceae;Leptospirillum;uncultured_bacterium	98.83	0	0	0	0.00102209	0
OTU1007	FJ628290.1.1438	Bacteria;Patescibacteria;Gracilibacteria;Candidatus_Peribacteria;uncultured_bacterium	85.89	0	0.00211224	0	0	0
OTU101	MNVX01000029.4758.6202_	Archaea;Nanoarchaeota;Nanoarchaeia;Woesearchaeales;GW2011_GWC1_47_15;Candidatus_Pacearchaeota_archaeon_CG1_02_32_21	83.07	0	0.00316837	0	0.00102209	0.002962
OTU1010	FPLL01003665.15.1315	Bacteria;Bacteroidota;Bacteroidia;Cytophagales;Microscillaceae;Hassallia;metagenome	100	0	0	0.00138673	0	0
OTU1019	AB681203.1.1455	Bacteria;Bacteroidota;Bacteroidia;Flavobacteriales;Flavobacteriaceae;Salegentibacter;Salegentibacter_mishustinae	100	0.0011083	0	0	0.00102209	0.00197467
OTU102	NFTV01000002.590109.591646	Bacteria;Bacteroidota;Bacteroidia;Chitinophagales;Chitinophagaceae;Sediminibacterium;Chitinophagaceae_bacterium_IBVTCB2	100	0.00443321	0.00422449	0.00277346	0.00102209	0.00197467
OTU1023	ACGD01000048.119.1624	Bacteria;Actinobacteriota;Actinobacteria;Corynebacteriales;Corynebacteriaceae;Corynebacterium;Corynebacterium_accolens_ATCC_49725	100	0.0011083	0	0.00138673	0	0.00098733
OTU1024	AB514517.1.1487	Bacteria;Actinobacteriota;Actinobacteria;Pseudonocardiales;Pseudonocardiaceae;Actinomycetospora;Actinomycetospora_chibensis	99.52	0	0.00105612	0	0	0
OTU1029	AB680133.1.1478	Bacteria;Firmicutes;Bacilli;Staphylococcales;Staphylococcaceae;Staphylococcus;Staphylococcus_sp._NBRC_3762	100	0	0	0	0	0.00098733
OTU103	EF188441.1.1496	Bacteria;Actinobacteriota;Actinobacteria;Corynebacteriales;Corynebacteriaceae;Lawsonella;uncultured_actinobacterium	100	0.0011083	0.00211224	0.00554693	0.00408835	0.00098733
OTU1030	DQ372983.1.1446	Bacteria;Proteobacteria;Gammaproteobacteria;Burkholderiales;Comamonadaceae;Hydrogenophaga;Hydrogenophaga_caeni	99.3	0	0	0	0	0.00197467
OTU1031	HQ904237.1.1498	Bacteria;Proteobacteria;Alphaproteobacteria;Rhodobacterales;Rhodobacteraceae;Rhodobacter;uncultured_bacterium	99.5	0	0	0	0.00102209	0.00098733
OTU1032	AB637159.1.1509	Bacteria;Firmicutes;Bacilli;Bacillales;Bacillaceae;Bacillus;uncultured_bacterium	99.77	0	0	0	0.00102209	0
OTU1033	KF836147.1.1531	Bacteria;Nitrospirota;Leptospirillia;Leptospirillales;Leptospirillaceae;Leptospirillum;uncultured_bacterium	91.59	0	0	0	0	0.00098733
OTU1034	AB511008.1.1436	Bacteria;Bdellovibrionota;Bdellovibrionia;Bdellovibrionales;Bdellovibrionaceae;Bdellovibrio;uncultured_bacterium	98.27	0	0	0	0.00102209	0
OTU1035	ET573788.1.1421	Bacteria;Actinobacteriota;Actinobacteria;Micrococcales;Microbacteriaceae;Microbacterium;Microbacterium_lacticum	100	0.0011083	0.00105612	0.00138673	0.00613252	0
OTU1036	AB071955.1.1454	Bacteria;Proteobacteria;Alphaproteobacteria;Sphingomonadales;Sphingomonadaceae;Sphingomonas;Sphingomonas_yabuuchiae	100	0	0.00105612	0	0	0.00098733
OTU1038	AY734239.1.1379	Bacteria;Nitrospirota;Leptospirillia;Leptospirillales;Leptospirillaceae;Leptospirillum;uncultured_bacterium	94.39	0.0011083	0.00211224	0	0	0.00098733
OTU1040	GQ083769.1.1348	Bacteria;Firmicutes;Clostridia;Peptostreptococcales-Tissierellales;Family_XI;Anaerococcus;uncultured_bacterium	100	0	0	0	0.00102209	0
OTU1041	JX105619.1.1337	Bacteria;Patescibacteria;Parcubacteria;Candidatus_Adlerbacteria;uncultured_bacterium	92.31	0	0.00105612	0	0	0
OTU1045	AB425060.1.1551	Bacteria;Bdellovibrionota;Oligoflexia;0319-6G20;uncultured_delta_proteobacterium	89.02	0	0.00105612	0	0	0
OTU1046	KM410599.1.1251	Bacteria;Patescibacteria;Parcubacteria;Candidatus_Kaiserbacteria;uncultured_prokaryote	97.04	0	0.00105612	0	0	0
OTU1048	ET801343.1.1495	Bacteria;Proteobacteria;Gammaproteobacteria;Burkholderiales;Comamonadaceae;Limnohabitans;uncultured_bacterium	99.77	0.0011083	0.00211224	0.00138673	0	0.00098733
OTU1049	AJ616763.1.1501	Bacteria;Actinobacteriota;Actinobacteria;Micrococcales;Micrococcaceae;Paeniglutamicibacter;Paeniglutamicibacter_psychrophenolicus	100	0	0	0.33142889	0.00204417	0.00197467
OTU1050	FPLK01001140.10.1511	Bacteria;Actinobacteriota;Actinobacteria;PeM15;metagenome	100	0.0011083	0	0	0	0.00098733
OTU1051	HM129552.1.1414	Bacteria;Cyanobacteria;Cyanobacteriia;Cyanobacteriales;Microcystaceae;Snowella_0TT37S04;uncultured_bacterium	100	0.0011083	0	0.00138673	0	0
OTU1055	ET050754.1.1409	Bacteria;Proteobacteria;Alphaproteobacteria;Sphingomonadales;Sphingomonadaceae;Sphingomonas;uncultured_Alphaproteobacteria_bacterium	99.5	0.0011083	0.00105612	0.0041602	0	0.00098733
OTU1057	ET937855.1.1501	Bacteria;Proteobacteria;Gammaproteobacteria;Burkholderiales;Gallionellaceae;Sideroxydans;uncultured_bacterium	99.77	0.0011083	0	0	0.00102209	0
OTU1058	MF942640.1.1495	Bacteria;Verrucomicrobiota;Omnitrophia;Omnitrophales;Omnitrophaceae;Candidatus_Omnitrophus;uncultured_bacterium	91.38	0	0	0	0.00102209	0
OTU1059	FPLK01002478.10.1517	Bacteria;Proteobacteria;Gammaproteobacteria;Burkholderiales;Alcaligenaceae;GKS98_freshwater_group;metagenome	100	0	0	0.00138673	0	0.00098733
OTU106	AF277208.1.1446	Bacteria;Actinobacteriota;Actinobacteria;Corynebacteriales;Nocardiaceae;Nocardia;Nocardia_sp._R112	100	0.00332491	0.00316837	0.00832039	0	0.00197467
OTU1062	EF444635.1.1169	Archaea;Nanoarchaeota;Nanoarchaeia;Woesearchaeales;uncultured_archaeon	79.17	0	0.00105612	0	0	0
OTU1063	FJ976603.1.1389	Bacteria;Proteobacteria;Gammaproteobacteria;Pseudomonadales;Moraxellaceae;Acinetobacter;Acinetobacter_johnsonii	99.77	0.0011083	0.00105612	0.00138673	0.00204417	0
OTU1064	HM186657.1.1389	Bacteria;Nitrospirota;Leptospirillia;Leptospirillales;Leptospirillaceae;Leptospirillum;uncultured_bacterium	87.38	0.0011083	0	0	0	0.00098733
OTU1065	JQ278884.1.1533	Bacteria;Verrucomicrobiota;Omnitrophia;Omnitrophales;Omnitrophaceae;Candidatus_Omnitrophus;uncultured_bacterium	91.86	0	0	0	0.00102209	0
OTU1067	HQ436493.1.1435	Bacteria;Proteobacteria;Alphaproteobacteria;Sphingomonadales;Sphingomonadaceae;Sphingorhabdus;Sphingorhabdus_wooponensis	99.75	0.00221661	0.00105612	0.01525405	0.00204417	0.00394933
OTU107	AB355617.1.1400	Bacteria;Firmicutes;Bacilli;Lactobacillales;Streptococcaceae;Streptococcus;Streptococcus_oralis	100	0.00775812	0	0.0041602	0.00408835	0.002962
OTU1070	AJ867908.1.1552	Bacteria;Verrucomicrobiota;Verrucomicrobiae;Opitutales;Opitutaceae;Cephaloticoccus;uncultured_bacterium	96.95	0	0	0.00277346	0	0
OTU1071	CP009281.5845090.5846653	Bacteria;Firmicutes;Bacilli;Paenibacillales;Paenibacillaceae;Paenibacillus;Paenibacillus_sp._FSL_R5-0345	99.53	0	0	0	0.18295363	0
OTU1072	AF533356.1.1433	Bacteria;Proteobacteria;Alphaproteobacteria;Acetobacterales;Acetobacteraceae;Roseomonas;Roseomonas_genomospecies_5	100	0.00221661	0.00211224	0	0	0
OTU1078	KT122121.1.1518	Bacteria;uncultured_Thioflavicoccus_sp.	79.86	0	0	0	0.00204417	0
OTU1080	FPLS01012850.10.1471	Bacteria;Proteobacteria;Alphaproteobacteria;Caulobacterales;Caulobacteraceae;Caulobacter;metagenome	99.5	0.00221661	0	0	0.00102209	0
OTU1081	ET196330.1.1411	Bacteria;Proteobacteria;Alphaproteobacteria;Caulobacterales;Hyphomonadaceae;Litorimonas;Arctic_bacterium_NP26	99.25	0	0	0.00138673	0.00102209	0
OTU1084	DQ450182.1.1483	Bacteria;Bacteroidota;Bacteroidia;Flavobacteriales;Crocinitomicaceae;Fluviicola;uncultured_proteobacterium	100	0	0	0	0	0.00098733
OTU1086	KY356865.1.937_	Archaea;Nanoarchaeota;Nanoarchaeia;Woesearchaeales;GW2011_GWC1_47_15;uncultured_archaeon	88.07	0	0.00105612	0	0	0
OTU1087	CP006608.270249.271746	Bacteria;Proteobacteria;Gammaproteobacteria;Enterobacterales;Enterobacteriaceae;Salmonella;Salmonella_bongori_N268-08	99.77	0.00221661	0.00105612	0	0	0.00098733
OTU1088	AF069742.1.1481	Bacteria;Firmicutes;Clostridia;Clostridiales;Clostridiaceae;Clostridium_sensu_stricto_2;Clostridium_frigidicarnis	100	0	0	0.00138673	0.18397572	0
OTU109	KM035956.1.1424	Bacteria;Bacteroidota;Bacteroidia;Flavobacteriales;Flavobacteriaceae;Flavobacterium;Flavobacterium_sp._THG-DN6.19	100	0.00443321	0.00528061	0.01664078	0.00511044	0.00394933
OTU1091	KX123380.1.2523	Bacteria;Patescibacteria;Parcubacteria;Candidatus_Jorgensenbacteria;Candidatus_Jorgensenbacteria_bacterium_GW2011_GWA2_45_13	83.94	0	0.00105612	0	0	0
OTU1093	AB680534.1.1414	Bacteria;Firmicutes;Bacilli;Lactobacillales;Streptococcaceae;Streptococcus;Streptococcus_salivarius	100	0	0	0	0.00102209	0
OTU1094	CP010350.2826891.2828435	Bacteria;Proteobacteria;Gammaproteobacteria;Pseudomonadales;Moraxellaceae;Acinetobacter;Acinetobacter_johnsonii_XBB1	100	0.0011083	0	0.00138673	0	0
OTU1095	AB360546.1.1550	Bacteria;Firmicutes;Bacilli;Paenibacillales;Paenibacillaceae;Paenibacillus;Paenibacillus_macquariensis_subsp._defensor	99.53	0	0.00105612	0.21494342	0.05008228	0
OTU1096	FJ598382.1.1459	Bacteria;Proteobacteria;Gammaproteobacteria;Thiotrichales;Thiotrichaceae;Thiothrix;uncultured_bacterium	99	0	0.00105612	0	0	0
OTU1098	AB021194.1.1526	Bacteria;Firmicutes;Bacilli;Bacillales;Bacillaceae;Bacillus;Bacillus_niacini	100	0	0.00105612	0.00554693	0.05825898	0.002962
OTU11	LN880482.1.1410	Bacteria;Patescibacteria;Parcubacteria;uncultured_bacterium	86.11	0.00664982	0.00739286	0.01386732	0.00919879	0.00493666
OTU110	FJ916095.1.1513	Bacteria;Actinobacteriota;Acidimicrobiia;Microtrichales;Ilumatobacteraceae;CL500-29_marine_group;uncultured_actinobacterium	99.5	0.00775812	0.00105612	0.00277346	0.00408835	0.002962
OTU1101	AF316776.1.1509	Bacteria;Planctomycetota;Planctomycetes;Planctomycetales;uncultured;uncultured_Crater_Lake_bacterium_CL500-4	99.53	0	0	0	0	0.00098733
OTU1103	HM244223.1.906_	Archaea;Aenigmarchaeota;Aenigmarchaeia;Aenigmarchaeales;uncultured_archaeon	88.5	0	0.00105612	0	0	0.00098733
OTU1104	HM444986.1.1341	Bacteria;Verrucomicrobiota;Chlamydiae;Chlamydiales;cvE6;uncultured_bacterium	85.71	0	0.00105612	0	0	0
OTU1106	JQ278839.1.1499	Bacteria;Nitrospirota;Nitrospiria;Nitrospirales;Nitrospiraceae;Nitrospira;uncultured_Nitrospira_sp.	99.52	0.0011083	0	0	0	0.00098733

OTU1108	HM124372.1.1399	Bacteria;Bacteroidota;Bacteroidia;Chitinophagales;Chitinophagaceae;Aurantisolimonas;Terrimonas_sp._16-45A	98.34	0	0.00105612	0	0	0
OTU111	AB025279.1.1421	Bacteria;Proteobacteria;Alphaproteobacteria;Sphingomonadales;Sphingomonadaceae;Sphingobium;Sphingomonas_sp._MBIC3020	100	0.00554152	0.00422449	0.00277346	0.00204417	0.002962
OTU1111	CT927611.1.1311	Bacteria;Proteobacteria;Alphaproteobacteria;Rhizobiales;Beijerinckiaceae;Bosea;uncultured_bacterium	100	0.0011083	0	0.00138673	0.00102209	0
OTU1112	KX172614.1.1403	Bacteria;Patescibacteria;Parcubacteria;Candidatus_Yanofskybacteria;uncultured_bacterium	93.31	0	0	0	0	0.00098733
OTU1114	HQ827945.1.1442	Bacteria;Proteobacteria;Alphaproteobacteria;Rhodospirillales;Rhodospirillaceae;uncultured;uncultured_bacterium	99	0	0.00105612	0	0	0
OTU1115	FN421557.1.1368	Bacteria;Proteobacteria;Alphaproteobacteria;Rhizobiales;Xanthobacteraceae;Rhodopseudomonas;uncultured_bacterium	100	0	0	0.00138673	0	0
OTU1116	AM997454.1.1434	Bacteria;Patescibacteria;Parcubacteria;Candidatus_Yanofskybacteria;uncultured_deep-sea_bacterium	91.96	0	0.00105612	0	0	0
OTU1118	AB294345.1.1480	Bacteria;Verrucomicrobiota;Omnitrophia;Omnitrophales;Omnitrophaceae;Candidatus_Omnitrophus;uncultured_bacterium	92.77	0.0011083	0	0	0	0
OTU1120	JX227646.1.1538	Bacteria;Verrucomicrobiota;Omnitrophia;Omnitrophales;Omnitrophaceae;Candidatus_Omnitrophus;uncultured_bacterium	93.26	0	0	0	0.00102209	0
OTU1122	MEZX01000002.366279.368183	Bacteria;Patescibacteria;WWE3;Candidatus_Berkelbacteria_bacterium_RIFCSPLOWO2_01_FTLL_50_28	94.22	0	0	0	0	0.00098733
OTU1123	AF454307.1.1449	Bacteria;Actinobacteriota;Acidimicrobiia;Microtrichales;uncultured;uncultured_actinobacterium	98.01	0	0.00105612	0	0	0
OTU1124	ET731242.1.1224_	Archaea;Nanoarchaeota;Nanoarchaeia;Woesearchaeales;uncultured_euryarchaeote	80.99	0	0.00105612	0	0	0
OTU1125	HM856568.1.1440	Bacteria;Actinobacteriota;Actinobacteria;Micrococcales;Microbacteriaceae;Rhodoluna;uncultured_Microbacteriaceae_bacterium	99.51	0	0	0.00693366	0	0
OTU1126	AF202056.1.1508	Bacteria;Firmicutes;Bacilli;Bacillales;Planococcaceae;Sporosarcina;Sporosarcina_aquimarina	99.53	0.00332491	0	0.00554693	0.04292767	0.27842777
OTU1128	JN245774.1.1480	Bacteria;Bdellovibrionota;Bdellovibrionia;Bdellovibrionales;Bdellovibrionaceae;Bdellovibrio;uncultured_bacterium	99.75	0	0.00105612	0	0	0.00098733
OTU1129	KP686751.1.1491	Bacteria;Verrucomicrobiota;Verrucomicrobiae;uncultured;uncultured_bacterium	99.76	0.0011083	0	0.00138673	0	0
OTU113	AY509485.1.1422	Bacteria;Proteobacteria;Gammaproteobacteria;Burkholderiales;Nitrosomonadaceae;Ellin6067;uncultured_beta_proteobacterium	100	0.00664982	0.01056122	0.0041602	0.00204417	0.00394933
OTU1132	MHGG01000050.25339.26910	Bacteria;Verrucomicrobiota;Omnitrophia;Omnitrophales;Omnitrophaceae;Candidatus_Omnitrophus;Omnitrophica_WOR_2_bacterium_GWF2_63_9	92.31	0	0	0	0.00102209	0
OTU1133	JN615822.1.1372	Bacteria;Nitrospirota;Nitrospiria;Nitrospirales;Nitrospiraceae;Nitrospira;uncultured_bacterium	100	0	0.00844898	0	0	0
OTU1136	HM128717.1.1432	Bacteria;Actinobacteriota;Actinobacteria;Micrococcales;Microbacteriaceae;Candidatus_Planktoluna;uncultured_bacterium	99.51	0	0	0.0041602	0.00102209	0
OTU1138	MF942642.1.1390	Bacteria;Verrucomicrobiota;Omnitrophia;Omnitrophales;Omnitrophaceae;Candidatus_Omnitrophus;uncultured_bacterium	91.61	0	0.00105612	0	0.00102209	0
OTU1139	AB680017.1.1388	Bacteria;Proteobacteria;Alphaproteobacteria;Caulobacterales;Caulobacteraceae;Brevundimonas;Brevundimonas_diminuta	100	0	0	0.00138673	0	0
OTU114	MGFA01000012.36590.38091	Bacteria;Patescibacteria;ABY1;Candidatus_Thrbacteria;Candidatus_Thrbacteria_bacterium_RIFOXYB12_FTLL_58_10	91.58	0	0.00105612	0.00277346	0.00306626	0.00098733
OTU1140	KF836147.1.1531	Bacteria;Nitrospirota;Leptospirillia;Leptospirillales;Leptospirillaceae;Leptospirillum;uncultured_bacterium	92.76	0	0	0	0.00102209	0
OTU1142	KC331522.1.1423	Bacteria;Patescibacteria;Parcubacteria;Candidatus_Adlerbacteria;uncultured_bacterium	86.04	0	0.00105612	0	0	0
OTU1143	MHGG01000050.25339.26910	Bacteria;Verrucomicrobiota;Omnitrophia;Omnitrophales;Omnitrophaceae;Candidatus_Omnitrophus;Omnitrophica_WOR_2_bacterium_GWF2_63_9	92.07	0	0	0.00138673	0.00102209	0
OTU1144	AB681044.1.1446	Bacteria;Bacteroidota;Bacteroidia;Cytophagales;Cytophagaceae;Cytophaga;Cytophaga_aurantiaca	99.52	0	0	0	0	0.00098733
OTU1145	DQ444978.1.1416	Bacteria;Firmicutes;Bacilli;Paenibacillales;Paenibacillaceae;Paenibacillus;Paenibacillus_sp._Eur1_9.9	99.53	0.00332491	0	0.1747282	0.08585533	0.01480999
OTU1146	AB680976.1.1464	Bacteria;Proteobacteria;Gammaproteobacteria;Pseudomonadales;Pseudomonadaceae;Pseudomonas;Pseudomonas_fluorescens	100	0.00332491	0.00105612	0.04437542	0	0.16488453
OTU1148	GQ472458.1.1490	Bacteria;Proteobacteria;Gammaproteobacteria;Burkholderiales;Comamonadaceae;uncultured;uncultured_bacterium	98.83	0	0.00105612	0	0.00102209	0.00197467
OTU115	JF703385.1.1436	Bacteria;Bacteroidota;Bacteroidia;Chitinophagales;Chitinophagaceae;Terrimonas;uncultured_Chitinophagaceae_bacterium	100	0.00221661	0.00211224	0.00277346	0.00306626	0.00098733
OTU1153	HQ532802.1.1513	Bacteria;Actinobacteriota;Actinobacteria;Micrococcales;Microbacteriaceae;Aurantimicrobium;uncultured_actinobacterium	99.75	0	0	0.00693366	0	0
OTU1154	JF344128.1.1458	Bacteria;Proteobacteria;Gammaproteobacteria;Chromatiales;Chromatiaceae;Candidatus_Thiobios;uncultured_gamma_proteobacterium	99.25	0	0.00211224	0	0	0
OTU1156	JQ278884.1.1533	Bacteria;Verrucomicrobiota;Omnitrophia;Omnitrophales;Omnitrophaceae;Candidatus_Omnitrophus;uncultured_bacterium	93.26	0	0.00105612	0	0	0
OTU1157	KP866212.1.1451	Bacteria;Bacteroidota;Bacteroidia;Flavobacteriales;Flavobacteriaceae;Flavobacterium;Flavobacterium_sp._HMF3121	99.29	0.0011083	0	0	0	0.00197467
OTU1158	DQ128511.1.1416	Bacteria;Elusimicrobiota;Lineage_IIa;uncultured_soil_bacterium	99.05	0	0	0	0.00102209	0.00197467
OTU1159	LCOT01000026.4898.6395	Bacteria;Patescibacteria;Parcubacteria;Candidatus_Jorgensenbacteria;Parcubacteria_group_bacterium_GW2011_GWA2_47_8b	90.37	0.0011083	0.00105612	0	0.00102209	0
OTU116	KF836147.1.1531	Bacteria;Nitrospirota;Leptospirillia;Leptospirillales;Leptospirillaceae;Leptospirillum;uncultured_bacterium	93.93	0.00221661	0	0.00277346	0.00102209	0.00098733
OTU1160	FPLS01006708.18.1524	Bacteria;Bacteroidota;Bacteroidia;Chitinophagales;Chitinophagaceae;Edaphobaculum;metagenome	94.79	0.0011083	0	0	0	0
OTU1163	JQ427491.1.1485	Bacteria;Proteobacteria;Gammaproteobacteria;Burkholderiales;Oxalobacteraceae;Herbaspirillum;uncultured_bacterium	98.59	0	0	0	0.00102209	0
OTU1164	MHJF01000011.24156.25754	Bacteria;Patescibacteria;Parcubacteria;Candidatus_Colwellbacteria;Candidatus_Harrisonbacteria_bacterium_RIFCSPHIGHO2_02_FTLL_40_20	83.87	0	0.00105612	0	0	0
OTU1165	AM936280.1.1283	Bacteria;Proteobacteria;Alphaproteobacteria;Caulobacterales;Parvularculaceae;Amphiplicatus;uncultured_Rhizobiales_bacterium	98.76	0	0	0.00138673	0	0
OTU1166	HM186378.1.1303	Bacteria;Patescibacteria;Parcubacteria;Candidatus_Yanofskybacteria;uncultured_bacterium	95.04	0	0	0	0	0.00098733
OTU1167	FPLK01002673.23.1523	Bacteria;Bacteroidota;Bacteroidia;Chitinophagales;Chitinophagaceae;Sediminibacterium;metagenome	99.53	0	0	0.00138673	0	0
OTU1168	FJ628329.1.1481	Bacteria;Bacteroidota;Bacteroidia;Flavobacteriales;Cryomorphaceae;uncultured;uncultured_bacterium	89.57	0	0	0.00138673	0	0
OTU1169	KF616714.1.1398	Bacteria;Patescibacteria;Parcubacteria;Candidatus_Staskawiczbacteria;uncultured_bacterium	95.69	0	0.00105612	0	0	0
OTU117	AJ575532.1.1471	Bacteria;Actinobacteriota;Actinobacteria;Frankiales;Sporichthyaceae;hgcI_clade;uncultured_actinobacterium	100	0.0011083	0.00422449	0.07765698	0.00204417	0.00098733
OTU1170	KF616728.1.1486	Bacteria;Myxococcota;bacteriap25;uncultured_bacterium	94.17	0.0011083	0	0	0.00204417	0
OTU1171	AF293012.1.1505	Bacteria;Nitrospirota;Nitrospiria;Nitrospirales;Nitrospiraceae;Nitrospira;uncultured_Green_Bay_ferromanganous_micronodule_bacterium_MNF8	99.76	0.00554152	0.00528061	0.01109385	0	0
OTU1174	AOIX01038262.3063.4572	Bacteria;Bacteroidota;Bacteroidia;Sphingobacteriales;Sphingobacteriaceae;Sphingobacterium;Brassica_oleracea_var._capitata_(cabbage)	100	0	0.00105612	0	0	0
OTU1177	DQ177465.1.1519	Bacteria;Firmicutes;Bacilli;Paenibacillales;Paenibacillaceae;Paenibacillus;Paenibacillus_sp._Tibet-IB15	99.3	0	0	0.51863767	0.01430922	0
OTU1178	AB681172.1.1449	Bacteria;Bacteroidota;Bacteroidia;Flavobacteriales;Flavobacteriaceae;Mesonia;Mesonia_algae	100	0.0011083	0	0	0	0
OTU1179	GQ339170.1.1501	Bacteria;Proteobacteria;Gammaproteobacteria;Burkholderiales;Gallionellaceae;Gallionella;uncultured_bacterium	98.59	0	0	0	0.00102209	0
OTU118	AB680378.1.1388	Bacteria;Proteobacteria;Alphaproteobacteria;Caulobacterales;Caulobacteraceae;Brevundimonas;Brevundimonas_diminuta	100	0.00443321	0.00422449	0.00554693	0.00408835	0.00197467
OTU1182	AY250096.1.1461	Bacteria;Proteobacteria;Gammaproteobacteria;Burkholderiales;Comamonadaceae;Rhodoferax;uncultured_bacterium	100	0	0.00105612	0.00138673	0.00102209	0.00197467
OTU1185	HM128098.1.1446	Bacteria;Bacteroidota;Bacteroidia;Sphingobacteriales;NS11-12_marine_group;uncultured_bacterium	99.53	0	0	0.00138673	0	0
OTU1188	JQ278816.1.1534	Bacteria;Verrucomicrobiota;Omnitrophia;Omnitrophales;Omnitrophaceae;Candidatus_Omnitrophus;uncultured_bacterium	90	0	0.00105612	0	0	0
OTU119	AB009937.1.1492	Bacteria;Firmicutes;Bacilli;Staphylococcales;Staphylococcaceae;Staphylococcus;Staphylococcus_capitis_subsp._urealyticus	100	0.00443321	0.00316837	0.00277346	0.00511044	0.005924
OTU1191	AB294345.1.1480	Bacteria;Verrucomicrobiota;Omnitrophia;Omnitrophales;Omnitrophaceae;Candidatus_Omnitrophus;uncultured_bacterium	93.71	0.0011083	0	0	0	0
OTU1192	AF361205.1.1491	Bacteria;Bacteroidota;Bacteroidia;Flavobacteriales;Crocinitomicaceae;Fluviicola;uncultured_Cytophagales_bacterium	99.76	0	0.00105612	0	0	0.00098733
OTU1194	DQ088797.1.1445	Bacteria;Proteobacteria;Alphaproteobacteria;Rhizobiales;Beijerinckiaceae;Methylobacterium-Methylorubrum;uncultured_bacterium	100	0	0	0.00138673	0.00204417	0
OTU1196	AB021387.1.1497	Bacteria;Proteobacteria;Gammaproteobacteria;Burkholderiales;Comamonadaceae;Malikia;Malikia_spinosa	100	0	0.00105612	0	0	0
OTU1197	AF035054.1.1416	Bacteria;Proteobacteria;Gammaproteobacteria;Burkholderiales;Comamonadaceae;Aquabacterium;Aquabacterium_commune	100	0.0011083	0.00211224	0.00138673	0	0
OTU1198	AJ237965.1.1459	Bacteria;Proteobacteria;Gammaproteobacteria;Pseudomonadales;Pseudomonadaceae;Pseudomonas;Pseudomonas_sp.	100	0	0	0.2010761	0.00204417	0.00098733
OTU1199	JN977180.1.1549	Bacteria;NB1-j;uncultured_bacterium	81.21	0	0	0.00138673	0	0
OTU12	AB680705.1.1460	Bacteria;Proteobacteria;Gammaproteobacteria;Burkholderiales;Comamonadaceae;Curvibacter;Curvibacter_delicatus	100	0.04987365	0.03590816	0.0263479	0.03066262	0.02172132
OTU120	HQ234270.1.1472	Bacteria;Firmicutes;Bacilli;Bacillales;Planococcaceae;Sporosarcina;Sporosarcina_aquimarina	100	0.88885933	0.09082652	0.19691591	0.93623197	0.80961267

OTU1200	JN941764.1.1424	Bacteria;Chloroflexi;Anaerolineae;Caldilineales;Caldilineaceae;uncultured;uncultured_bacterium	99.26	0	0.00105612	0	0.00102209	0
OTU1203	JF809778.1.1414	Bacteria;Myxococcota;bacteriap25;uncultured_bacterium	95.33	0	0	0.00138673	0	0.00098733
OTU121	FJ612200.1.1491	Bacteria;Bacteroidota;Bacteroidia;Sphingobacteriales;NS11-12_marine_group;uncultured_bacterium	100	0.01551625	0.01056122	0.04160195	0.00102209	0.00098733
OTU1211	KC990423.1.1445	Bacteria;Patescibacteria;Parcubacteria;Candidatus_Vogelbacteria;uncultured_Parcubacteria_group_bacterium	83.37	0.0011083	0	0.00277346	0	0
OTU1213	FJ820458.1.1498	Bacteria;Proteobacteria;Gammaproteobacteria;Burkholderiales;Comamonadaceae;Hydrogenophaga;uncultured_bacterium	100	0	0.00105612	0.00277346	0.00102209	0
OTU122	CXWL01054502.320.1767	Bacteria;Elusimicrobiota;Elusimicrobia;Lineage_IV;groundwater_metagenome	100	0.0011083	0.00422449	0.00277346	0.00102209	0.002962
OTU1220	KR868710.1.1452	Bacteria;Bacteroidota;Bacteroidia;Flavobacteriales;Flavobacteriaceae;Gramella;Gramella_aquimixticola	95.73	0	0	0	0.00102209	0
OTU1221	JF211081.1.1356	Bacteria;Bacteroidota;Bacteroidia;Flavobacteriales;Crocinitomicaceae;Fluviicola;uncultured_bacterium	98.34	0	0.00105612	0	0	0
OTU1222	FJ382160.1.1341	Bacteria;Actinobacteriota;Actinobacteria;Corynebacteriales;Mycobacteriaceae;Mycobacterium;uncultured_bacterium	99.51	0.0011083	0.00105612	0	0	0.00197467
OTU1223	LN870812.1.1394	Bacteria;Proteobacteria;Gammaproteobacteria;Burkholderiales;Nitrosomonadaceae;MND1;uncultured_bacterium	99.77	0	0.00211224	0.00138673	0	0
OTU1224	LCOT01000026.4898.6395	Bacteria;Patescibacteria;Parcubacteria;Candidatus_Jorgensenbacteria;Parcubacteria_group_bacterium_GW2011_GWA2_47_8b	82.54	0	0	0.00138673	0	0
OTU1228	FJ423763.1.1514	Bacteria;Actinobacteriota;Actinobacteria;Micrococcales;Micrococcaceae;Micrococcus;Micrococcus_terreus	100	0.0011083	0	0	0.00306626	0.00098733
OTU123	AJ244648.1.1416	Bacteria;Proteobacteria;Alphaproteobacteria;Caulobacterales;Caulobacteraceae;Brevundimonas;Brevundimonas_sp._LMG_11070	100	0.00221661	0.00422449	0.0041602	0.00102209	0.002962
OTU1233	AB858570.1.1334	Bacteria;Patescibacteria;Parcubacteria;TBA9983;uncultured_bacterium	89.45	0	0	0	0.00102209	0
OTU1235	GT217697.1.1430	Bacteria;Actinobacteriota;Actinobacteria;Micrococcales;Micrococcaceae;Kocuria;Kocuria_sp._ljh-23	100	0.01551625	0.00105612	0	0.00204417	0
OTU1236	AF094743.1.1494	Bacteria;Proteobacteria;Gammaproteobacteria;Pseudomonadales;Pseudomonadaceae;Pseudomonas;Pseudomonas_putida	99.53	0.0011083	0	0.20384957	0.00204417	0
OTU1237	HM187253.1.1481	Bacteria;Nitrospirota;Nitrospiria;Nitrospirales;Nitrospiraceae;Nitrospira;uncultured_bacterium	98.13	0	0	0	0	0.00098733
OTU124	CT921528.1.1343	Bacteria;Nitrospirota;Nitrospiria;Nitrospirales;Nitrospiraceae;Nitrospira;uncultured_bacterium	99.28	0.0011083	0.00211224	0.0041602	0.00102209	0.00394933
OTU1243	ET134560.1.1376	Bacteria;Myxococcota;Polyangia;Haliangiales;Haliangiaceae;Haliangium;uncultured_bacterium	96.03	0.0011083	0	0	0.00102209	0
OTU1244	FPLS01011485.12.1510	Bacteria;Bacteroidota;Bacteroidia;Bacteroidetes_VC2.1_Bac22;metagenome	97.39	0	0.00105612	0	0.00102209	0
OTU1246	ACDZ02000008.284832.286369	Bacteria;Firmicutes;Bacilli;Staphylococcales;Gemellaceae;Gemella;Gemella_haemolysans_ATCC_10379	100	0	0	0.00138673	0	0
OTU1247	FPLK01001140.10.1511	Bacteria;Actinobacteriota;Actinobacteria;PeM15;metagenome	99.76	0.0011083	0.00105612	0	0	0
OTU1248	ET134418.1.1258	Bacteria;Bdellovibrionota;Oligoflexia;0319-6G20;uncultured_bacterium	97.66	0	0	0	0	0.00098733
OTU1249	AY454668.1.1080	Archaea;Nanoarchaeota;Nanoarchaeia;Woesearchaeales;uncultured_crenarchaeote	83.12	0	0.00105612	0	0	0
OTU125	ET800805.1.1489	Bacteria;Bacteroidota;Bacteroidia;Flavobacteriales;Crocinitomicaceae;Fluviicola;uncultured_bacterium	100	0.00664982	0.00105612	0.03328156	0.00306626	0.00493666
OTU1252	KC358393.1.1301	Bacteria;Verrucomicrobiota;Omnitrophia;Omnitrophales;Omnitrophaceae;Candidatus_Omnitrophus;uncultured_bacterium	92.31	0	0.00105612	0	0	0
OTU1255	AB722124.1.1353	Bacteria;Patescibacteria;Parcubacteria;TBA9983;uncultured_bacterium	85.38	0.0011083	0	0	0	0
OTU126	ET801430.1.1495	Bacteria;Proteobacteria;Gammaproteobacteria;Burkholderiales;Comamonadaceae;Limnohabitans;uncultured_bacterium	100	0.00443321	0.00211224	0.0041602	0.00204417	0.00197467
OTU1260	FJ612447.1.1477	Bacteria;Proteobacteria;Gammaproteobacteria;Enterobacterales;Alteromonadaceae;Rheinheimera;uncultured_bacterium	100	0	0	0.00138673	0	0.00098733
OTU1264	AB778259.1.1480	Bacteria;Actinobacteriota;Actinobacteria;Micrococcales;Intrasporangiaceae;Janibacter;Janibacter_cremeus	100	0	0	0	0.00204417	0
OTU1265	HE603185.1.1416	Bacteria;Proteobacteria;Gammaproteobacteria;Burkholderiales;Nitrosomonadaceae;MND1;uncultured_beta_proteobacterium	98.59	0	0.00105612	0	0	0.00098733
OTU1266	HM270025.1.1338	Bacteria;Actinobacteriota;Actinobacteria;Frankiales;Acidothermaceae;Acidothermus;uncultured_bacterium	98.77	0.01329964	0	0	0.16660023	0
OTU1267	ET512011.1.1361	Bacteria;Proteobacteria;Gammaproteobacteria;Pseudomonadales;Pseudomonadaceae;Pseudomonas;uncultured_bacterium	95.55	0.24271845	0.00105612	0.00832039	0.00715461	0.00098733
OTU1269	AY221078.1.1469	Bacteria;Proteobacteria;Alphaproteobacteria;Ferrovibrionales;uncultured;uncultured_bacterium	99.5	0	0.00316837	0	0	0
OTU127	AF235117.1.1436	Bacteria;Bacteroidota;Bacteroidia;Flavobacteriales;Flavobacteriaceae;Gramella;Gramella_forsetii_KT0803	99.76	0.0011083	0.00211224	0.00277346	0.00204417	0.002962
OTU1272	JQ278884.1.1533	Bacteria;Verrucomicrobiota;Omnitrophia;Omnitrophales;Omnitrophaceae;Candidatus_Omnitrophus;uncultured_bacterium	98.12	0	0	0	0	0.00098733
OTU1276	FJ484483.1.1366	Bacteria;Proteobacteria;Gammaproteobacteria;Beggiatoales;Beggiatoaceae;uncultured;uncultured_proteobacterium	97.66	0	0.00211224	0.00138673	0	0
OTU1277	EF486353.1.1485	Bacteria;Bacteroidota;Bacteroidia;Flavobacteriales;Flavobacteriaceae;Salegentibacter;Salegentibacter_salarius	97.39	0	0	0.00138673	0	0
OTU1278	HQ857761.1.1442	Bacteria;Proteobacteria;Gammaproteobacteria;Pseudomonadales;Moraxellaceae;Acinetobacter;Acinetobacter_ursingii	99.77	0	0.00105612	0	0	0
OTU1279	AY734239.1.1379	Bacteria;Nitrospirota;Leptospirillia;Leptospirillales;Leptospirillaceae;Leptospirillum;uncultured_bacterium	94.39	0	0	0.00138673	0	0
OTU128	AB008393.1.1252	Bacteria;Proteobacteria;Alphaproteobacteria;Caulobacterales;Caulobacteraceae;Brevundimonas;Brevundimonas_subvibrioides	100	0.00886643	0.00528061	0.0041602	0.00204417	0.00394933
OTU1281	JX950039.1.1361	Bacteria;Proteobacteria;Alphaproteobacteria;Sphingomonadales;Sphingomonadaceae;Sphingorhabdus;Sphingopyxis_sp._TMB2-10	100	0.0011083	0.00211224	0.00832039	0	0.002962
OTU1282	HM187000.1.1345	Bacteria;Chloroflexi;Dehalococcoidia;SAR202_clade;uncultured_bacterium	98.74	0.0011083	0	0.00138673	0	0
OTU1283	AB680769.1.1412	Bacteria;Proteobacteria;Alphaproteobacteria;Sphingomonadales;Sphingomonadaceae;Sphingobium;Sphingobium_yanoikuyae	100	0	0.00105612	0	0.00102209	0
OTU1288	FJ848555.1.1359	Bacteria;Bacteroidota;Bacteroidia;Bacteroidales;Porphyromonadaceae;Porphyromonas;Porphyromonas_sp._2007b	100	0	0	0	0.00102209	0
OTU1289	AB006899.1.1452	Bacteria;Proteobacteria;Alphaproteobacteria;Rhodobacterales;Rhodobacteraceae;Paracoccus;Paracoccus_carotinifaciens	100	0.0011083	0	0	0	0
OTU129	KT696541.1.1452	Bacteria;Bacteroidota;Bacteroidia;Flavobacteriales;Flavobacteriaceae;Gramella;Gramella_sediminilitoris	100	0.0011083	0	0.0041602	0.00204417	0.00394933
OTU1292	GT272231.1.1501	Bacteria;Proteobacteria;Gammaproteobacteria;Pseudomonadales;Pseudomonadaceae;Pseudomonas;uncultured_bacterium	99.77	0.00332491	0.00105612	0	0	0.15896054
OTU1293	MHEO01000007.6346.7903	Bacteria;Nitrospirota;Leptospirillia;Leptospirillales;Leptospirillaceae;Leptospirillum;Nitrospirae_bacterium_RIFCSPHIGHO2_01_FTLL_66_17	98.6	0	0.00105612	0	0	0
OTU1297	KC189686.1.1429	Bacteria;Proteobacteria;Alphaproteobacteria;Rhodobacterales;Rhodobacteraceae;Rhodobacter;uncultured_bacterium	100	0	0.00105612	0.00554693	0	0.00987333
OTU1298	KC844795.1.1392	Bacteria;Actinobacteriota;Actinobacteria;Micrococcales;Intrasporangiaceae;Janibacter;Janibacter_anophelis	100	0.0011083	0	0	0.00102209	0
OTU1299	FR774621.1.1328	Bacteria;Bacteroidota;Bacteroidia;Flavobacteriales;Flavobacteriaceae;Flavobacterium;uncultured_bacterium	99.53	0	0	0.00277346	0	0
OTU13	KF911258.1.1474	Bacteria;Firmicutes;Bacilli;Bacillales;Planococcaceae;Sporosarcina;uncultured_bacterium	100	0.07979785	0.01056122	0.34390947	0.29844949	15.6018285
OTU130	DQ444978.1.1416	Bacteria;Firmicutes;Bacilli;Paenibacillales;Paenibacillaceae;Paenibacillus;Paenibacillus_sp._Eur1_9.9	99.06	0.00886643	0.00422449	0.38135123	2.15660422	0.00394933
OTU1303	KC604492.1.913	Archaea;Aenigmarchaeota;Aenigmarchaeia;Aenigmarchaeales;uncultured_archaeon	89.42	0.0011083	0.00316837	0	0	0
OTU1306	KX177477.1.1427	Bacteria;Proteobacteria;Alphaproteobacteria;Rhodobacterales;Rhodobacteraceae;uncultured;uncultured_bacterium	99.25	0	0.00105612	0.00138673	0	0
OTU1307	AY869677.1.1383	Bacteria;Chloroflexi;Dehalococcoidia;MSBL5;uncultured_bacterium	87.72	0	0.00105612	0	0	0
OTU1309	FPLS01027703.13.1510	Bacteria;Spirochaetota;Leptospirae;Leptospirales;Leptospiraceae;Turneriella;metagenome	100	0	0.00105612	0	0	0
OTU131	AF320989.1.1452	Bacteria;Proteobacteria;Gammaproteobacteria;Pseudomonadales;Pseudomonadaceae;Pseudomonas;Pseudomonas_tolaasii	99.77	4.18052046	0.10138774	0.04021522	0.11038543	0.01086066
OTU1311	HE603186.1.1453	Bacteria;Verrucomicrobiota;Omnitrophia;Omnitrophales;Omnitrophaceae;Candidatus_Omnitrophus;uncultured_Firmicutes_bacterium	89.74	0	0	0	0.00102209	0
OTU1312	AB680133.1.1478	Bacteria;Firmicutes;Bacilli;Staphylococcales;Staphylococcaceae;Staphylococcus;Staphylococcus_sp._NBRC_3762	99.77	0	0.00105612	0	0.00102209	0
OTU1313	ET134905.1.1331	Bacteria;Verrucomicrobiota;Omnitrophia;Omnitrophales;Omnitrophaceae;Candidatus_Omnitrophus;uncultured_bacterium	90.42	0	0	0.00138673	0	0
OTU1315	JX828409.1.1501	Bacteria;Acidobacteriota;Holophagae;Holophagales;Holophagaceae;Geothrix;Geothrix_sp._enrichment_culture_clone_AP-FeEnrich1	99.53	0.0011083	0	0	0	0
OTU1316	KM263149.1.1353	Bacteria;Cyanobacteria;Cyanobacteriia;Leptolyngbyales;Leptolyngbyaceae;Leptolyngbya_ANT.L52.2;uncultured_bacterium	96.04	0	0	0	0.00102209	0.14217588
OTU1319	AJ233430.1.1495	Bacteria;Proteobacteria;Gammaproteobacteria;Enterobacterales;Yersiniaceae;Serratia;Serratia_grimesii	100	0	0.00105612	0.00277346	0	0
OTU1320	JQ198499.1.1329	Bacteria;Patescibacteria;Parcubacteria;Candidatus_Nomurabacteria;uncultured_bacterium	88.42	0	0	0.00138673	0	0
OTU1322	JF703365.1.1427	Bacteria;Bacteroidota;Bacteroidia;Cytophagales;Microscillaceae;Hassallia;uncultured_Sphingobacteriales_bacterium	100	0	0	0.0041602	0	0

OTU1323	JN391902.1.1488	Bacteria;Bacteroidota;Bacteroidia;Sphingobacteriales;env.OPS_17;uncultured_bacterium	96.92	0	0	0	0.00102209	0
OTU1325	CP020892.1158451.1160002	Bacteria;Proteobacteria;Gammaproteobacteria;Pseudomonadales;Pseudomonadaceae;Pseudomonas;Pseudomonas_sp._M30-35	99.77	0.05430687	0.00211224	0	0.1206063	0
OTU1329	AJ633637.1.1454	Bacteria;Proteobacteria;Gammaproteobacteria;Pseudomonadales;Moraxellaceae;Acinetobacter;Acinetobacter_sp._RD4355	100	0	0	0	0.00102209	0
OTU133	EF018579.1.1388	Bacteria;Nitrospirota;Nitrospiria;Nitrospirales;Nitrospiraceae;Nitrospira;uncultured_Nitrospira_sp.	99.28	0.00443321	0.00316837	0	0.00306626	0.002962
OTU1330	AY222321.1.1446	Bacteria;Actinobacteriota;Actinobacteria;Corynebacteriales;Nocardiaceae;Nocardia;Nocardia_alba	100	0.0011083	0	0	0.00102209	0
OTU1331	KY356865.1.937	Archaea;Nanoarchaeota;Nanoarchaeia;Woesearchaeales;GW2011_GWC1_47_15;uncultured_archaeon	89.15	0	0	0	0	0.00098733
OTU1333	EF988634.1.1403	Bacteria;Proteobacteria;Gammaproteobacteria;Salinisphaerales;Salinisphaeraceae;Salinisphaera;Salinisphaera_dokdonensis_CL-ES53	99.07	0	0.00105612	0	0	0
OTU1335	MF942642.1.1390	Bacteria;Verrucomicrobiota;Omnitrophia;Omnitrophales;Omnitrophaceae;Candidatus_Omnitrophus;uncultured_bacterium	93.01	0	0	0.00138673	0	0
OTU1336	MHGG01000050.25339.26910	Bacteria;Verrucomicrobiota;Omnitrophia;Omnitrophales;Omnitrophaceae;Candidatus_Omnitrophus;Omnitrophica_WOR_2_bacterium_GWF2_63_9	91.38	0	0.00105612	0	0.00102209	0
OTU1337	AB722258.1.1321	Bacteria;Chloroflexi;Anaerolineae;RBG-13-54-9;uncultured_bacterium	94.79	0	0	0	0.00102209	0
OTU1338	LN558648.1.1433	Bacteria;Proteobacteria;Gammaproteobacteria;Burkholderiales;Comamonadaceae;Comamonas;Comamonas_aquatica	100	0	0.00211224	0.00138673	0	0.00098733
OTU134	ET800362.1.1247	Bacteria;Actinobacteriota;Acidimicrobiia;Microtrichales;Ilumatobacteraceae;CL500-29_marine_group;uncultured_bacterium	100	0.00664982	0.00105612	0.00693366	0.00204417	0.00394933
OTU1340	KX123464.1.1579	Bacteria;Patescibacteria;Parcubacteria;Candidatus_Jorgensenbacteria;Candidatus_Adlerbacteria_bacterium_GW2011_GWC1_50_9	89.2	0.0011083	0	0	0	0
OTU1341	FPLS01029252.9.1542	Bacteria;Verrucomicrobiota;Chlamydiae;Chlamydiales;Simkaniaceae;uncultured;metagenome	87.15	0	0.00105612	0	0	0
OTU1342	AB630404.1.1410	Bacteria;Proteobacteria;Alphaproteobacteria;Rhizobiales;Hyphomicrobiaceae;Pedomicrobium;uncultured_bacterium	100	0	0.00105612	0	0.00102209	0.00098733
OTU1343	KX163289.1.1349	Bacteria;Proteobacteria;Gammaproteobacteria;Acidiferrobacterales;Acidiferrobacteraceae;Sulfurifustis;uncultured_bacterium	99.53	0	0.00211224	0	0.00102209	0
OTU1347	FPLS01060071.8.1519	Bacteria;Proteobacteria;Gammaproteobacteria;Pseudomonadales;Pseudomonadaceae;Pseudomonas;metagenome	100	0.0011083	0.00105612	0.26763923	0.00102209	0
OTU1349	KP686953.1.1446	Bacteria;Bacteroidota;Bacteroidia;Sphingobacteriales;env.OPS_17;uncultured_bacterium	99.53	0	0	0.00138673	0	0
OTU135	JQ867302.1.1435	Bacteria;Nitrospirota;Leptospirillia;Leptospirillales;Leptospirillaceae;Leptospirillum;Candidatus_Troglogloea_absoloni	98.37	0.0011083	0.00316837	0.00138673	0.00408835	0.00098733
OTU1352	ET133649.1.1363	Bacteria;Bacteroidota;Bacteroidia;Cytophagales;Microscillaceae;uncultured;uncultured_bacterium	100	0	0	0.00138673	0.00102209	0
OTU1357	AB930587.1.1463	Bacteria;Nitrospirota;Nitrospiria;Nitrospirales;Nitrospiraceae;Nitrospira;uncultured_bacterium	99.76	0	0.00105612	0	0	0
OTU1358	FJ612177.1.1438	Bacteria;Proteobacteria;Gammaproteobacteria;Burkholderiales;Comamonadaceae;Limnohabitans;uncultured_bacterium	99.77	0.0011083	0	0.00138673	0	0
OTU136	JF176888.1.1309	Bacteria;Proteobacteria;Alphaproteobacteria;Micavibrionales;uncultured;uncultured_bacterium	93.28	0.00332491	0.00105612	0.00277346	0.00204417	0.002962
OTU1361	JX080239.1.1485	Bacteria;Acidobacteriota;Subgroup_22;uncultured_Acidobacteriales_bacterium	99.3	0	0	0	0	0.00098733
OTU1363	HM445499.1.1323	Bacteria;Proteobacteria;Alphaproteobacteria;Paracaedibacterales;Paracaedibacteraceae;uncultured;uncultured_bacterium	99.75	0	0	0	0.00204417	0
OTU137	KT179367.1.1485	Bacteria;Proteobacteria;Gammaproteobacteria;Enterobacterales;Enterobacteriaceae;Buttiauxella;Buttiauxella_brennerae	99.53	1.9195815	0.00739286	0.00554693	0.00408835	0.005924
OTU1371	KX239215.1.1544	Bacteria;Firmicutes;Bacilli;Bacillales;Bacillaceae;Bacillus;uncultured_bacterium	100	0.0011083	0	0	0.00511044	0.00098733
OTU1373	AY546095.1.1449	Bacteria;Actinobacteriota;Actinobacteria;Micrococcales;Micrococcaceae;Rothia;Rothia_dentocariosa	100	0	0	0	0	0.00098733
OTU1375	JF266431.1.1322	Bacteria;Chloroflexi;Anaerolineae;Anaerolineales;Anaerolineaceae;uncultured;uncultured_bacterium	99.26	0	0	0.00138673	0	0
OTU1377	FN824831.1.1299	Bacteria;Actinobacteriota;Actinobacteria;Micrococcales;Microbacteriaceae;Aurantimicrobium;uncultured_bacterium	99.75	0.00221661	0.00105612	0.00138673	0.00306626	0
OTU138	HM186579.1.1365	Bacteria;Elusimicrobiota;Elusimicrobia;Lineage_IV;uncultured_bacterium	94.69	0.0011083	0.00105612	0	0.00102209	0
OTU1385	HQ397560.1.1454	Bacteria;Chloroflexi;Dehalococcoidia;S085;uncultured_bacterium	98.51	0	0.00105612	0	0	0
OTU1388	FPLS01054955.17.1552	Bacteria;Bdellovibrionota;Oligoflexia;0319-6G20;metagenome	93.46	0.0011083	0	0	0	0
OTU139	HM129384.1.1451	Bacteria;Bacteroidota;Bacteroidia;Chitinophagales;Chitinophagaceae;Dinghuibacter;uncultured_bacterium	99.76	0.0011083	0	0.00277346	0	0.002962
OTU1390	EF660756.1.1453	Bacteria;Proteobacteria;Alphaproteobacteria;Rhizobiales;Rhizobiaceae;Aliihoeflea;Aliihoeflea_aestuarii	100	0	0	0.00277346	0	0
OTU1391	JQ278884.1.1533	Bacteria;Verrucomicrobiota;Omnitrophia;Omnitrophales;Omnitrophaceae;Candidatus_Omnitrophus;uncultured_bacterium	91.4	0	0.00211224	0	0	0.00098733
OTU1393	KJ615248.1.1503	Bacteria;Proteobacteria;Gammaproteobacteria;Burkholderiales;TRA3-20;uncultured_bacterium	100	0	0	0	0	0.00098733
OTU1395	ET286996.1.1484	Bacteria;Chloroflexi;Dehalococcoidia;SAR202_clade;uncultured_bacterium	93.7	0	0	0	0	0.00098733
OTU14	AJ227801.1.1416	Bacteria;Proteobacteria;Alphaproteobacteria;Caulobacterales;Caulobacteraceae;Brevundimonas;Brevundimonas_mediterranea	100	0.01884116	0.02745918	0.05546927	0.01737548	0.01480999
OTU140	JF697410.1.1489	Bacteria;Bacteroidota;Bacteroidia;Flavobacteriales;Crocinitomicaceae;Fluviicola;uncultured_bacterium	99.05	0.0011083	0.00422449	0.0041602	0	0.002962
OTU1400	AB016610.1.1426	Bacteria;Proteobacteria;Alphaproteobacteria;Caulobacterales;Caulobacteraceae;Asticcacaulis;Asticcacaulis_excentricus	98.51	0	0	0	0	0.00197467
OTU1401	DQ915621.1.1306	Bacteria;Proteobacteria;Alphaproteobacteria;Rhodobacterales;Rhodobacteraceae;Roseivivax;Roseivivax_halodurans	100	0	0	0	0	0.00197467
OTU1402	LCCV01000010.74269.78529	Bacteria;Patescibacteria;Parcubacteria;Candidatus_Brennerbacteria;Parcubacteria_group_bacterium_GW2011_GWA2_42_14	86.25	0	0	0.00138673	0	0
OTU1404	ET801395.1.1497	Bacteria;Proteobacteria;Gammaproteobacteria;Burkholderiales;Comamonadaceae;Limnohabitans;uncultured_bacterium	99.53	0.0011083	0	0	0	0
OTU1405	KF836216.1.1416	Bacteria;Patescibacteria;Gracilibacteria;uncultured_bacterium	87.38	0.0011083	0	0	0	0.00098733
OTU1407	HM159118.1.1405	Bacteria;Proteobacteria;Alphaproteobacteria;Sphingomonadales;Sphingomonadaceae;Sphingomonas;Sphingomonas_laterariae	99.25	0	0.00105612	0	0	0.00098733
OTU1408	GQ402722.1.1440	Bacteria;Elusimicrobiota;Elusimicrobia;Lineage_IV;uncultured_bacterium	96.38	0	0	0	0	0.00098733
OTU141	JQ867302.1.1435	Bacteria;Nitrospirota;Leptospirillia;Leptospirillales;Leptospirillaceae;Leptospirillum;Candidatus_Troglogloea_absoloni	97.9	0.00443321	0.00633673	0.00277346	0.00306626	0.00098733
OTU1410	CXWL01054502.320.1767	Bacteria;Elusimicrobiota;Elusimicrobia;Lineage_IV;groundwater_metagenome	98.79	0	0	0.00138673	0	0
OTU1411	HM187081.1.1505	Bacteria;Nitrospirota;Leptospirillia;Leptospirillales;Leptospirillaceae;Leptospirillum;uncultured_bacterium	96.71	0	0	0.00138673	0	0
OTU1413	DQ295905.1.1497	Bacteria;Proteobacteria;Gammaproteobacteria;Burkholderiales;Gallionellaceae;Gallionella;uncultured_bacterium	100	0	0	0	0.00102209	0
OTU1414	HQ184019.1.1434	Bacteria;Patescibacteria;Parcubacteria;Candidatus_Spechtbacteria;uncultured_bacterium	81.58	0.0011083	0	0	0	0
OTU1415	HM187047.1.1427	Bacteria;Verrucomicrobiota;Omnitrophia;Omnitrophales;Omnitrophaceae;Candidatus_Omnitrophus;uncultured_bacterium	92.77	0	0	0	0	0.00098733
OTU1416	KC358393.1.1301	Bacteria;Verrucomicrobiota;Omnitrophia;Omnitrophales;Omnitrophaceae;Candidatus_Omnitrophus;uncultured_bacterium	91.38	0	0.00105612	0	0	0
OTU1418	JF219539.1.1329	Bacteria;Cyanobacteria;Vampirivibrionia;Obscuribacterales;Obscuribacteraceae;uncultured_bacterium	100	0	0	0.00554693	0	0
OTU1426	FJ660503.1.1489	Bacteria;Bacteroidota;Bacteroidia;Cytophagales;Microscillaceae;Chryseolinea;uncultured_bacterium	100	0	0	0	0	0.00098733
OTU143	AB300444.1.1439	Bacteria;Proteobacteria;Alphaproteobacteria;Rhizobiales;Rhizobiaceae;Aminobacter;Mesorhizobium_sp._KA5-A	100	0.00443321	0.00528061	0.0041602	0.00511044	0.002962
OTU1430	HM318949.1.1375	Bacteria;Bdellovibrionota;Oligoflexia;0319-6G20;uncultured_bacterium	94.63	0.0011083	0	0	0	0
OTU1431	AF001477.1.1438	Bacteria;Cyanobacteria;Cyanobacteriia;Synechococcales;Cyanobiaceae;Cyanobium_PCC-6307;Cyanobium_gracile	100	0	0.00105612	0	0	0
OTU1432	JF219094.1.1310	Bacteria;Proteobacteria;Alphaproteobacteria;Sphingomonadales;Sphingomonadaceae;Novosphingobium;uncultured_bacterium	100	0.0011083	0.00105612	0	0.00102209	0
OTU1433	KC551734.1.1515	Bacteria;Bdellovibrionota;Bdellovibrionia;Bacteriovoracales;Bacteriovoracaceae;Peredibacter;uncultured_bacterium	94.17	0	0	0.00138673	0	0
OTU1437	FPLS01024945.16.1506	Bacteria;Bdellovibrionota;Bdellovibrionia;Bdellovibrionales;Bdellovibrionaceae;Bdellovibrio;metagenome	99.51	0	0	0.00138673	0	0
OTU144	AB008531.1.1246	Bacteria;Proteobacteria;Alphaproteobacteria;Caulobacterales;Caulobacteraceae;Caulobacter;Caulobacter_vibrioides	100	0.00554152	0	0.00277346	0.0081767	0.00197467
OTU1443	AY509377.1.1407	Bacteria;Bacteroidota;Bacteroidia;Sphingobacteriales;Sphingobacteriaceae;uncultured;uncultured_Sphingobacteriaceae_bacterium	100	0	0	0.0041602	0	0
OTU1446	KY190928.1.1451	Bacteria;Bacteroidota;Bacteroidia;Flavobacteriales;Flavobacteriaceae;Pseudozobellia;uncultured_bacterium	93.84	0	0	0.00138673	0	0
OTU1447	AJ252823.1.1510	Bacteria;Actinobacteriota;Actinobacteria;Pseudonocardiales;Pseudonocardiaceae;Pseudonocardia;Pseudonocardia_alni	100	0	0.00105612	0.00138673	0	0
OTU1449	ET283364.1.1448	Bacteria;Bacteroidota;Bacteroidia;Sphingobacteriales;env.OPS_17;uncultured_Bacteroidetes_bacterium	100	0	0	0.00138673	0	0

OTU145	AY212615.1.1528	Bacteria;Proteobacteria;Gammaproteobacteria;Burkholderiales;Methylophilaceae;Candidatus_Methylopumilus;uncultured_bacterium	100	0.00886643	0.00422449	0.01941424	0.00102209	0.002962
OTU1452	ET134560.1.1376	Bacteria;Myxococcota;Polyangia;Haliangiales;Haliangiaceae;Haliangium;uncultured_bacterium	94.63	0	0	0.00138673	0	0.00098733
OTU1456	JX537764.1.1475	Bacteria;SAR324_clade(Marine_group_B);uncultured_delta_proteobacterium	94.29	0	0	0	0.00102209	0
OTU1457	GT293185.1.1498	Bacteria;Proteobacteria;Gammaproteobacteria;Enterobacterales;Alteromonadaceae;Rheinheimera;uncultured_bacterium	97.66	0.00221661	0.00105612	0.00138673	0.00102209	0.19450451
OTU146	ATSF01000048.11636.13144	Bacteria;Bacteroidota;Bacteroidia;Chitinophagales;Chitinophagaceae;Sediminibacterium;Bacteroidetes_bacterium_SCGC_AAA027-G08	100	0.00221661	0.00528061	0.03466829	0	0.002962
OTU1460	AB900956.1.1414	Bacteria;Proteobacteria;Alphaproteobacteria;Sphingomonadales;Sphingomonadaceae;Sphingomonas;uncultured_Sphingomonas_sp.	100	0.00332491	0	0	0	0
OTU1464	FPLK01003001.11.1522	Bacteria;Proteobacteria;Gammaproteobacteria;Gammaproteobacteria_Incertae_Sedis;Tnknown_Family;Acidibacter;metagenome	99.53	0.0011083	0	0	0	0
OTU1468	AB694367.1.1489	Bacteria;Zixibacteria;uncultured_bacterium	90.16	0.0011083	0	0	0.00102209	0
OTU147	KP686647.1.1446	Bacteria;Bacteroidota;Bacteroidia;Flavobacteriales;Crocinitomicaceae;Fluviicola;uncultured_bacterium	99.53	0.00221661	0.00105612	0.01386732	0.00204417	0.00098733
OTU1471	FJ625343.1.1541	Bacteria;Elusimicrobiota;Lineage_IIa;uncultured_bacterium	90.28	0	0	0	0.00102209	0.00098733
OTU1472	AB597534.1.1537	Bacteria;Proteobacteria;Gammaproteobacteria;Enterobacterales;Alteromonadaceae;Rheinheimera;uncultured_gamma_proteobacterium	99.06	0	0.00105612	0	0.01022087	0
OTU1477	ET104052.1.1447	Bacteria;Bacteroidota;Bacteroidia;Chitinophagales;Chitinophagaceae;uncultured;uncultured_bacterium	99.76	0	0	0.00693366	0	0
OTU148	DQ071101.1.1438	Bacteria;Campylobacterota;Campylobacteria;Campylobacterales;Sulfurimonadaceae;Sulfurimonas;uncultured_marine_bacterium	100	0.00332491	0	0.00277346	0.00102209	0
OTU1482	FJ444635.1.1455	Bacteria;Cyanobacteria;Cyanobacteriia;Oxyphotobacteria_Incertae_Sedis;Tnknown_Family;Leptolyngbya_EcFYyyy-00;uncultured_bacterium	93.33	0	0.00211224	0	0	0
OTU1483	KF836147.1.1531	Bacteria;Nitrospirota;Leptospirillia;Leptospirillales;Leptospirillaceae;Leptospirillum;uncultured_bacterium	92.99	0	0	0.00277346	0	0
OTU1489	ET175887.1.1299	Bacteria;Bacteroidota;Bacteroidia;Flavobacteriales;Flavobacteriaceae;Salegentibacter;uncultured_Salegentibacter_sp.	98.58	0.0011083	0.00105612	0	0	0.00098733
OTU149	ET488452.1.1442	Bacteria;Patescibacteria;Parcubacteria;Paceibacterales;Paceibacteraceae;Candidatus_Paceibacter;uncultured_bacterium	76.11	0.00332491	0.00211224	0	0.00204417	0.00197467
OTU1491	FPLP01008072.2.1307	Bacteria;Bacteroidota;Bacteroidia;Bacteroidales;Prolixibacteraceae;uncultured;metagenome	96.21	0.0011083	0	0	0	0
OTU1492	HM187226.1.1470	Bacteria;Actinobacteriota;Thermoleophilia;Gaiellales;Gaiellaceae;Gaiella;uncultured_bacterium	100	0.0011083	0	0	0	0
OTU1496	MHFR01000015.11934.13497	Bacteria;Verrucomicrobiota;Omnitrophia;Omnitrophales;Omnitrophaceae;Candidatus_Omnitrophus;Omnitrophica_bacterium_RIFCSPLOWO2_12_FTLL_44_17	90.91	0	0.00105612	0	0	0
OTU1498	AM991204.1.1392	Bacteria;Patescibacteria;Parcubacteria;Candidatus_Giovannonibacteria;uncultured_bacterium	89.54	0	0.00105612	0	0.00102209	0.00098733
OTU1499	JQ675527.1.1336	Bacteria;Planctomycetota;Pla4_lineage;uncultured_bacterium	89.83	0.00221661	0.00105612	0	0	0
OTU15	AACY020469417.593.2086	Bacteria;Bacteroidota;Bacteroidia;Flavobacteriales;Flavobacteriaceae;Flavobacterium;marine_metagenome	100	0.01440794	0.02112245	0.12064566	0.01533131	0.01875932
OTU150	JN475265.1.1496	Bacteria;Verrucomicrobiota;Chlamydiae;LD1-PA32;uncultured_organism	81.5	0.0011083	0.00105612	0.00277346	0	0
OTU1501	KC990424.1.1439	Bacteria;Patescibacteria;Parcubacteria;TBA9983;uncultured_Parcubacteria_group_bacterium	81.82	0	0.00105612	0	0	0
OTU1506	AB608701.1.1475	Bacteria;Desulfobacterota;Desulfuromonadia;Geobacterales;Geobacteraceae;Citrifermentans;uncultured_bacterium	83.61	0	0.00105612	0	0	0
OTU1507	LJNF01000007.41371.42892	Bacteria;Patescibacteria;Parcubacteria;Candidatus_Yanofskybacteria;Parcubacteria_bacterium_DG_74_2	80.84	0.0011083	0	0	0	0
OTU1508	ET134927.1.1286	Bacteria;Patescibacteria;Gracilibacteria;Candidatus_Peribacteria;uncultured_bacterium	88.09	0	0.00105612	0	0.00102209	0
OTU1509	DQ520182.1.1476	Bacteria;Actinobacteriota;Acidimicrobiia;Microtrichales;Ilumatobacteraceae;CL500-29_marine_group;uncultured_bacterium	100	0.0011083	0	0	0	0
OTU151	JQ396606.1.1540	Bacteria;Firmicutes;Bacilli;Paenibacillales;Paenibacillaceae;Paenibacillus;Paenibacillus_sp._MN8-13	100	1.83756705	0.00105612	0.00970712	0.24427887	0.00394933
OTU1511	HM187369.1.1327	Bacteria;Chloroflexi;Dehalococcoidia;S085;uncultured_bacterium	99.75	0	0	0	0	0.00098733
OTU1513	CP009211.1247078.1248607	Bacteria;Actinobacteriota;Actinobacteria;Corynebacteriales;Corynebacteriaceae;Corynebacterium;Corynebacterium_imitans	100	0.0011083	0.00105612	0	0	0
OTU1519	AEVM02000006.31074.32539	Bacteria;Proteobacteria;Alphaproteobacteria;Rhizobiales;Beijerinckiaceae;Methylocystis;Methylocystis_sp._ATCC_49242	100	0	0.00105612	0	0.00102209	0.00098733
OTU152	ET117725.1.1506	Bacteria;Actinobacteriota;Actinobacteria;Frankiales;Sporichthyaceae;hgcI_clade;uncultured_actinobacterium	99.51	0.00221661	0.00739286	0.04298868	0.00204417	0.00098733
OTU1520	GQ500766.1.1431	Bacteria;Proteobacteria;Alphaproteobacteria;Caulobacterales;Hyphomonadaceae;SWB02;uncultured_bacterium	99.75	0.01219134	0	0	0	0.00098733
OTU1521	AY923084.1.1237	Bacteria;Proteobacteria;Alphaproteobacteria;Acetobacterales;Acetobacteraceae;Craurococcus-Caldovatus;uncultured_bacterium	99.75	0	0.00105612	0.00138673	0	0
OTU1523	JN869133.1.1476	Bacteria;Proteobacteria;Alphaproteobacteria;Rhizobiales;A0839;uncultured_bacterium	99.26	0	0	0	0	0.00098733
OTU1526	FJ810552.1.1456	Bacteria;Cyanobacteria;Vampirivibrionia;Caenarcaniphilales;uncultured_bacterium	95.06	0	0.00211224	0	0	0
OTU1527	EF471629.1.1438	Bacteria;Bacteroidota;Bacteroidia;Flavobacteriales;Crocinitomicaceae;Fluviicola;uncultured_Bacteroidetes_bacterium	100	0	0	0.00138673	0	0
OTU1531	HM187241.1.1433	Bacteria;Chloroflexi;Dehalococcoidia;SAR202_clade;uncultured_bacterium	96.03	0	0.00105612	0	0	0
OTU1532	LCOT01000026.4898.6395	Bacteria;Patescibacteria;Parcubacteria;Candidatus_Jorgensenbacteria;Parcubacteria_group_bacterium_GW2011_GWA2_47_8b	90.12	0.0011083	0.00211224	0	0	0
OTU1533	AF534216.1.1462	Bacteria;Proteobacteria;Gammaproteobacteria;Pseudomonadales;Pseudomonadaceae;Pseudomonas;uncultured_bacterium	96.25	0.00332491	0.43195404	0.0041602	0.00102209	0.002962
OTU1534	FPLK01001074.12.1524	Bacteria;Proteobacteria;Gammaproteobacteria;Burkholderiales;Oxalobacteraceae;uncultured;metagenome	99.06	0.0011083	0	0	0	0
OTU1535	KX911209.1.1480	Bacteria;Bacteroidota;Bacteroidia;Flavobacteriales;Flavobacteriaceae;Flavobacterium;Flavobacterium_ardleyense	98.58	0	0.00105612	0.00277346	0	0
OTU1537	JX096997.1.1467	Bacteria;Actinobacteriota;Actinobacteria;Micrococcales;Micrococcaceae;Arthrobacter;Arthrobacter_sp._C0803	100	0.0011083	0	0.00138673	0.00102209	0
OTU1538	AB279889.1.1465	Bacteria;Actinobacteriota;Actinobacteria;Micrococcales;Micrococcaceae;Arthrobacter;Arthrobacter_oryzae	100	0.03657401	0	0.00693366	0.01124296	0.002962
OTU154	FJ916095.1.1513	Bacteria;Actinobacteriota;Acidimicrobiia;Microtrichales;Ilumatobacteraceae;CL500-29_marine_group;uncultured_actinobacterium	100	0.00664982	0.01056122	0.00970712	0.00511044	0.00098733
OTU1541	KY356869.1.921	Archaea;Nanoarchaeota;Nanoarchaeia;Woesearchaeales;GW2011_GWC1_47_15;uncultured_archaeon	85.17	0.0011083	0	0	0	0
OTU1542	JX406264.1.1501	Bacteria;Proteobacteria;Gammaproteobacteria;Burkholderiales;Comamonadaceae;Limnohabitans;uncultured_bacterium	100	0	0	0.0041602	0.00102209	0.00098733
OTU1543	JWKV01000003.14465.15932	Archaea;Nanoarchaeota;Nanoarchaeia;Woesearchaeales;archaeon_GW2011_AR16	86.65	0	0.00105612	0	0	0
OTU1544	FPLS01062691.2.1377	Bacteria;Bdellovibrionota;Oligoflexia;Oligoflexales;Oligoflexaceae;uncultured;metagenome	91.82	0	0	0	0.00102209	0
OTU1545	FPLS01042162.19.1480	Bacteria;Proteobacteria;Alphaproteobacteria;Rickettsiales;SM2D12;metagenome	92.54	0.0011083	0.00105612	0	0	0.00098733
OTU1546	KY356865.1.937	Archaea;Nanoarchaeota;Nanoarchaeia;Woesearchaeales;GW2011_GWC1_47_15;uncultured_archaeon	93.75	0	0.00105612	0.00138673	0	0
OTU1547	KY356875.1.908	Archaea;Nanoarchaeota;Nanoarchaeia;Woesearchaeales;uncultured_archaeon	86.75	0.0011083	0	0	0	0
OTU1549	JQ278884.1.1533	Bacteria;Verrucomicrobiota;Omnitrophia;Omnitrophales;Omnitrophaceae;Candidatus_Omnitrophus;uncultured_bacterium	91.63	0	0	0.00138673	0	0
OTU155	HM357758.1.1424	Bacteria;Proteobacteria;Gammaproteobacteria;Burkholderiales;Comamonadaceae;Curvibacter;Curvibacter_sp._ATCC_700892	100	0.00221661	0.00211224	0.0041602	0.00204417	0
OTU1550	KJ615244.1.1500	Bacteria;Proteobacteria;Gammaproteobacteria;Burkholderiales;Comamonadaceae;Polaromonas;uncultured_bacterium	99.53	0	0	0	0.00102209	0
OTU1552	MF942642.1.1390	Bacteria;Verrucomicrobiota;Omnitrophia;Omnitrophales;Omnitrophaceae;Candidatus_Omnitrophus;uncultured_bacterium	91.38	0	0	0	0	0.00098733
OTU1557	AF351236.1.1423	Bacteria;Proteobacteria;Gammaproteobacteria;Burkholderiales;Gallionellaceae;Gallionella;uncultured_beta_proteobacterium	100	0.0011083	0	0	0	0.00098733
OTU1558	AB051699.1.1364	Bacteria;Proteobacteria;Gammaproteobacteria;Pseudomonadales;Pseudomonadaceae;Pseudomonas;Pseudomonas_sp._LAB-23	100	0	0.00105612	0.03882849	0.00102209	0.07799927
OTU1559	ET134909.1.1431	Bacteria;Verrucomicrobiota;Omnitrophia;Omnitrophales;Omnitrophaceae;Candidatus_Omnitrophus;uncultured_bacterium	91.61	0	0.00105612	0	0	0
OTU156	AM237344.1.1435	Bacteria;Proteobacteria;Alphaproteobacteria;Rhizobiales;Beijerinckiaceae;Methylobacterium-Methylorubrum;Methylobacterium_sp._OS-16.b	100	0.00443321	0.00422449	0	0.00408835	0.002962
OTU1560	DQ228369.1.1519	Bacteria;Myxococcota;Polyangia;Polyangiales;Sandaracinaceae;Sandaracinus;uncultured_bacterium	99.3	0	0	0	0.00102209	0
OTU1563	FJ236054.1.1523	Bacteria;Proteobacteria;Alphaproteobacteria;Caedibacterales;Caedibacteraceae;Caedibacter;uncultured_[Caedibacter]_sp.	94.78	0.00221661	0	0.00138673	0	0
OTU157	FM242312.1.1324	Bacteria;Proteobacteria;Gammaproteobacteria;Chromatiales;Chromatiaceae;Halochromatium;uncultured_gamma_proteobacterium	90.87	0.00554152	0.00528061	0.00277346	0.00511044	0.00493666
OTU1572	KF085051.1.1284	Bacteria;Proteobacteria;Alphaproteobacteria;Rhodobacterales;Rhodobacteraceae;Rubellimicrobium;uncultured_bacterium	99.25	0.0011083	0	0	0.00204417	0.00098733
OTU1574	KC442603.1.1433	Bacteria;Actinobacteriota;Actinobacteria;Propionibacteriales;Nocardioidaceae;Nocardioides;uncultured_actinobacterium	99.28	0	0	0.00138673	0	0

OTU1578	JF168382.1.1347	Bacteria;Actinobacteriota;Acidimicrobiia;Actinomarinales;uncultured;uncultured_bacterium	100	0	0	0	0.00204417	0
OTU158	AACY020174722.993.2486	Bacteria;Actinobacteriota;Acidimicrobiia;Microtrichales;Ilumatobacteraceae;CL500-29_marine_group;marine_metagenome	100	0.00221661	0	0.00277346	0.00204417	0.00197467
OTU1580	KC358393.1.1301	Bacteria;Verrucomicrobiota;Omnitrophia;Omnitrophales;Omnitrophaceae;Candidatus_Omnitrophus;uncultured_bacterium	94.17	0	0	0	0.00102209	0
OTU1583	AB110484.1.1450	Bacteria;Proteobacteria;Alphaproteobacteria;Rhizobiales;Xanthobacteraceae;Bradyrhizobium;Bradyrhizobium_elkanii	100	0	0.00105612	0.00554693	0	0
OTU1584	KC990423.1.1445	Bacteria;Patescibacteria;Parcubacteria;Candidatus_Vogelbacteria;uncultured_Parcubacteria_group_bacterium	85.39	0	0	0.00138673	0	0
OTU1585	KT524878.1.1437	Bacteria;Patescibacteria;Parcubacteria;Candidatus_Vogelbacteria;uncultured_bacterium	76.82	0.0011083	0	0	0	0
OTU1589	DQ417474.1.1053	Archaea;Nanoarchaeota;Nanoarchaeia;Woesearchaeales;uncultured_euryarchaeote	86.01	0	0.00105612	0	0	0
OTU159	MNVX01000029.4758.6202	Archaea;Nanoarchaeota;Nanoarchaeia;Woesearchaeales;GW2011_GWC1_47_15;Candidatus_Pacearchaeota_archaeon_CG1_02_32_21	83.07	0.00221661	0.00528061	0	0	0
OTU1592	KX172614.1.1403	Bacteria;Patescibacteria;Parcubacteria;Candidatus_Yanofskybacteria;uncultured_bacterium	93.83	0	0.00105612	0	0	0
OTU1593	ET801458.1.1504	Bacteria;Proteobacteria;Gammaproteobacteria;Burkholderiales;Methylophilaceae;Methylotenera;uncultured_bacterium	100	0	0	0.00138673	0.00102209	0.00197467
OTU1594	JX222461.1.1361	Bacteria;Firmicutes;Clostridia;Clostridiales;Clostridiaceae;Clostridium_sensu_stricto_1;uncultured_bacterium	99.75	0	0	0.07211005	0.11549587	0.00098733
OTU1597	CP002876.632833.634358	Bacteria;Proteobacteria;Gammaproteobacteria;Burkholderiales;Nitrosomonadaceae;Nitrosomonas;Nitrosomonas_sp._Is79A3	96.49	0.00221661	0.00105612	0.00277346	0.00204417	0
OTU16	HQ876463.1.1508	Bacteria;Proteobacteria;Gammaproteobacteria;Pseudomonadales;Pseudomonadaceae;Pseudomonas;Pseudomonas_fluorescens	100	13.000399	0.03696428	0.04992234	0.03168471	0.06516395
OTU160	HM187432.1.1403	Bacteria;Nitrospirota;Nitrospiria;Nitrospirales;Nitrospiraceae;Nitrospira;uncultured_bacterium	84.35	0.00221661	0.00316837	0	0.00306626	0.00098733
OTU1603	JQ580129.1.1522	Bacteria;Planctomycetota;BD7-11;uncultured_planctomycete	89.04	0	0	0.00138673	0	0
OTU1604	AJ009457.1.1525	Bacteria;Proteobacteria;Gammaproteobacteria;Burkholderiales;Comamonadaceae;Acidovorax;uncultured_bacterium_SJA-23	100	0.0011083	0	0.00138673	0	0
OTU1605	MHGG01000050.25339.26910	Bacteria;Verrucomicrobiota;Omnitrophia;Omnitrophales;Omnitrophaceae;Candidatus_Omnitrophus;Omnitrophica_WOR_2_bacterium_GWF2_63_9	92.92	0	0	0	0.00102209	0
OTU1607	AB637108.1.1511	Bacteria;Firmicutes;Bacilli;Bacillales;Bacillaceae;Bacillus;uncultured_bacterium	100	0	0	0	0.00102209	0.00098733
OTU161	KF836147.1.1531	Bacteria;Nitrospirota;Leptospirillia;Leptospirillales;Leptospirillaceae;Leptospirillum;uncultured_bacterium	91.36	0.0011083	0.00211224	0	0.00306626	0
OTU1610	AJ289164.1.1476	Bacteria;Proteobacteria;Gammaproteobacteria;Pseudomonadales;Cellvibrionaceae;Cellvibrio;Cellvibrio_fibrivorans	100	0.0011083	0.00105612	0.00138673	0.00102209	0
OTU1611	HM186645.1.1324	Bacteria;Proteobacteria;Alphaproteobacteria;Zavarziniales;uncultured;uncultured_bacterium	99.25	0.0011083	0.00105612	0	0	0.00098733
OTU1612	MH177974.1.1498	Bacteria;Actinobacteriota;Actinobacteria;Micrococcales;Intrasporangiaceae;Ornithinimicrobium;Ornithinimicrobium_sp.	98.53	0	0.00105612	0	0	0.00098733
OTU1620	HM187047.1.1427	Bacteria;Verrucomicrobiota;Omnitrophia;Omnitrophales;Omnitrophaceae;Candidatus_Omnitrophus;uncultured_bacterium	93.94	0	0.00105612	0	0	0
OTU1621	AF452106.1.1414	Bacteria;Proteobacteria;Alphaproteobacteria;Rhodobacterales;Rhodobacteraceae;Haematobacter;Haematobacter_massiliensis	100	0	0.00105612	0	0	0
OTU1624	MF942653.1.1445	Bacteria;Verrucomicrobiota;Omnitrophia;Omnitrophales;Omnitrophaceae;Candidatus_Omnitrophus;uncultured_bacterium	90.65	0	0	0	0.00102209	0
OTU1627	HQ118288.1.1479	Bacteria;Actinobacteriota;Rubrobacteria;Rubrobacterales;Rubrobacteriaceae;Rubrobacter;uncultured_bacterium	95.8	0.0011083	0	0	0	0
OTU1628	KP064190.1.1447	Bacteria;Proteobacteria;Alphaproteobacteria;Rhodobacterales;Rhodobacteraceae;Palleronia-Pseudomaribius;Palleronia_soli	99	0	0.00105612	0	0	0
OTU1629	FPLK01002409.9.1522	Bacteria;Proteobacteria;Gammaproteobacteria;Methylococcales;Methylomonadaceae;uncultured;metagenome	100	0	0.00105612	0	0	0.00098733
OTU163	ET703423.1.1486	Bacteria;Bacteroidota;Bacteroidia;Bacteroidetes_VC2.1_Bac22;uncultured_Bacteroidetes_bacterium	99.76	0.00332491	0.00316837	0.00277346	0	0.00098733
OTU1630	JF772543.1.1450	Bacteria;Proteobacteria;Gammaproteobacteria;Xanthomonadales;Xanthomonadaceae;Stenotrophomonas;Stenotrophomonas_sp._bC64(2011)	100	0	0	0	0.00204417	0
OTU1636	KC437138.1.1280	Bacteria;NB1-j;uncultured_bacterium	98.36	0.0011083	0	0.00138673	0	0
OTU1638	LN875336.1.1422	Bacteria;Proteobacteria;Gammaproteobacteria;Chromatiales;Chromatiaceae;Candidatus_Thiobios;uncultured_bacterium	99.5	0	0.00105612	0	0	0
OTU164	AB240317.1.1489	Bacteria;Proteobacteria;Gammaproteobacteria;Burkholderiales;Comamonadaceae;Ideonella;uncultured_bacterium	99.53	0.00332491	0.0095051	0.00138673	0.00408835	0.00197467
OTU1642	HM856568.1.1440	Bacteria;Actinobacteriota;Actinobacteria;Micrococcales;Microbacteriaceae;Rhodoluna;uncultured_Microbacteriaceae_bacterium	99.75	0	0	0.01109385	0	0
OTU1643	FR667509.1.1337	Bacteria;Bacteroidota;Bacteroidia;Cytophagales;Microscillaceae;uncultured;uncultured_bacterium	100	0	0	0.00277346	0	0
OTU1646	GT208432.1.1434	Bacteria;Proteobacteria;Gammaproteobacteria;Burkholderiales;Comamonadaceae;Comamonas;uncultured_prokaryote	98.36	0.0011083	0	0	0	0
OTU1649	KX123607.1.1467	Bacteria;Patescibacteria;Parcubacteria;Candidatus_Yanofskybacteria;Candidatus_Yanofskybacteria_bacterium_GW2011_GWF1_44_227	78.86	0	0.00105612	0	0	0
OTU165	KF836147.1.1531	Bacteria;Nitrospirota;Leptospirillia;Leptospirillales;Leptospirillaceae;Leptospirillum;uncultured_bacterium	93.69	0.00221661	0.00105612	0.00138673	0	0
OTU1650	HM187445.1.1351	Bacteria;Patescibacteria;Parcubacteria;Paceibacterales;Paceibacteraceae;Candidatus_Paceibacter;uncultured_bacterium	79.32	0	0.00105612	0	0.00102209	0
OTU1651	KX123380.1.2523	Bacteria;Patescibacteria;Parcubacteria;Candidatus_Jorgensenbacteria;Candidatus_Jorgensenbacteria_bacterium_GW2011_GWA2_45_13	91.03	0	0.00105612	0	0	0
OTU1659	MGTY01000053.17208.18715	Bacteria;Elusimicrobiota;Elusimicrobia;Lineage_IV;Elusimicrobia_bacterium_GWA2_69_24	92.35	0	0	0.00138673	0	0
OTU166	KP686792.1.1453	Bacteria;Bacteroidota;Bacteroidia;Flavobacteriales;NS9_marine_group;uncultured_bacterium	100	0.00554152	0.00422449	0.00693366	0.00102209	0
OTU1660	FJ485349.1.1378	Bacteria;Planctomycetota;OM190;uncultured_planctomycete	82.35	0	0.00105612	0	0	0
OTU1661	JQ278769.1.1494	Bacteria;Proteobacteria;Gammaproteobacteria;Burkholderiales;Gallionellaceae;Gallionella;uncultured_Rhodocyclaceae_bacterium	99.53	0	0.00105612	0	0	0
OTU1666	JN606985.1.1382	Bacteria;MBNT15;uncultured_bacterium	98.36	0	0	0	0	0.00098733
OTU1668	AB252200.1.1433	Bacteria;Proteobacteria;Alphaproteobacteria;Rhizobiales;Beijerinckiaceae;Methylobacterium-Methylorubrum;Methylobacterium_gregans	100	0.0011083	0.00105612	0	0.00102209	0
OTU1669	ET801739.1.1501	Bacteria;Proteobacteria;Gammaproteobacteria;Burkholderiales;Comamonadaceae;Limnohabitans;uncultured_bacterium	99.77	0	0	0	0.00102209	0
OTU167	FJ593904.1.1405	Bacteria;Bacteroidota;Bacteroidia;Flavobacteriales;Flavobacteriaceae;Flavobacterium;Flavobacterium_macrobrachii	100	0.0011083	0.00105612	0.00554693	0.00408835	0.00197467
OTU1672	KX172614.1.1403	Bacteria;Patescibacteria;Parcubacteria;Candidatus_Yanofskybacteria;uncultured_bacterium	94.9	0	0.00105612	0	0	0
OTU1675	JQ675514.1.1390	Bacteria;Myxococcota;bacteriap25;uncultured_bacterium	97.91	0	0.00105612	0	0	0.00197467
OTU1679	KM205513.1.1426	Bacteria;Acidobacteriota;Acidobacteriae;GOTTB8;uncultured_soil_bacterium	96.52	0.0011083	0	0	0	0
OTU168	AB009935.1.1492	Bacteria;Firmicutes;Bacilli;Staphylococcales;Staphylococcaceae;Staphylococcus;Staphylococcus_caprae	100	0.00221661	0.00316837	0.00138673	0.00204417	0.00197467
OTU1684	AB038030.1.1510	Bacteria;Proteobacteria;Gammaproteobacteria;Enterobacterales;Vibrionaceae;Vibrio;Vibrio_splendidus	100	0.0011083	0.00211224	0.00138673	0.00204417	0
OTU1688	AJ536880.1.1308	Bacteria;Proteobacteria;Gammaproteobacteria;Burkholderiales;Nitrosomonadaceae;IS-44;uncultured_bacterium	99.53	0	0	0	0.00102209	0.00098733
OTU169	AJ863252.1.1485	Bacteria;Nitrospirota;Nitrospiria;Nitrospirales;Nitrospiraceae;Nitrospira;uncultured_bacterium	86.21	0	0.00211224	0.00138673	0.00102209	0.00197467
OTU1693	AB686546.1.1350	Bacteria;Proteobacteria;Alphaproteobacteria;Sphingomonadales;Sphingomonadaceae;Sphingopyxis;Sphingopyxis_sp._KW066	100	0	0	0	0.00102209	0.00098733
OTU1699	HM187111.1.1482	Bacteria;Myxococcota;bacteriap25;uncultured_bacterium	99.07	0	0	0	0	0.00098733
OTU17	CT921528.1.1343	Bacteria;Nitrospirota;Nitrospiria;Nitrospirales;Nitrospiraceae;Nitrospira;uncultured_bacterium	100	0.01662455	0.00844898	0.01525405	0.00511044	0.00493666
OTU170	FPLS01020463.11.1479	Bacteria;Proteobacteria;Alphaproteobacteria;Reyranellales;Reyranellaceae;Reyranella;metagenome	99.25	0.00443321	0.00528061	0.00138673	0.00511044	0.00493666
OTU1701	FPLS01056647.10.1454	Bacteria;Proteobacteria;Alphaproteobacteria;Caulobacterales;Hyphomonadaceae;SWB02;metagenome	99.5	0.0011083	0.00105612	0	0	0.00098733
OTU1702	AB688628.1.1422	Bacteria;Proteobacteria;Alphaproteobacteria;Rickettsiales;Rickettsiaceae;Candidatus_Megaira;Candidatus_Megaira_polyxenophila	100	0	0	0.00277346	0	0
OTU1704	AY454638.1.1047	Archaea;Nanoarchaeota;Nanoarchaeia;Woesearchaeales;uncultured_crenarchaeote	90.18	0	0.00105612	0	0.00102209	0.00098733
OTU1705	HM921115.1.1279	Bacteria;Proteobacteria;Alphaproteobacteria;Micavibrionales;Micavibrionaceae;uncultured;uncultured_bacterium	99.75	0	0	0	0.00204417	0
OTU171	AY752098.1.1391	Bacteria;Actinobacteriota;Actinobacteria;Frankiales;Sporichthyaceae;hgcI_clade;uncultured_bacterium	100	0.00443321	0.00211224	0.00832039	0.00204417	0.00098733
OTU1710	HE603186.1.1453	Bacteria;Verrucomicrobiota;Omnitrophia;Omnitrophales;Omnitrophaceae;Candidatus_Omnitrophus;uncultured_Firmicutes_bacterium	89.28	0	0.00105612	0	0	0
OTU1712	KX123338.1.3317	Archaea;Thermoplasmatota;Thermoplasmata;uncultured;Candidatus_Amesbacteria_bacterium_GW2011_GWC1_47_15	84.6	0	0	0.00277346	0	0
OTU1715	MHGG01000050.25339.26910	Bacteria;Verrucomicrobiota;Omnitrophia;Omnitrophales;Omnitrophaceae;Candidatus_Omnitrophus;Omnitrophica_WOR_2_bacterium_GWF2_63_9	93.24	0	0.00105612	0	0.00102209	0

OTU1716	CXWL01054502.320.1767	Bacteria;Elusimicrobiota;Elusimicrobia;Lineage_IV;groundwater_metagenome	99.03	0	0.00105612	0	0	0
OTU1718	KY194098.1.1393	Bacteria;Chloroflexi;Dehalococcoidia;SAR202_clade;uncultured_bacterium	93.3	0	0.00105612	0	0	0
OTU1720	MF942642.1.1390	Bacteria;Verrucomicrobiota;Omnitrophia;Omnitrophales;Omnitrophaceae;Candidatus_Omnitrophus;uncultured_bacterium	93.01	0	0.00105612	0	0	0
OTU1721	LJNF01000007.41371.42892	Bacteria;Patescibacteria;Parcubacteria;Candidatus_Yanofskybacteria;Parcubacteria_bacterium_DG_74_2	81.57	0	0.00211224	0	0	0
OTU1722	AM997293.1.1472	Bacteria;Chloroflexi;Dehalococcoidia;SAR202_clade;uncultured_deep-sea_bacterium	97.02	0	0	0.00138673	0.00102209	0
OTU1724	MF942642.1.1390	Bacteria;Verrucomicrobiota;Omnitrophia;Omnitrophales;Omnitrophaceae;Candidatus_Omnitrophus;uncultured_bacterium	91.38	0.0011083	0	0	0.00102209	0
OTU173	AB046362.1.1501	Bacteria;Actinobacteriota;Actinobacteria;Corynebacteriales;Nocardiaceae;Rhodococcus;Rhodococcus_erythropolis	100	0.00554152	0.00211224	0.00138673	0.00408835	0.00098733
OTU1730	HM187445.1.1351	Bacteria;Patescibacteria;Parcubacteria;Paceibacterales;Paceibacteraceae;Candidatus_Paceibacter;uncultured_bacterium	79.08	0	0	0	0.00102209	0
OTU1731	FPLP01003652.16.1533	Bacteria;Proteobacteria;Gammaproteobacteria;Burkholderiales;Rhodocyclaceae;Ferribacterium;metagenome	100	0	0.00105612	0	0	0.00098733
OTU1733	ASNX01000003.12819.14290	Bacteria;Patescibacteria;ABY1;Candidatus_Kuenenbacteria;Parcubacteria_bacterium_SCGC_AAA011-N16	97.99	0	0.00105612	0	0	0
OTU1736	FJ373023.1.1444	Bacteria;Proteobacteria;Gammaproteobacteria;Pseudomonadales;Pseudomonadaceae;Pseudomonas;Pseudomonas_sp._W1-1	99.53	0.0011083	0.00105612	0.00277346	0	0.18463118
OTU1737	HM066571.1.1533	Bacteria;Bdellovibrionota;Oligoflexia;0319-6G20;uncultured_bacterium	93.91	0.0011083	0	0	0	0
OTU174	AB360546.1.1550	Bacteria;Firmicutes;Bacilli;Paenibacillales;Paenibacillaceae;Paenibacillus;Paenibacillus_macquariensis_subsp._defensor	99.53	0.0011083	0.00105612	1.46854892	0.29231697	0.00197467
OTU1740	AF425608.1.1465	Bacteria;Firmicutes;Bacilli;Lactobacillales;Carnobacteriaceae;Carnobacterium;Carnobacterium_viridans	100	0.0011083	0	0	0	0
OTU1741	AM934690.1.1525	Bacteria;Firmicutes;Bacilli;Paenibacillales;Paenibacillaceae;Paenibacillus;Paenibacillus_sp._BF92	100	0.00221661	0.11089285	0.09845795	0.05212645	0.01283532
OTU1745	AF523022.1.1496	Bacteria;Proteobacteria;Gammaproteobacteria;Burkholderiales;Comamonadaceae;Aquabacterium;uncultured_Aquabacterium_sp.	99.3	0.0011083	0.00211224	0.00138673	0	0
OTU1746	KC886744.1.1484	Bacteria;Bacteroidota;Bacteroidia;Flavobacteriales;Crocinitomicaceae;Fluviicola;uncultured_Cryomorphaceae_bacterium	99.29	0.00221661	0	0.00138673	0	0
OTU1747	ET488084.1.1493	Bacteria;Bacteroidota;Bacteroidia;Bacteroidales;Marinilabiliaceae;uncultured;uncultured_bacterium	94.33	0	0.00105612	0	0	0.00098733
OTU1749	MF942642.1.1390	Bacteria;Verrucomicrobiota;Omnitrophia;Omnitrophales;Omnitrophaceae;Candidatus_Omnitrophus;uncultured_bacterium	91.84	0.00221661	0	0	0	0
OTU1751	JX227655.1.1532	Bacteria;Verrucomicrobiota;Omnitrophia;Omnitrophales;uncultured_bacterium	91.27	0	0.00105612	0	0	0
OTU1752	DQ404908.1.1516	Bacteria;Bacteroidota;Bacteroidia;Chitinophagales;Chitinophagaceae;Lacibacter;uncultured_bacterium	100	0.0011083	0	0	0	0
OTU1753	FJ827859.1.1493	Bacteria;Actinobacteriota;Acidimicrobiia;Microtrichales;Ilumatobacteraceae;CL500-29_marine_group;uncultured_actinobacterium	99.75	0.0011083	0	0.00138673	0	0
OTU1756	AM997330.1.1642	Bacteria;Patescibacteria;Parcubacteria;Candidatus_Portnoybacteria;uncultured_deep-sea_bacterium	92.13	0	0	0	0.00102209	0
OTU1758	FJ902046.1.1276	Bacteria;Elusimicrobiota;Elusimicrobia;FCPT453;uncultured_Termite_group_1_bacterium	89.61	0	0	0	0	0.00098733
OTU1759	HM187304.1.1451	Bacteria;Acidobacteriota;Acidobacteriae;Acidobacteriales;uncultured;uncultured_bacterium	99.75	0.0011083	0	0	0	0
OTU176	AB930606.1.1451	Bacteria;Verrucomicrobiota;Verrucomicrobiae;uncultured;uncultured_bacterium	100	0.00221661	0	0.00138673	0	0
OTU1760	FJ901595.1.1386	Bacteria;Verrucomicrobiota;Omnitrophia;Omnitrophales;Omnitrophaceae;Candidatus_Omnitrophus;uncultured_Omnitrophica_bacterium	83.95	0	0.00211224	0	0	0.00098733
OTU1762	MF942642.1.1390	Bacteria;Verrucomicrobiota;Omnitrophia;Omnitrophales;Omnitrophaceae;Candidatus_Omnitrophus;uncultured_bacterium	92.62	0	0	0	0.00102209	0
OTU177	AJ550672.1.1506	Bacteria;Proteobacteria;Gammaproteobacteria;Burkholderiales;Burkholderiaceae;Polynucleobacter;Polynucleobacter_cosmopolitanus	100	0.00221661	0	0.08459064	0.00204417	0.002962
OTU1771	KP308722.320.1868	Archaea;Iainarchaeota;Iainarchaeia;Iainarchaeales;Candidatus_Iainarchaeum;uncultured_archaeon	84.92	0	0	0	0	0.00098733
OTU1778	EF020104.1.1375	Bacteria;Planctomycetota;Pla4_lineage;uncultured_bacterium	85.32	0.0011083	0	0	0.00102209	0
OTU178	ET703498.1.1471	Bacteria;Proteobacteria;Alphaproteobacteria;Rhodobacterales;Rhodobacteraceae;uncultured;uncultured_Rhodobacter_sp.	99.75	0.00332491	0.00633673	0.01664078	0.00306626	0.00394933
OTU1780	ET762872.1.1372	Bacteria;Proteobacteria;Gammaproteobacteria;Enterobacterales;Pasteurellaceae;Haemophilus;uncultured_bacterium	100	0	0	0.00138673	0	0.00098733
OTU1783	AB245382.1.1495	Bacteria;Firmicutes;Bacilli;Paenibacillales;Paenibacillaceae;Paenibacillus;Paenibacillus_anaericanus	99.3	0	0	0.1442201	0.06234733	0
OTU1785	JQ278945.1.1494	Bacteria;Proteobacteria;Gammaproteobacteria;Burkholderiales;Gallionellaceae;Gallionella;uncultured_beta_proteobacterium	97.89	0	0	0	0	0.00197467
OTU1787	GFKG01015589.362.1828	Bacteria;Bacteroidota;Bacteroidia;Chitinophagales;Saprospiraceae;uncultured;Sargassum_vachellianum	98.58	0.0011083	0	0.00277346	0	0
OTU1788	AY316680.1.1453	Bacteria;Actinobacteriota;Actinobacteria;Micrococcales;Micrococcaceae;Arthrobacter;Arthrobacter_sp._E28	100	0	0	0	0	0.00197467
OTU1789	FPLS01062661.12.1527	Bacteria;Proteobacteria;Gammaproteobacteria;Pseudomonadales;Pseudomonadaceae;Pseudomonas;metagenome	99.53	0	0	0.16224761	0	0
OTU1790	MHGG01000050.25339.26910	Bacteria;Verrucomicrobiota;Omnitrophia;Omnitrophales;Omnitrophaceae;Candidatus_Omnitrophus;Omnitrophica_WOR_2_bacterium_GWF2_63_9	91.38	0	0.00105612	0	0	0
OTU1796	FPLS01015639.11.1489	Bacteria;Proteobacteria;Alphaproteobacteria;Rhodospirillales;Rhodospirillaceae;uncultured;metagenome	99.75	0.0011083	0	0	0	0
OTU1797	JQ197106.1.1330	Bacteria;Patescibacteria;Parcubacteria;Candidatus_Nomurabacteria;uncultured_bacterium	76.32	0	0	0	0.00102209	0
OTU1799	KP686955.1.1439	Bacteria;Bacteroidota;Bacteroidia;Flavobacteriales;Crocinitomicaceae;Fluviicola;uncultured_bacterium	100	0.0011083	0.00316837	0	0	0
OTU18	CP001620.1003200.1004700	Bacteria;Actinobacteriota;Actinobacteria;Corynebacteriales;Corynebacteriaceae;Corynebacterium;Corynebacterium_kroppenstedtii_DSM_44385	100	0.01884116	0.0190102	0.01525405	0.02350801	0.01283532
OTU180	AB643598.1.1483	Bacteria;Proteobacteria;Gammaproteobacteria;Pseudomonadales;Moraxellaceae;Enhydrobacter;Moraxella_osloensis	100	0.00332491	0.00105612	0.00138673	0.00102209	0.002962
OTU1800	AB691575.1.1488	Bacteria;Firmicutes;Clostridia;Peptostreptococcales-Tissierellales;Family_XI;Anaerococcus;Anaerococcus_vaginalis	100	0	0	0.00138673	0	0
OTU1802	HQ121009.1.1507	Bacteria;Bdellovibrionota;Oligoflexia;Oligoflexales;Oligoflexaceae;uncultured;uncultured_bacterium	94.63	0	0	0	0	0.00098733
OTU1803	HM187166.1.1462	Bacteria;Verrucomicrobiota;Omnitrophia;Omnitrophales;uncultured_bacterium	95.56	0	0	0.00138673	0	0
OTU1804	HM187241.1.1433	Bacteria;Chloroflexi;Dehalococcoidia;SAR202_clade;uncultured_bacterium	97.02	0.0011083	0	0	0	0.00098733
OTU1805	JQ278923.1.1495	Bacteria;Proteobacteria;Gammaproteobacteria;Acidiferrobacterales;Acidiferrobacteraceae;uncultured;uncultured_gamma_proteobacterium	99.53	0	0.00105612	0.00138673	0.00102209	0.00098733
OTU1808	FPLS01056716.36.1538	Bacteria;Actinobacteriota;Thermoleophilia;Solirubrobacterales;Solirubrobacteraceae;Conexibacter;metagenome	97.66	0	0.00105612	0	0	0
OTU1809	JN656867.1.1490	Bacteria;Bacteroidota;Bacteroidia;Chitinophagales;Chitinophagaceae;Sediminibacterium;uncultured_Bacteroidetes_bacterium	99.76	0	0	0	0	0.00098733
OTU181	MFTV01000001.3080.4539	Bacteria;Patescibacteria;Parcubacteria;TBA9983;Candidatus_Nomurabacteria_bacterium_RIFCSPHIGHO2_02_40_30	96.4	0	0.00316837	0.00138673	0.00102209	0.00098733
OTU1811	AB030598.1.1449	Bacteria;Campylobacterota;Campylobacteria;Campylobacterales;Arcobacteraceae;uncultured;uncultured_epsilon_proteobacterium_1065	100	0	0.00105612	0.00277346	0	0
OTU1814	KX505865.1.1432	Bacteria;Bacteroidota;Bacteroidia;Flavobacteriales;Flavobacteriaceae;Flavobacterium;Flavobacterium_sp.	96.68	0	0	0.00138673	0	0
OTU182	ET801343.1.1495	Bacteria;Proteobacteria;Gammaproteobacteria;Burkholderiales;Comamonadaceae;Limnohabitans;uncultured_bacterium	100	0	0	0.00554693	0.00102209	0.00197467
OTU1820	AB682160.1.1448	Bacteria;Bacteroidota;Bacteroidia;Cytophagales;Cyclobacteriaceae;Algoriphagus;Algoriphagus_aquatilis	99.52	0.0011083	0	0	0	0
OTU1821	DQ411542.1.1398	Bacteria;Actinobacteriota;Actinobacteria;Propionibacteriales;Nocardioidaceae;Nocardioides;Nocardioides_furvisabuli	100	0	0.00105612	0.00277346	0.00102209	0
OTU1827	AM745262.1.1504	Bacteria;Firmicutes;Bacilli;Paenibacillales;Paenibacillaceae;Paenibacillus;Paenibacillus_anaericanus	99.3	0	0	0.23990459	0.04088349	0
OTU1828	KY356869.1.921	Archaea;Nanoarchaeota;Nanoarchaeia;Woesearchaeales;GW2011_GWC1_47_15;uncultured_archaeon	85.3	0	0.00105612	0	0	0
OTU183	AF288300.1.1446	Bacteria;Proteobacteria;Alphaproteobacteria;Rhizobiales;Beijerinckiaceae;Bosea;Bosea_eneae	99.75	0.0011083	0.00316837	0.00277346	0.00306626	0
OTU1832	HM069053.1.1447	Bacteria;Proteobacteria;Alphaproteobacteria;Rhizobiales;Rhizobiales_Incertae_Sedis;uncultured;uncultured_bacterium	99.75	0	0.00105612	0	0	0.00098733
OTU1834	AB506174.1.1514	Bacteria;Firmicutes;Clostridia;Oscillospirales;Butyricicoccaceae;TCG-008;uncultured_bacterium	100	0.0011083	0	0	0.00102209	0
OTU1835	ET801712.1.1499	Bacteria;Proteobacteria;Gammaproteobacteria;Burkholderiales;Alcaligenaceae;GKS98_freshwater_group;uncultured_bacterium	100	0.0011083	0	0	0	0.00098733
OTU1836	KP308750.18552.19877	Archaea;Nanoarchaeota;Nanoarchaeia;Woesearchaeales;SCGC_AAA011-D5;uncultured_archaeon	88.05	0	0.00105612	0	0	0
OTU1837	FJ557586.1.1389	Bacteria;Actinobacteriota;Actinobacteria;Micrococcales;Micrococcaceae;Rothia;uncultured_bacterium	100	0.0011083	0.00105612	0	0.00102209	0
OTU1838	JQ675510.1.1289	Bacteria;Proteobacteria;Gammaproteobacteria;Acidiferrobacterales;Acidiferrobacteraceae;Sulfurifustis;uncultured_bacterium	97.89	0	0.00211224	0	0	0
OTU184	CP016889.1470665.1472216	Bacteria;Proteobacteria;Gammaproteobacteria;Enterobacterales;Erwiniaceae;Pantoea;Pantoea_agglomerans	100	0.01884116	1.09942336	0.00138673	0.00102209	0

OTU1840	MHGG01000050.25339.26910	Bacteria;Verrucomicrobiota;Omnitrophia;Omnitrophales;Omnitrophaceae;Candidatus_Omnitrophus;Omnitrophica_WOR_2_bacterium_GWF2_63_9	91.4	0.0011083	0.00105612	0	0	0.00098733
OTU1841	AB273732.1.1489	Bacteria;Proteobacteria;Gammaproteobacteria;Enterobacterales;Enterobacteriaceae;Escherichia-Shigella;Shigella_sonnei	100	0.0011083	0.00105612	0	0	0
OTU1846	AF235999.1.1446	Bacteria;Proteobacteria;Alphaproteobacteria;Rhizobiales;Labraceae;Labrys;alpha_proteobacterium_A0838	100	0	0	0.00138673	0	0
OTU185	AB681633.1.1458	Bacteria;Proteobacteria;Gammaproteobacteria;Burkholderiales;Burkholderiaceae;Polynucleobacter;Polynucleobacter_sp._NBRC_101963	100	0.00332491	0.00422449	0.04853561	0.00511044	0.00197467
OTU1850	AF016390.1.1538	Bacteria;Firmicutes;Bacilli;Lactobacillales;Carnobacteriaceae;Granulicatella;Granulicatella_elegans	100	0	0	0	0	0.00098733
OTU1853	FJ820409.1.1488	Bacteria;Proteobacteria;Gammaproteobacteria;Burkholderiales;Comamonadaceae;Limnohabitans;uncultured_bacterium	100	0	0	0.00554693	0	0
OTU1855	HM127655.1.1467	Bacteria;Proteobacteria;Gammaproteobacteria;Burkholderiales;MWH-TniP1_aquatic_group;uncultured_bacterium	99.3	0	0.00105612	0	0	0
OTU1856	AM991204.1.1392	Bacteria;Patescibacteria;Parcubacteria;Candidatus_Giovannonibacteria;uncultured_bacterium	80.67	0	0.00105612	0	0	0
OTU186	KT719559.1.1407	Bacteria;Firmicutes;Bacilli;Bacillales;Bacillaceae;Bacillus;Bacillus_soli	100	0.0011083	0	0.00693366	1.44625354	0
OTU1865	AF537601.1.1468	Bacteria;Actinobacteriota;Actinobacteria;Corynebacteriales;Corynebacteriaceae;Corynebacterium;Corynebacterium_mucifaciens	100	0	0.00105612	0	0	0
OTU1866	JQ278884.1.1533	Bacteria;Verrucomicrobiota;Omnitrophia;Omnitrophales;Omnitrophaceae;Candidatus_Omnitrophus;uncultured_bacterium	90.7	0.0011083	0	0.00138673	0	0
OTU1868	ET777664.1.1396	Bacteria;Proteobacteria;Gammaproteobacteria;Pseudomonadales;Pseudomonadaceae;Pseudomonas;uncultured_bacterium	95.08	0	0	0	0	0.1244039
OTU1869	JQ278884.1.1533	Bacteria;Verrucomicrobiota;Omnitrophia;Omnitrophales;Omnitrophaceae;Candidatus_Omnitrophus;uncultured_bacterium	92.71	0	0.00105612	0	0	0.00098733
OTU1870	HM186547.1.1393	Bacteria;Verrucomicrobiota;Omnitrophia;Omnitrophales;Omnitrophaceae;Candidatus_Omnitrophus;uncultured_bacterium	93.26	0	0	0	0.00102209	0
OTU1871	JQ278816.1.1534	Bacteria;Verrucomicrobiota;Omnitrophia;Omnitrophales;Omnitrophaceae;Candidatus_Omnitrophus;uncultured_bacterium	90.72	0.0011083	0	0	0	0
OTU1873	ET431733.1.1476	Bacteria;Bacteroidota;Bacteroidia;Flavobacteriales;Flavobacteriaceae;Flavobacterium;uncultured_bacterium	98.58	0	0.00105612	0.00138673	0	0
OTU1874	HM187014.1.1332	Bacteria;Chloroflexi;P2-11E;uncultured_bacterium	99.01	0	0	0	0	0.00098733
OTU1876	KF836218.1.1353	Bacteria;Patescibacteria;Parcubacteria;Candidatus_Yanofskybacteria;uncultured_bacterium	80.6	0	0.00105612	0	0	0
OTU1877	JX222018.1.1446	Bacteria;Proteobacteria;Alphaproteobacteria;Caulobacterales;Caulobacteraceae;Caulobacter;uncultured_bacterium	99.75	0	0.00105612	0	0	0
OTU1878	ET134909.1.1431	Bacteria;Verrucomicrobiota;Omnitrophia;Omnitrophales;Omnitrophaceae;Candidatus_Omnitrophus;uncultured_bacterium	94.17	0	0	0	0.00102209	0
OTU188	AY957924.1.1459	Bacteria;Proteobacteria;Alphaproteobacteria;Rhodobacterales;Rhodobacteraceae;Rhodobacter;uncultured_bacterium	100	0.00664982	0	0.00138673	0.00306626	0.00394933
OTU1884	AB680704.1.1454	Bacteria;Proteobacteria;Gammaproteobacteria;Burkholderiales;Comamonadaceae;Comamonas;Comamonas_aquatica	100	0	0.00105612	0.00277346	0	0
OTU1885	AM114534.1.1497	Bacteria;Proteobacteria;Gammaproteobacteria;Pseudomonadales;Pseudomonadaceae;Pseudomonas;Pseudomonas_peli	99.77	0.00332491	0	0.00277346	0.00102209	0.13328989
OTU1887	JF809737.1.1377	Bacteria;Chloroflexi;Dehalococcoidia;SAR202_clade;uncultured_bacterium	92.71	0.0011083	0	0	0	0
OTU1888	KJ615244.1.1500	Bacteria;Proteobacteria;Gammaproteobacteria;Burkholderiales;Comamonadaceae;Polaromonas;uncultured_bacterium	99.77	0	0.00105612	0.00138673	0	0
OTU189	KX123523.1.1506	Bacteria;Patescibacteria;Parcubacteria;Candidatus_Azambacteria;Candidatus_Azambacteria_bacterium_GW2011_GWC2_45_7b	89.95	0.0011083	0	0	0.00102209	0.00197467
OTU1890	LCOT01000026.4898.6395	Bacteria;Patescibacteria;Parcubacteria;Candidatus_Jorgensenbacteria;Parcubacteria_group_bacterium_GW2011_GWA2_47_8b	86.93	0.0011083	0	0	0	0
OTU1892	AB017489.1.1474	Bacteria;Proteobacteria;Gammaproteobacteria;Burkholderiales;Chromobacteriaceae;Chromobacterium;beta_proteobacterium_MBIC3903	94.61	0	0	0	0.00102209	0.00098733
OTU1894	LN555101.1.1365	Bacteria;Proteobacteria;Alphaproteobacteria;Dongiales;Dongiaceae;Dongia;uncultured_Dongia_sp.	99.5	0	0	0	0	0.00098733
OTU1895	KF712676.1.1487	Bacteria;Proteobacteria;Gammaproteobacteria;Burkholderiales;TRA3-20;uncultured_bacterium	99.3	0.0011083	0	0	0.00102209	0
OTU1897	HE603186.1.1453	Bacteria;Verrucomicrobiota;Omnitrophia;Omnitrophales;Omnitrophaceae;Candidatus_Omnitrophus;uncultured_Firmicutes_bacterium	89.51	0	0	0	0	0.00098733
OTU1899	KC255301.1.1472	Bacteria;Chloroflexi;Anaerolineae;Anaerolineales;Anaerolineaceae;uncultured;uncultured_bacterium	99.75	0	0.00105612	0	0.00102209	0
OTU19	ET117878.1.1506	Bacteria;Actinobacteriota;Actinobacteria;Frankiales;Sporichthyaceae;hgcI_clade;uncultured_actinobacterium	100	0.02659928	0.02429081	0.18443532	0.0163534	0.005924
OTU190	ET937837.1.1527	Bacteria;Nitrospirota;Nitrospiria;Nitrospirales;Nitrospiraceae;Nitrospira;uncultured_bacterium	99.76	0.0011083	0.00105612	0.0041602	0.00511044	0
OTU1900	FPLS01044324.16.1483	Bacteria;Proteobacteria;Alphaproteobacteria;Sphingomonadales;Sphingomonadaceae;Sphingomonas;metagenome	100	0	0	0	0.00102209	0
OTU1901	JQ769573.1.1465	Bacteria;Proteobacteria;Alphaproteobacteria;Puniceispirillales;EF100-94H03;uncultured_bacterium	96.52	0	0.00105612	0	0.00102209	0
OTU1904	AJ315066.1.1430	Bacteria;Firmicutes;Bacilli;Bacillales;Bacillaceae;Bacillus;Bacillus_sp._19498	100	0.0011083	0	0.00138673	0.03679514	0
OTU1906	HM187376.1.1395	Bacteria;Methylomirabilota;Methylomirabilia;Rokubacteriales;uncultured_bacterium	98.13	0	0	0	0.00102209	0
OTU1907	AY734239.1.1379	Bacteria;Nitrospirota;Leptospirillia;Leptospirillales;Leptospirillaceae;Leptospirillum;uncultured_bacterium	99.3	0	0.00105612	0	0	0
OTU1908	HM186378.1.1303	Bacteria;Patescibacteria;Parcubacteria;Candidatus_Yanofskybacteria;uncultured_bacterium	88.55	0.0011083	0	0.00138673	0	0
OTU1909	MF942641.1.1510	Bacteria;Verrucomicrobiota;Omnitrophia;Omnitrophales;uncultured_bacterium	94.37	0	0	0	0	0.00098733
OTU191	CXWL01035971.2106.3526	Bacteria;Patescibacteria;Parcubacteria;Candidatus_Kaiserbacteria;groundwater_metagenome	93.35	0.0011083	0	0	0.00102209	0
OTU1913	JQ278816.1.1534	Bacteria;Verrucomicrobiota;Omnitrophia;Omnitrophales;Omnitrophaceae;Candidatus_Omnitrophus;uncultured_bacterium	92.79	0	0.00105612	0	0	0
OTU1916	AM991204.1.1392	Bacteria;Patescibacteria;Parcubacteria;Candidatus_Giovannonibacteria;uncultured_bacterium	92.14	0	0	0	0.00102209	0
OTU1917	FJ744803.1.1357	Bacteria;Firmicutes;Bacilli;Erysipelotrichales;Erysipelatoclostridiaceae;TCG-004;uncultured_bacterium	96.01	0	0.00105612	0	0.00102209	0
OTU192	DQ177465.1.1519	Bacteria;Firmicutes;Bacilli;Paenibacillales;Paenibacillaceae;Paenibacillus;Paenibacillus_sp._Tibet-IB15	100	0.0011083	0.00105612	1.64605059	0.08585533	0
OTU1921	JN003063.1.1296	Bacteria;Proteobacteria;Gammaproteobacteria;Acidiferrobacterales;Acidiferrobacteraceae;Sulfurifustis;uncultured_gamma_proteobacterium	99.53	0.0011083	0.00105612	0.00138673	0.00102209	0
OTU1927	AM396260.1.1415	Bacteria;Actinobacteriota;Actinobacteria;Micrococcales;Microbacteriaceae;Agrococcus;Agrococcus_jejuensis	100	0.0011083	0	0	0	0
OTU1929	AM396909.1.1503	Bacteria;Proteobacteria;Gammaproteobacteria;Enterobacterales;Erwiniaceae;Pantoea;Enterobacter_sp._NJ-1	100	0.11083034	0	0	0	0
OTU193	ET117582.1.1509	Bacteria;Actinobacteriota;Actinobacteria;Frankiales;Sporichthyaceae;Candidatus_Planktophila;uncultured_actinobacterium	100	0.00332491	0.00528061	0.0041602	0.00613252	0.00098733
OTU194	FJ612177.1.1438	Bacteria;Proteobacteria;Gammaproteobacteria;Burkholderiales;Comamonadaceae;Limnohabitans;uncultured_bacterium	100	0	0.00211224	0.01802751	0.00306626	0
OTU1940	KY356869.1.921	Archaea;Nanoarchaeota;Nanoarchaeia;Woesearchaeales;GW2011_GWC1_47_15;uncultured_archaeon	82.24	0	0	0	0	0.00098733
OTU1941	AFNK01000005.58.1579	Bacteria;Proteobacteria;Gammaproteobacteria;Enterobacterales;Pasteurellaceae;Haemophilus;Haemophilus_sputorum_CCTG_13788	100	0.0011083	0	0	0	0.00098733
OTU1942	AY326584.1.1479	Bacteria;Myxococcota;bacteriap25;uncultured_soil_bacterium	92.79	0	0.00105612	0	0	0
OTU1944	KC554529.1.1519	Bacteria;Actinobacteriota;Actinobacteria;Propionibacteriales;Nocardioidaceae;Nocardioides;uncultured_bacterium	99.75	0.0011083	0.00105612	0	0	0
OTU1945	HQ857685.1.1499	Bacteria;Bacteroidota;Rhodothermia;Balneolales;Balneolaceae;uncultured;uncultured_Bacteroidetes_bacterium	89.81	0	0	0	0	0.002962
OTU1946	KY190469.1.1442	Bacteria;Actinobacteriota;MB-A2-108;uncultured_bacterium	99.75	0	0	0	0.00102209	0
OTU1948	JF265705.1.1359	Bacteria;Bacteroidota;Bacteroidia;Sphingobacteriales;AKYH767;uncultured_bacterium	97.16	0	0	0	0	0.00098733
OTU195	CT466895.1.1355	Bacteria;Proteobacteria;Gammaproteobacteria;Burkholderiales;Rhodocyclaceae;Dechloromonas;uncultured_beta_proteobacterium	100	0	0	0.00277346	0	0.00197467
OTU1953	AF507695.1.1458	Bacteria;Chloroflexi;Anaerolineae;RBG-13-54-9;uncultured_soil_bacterium	97.02	0	0	0.00138673	0	0
OTU1954	GFKG01015589.362.1828	Bacteria;Bacteroidota;Bacteroidia;Chitinophagales;Saprospiraceae;uncultured;Sargassum_vachellianum	98.82	0	0	0.00138673	0	0
OTU1955	JQ712468.1.1356	Bacteria;Patescibacteria;Gracilibacteria;JGI_0000069-P22;uncultured_bacterium	96.52	0	0	0	0	0.00098733
OTU1958	AB637152.1.1508	Bacteria;Firmicutes;Bacilli;Bacillales;Bacillaceae;Bacillus;uncultured_bacterium	100	0	0.00105612	0	0.00204417	0.00098733
OTU196	CXOM01000002.332084.333594	Bacteria;Proteobacteria;Gammaproteobacteria;Burkholderiales;Comamonadaceae;Limnohabitans;Limnohabitans_sp._Rim11	100	0.00664982	0.00739286	0.01802751	0.00102209	0.00098733
OTU1961	GT183621.1.1423	Bacteria;Verrucomicrobiota;Verrucomicrobiae;Pedosphaerales;Pedosphaeraceae;DEV114;uncultured_bacterium	96.24	0	0.00105612	0	0	0
OTU1967	AM882530.1.1493	Bacteria;Proteobacteria;Gammaproteobacteria;Pseudomonadales;Halieaceae;OM60(NOR5)_clade;uncultured_gamma_proteobacterium	100	0.0011083	0	0	0	0
OTU1968	MF942642.1.1390	Bacteria;Verrucomicrobiota;Omnitrophia;Omnitrophales;Omnitrophaceae;Candidatus_Omnitrophus;uncultured_bacterium	89.98	0.0011083	0	0	0	0

OTU1969	JQ278773.1.1521	Bacteria;Methylomirabilota;Methylomirabilia;Methylomirabilales;Methylomirabilaceae;wb1-A12;uncultured_Thermoanaerobacteraceae_bacterium	99.77	0.0011083	0	0	0	0
OTU1975	HQ343231.1.1502	Bacteria;Proteobacteria;Gammaproteobacteria;Burkholderiales;Rhodocyclaceae;Dechloromonas;uncultured_bacterium	99.77	0	0	0	0.00102209	0
OTU1978	AB252879.1.1478	Bacteria;Planctomycetota;Pla4_lineage;uncultured_Planctomycetaceae_bacterium	99.75	0	0.00105612	0.00138673	0	0
OTU1979	ET815294.1.1499	Bacteria;Firmicutes;Bacilli;Paenibacillales;Paenibacillaceae;Paenibacillus;Paenibacillus_glacialis	98.36	0.0011083	0.00105612	0.50615709	0.22383712	0.002962
OTU198	AJ227766.1.1439	Bacteria;Proteobacteria;Alphaproteobacteria;Caulobacterales;Caulobacteraceae;Caulobacter;Caulobacter_sp.	100	0	0	0	0	0.00098733
OTU1985	JQ278784.1.1466	Bacteria;Elusimicrobiota;Elusimicrobia;Lineage_IV;uncultured_bacterium	90.32	0	0	0.00138673	0	0
OTU1986	AM997374.1.1461	Bacteria;Patescibacteria;Parcubacteria;uncultured_deep-sea_bacterium	87.16	0.0011083	0.00105612	0	0	0
OTU1987	AF542230.1.1406	Bacteria;Firmicutes;Clostridia;Peptostreptococcales-Tissierellales;Family_XI;Peptoniphilus;Peptoniphilus_lacrimalis	100	0.0011083	0	0	0	0
OTU1988	LN870758.1.1414	Bacteria;Verrucomicrobiota;Omnitrophia;Omnitrophales;Omnitrophaceae;Candidatus_Omnitrophus;uncultured_bacterium	88.03	0	0	0	0.00102209	0
OTU1989	KY356865.1.937	Archaea;Nanoarchaeota;Nanoarchaeia;Woesearchaeales;GW2011_GWC1_47_15;uncultured_archaeon	87.2	0	0.00105612	0	0	0
OTU199	FJ612299.1.1484	Bacteria;Bacteroidota;Bacteroidia;Sphingobacteriales;NS11-12_marine_group;uncultured_bacterium	99.53	0.0011083	0.00105612	0	0	0
OTU1990	FPLK01002510.10.1490	Bacteria;Actinobacteriota;Actinobacteria;Micrococcales;Microbacteriaceae;Rhodoluna;metagenome	100	0	0.00105612	0.00138673	0	0
OTU1996	FJ810561.1.1518	Bacteria;Verrucomicrobiota;Omnitrophia;Omnitrophales;Omnitrophaceae;Candidatus_Omnitrophus;uncultured_bacterium	94.37	0.0011083	0	0	0	0
OTU1997	FN553930.1.1480	Bacteria;Bacteroidota;Bacteroidia;Flavobacteriales;Crocinitomicaceae;uncultured;uncultured_sediment_bacterium	92.89	0	0.00105612	0	0.00102209	0
OTU1998	ET512011.1.1361	Bacteria;Proteobacteria;Gammaproteobacteria;Pseudomonadales;Pseudomonadaceae;Pseudomonas;uncultured_bacterium	95.55	0.00221661	0.13518366	0	0	0
OTU1999	JQ278884.1.1533	Bacteria;Verrucomicrobiota;Omnitrophia;Omnitrophales;Omnitrophaceae;Candidatus_Omnitrophus;uncultured_bacterium	92.33	0.0011083	0	0	0	0
OTU2	AB305067.1.1351	Bacteria;Cyanobacteria;Cyanobacteriia;Cyanobacteriales;Microcystaceae;Microcystis_PCC-7914;Microcystis_aeruginosa	100	0.08866427	0.11406121	0.12896605	0.10016456	0.06022728
OTU20	AJ609630.1.1468	Bacteria;Actinobacteriota;Actinobacteria;Micrococcales;Micrococcaceae;Glutamicibacter;Glutamicibacter_bergerei	100	0.02216607	0.08237754	0.01664078	10.9056716	0.01184799
OTU200	AB078079.1.1474	Bacteria;Bacteroidota;Bacteroidia;Cytophagales;Cyclobacteriaceae;Marinoscillum;Marinoscillum_furvescens	95.72	0.00221661	0	0	0.00102209	0.00098733
OTU2000	HE602816.1.1418	Bacteria;Nitrospirota;Nitrospiria;Nitrospirales;Nitrospiraceae;Nitrospira;uncultured_bacterium	99.76	0.0011083	0.00105612	0.01248059	0.00204417	0
OTU2004	AB930763.1.1448	Bacteria;Bacteroidota;Bacteroidia;Flavobacteriales;Crocinitomicaceae;Fluviicola;uncultured_bacterium	96.92	0	0	0.00554693	0	0
OTU2006	AB672229.1.1478	Bacteria;Myxococcota;bacteriap25;uncultured_bacterium	96.03	0	0	0	0	0.00098733
OTU2009	MGDZ01000002.11263.12728	Bacteria;Patescibacteria;ABY1;Candidatus_Magasanikbacteria;Candidatus_Thrbacteria_bacterium_RIFCSPHIGHO2_02_FTLL_57_19	84.31	0	0.00105612	0	0	0
OTU201	AB062105.1.1479	Bacteria;Proteobacteria;Alphaproteobacteria;Sphingomonadales;Sphingomonadaceae;Porphyrobacter;Porphyrobacter_sanguineus	100	0.00221661	0.00422449	0.00277346	0.00204417	0.00394933
OTU2012	JN178928.1.1398	Bacteria;Actinobacteriota;Acidimicrobiia;Microtrichales;Iamiaceae;Iamia;uncultured_actinobacterium	99.01	0.00332491	0	0	0	0.00098733
OTU2019	JN128637.1.1414	Bacteria;Proteobacteria;Gammaproteobacteria;Burkholderiales;Comamonadaceae;Ideonella;Aquincola_sp._THE-49	99.53	0	0.01161735	0.00138673	0	0.00098733
OTU202	KX363701.1.1459	Bacteria;Proteobacteria;Gammaproteobacteria;Pseudomonadales;Pseudomonadaceae;Pseudomonas;uncultured_Pseudomonas_sp.	99.53	0.00886643	0.00633673	1.9261704	0.00408835	0.00493666
OTU2021	MG758043.1.1496	Bacteria;Firmicutes;Bacilli;Paenibacillales;Paenibacillaceae;Paenibacillus;Paenibacillus_sp.	98.36	0	0	0.08459064	0.26063226	0
OTU2022	KF836147.1.1531	Bacteria;Nitrospirota;Leptospirillia;Leptospirillales;Leptospirillaceae;Leptospirillum;uncultured_bacterium	91.12	0	0	0	0	0.00098733
OTU2023	JQ278884.1.1533	Bacteria;Verrucomicrobiota;Omnitrophia;Omnitrophales;Omnitrophaceae;Candidatus_Omnitrophus;uncultured_bacterium	97.41	0	0	0	0	0.00098733
OTU2027	MF942640.1.1495	Bacteria;Verrucomicrobiota;Omnitrophia;Omnitrophales;Omnitrophaceae;Candidatus_Omnitrophus;uncultured_bacterium	89.51	0	0	0	0.00102209	0
OTU2029	JN496812.1.1383	Bacteria;NKB15;uncultured_organism	83.88	0.0011083	0.00105612	0	0	0
OTU203	AB506165.1.1522	Bacteria;Firmicutes;Clostridia;Lachnospirales;Lachnospiraceae;Blautia;uncultured_bacterium	100	0.0011083	0.00316837	0.00277346	0	0.00098733
OTU2031	FPLK01002810.11.1518	Bacteria;Bacteroidota;Bacteroidia;Flavobacteriales;Crocinitomicaceae;Fluviicola;metagenome	99.76	0	0	0.00138673	0.00102209	0
OTU2034	AF522998.1.1493	Bacteria;Proteobacteria;Gammaproteobacteria;Burkholderiales;Comamonadaceae;Aquabacterium;uncultured_Aquabacterium_sp.	100	0	0.00105612	0	0	0
OTU2035	AM991241.1.1448	Bacteria;Bacteroidota;Bacteroidia;Sphingobacteriales;AKYH767;uncultured_bacterium	99.29	0	0.00105612	0	0	0
OTU2036	HM187047.1.1427	Bacteria;Verrucomicrobiota;Omnitrophia;Omnitrophales;Omnitrophaceae;Candidatus_Omnitrophus;uncultured_bacterium	94.64	0	0.00105612	0	0	0
OTU204	AY752131.1.1412	Bacteria;Actinobacteriota;Actinobacteria;Micrococcales;Microbacteriaceae;Candidatus_Limnoluna;uncultured_bacterium	99.51	0.00443321	0.00422449	0.01248059	0.00204417	0.00098733
OTU2040	HM316162.1.1353	Bacteria;Bacteroidota;Bacteroidia;Flavobacteriales;Crocinitomicaceae;Crocinitomix;uncultured_bacterium	88.18	0	0	0	0	0.00098733
OTU2041	FJ542862.1.1481	Bacteria;Bdellovibrionota;Bdellovibrionia;Bdellovibrionales;Bdellovibrionaceae;Bdellovibrio;uncultured_Bdellovibrio_sp.	99.75	0	0.00105612	0	0	0
OTU2045	KX123464.1.1579	Bacteria;Patescibacteria;Parcubacteria;Candidatus_Jorgensenbacteria;Candidatus_Adlerbacteria_bacterium_GW2011_GWC1_50_9	88.5	0.0011083	0	0	0	0.00098733
OTU205	AF195877.1.1457	Bacteria;Proteobacteria;Gammaproteobacteria;Pseudomonadales;Pseudomonadaceae;Pseudomonas;Pseudomonas_sp._J1	100	0.00443321	0.00528061	0.50754382	0.00306626	0.64176614
OTU2051	JN232899.1.1481	Bacteria;Bacteroidota;Bacteroidia;Flavobacteriales;Flavobacteriaceae;Flavobacterium;uncultured_bacterium	100	0.0011083	0	0.00277346	0.00102209	0
OTU2054	MHOD01000002.3577.5272	Bacteria;Patescibacteria;Parcubacteria;Candidatus_Portnoybacteria;Candidatus_Spechtbacteria_bacterium_RIFCSPHIGHO2_01_FTLL_43_30	79.86	0.0011083	0.00105612	0	0	0
OTU2055	ET090709.1.1445	Bacteria;Verrucomicrobiota;Chlamydiae;Chlamydiales;Simkaniaceae;Candidatus_Renichlamydia;uncultured_Candidatus_Rhabdochlamydia_sp.	97.67	0	0	0.00970712	0	0
OTU2056	AB008814.1.1440	Bacteria;Proteobacteria;Alphaproteobacteria;Sphingomonadales;Sphingomonadaceae;Erythrobacter;alpha_proteobacterium_MBIC3030	100	0	0.00105612	0	0	0
OTU2057	CP012157.1819446.1820984	Bacteria;Proteobacteria;Gammaproteobacteria;Burkholderiales;Nitrosomonadaceae;Ellin6067;Betaproteobacteria_bacterium_TKL13-2	99.3	0.0011083	0	0	0	0
OTU206	FPLS01036612.10.1469	Bacteria;Proteobacteria;Alphaproteobacteria;Caulobacterales;Caulobacteraceae;Phenylobacterium;metagenome	100	0.00221661	0.00105612	0.00138673	0	0.00197467
OTU2060	HM185936.1.1403	Bacteria;Nitrospirota;Leptospirillia;Leptospirillales;Leptospirillaceae;Leptospirillum;uncultured_bacterium	98.83	0	0.00105612	0	0.00102209	0
OTU2061	JF265987.1.1391	Bacteria;Methylomirabilota;Methylomirabilia;Rokubacteriales;uncultured_bacterium	99.3	0	0	0.00138673	0	0
OTU2063	JF703341.1.1433	Bacteria;Proteobacteria;Gammaproteobacteria;Acidiferrobacterales;Acidiferrobacteraceae;Sulfurifustis;uncultured_proteobacterium	99.06	0	0	0.00138673	0	0
OTU2064	AF202056.1.1508	Bacteria;Firmicutes;Bacilli;Bacillales;Planococcaceae;Sporosarcina;Sporosarcina_aquimarina	99.77	0	0	0	0.08483325	0
OTU2066	JQ278884.1.1533	Bacteria;Verrucomicrobiota;Omnitrophia;Omnitrophales;Omnitrophaceae;Candidatus_Omnitrophus;uncultured_bacterium	93.26	0.00221661	0	0	0	0
OTU2067	JN453475.1.1320	Bacteria;Bacteroidota;Bacteroidia;Bacteroidales;SB-5;uncultured_organism	92.42	0	0	0	0.00102209	0
OTU2068	HM187158.1.1448	Bacteria;Acidobacteriota;Subgroup_5;uncultured_bacterium	96.83	0.0011083	0	0	0	0
OTU2069	KX123380.1.2523	Bacteria;Patescibacteria;Parcubacteria;Candidatus_Jorgensenbacteria;Candidatus_Jorgensenbacteria_bacterium_GW2011_GWA2_45_13	82.37	0.0011083	0	0	0	0
OTU207	JQ867302.1.1435	Bacteria;Nitrospirota;Leptospirillia;Leptospirillales;Leptospirillaceae;Leptospirillum;Candidatus_Troglogloea_absoloni	96.04	0.0011083	0.00105612	0.00277346	0.00408835	0
OTU2071	KY194033.1.1438	Bacteria;Chloroflexi;Dehalococcoidia;SAR202_clade;uncultured_bacterium	95.29	0	0	0.00138673	0	0
OTU2073	FJ763789.1.1413	Bacteria;Cyanobacteria;Cyanobacteriia;Synechococcales;Cyanobiaceae;Cyanobium_PCC-6307;Synechococcus_sp._BE0807I	100	0	0	0.00138673	0	0
OTU2075	GQ927604.1.916	Archaea;Aenigmarchaeota;Aenigmarchaeia;Aenigmarchaeales;uncultured_archaeon	87.09	0	0	0.00138673	0	0
OTU2078	MF040398.1.1476	Bacteria;Bacteroidota;Bacteroidia;Flavobacteriales;Cryomorphaceae;uncultured;uncultured_bacterium	100	0.0011083	0.00105612	0.00138673	0	0
OTU208	KF836147.1.1531	Bacteria;Nitrospirota;Leptospirillia;Leptospirillales;Leptospirillaceae;Leptospirillum;uncultured_bacterium	93.46	0	0.00105612	0.00138673	0.00102209	0.00197467
OTU2082	JQ278884.1.1533	Bacteria;Verrucomicrobiota;Omnitrophia;Omnitrophales;Omnitrophaceae;Candidatus_Omnitrophus;uncultured_bacterium	91.53	0	0	0	0.00102209	0
OTU2083	AF447767.1.1445	Bacteria;Actinobacteriota;Actinobacteria;Micrococcales;Microbacteriaceae;uncultured;uncultured_actinobacterium	100	0	0.00105612	0	0	0
OTU2085	AB746421.1.1449	Bacteria;Proteobacteria;Gammaproteobacteria;Pseudomonadales;Pseudomonadaceae;Pseudomonas;bacterium_endosymbiont_of_Curculio_koreanus	100	0.0011083	0	0.11093854	0	0.01086066
OTU2086	HM186395.1.1370	Bacteria;Acidobacteriota;Acidobacteriae;Subgroup_2;uncultured_bacterium	98.08	0	0	0.00138673	0	0
OTU2087	AB694455.1.1350	Bacteria;Patescibacteria;Parcubacteria;Candidatus_Portnoybacteria;uncultured_bacterium	84.45	0	0	0	0	0.00098733

OTU2088	KF836218.1.1353	Bacteria;Patescibacteria;Parcubacteria;Candidatus_Yanofskybacteria;uncultured_bacterium	79.65	0	0	0	0	0.00098733
OTU209	ET703418.1.1485	Bacteria;Bacteroidota;Bacteroidia;Flavobacteriales;Cryomorphaceae;uncultured;uncultured_Cryomorphaceae_bacterium	100	0	0	0.00693366	0	0.00098733
OTU2092	JX441387.1.1480	Bacteria;Actinobacteriota;Acidimicrobiia;Microtrichales;uncultured;uncultured_bacterium	99.75	0	0	0.00138673	0.00204417	0.00098733
OTU2094	AB490786.1.1488	Bacteria;Firmicutes;Bacilli;Bacillales;Planococcaceae;Paenisporosarcina;Sporosarcina_sp._MB6	99.06	0	0	0.00277346	0.10527499	0.11749257
OTU21	FPLS01013444.12.1478	Bacteria;Proteobacteria;Gammaproteobacteria;Burkholderiales;Comamonadaceae;uncultured;metagenome	97.89	0.01551625	0.01584184	0.01525405	0.02350801	0.03258197
OTU210	ET385709.1.1495	Bacteria;Nitrospirota;Thermodesulfovibrionia;uncultured;uncultured_bacterium	94.72	0.0011083	0.00105612	0	0.00102209	0.00098733
OTU2104	JN672018.1.1292	Bacteria;Elusimicrobiota;Elusimicrobia;Lineage_IV;uncultured_bacterium	96.62	0	0.00105612	0	0	0.00098733
OTU2109	AM409361.1.1463	Bacteria;Actinobacteriota;Actinobacteria;Micrococcales;Micrococcaceae;Pseudarthrobacter;Pseudarthrobacter_defluvii	100	0	0.00105612	0	0	0.00098733
OTU211	KF596564.1.1479	Bacteria;Actinobacteriota;Acidimicrobiia;Microtrichales;Ilumatobacteraceae;CL500-29_marine_group;uncultured_bacterium	99.75	0.0011083	0.00105612	0	0.00102209	0.00394933
OTU2114	MHGG01000050.25339.26910	Bacteria;Verrucomicrobiota;Omnitrophia;Omnitrophales;Omnitrophaceae;Candidatus_Omnitrophus;Omnitrophica_WOR_2_bacterium_GWF2_63_9	92.31	0	0	0.00138673	0	0
OTU2115	HM187269.1.1473	Bacteria;Planctomycetota;Phycisphaerae;CCM11a;uncultured_bacterium	98.26	0	0.00105612	0	0	0
OTU2117	KY190559.1.1448	Bacteria;Elusimicrobiota;Elusimicrobia;Lineage_IV;uncultured_bacterium	99.76	0	0.00105612	0	0	0
OTU2118	AB021381.1.1503	Bacteria;Proteobacteria;Gammaproteobacteria;Pseudomonadales;Pseudomonadaceae;Pseudomonas;Pseudomonas_fuscovaginae	99.53	0	0	0	0.00102209	0.10663191
OTU2119	LN870984.1.1513	Bacteria;Patescibacteria;Parcubacteria;Candidatus_Nomurabacteria;uncultured_bacterium	95.06	0	0	0	0	0.00098733
OTU212	AB233326.1.1454	Bacteria;Firmicutes;Bacilli;Staphylococcales;Staphylococcaceae;Staphylococcus;Staphylococcus_hominis_subsp._novobiosepticus	100	0.00332491	0	0.00277346	0.00204417	0.00197467
OTU2122	JF922476.1.1470	Bacteria;Cyanobacteria;Vampirivibrionia;Obscuribacterales;Obscuribacteraceae;uncultured_bacterium	100	0	0.00105612	0	0	0
OTU2124	CXWL01024582.15347.16848	Bacteria;Actinobacteriota;Acidimicrobiia;Microtrichales;Ilumatobacteraceae;CL500-29_marine_group;groundwater_metagenome	100	0	0	0.00138673	0	0
OTU2126	MF942642.1.1390	Bacteria;Verrucomicrobiota;Omnitrophia;Omnitrophales;Omnitrophaceae;Candidatus_Omnitrophus;uncultured_bacterium	91.61	0	0.00105612	0	0	0
OTU2129	KJ566470.1.959	Archaea;Nanoarchaeota;Nanoarchaeia;Woesearchaeales;uncultured_euryarchaeote	89.69	0.0011083	0	0	0	0
OTU2131	AB364882.1.1417	Bacteria;Patescibacteria;Parcubacteria;uncultured_bacterium	80.56	0.0011083	0	0	0	0
OTU2132	DQ836756.1.1370	Bacteria;Proteobacteria;Gammaproteobacteria;Ectothiorhodospirales;Thioalkalispiraceae;Thioalkalispira-Sulfurivermis;uncultured_bacterium	86.89	0.0011083	0.00105612	0	0	0
OTU2134	GT359064.1.1480	Bacteria;Verrucomicrobiota;Verrucomicrobiae;Opitutales;Opitutaceae;Opitutus;uncultured_bacterium	98.59	0	0	0	0.00102209	0
OTU2135	KJ566514.1.1077	Archaea;Nanoarchaeota;Nanoarchaeia;Woesearchaeales;uncultured_euryarchaeote	96.33	0	0.00105612	0	0	0
OTU2136	AB294344.1.1468	Bacteria;Verrucomicrobiota;Omnitrophia;Omnitrophales;uncultured_bacterium	93.9	0.0011083	0	0	0	0
OTU214	KP686959.1.1438	Bacteria;Bacteroidota;Bacteroidia;Flavobacteriales;Flavobacteriaceae;Flavobacterium;uncultured_bacterium	99.76	0	0.00211224	0.04160195	0.00102209	0.002962
OTU2141	JF490034.1.1450	Bacteria;Proteobacteria;Alphaproteobacteria;Rhodospirillales;Magnetospirillaceae;uncultured;alpha_proteobacterium_TT-4	97.01	0	0.00211224	0.00138673	0	0
OTU2143	GQ472402.1.1482	Bacteria;Proteobacteria;Gammaproteobacteria;Xanthomonadales;Xanthomonadaceae;Arenimonas;uncultured_bacterium	100	0	0.00211224	0	0	0
OTU2146	MHGG01000050.25339.26910	Bacteria;Verrucomicrobiota;Omnitrophia;Omnitrophales;Omnitrophaceae;Candidatus_Omnitrophus;Omnitrophica_WOR_2_bacterium_GWF2_63_9	92.54	0.0011083	0	0	0	0.00098733
OTU2147	HM445498.1.1382	Bacteria;Verrucomicrobiota;Omnitrophia;Omnitrophales;Omnitrophaceae;Candidatus_Omnitrophus;uncultured_bacterium	87.88	0	0	0	0.00102209	0.00098733
OTU2148	AB720115.1.1385	Bacteria;Proteobacteria;Gammaproteobacteria;Burkholderiales;Gallionellaceae;Ferriphaselus;Ferriphaselus_amnicola	100	0	0	0.00277346	0	0
OTU215	FPLK01002690.9.1503	Bacteria;Actinobacteriota;Actinobacteria;Frankiales;Sporichthyaceae;hgcI_clade;metagenome	99.75	0.0011083	0.00528061	0.0041602	0.00204417	0.00098733
OTU2151	KC358393.1.1301	Bacteria;Verrucomicrobiota;Omnitrophia;Omnitrophales;Omnitrophaceae;Candidatus_Omnitrophus;uncultured_bacterium	91.61	0	0	0	0.00102209	0
OTU2153	FJ593904.1.1405	Bacteria;Bacteroidota;Bacteroidia;Flavobacteriales;Flavobacteriaceae;Flavobacterium;Flavobacterium_macrobrachii	99.53	0.0011083	0	0	0.00102209	0
OTU2154	HE602802.1.1436	Bacteria;Elusimicrobiota;Lineage_IIb;uncultured_bacterium	93.62	0	0.00105612	0	0.00102209	0
OTU2158	HE603186.1.1453	Bacteria;Verrucomicrobiota;Omnitrophia;Omnitrophales;Omnitrophaceae;Candidatus_Omnitrophus;uncultured_Firmicutes_bacterium	92.31	0	0.00105612	0	0	0
OTU2159	KC990421.1.1249	Bacteria;Patescibacteria;Parcubacteria;Candidatus_Azambacteria;uncultured_Parcubacteria_group_bacterium	92.49	0.0011083	0	0.00138673	0	0
OTU216	EF019106.1.1344	Bacteria;Proteobacteria;Alphaproteobacteria;Caulobacterales;Caulobacteraceae;Brevundimonas;uncultured_proteobacterium	100	0.00332491	0.00105612	0.0041602	0.00408835	0.00098733
OTU2161	HE650039.1.1310	Bacteria;Planctomycetota;Phycisphaerae;Phycisphaerales;Phycisphaeraceae;SM1A02;uncultured_bacterium	99.75	0.0011083	0	0	0	0
OTU2163	KF071197.1.1344	Bacteria;Actinobacteriota;Actinobacteria;Micrococcales;Intrasporangiaceae;Janibacter;uncultured_bacterium	100	0	0.00105612	0.00138673	0	0.00098733
OTU217	AY734239.1.1379	Bacteria;Nitrospirota;Leptospirillia;Leptospirillales;Leptospirillaceae;Leptospirillum;uncultured_bacterium	97.9	0	0	0	0	0.00098733
OTU2171	CP015378.146768.148325	Bacteria;Firmicutes;Bacilli;Bacillales;Bacillaceae;Fictibacillus;Fictibacillus_phosphorivorans	100	0	0	0	0.00102209	0.00197467
OTU2172	CP009280.1064126.1065690	Bacteria;Firmicutes;Bacilli;Paenibacillales;Paenibacillaceae;Paenibacillus;Paenibacillus_sp._FSL_P4-0081	100	0	0.00105612	0.04992234	0.06132524	0
OTU2176	KP308750.18552.19877	Archaea;Nanoarchaeota;Nanoarchaeia;Woesearchaeales;SCGC_AAA011-D5;uncultured_archaeon	84.8	0	0	0	0.00102209	0
OTU2177	MHGG01000050.25339.26910	Bacteria;Verrucomicrobiota;Omnitrophia;Omnitrophales;Omnitrophaceae;Candidatus_Omnitrophus;Omnitrophica_WOR_2_bacterium_GWF2_63_9	91.14	0	0	0	0	0.00098733
OTU218	AB599923.1.1356	Bacteria;Proteobacteria;Alphaproteobacteria;Sphingomonadales;Sphingomonadaceae;Novosphingobium;alpha_proteobacterium_INB22	100	0.00332491	0.00211224	0.00138673	0.00511044	0.00098733
OTU2180	AB360546.1.1550	Bacteria;Firmicutes;Bacilli;Paenibacillales;Paenibacillaceae;Paenibacillus;Paenibacillus_macquariensis_subsp._defensor	98.83	0	0	0.18027513	0.0562148	0
OTU2181	DQ499288.1.1481	Bacteria;Bdellovibrionota;Bdellovibrionia;Bdellovibrionales;Bdellovibrionaceae;OM27_clade;uncultured_bacterium	85.79	0	0.00105612	0	0	0
OTU2185	JF141755.1.1356	Bacteria;Fusobacteriota;Fusobacteriia;Fusobacteriales;Fusobacteriaceae;Fusobacterium;uncultured_bacterium	100	0	0.00105612	0	0	0
OTU2189	AB184058.1.1465	Bacteria;Actinobacteriota;Actinobacteria;Streptomycetales;Streptomycetaceae;Streptomyces;Streptomyces_gangtokensis	99.75	0.00221661	0.1182857	0.00693366	0.00102209	0.00098733
OTU219	DQ676441.1.1388	Bacteria;Patescibacteria;Parcubacteria;uncultured_Parcubacteria_group_bacterium	97.28	0.0011083	0.00105612	0.0041602	0.00102209	0
OTU2190	AM991241.1.1448	Bacteria;Bacteroidota;Bacteroidia;Sphingobacteriales;AKYH767;uncultured_bacterium	98.35	0	0	0	0	0.00098733
OTU2191	MEPS01000173.1634.3193	Bacteria;Bdellovibrionota;Oligoflexia;0319-6G20;Bdellovibrionales_bacterium_GWC1_52_8	94.63	0	0.00211224	0	0.00102209	0
OTU22	AB680483.1.1462	Bacteria;Proteobacteria;Gammaproteobacteria;Pseudomonadales;Pseudomonadaceae;Pseudomonas;Pseudomonas_putida	100	0.04654874	0.04646938	13.7341913	0.03168471	0.03159464
OTU2202	HM243797.1.1434	Bacteria;Patescibacteria;Gracilibacteria;Candidatus_Peribacteria;uncultured_bacterium	91.36	0	0.00211224	0	0	0
OTU2205	MFTV01000001.3080.4539	Bacteria;Patescibacteria;Parcubacteria;TBA9983;Candidatus_Nomurabacteria_bacterium_RIFCSPHIGHO2_02_40_30	95.44	0	0.00105612	0	0	0
OTU2207	MF942642.1.1390	Bacteria;Verrucomicrobiota;Omnitrophia;Omnitrophales;Omnitrophaceae;Candidatus_Omnitrophus;uncultured_bacterium	90.8	0.0011083	0	0	0	0
OTU2208	JF681401.1.1236	Bacteria;Patescibacteria;Saccharimonadia;Saccharimonadales;uncultured_bacterium	96.53	0	0	0	0	0.00098733
OTU2209	JX000953.1.1397	Bacteria;Patescibacteria;Parcubacteria;Candidatus_Yanofskybacteria;uncultured_bacterium	91.81	0.0011083	0	0	0	0
OTU221	MF040478.1.1461	Bacteria;Proteobacteria;Alphaproteobacteria;Acetobacterales;Acetobacteraceae;Roseomonas;uncultured_bacterium	99	0.00332491	0.00316837	0	0.00408835	0.002962
OTU2210	EF032738.1.1406	Bacteria;Proteobacteria;Alphaproteobacteria;Rickettsiales;SM2D12;uncultured_Alphaproteobacteria_bacterium	96.77	0.00221661	0	0	0	0
OTU2212	JQ278816.1.1534	Bacteria;Verrucomicrobiota;Omnitrophia;Omnitrophales;Omnitrophaceae;Candidatus_Omnitrophus;uncultured_bacterium	90.93	0	0	0	0	0.00098733
OTU2213	KX177630.1.1483	Bacteria;Campylobacterota;Campylobacteria;Campylobacterales;Arcobacteraceae;uncultured;uncultured_bacterium	78.91	0.0011083	0	0	0	0
OTU2215	AP012048.1097572.1099087	Bacteria;Campylobacterota;Campylobacteria;Campylobacterales;Arcobacteraceae;Pseudarcobacter;Arcobacter_sp._L	100	0.0011083	0.00105612	0	0	0
OTU2218	CP000949.5254580.5256108	Bacteria;Proteobacteria;Gammaproteobacteria;Pseudomonadales;Pseudomonadaceae;Pseudomonas;Pseudomonas_putida_W619	99.53	0	0.00105612	0.13173952	0	0
OTU2219	GQ214127.1.1414	Bacteria;Chloroflexi;Dehalococcoidia;S085;uncultured_bacterium	98.51	0.0011083	0	0	0	0
OTU2220	ET703169.1.1476	Bacteria;Bacteroidota;Bacteroidia;Flavobacteriales;Flavobacteriaceae;Flavobacterium;uncultured_Flavobacterium_sp.	99.05	0	0	0.00138673	0	0
OTU2221	EF520461.1.1498	Bacteria;Proteobacteria;Gammaproteobacteria;Burkholderiales;T34;uncultured_beta_proteobacterium	91.08	0	0	0.00277346	0	0.00098733

OTU2222	JN485075.1.1362	Bacteria;Patescibacteria;Parcubacteria;Candidatus_Kaiserbacteria;uncultured_organism	78.65	0	0.00105612	0	0	0
OTU2223	AF092548.1.1483	Bacteria;Firmicutes;Clostridia;Clostridiales;Clostridiaceae;Clostridium_sensu_stricto_1;Clostridium_gasigenes	100	0	0	0	0.12469465	0.00098733
OTU2225	JF703474.1.1437	Bacteria;Proteobacteria;Gammaproteobacteria;Acidiferrobacterales;Acidiferrobacteraceae;Sulfurifustis;uncultured_proteobacterium	99.06	0	0.00105612	0	0	0
OTU2226	AB981050.1.1478	Bacteria;Actinobacteriota;Actinobacteria;Pseudonocardiales;Pseudonocardiaceae;Pseudonocardia;Pseudonocardia_soli	99.75	0.0011083	0	0	0	0
OTU2227	HG969252.1.1474	Bacteria;Proteobacteria;Gammaproteobacteria;Pseudomonadales;Marinobacteraceae;Marinobacter;Tamilnaduibacter_salinus	94.61	0	0.00211224	0.00138673	0	0
OTU2230	MHGG01000050.25339.26910	Bacteria;Verrucomicrobiota;Omnitrophia;Omnitrophales;Omnitrophaceae;Candidatus_Omnitrophus;Omnitrophica_WOR_2_bacterium_GWF2_63_9	92.31	0	0	0.00138673	0	0
OTU2231	JN868974.1.1523	Bacteria;Proteobacteria;Gammaproteobacteria;Burkholderiales;Comamonadaceae;Limnohabitans;uncultured_bacterium	100	0	0.00105612	0.00277346	0.00102209	0
OTU2237	FJ482182.1.1476	Bacteria;Patescibacteria;Parcubacteria;Candidatus_Nomurabacteria;uncultured_Parcubacteria_group_bacterium	98.77	0	0	0.00277346	0	0
OTU2239	DQ856516.1.1473	Bacteria;Bacteroidota;Bacteroidia;Chitinophagales;uncultured;uncultured_bacterium	92.05	0	0.00105612	0.00138673	0	0
OTU224	KF836147.1.1531	Bacteria;Nitrospirota;Leptospirillia;Leptospirillales;Leptospirillaceae;Leptospirillum;uncultured_bacterium	91.59	0	0.00211224	0	0.00306626	0
OTU2245	AF236003.1.1443	Bacteria;Proteobacteria;Alphaproteobacteria;Caulobacterales;Caulobacteraceae;Phenylobacterium;alpha_proteobacterium_A0902	100	0.0011083	0	0	0.00102209	0
OTU2248	DQ067002.1.1411	Bacteria;Latescibacterota;Latescibacteria;Latescibacterales;Latescibacteraceae;uncultured_bacterium	99.3	0	0	0	0.00102209	0
OTU2249	MHOD01000002.3577.5272	Bacteria;Patescibacteria;Parcubacteria;Candidatus_Portnoybacteria;Candidatus_Spechtbacteria_bacterium_RIFCSPHIGHO2_01_FTLL_43_30	88.81	0	0	0	0	0.00098733
OTU225	KX163471.1.1290	Bacteria;Nitrospirota;Nitrospiria;Nitrospirales;Nitrospiraceae;Nitrospira;uncultured_bacterium	99.3	0	0.00211224	0.00554693	0.00204417	0.00098733
OTU2250	AB007415.1.1456	Bacteria;Actinobacteriota;Actinobacteria;Micrococcales;Microbacteriaceae;Microbacterium;Microbacterium_lacticum	99.75	0.00221661	0	0.00138673	0.00408835	0
OTU2256	KX172267.1.1394	Bacteria;Patescibacteria;Parcubacteria;Candidatus_Yanofskybacteria;uncultured_bacterium	89.73	0	0.00105612	0	0	0.00098733
OTU2257	GQ340324.1.1377	Bacteria;Actinobacteriota;Actinobacteria;Frankiales;Sporichthyaceae;hgcI_clade;uncultured_bacterium	98.77	0.00221661	0	0	0	0
OTU226	HM856394.1.1444	Bacteria;Bacteroidota;Bacteroidia;Sphingobacteriales;Sphingobacteriaceae;Solitalea;uncultured_Sphingobacteriales_bacterium	99.53	0.00443321	0.00105612	0.01109385	0.00102209	0
OTU2265	ET731078.1.1221	Archaea;Nanoarchaeota;Nanoarchaeia;Woesearchaeales;uncultured_euryarchaeote	81.72	0.0011083	0	0	0	0
OTU227	AB179661.1.1396	Bacteria;Patescibacteria;ABY1;Candidatus_Kerfeldbacteria;uncultured_bacterium	82.31	0	0.00105612	0.00138673	0.00204417	0
OTU2271	AJ416410.1.1482	Bacteria;Proteobacteria;Alphaproteobacteria;Sphingomonadales;Sphingomonadaceae;Sphingopyxis;Sphingopyxis_witflariensis	100	0.0011083	0	0	0.00102209	0.09478392
OTU2272	MGPE01000013.58031.59587	Bacteria;Bdellovibrionota;Oligoflexia;0319-6G20;Deltaproteobacteria_bacterium_GWA2_38_16	91.59	0	0	0	0	0.00098733
OTU2273	FJ793181.1.1465	Bacteria;Acidobacteriota;Acidobacteriae;Paludibaculum;uncultured_bacterium	98.76	0	0.00422449	0	0	0
OTU2274	AB023371.1.1468	Bacteria;Actinobacteriota;Actinobacteria;Micrococcales;Micrococcaceae;Micrococcus;Micrococcus_luteus	100	0	0	0.00138673	0.00204417	0
OTU2275	KX123380.1.2523	Bacteria;Patescibacteria;Parcubacteria;Candidatus_Jorgensenbacteria;Candidatus_Jorgensenbacteria_bacterium_GW2011_GWA2_45_13	83.9	0	0.00105612	0	0	0
OTU2276	JQ675520.1.1287	Bacteria;Cyanobacteria;Vampirivibrionia;Caenarcaniphilales;uncultured_bacterium	94.81	0	0	0	0	0.00098733
OTU228	FPLP01011424.11.1477	Bacteria;Proteobacteria;Alphaproteobacteria;Rhizobiales;Rhizobiaceae;Mesorhizobium;metagenome	100	0.00664982	0.00316837	0.00554693	0.00919879	0.00098733
OTU2281	KX123464.1.1579	Bacteria;Patescibacteria;Parcubacteria;Candidatus_Jorgensenbacteria;Candidatus_Adlerbacteria_bacterium_GW2011_GWC1_50_9	91.64	0.0011083	0	0	0	0
OTU2282	HQ403245.1.1488	Bacteria;Bacteroidota;Bacteroidia;Flavobacteriales;NS9_marine_group;uncultured_bacterium	94.33	0.0011083	0	0	0	0
OTU2284	KF616728.1.1486	Bacteria;Myxococcota;bacteriap25;uncultured_bacterium	90.26	0.0011083	0.00105612	0	0	0
OTU2285	MHST01000019.19787.21369	Bacteria;Patescibacteria;Parcubacteria;Candidatus_Terrybacteria;Candidatus_Terrybacteria_bacterium_RIFCSPHIGHO2_01_FTLL_58_15	89.16	0	0	0	0	0.00098733
OTU2289	KP308707.11687.14046	Archaea;Nanoarchaeota;Nanoarchaeia;Woesearchaeales;uncultured_archaeon	82.17	0	0	0.00138673	0	0
OTU229	FN824831.1.1299	Bacteria;Actinobacteriota;Actinobacteria;Micrococcales;Microbacteriaceae;Aurantimicrobium;uncultured_bacterium	100	0	0	0.00277346	0	0.00098733
OTU2290	GT118951.1.1445	Bacteria;Bdellovibrionota;Oligoflexia;Oligoflexales;Oligoflexaceae;uncultured;uncultured_bacterium	87.53	0.0011083	0	0	0	0
OTU2292	FJ425213.1.1434	Bacteria;Bacteroidota;Bacteroidia;Flavobacteriales;Flavobacteriaceae;Polaribacter;Polaribacter_gangjinensis	98.34	0	0	0.00138673	0	0
OTU2294	JQ278884.1.1533	Bacteria;Verrucomicrobiota;Omnitrophia;Omnitrophales;Omnitrophaceae;Candidatus_Omnitrophus;uncultured_bacterium	92.56	0	0	0.00138673	0	0
OTU2296	HM066571.1.1533	Bacteria;Bdellovibrionota;Oligoflexia;0319-6G20;uncultured_bacterium	92.97	0	0.00105612	0	0	0
OTU2299	KF093960.1.1375	Bacteria;Bdellovibrionota;Oligoflexia;0319-6G20;uncultured_bacterium	92.29	0.0011083	0.00105612	0.00138673	0	0.00098733
OTU23	AB025279.1.1421	Bacteria;Proteobacteria;Alphaproteobacteria;Sphingomonadales;Sphingomonadaceae;Sphingobium;Sphingomonas_sp._MBIC3020	99.5	0.01662455	0.01584184	0.01386732	0.0163534	0.01875932
OTU230	LCOK01000044.9216.10662	Bacteria;Patescibacteria;Parcubacteria;Candidatus_Ryanbacteria;Candidatus_Giovannonibacteria_bacterium_GW2011_GWB1_47_6b	81.35	0	0	0	0	0.00098733
OTU2301	FJ916090.1.1522	Bacteria;Bacteroidota;Bacteroidia;Chitinophagales;Chitinophagaceae;Dinghuibacter;uncultured_Bacteroidetes_bacterium	99.05	0.0011083	0	0	0.00102209	0
OTU2302	JF809687.1.1323	Bacteria;Patescibacteria;Berkelbacteria;uncultured_bacterium	98.25	0	0.00105612	0	0	0
OTU2304	MGDZ01000002.11263.12728	Bacteria;Patescibacteria;ABY1;Candidatus_Magasanikbacteria;Candidatus_Thrbacteria_bacterium_RIFCSPHIGHO2_02_FTLL_57_19	81.91	0	0	0.00138673	0	0
OTU2308	AB680972.1.1462	Bacteria;Proteobacteria;Gammaproteobacteria;Pseudomonadales;Pseudomonadaceae;Pseudomonas;Pseudomonas_fluorescens	98.13	0.09088088	0.08765815	0.23019747	0.55397132	0.00197467
OTU2309	MHGG01000050.25339.26910	Bacteria;Verrucomicrobiota;Omnitrophia;Omnitrophales;Omnitrophaceae;Candidatus_Omnitrophus;Omnitrophica_WOR_2_bacterium_GWF2_63_9	92.07	0	0	0	0	0.00098733
OTU231	AM490846.1.1395	Bacteria;Elusimicrobiota;Elusimicrobia;Elusimicrobiales;Elusimicrobiaceae;Elusimicrobium;Elusimicrobium_minutum	96.15	0	0.00105612	0.00277346	0	0
OTU2310	MF942653.1.1445	Bacteria;Verrucomicrobiota;Omnitrophia;Omnitrophales;Omnitrophaceae;Candidatus_Omnitrophus;uncultured_bacterium	92.07	0	0.00105612	0	0	0
OTU2313	KC633538.1.1495	Bacteria;Proteobacteria;Gammaproteobacteria;Burkholderiales;Comamonadaceae;Acidovorax;uncultured_beta_proteobacterium	99.77	0	0.00105612	0	0.00102209	0.00197467
OTU2315	AM490657.1.1517	Bacteria;Verrucomicrobiota;Verrucomicrobiae;Pedosphaerales;Pedosphaeraceae;DEV008;uncultured_bacterium	85.88	0	0	0	0.00102209	0.00098733
OTU232	CP017150.1369838.1371314	Bacteria;Actinobacteriota;Actinobacteria;Micrococcales;Brevibacteriaceae;Brevibacterium;Brevibacterium_aurantiacum	100	0.0011083	0	0	0	0
OTU2326	FPLP01006605.10.1527	Bacteria;Proteobacteria;Gammaproteobacteria;Burkholderiales;Nitrosomonadaceae;Nitrosomonas;metagenome	99.77	0	0.00105612	0	0	0.00098733
OTU233	AB109769.669.2189	Bacteria;Firmicutes;Clostridia;Peptostreptococcales-Tissierellales;Family_XI;Finegoldia;Finegoldia_magna	100	0.00332491	0.00211224	0.00277346	0.00102209	0
OTU2332	KP866212.1.1451	Bacteria;Bacteroidota;Bacteroidia;Flavobacteriales;Flavobacteriaceae;Flavobacterium;Flavobacterium_sp._HMF3121	99.53	0	0	0.00277346	0	0
OTU2333	FQ659751.1.1284	Bacteria;Proteobacteria;Alphaproteobacteria;Rhodobacterales;Rhodobacteraceae;Tabrizicola;uncultured_soil_bacterium	100	0.0011083	0.00105612	0.00554693	0	0.00197467
OTU2334	LCOK01000044.9216.10662	Bacteria;Patescibacteria;Parcubacteria;Candidatus_Ryanbacteria;Candidatus_Giovannonibacteria_bacterium_GW2011_GWB1_47_6b	90.37	0.0011083	0	0	0	0
OTU2336	JQ278816.1.1534	Bacteria;Verrucomicrobiota;Omnitrophia;Omnitrophales;Omnitrophaceae;Candidatus_Omnitrophus;uncultured_bacterium	91.86	0	0.00105612	0	0	0
OTU2339	AF480585.1.1435	Bacteria;Actinobacteriota;Actinobacteria;Corynebacteriales;Mycobacteriaceae;Mycobacterium;Mycobacterium_mucogenicum	100	0	0	0.00138673	0	0
OTU234	FPLS01017925.13.1507	Bacteria;Elusimicrobiota;Elusimicrobia;Lineage_IV;metagenome	98.79	0	0.00211224	0.00277346	0	0.00098733
OTU2342	JF344507.1.1479	Bacteria;Bacteroidota;Bacteroidia;Bacteroidetes_VC2.1_Bac22;uncultured_Bacteroidetes_bacterium	95.26	0	0	0.00138673	0	0
OTU2345	KC432036.1.1366	Bacteria;Patescibacteria;Parcubacteria;Candidatus_Nomurabacteria;uncultured_bacterium	87.58	0.0011083	0	0	0	0
OTU2348	FJ516920.1.1479	Bacteria;Bacteroidota;Bacteroidia;Bacteroidales;Prolixibacteraceae;uncultured;uncultured_Bacteroidetes_bacterium	99.76	0	0	0.00138673	0.00102209	0
OTU2349	JQ427236.1.1509	Bacteria;Myxococcota;Polyangia;Haliangiales;Haliangiaceae;Haliangium;uncultured_bacterium	97.42	0.0011083	0.00211224	0	0	0
OTU2350	FJ755773.1.1481	Bacteria;Verrucomicrobiota;Omnitrophia;Omnitrophales;Omnitrophaceae;Candidatus_Omnitrophus;uncultured_bacterium	88.52	0	0.00105612	0	0	0
OTU2354	KC009894.1.1266	Bacteria;Sva0485;uncultured_Desulfobulbaceae_bacterium	84.54	0	0.00105612	0	0	0
OTU2357	AB637280.1.1478	Bacteria;Actinobacteriota;Actinobacteria;Micrococcales;Microbacteriaceae;Marisediminicola;uncultured_bacterium	99.75	0.0011083	0	0	0	0.00098733
OTU2358	JQ675510.1.1289	Bacteria;Proteobacteria;Gammaproteobacteria;Acidiferrobacterales;Acidiferrobacteraceae;Sulfurifustis;uncultured_bacterium	97.65	0	0	0	0.00102209	0
OTU236	CP009281.142106.143669	Bacteria;Firmicutes;Bacilli;Paenibacillales;Paenibacillaceae;Paenibacillus;Paenibacillus_sp._FSL_R5-0345	100	0	0.00211224	0.57549368	0.82993489	0.00098733

OTU2362	MF942641.1.1510	Bacteria;Verrucomicrobiota;Omnitrophia;Omnitrophales;uncultured_bacterium	97.18	0	0.00105612	0	0	0
OTU2363	FJ612179.1.1424	Bacteria;Proteobacteria;Alphaproteobacteria;Rhodobacterales;Rhodobacteraceae;Rhodobacter;uncultured_bacterium	100	0	0	0.00138673	0	0
OTU2365	ET134908.1.1328	Bacteria;Verrucomicrobiota;Omnitrophia;Omnitrophales;Omnitrophaceae;Candidatus_Omnitrophus;uncultured_bacterium	90.21	0	0	0	0.00204417	0
OTU2367	FP929140.1090464.1091986	Bacteria;Proteobacteria;Gammaproteobacteria;Pseudomonadales;Halomonadaceae;HdN1;gamma_proteobacterium_HdN1	92.97	0.0011083	0	0	0.00102209	0
OTU237	MHGG01000050.25339.26910	Bacteria;Verrucomicrobiota;Omnitrophia;Omnitrophales;Omnitrophaceae;Candidatus_Omnitrophus;Omnitrophica_WOR_2_bacterium_GWF2_63_9	93.24	0	0	0.00138673	0	0
OTU2371	AF538712.1.1482	Bacteria;Proteobacteria;Alphaproteobacteria;Acetobacterales;Acetobacteraceae;Roseomonas;Roseomonas_mucosa	100	0	0.00105612	0	0	0
OTU2372	KY190626.1.1442	Bacteria;Actinobacteriota;Acidimicrobiia;Microtrichales;Ilumatobacteraceae;Ilumatobacter;uncultured_bacterium	99.75	0	0.00105612	0.00554693	0.00102209	0
OTU2373	GQ262975.1.1448	Bacteria;Chloroflexi;Gitt-GS-136;uncultured_bacterium	99.75	0	0	0	0.00102209	0
OTU2379	CP010350.26132.27676	Bacteria;Proteobacteria;Gammaproteobacteria;Pseudomonadales;Moraxellaceae;Acinetobacter;Acinetobacter_johnsonii_XBB1	99.77	0	0	0	0	0.00098733
OTU238	FPLS01007638.18.1486	Bacteria;Proteobacteria;Alphaproteobacteria;Rickettsiales;Mitochondria;metagenome	92.54	0.0011083	0.00316837	0.00277346	0.00102209	0
OTU2381	AB472943.1.1415	Bacteria;Proteobacteria;Gammaproteobacteria;Enterobacterales;Aeromonadaceae;Aeromonas;Aeromonas_sobria	100	0	0	0.00277346	0	0
OTU2382	FPLL01007970.1.1334	Bacteria;Proteobacteria;Alphaproteobacteria;Rickettsiales;Mitochondria;metagenome	92.79	0	0	0.00277346	0	0
OTU2384	GT583983.1.1480	Bacteria;Campylobacterota;Campylobacteria;Campylobacterales;Sulfurimonadaceae;Sulfurimonas;uncultured_bacterium	70.57	0	0.00105612	0	0	0
OTU2386	AY977333.1.1308	Bacteria;Firmicutes;Clostridia;Oscillospirales;Ruminococcaceae;Faecalibacterium;uncultured_bacterium	100	0	0.00105612	0	0.00102209	0.00098733
OTU2388	FNVZ01000002.45.1536	Bacteria;Fusobacteriota;Fusobacteriia;Fusobacteriales;Leptotrichiaceae;Leptotrichia;Leptotrichia_sp._Marseille-P3007	100	0	0	0.00138673	0	0
OTU2389	LCDF01000001.64365.66805	Bacteria;Patescibacteria;Parcubacteria;Candidatus_Giovannonibacteria;Candidatus_Giovannonibacteria_bacterium_GW2011_GWF2_42_19	87.87	0	0	0	0	0.00098733
OTU239	JN656867.1.1490	Bacteria;Bacteroidota;Bacteroidia;Chitinophagales;Chitinophagaceae;Sediminibacterium;uncultured_Bacteroidetes_bacterium	100	0.0011083	0.00211224	0.0041602	0	0
OTU2391	ACDY02000019.18010.19538	Bacteria;Proteobacteria;Gammaproteobacteria;Burkholderiales;Neisseriaceae;Neisseria;Neisseria_cinerea_ATCC_14685	100	0	0.00105612	0	0.00102209	0
OTU2392	JN672018.1.1292	Bacteria;Elusimicrobiota;Elusimicrobia;Lineage_IV;uncultured_bacterium	97.1	0	0	0	0	0.00098733
OTU2393	DQ181674.1.1465	Bacteria;Cyanobacteria;Cyanobacteriia;RD017;uncultured_cyanobacterium	91.11	0	0	0.0041602	0.00102209	0
OTU2399	JQ278816.1.1534	Bacteria;Verrucomicrobiota;Omnitrophia;Omnitrophales;Omnitrophaceae;Candidatus_Omnitrophus;uncultured_bacterium	91.86	0	0	0	0	0.00098733
OTU24	GT437440.1.1500	Bacteria;Bacteroidota;Bacteroidia;Flavobacteriales;Flavobacteriaceae;Flavobacterium;uncultured_bacterium	95.75	0.01108303	0.01267347	0.02912137	0.00613252	0.01086066
OTU240	GQ500746.1.1487	Bacteria;Nitrospirota;Nitrospiria;Nitrospirales;Nitrospiraceae;Nitrospira;uncultured_bacterium	99.52	0.00221661	0.00211224	0.00832039	0.00102209	0
OTU2402	CP016889.1470665.1472216	Bacteria;Proteobacteria;Gammaproteobacteria;Enterobacterales;Erwiniaceae;Pantoea;Pantoea_agglomerans	99.06	0	0.17109182	0.0041602	0	0.00098733
OTU2403	AB741661.1.1324	Bacteria;Proteobacteria;Gammaproteobacteria;Enterobacterales;Enterobacteriaceae;Citrobacter;Citrobacter_youngae	100	0.0011083	0	0	0.00102209	0.00098733
OTU241	KP686647.1.1446	Bacteria;Bacteroidota;Bacteroidia;Flavobacteriales;Crocinitomicaceae;Fluviicola;uncultured_bacterium	99.76	0.0011083	0.00105612	0.00138673	0.00204417	0.00098733
OTU2411	CP016889.1470665.1472216	Bacteria;Proteobacteria;Gammaproteobacteria;Enterobacterales;Erwiniaceae;Pantoea;Pantoea_agglomerans	99.53	0.00221661	0.09505101	0	0.00102209	0
OTU2412	KC358031.1.1241	Bacteria;Chloroflexi;Dehalococcoidia;SAR202_clade;uncultured_bacterium	98.76	0	0.00105612	0	0	0
OTU2418	MF942640.1.1495	Bacteria;Verrucomicrobiota;Omnitrophia;Omnitrophales;Omnitrophaceae;Candidatus_Omnitrophus;uncultured_bacterium	90.91	0.0011083	0	0.00138673	0	0
OTU2419	FJ484808.1.1365	Bacteria;Sva0485;uncultured_delta_proteobacterium	85.05	0	0.00105612	0	0	0
OTU242	HQ532763.1.1516	Bacteria;Actinobacteriota;Actinobacteria;Micrococcales;Microbacteriaceae;MWH-Ta3;uncultured_actinobacterium	99.26	0.00332491	0.00422449	0.02496117	0	0
OTU2422	JX222018.1.1446	Bacteria;Proteobacteria;Alphaproteobacteria;Caulobacterales;Caulobacteraceae;Caulobacter;uncultured_bacterium	99.5	0.00221661	0.00105612	0.00138673	0	0
OTU2426	GT118855.1.1485	Bacteria;Myxococcota;Polyangia;Polyangiales;Sandaracinaceae;uncultured;uncultured_bacterium	94.41	0	0.00105612	0.00138673	0	0
OTU243	FPLK01000798.18.1475	Bacteria;Verrucomicrobiota;Verrucomicrobiae;uncultured;metagenome	98.58	0.0011083	0.00211224	0	0.00204417	0
OTU2430	MF942640.1.1495	Bacteria;Verrucomicrobiota;Omnitrophia;Omnitrophales;Omnitrophaceae;Candidatus_Omnitrophus;uncultured_bacterium	82.75	0	0	0	0	0.00098733
OTU2431	FPLM01006002.16.1532	Bacteria;Proteobacteria;Gammaproteobacteria;Burkholderiales;Rhodocyclaceae;Dechloromonas;metagenome	99.53	0.0011083	0.00105612	0	0	0.00098733
OTU2433	AB294345.1.1480	Bacteria;Verrucomicrobiota;Omnitrophia;Omnitrophales;Omnitrophaceae;Candidatus_Omnitrophus;uncultured_bacterium	93.01	0	0	0	0.00204417	0
OTU2434	FPLS01013435.10.1398	Bacteria;Bdellovibrionota;Oligoflexia;0319-6G20;metagenome	98.59	0.0011083	0	0	0	0
OTU2435	JF417820.1.1499	Bacteria;Elusimicrobiota;Lineage_IIc;uncultured_bacterium	91.71	0.0011083	0	0	0	0
OTU2438	HM129010.1.1461	Bacteria;Verrucomicrobiota;Verrucomicrobiae;Verrucomicrobiales;Rubritaleaceae;Luteolibacter;uncultured_bacterium	100	0	0	0.00138673	0	0
OTU2439	AJ431636.1.1484	Bacteria;Proteobacteria;Alphaproteobacteria;Rhizobiales;Xanthobacteraceae;Bradyrhizobium;Bradyrhizobium_sp._TSDA_3475	100	0	0	0.00138673	0	0
OTU244	DQ289927.1.1347	Bacteria;Cyanobacteria;Cyanobacteriia;Cyanobacteriales;Microcystaceae;ST2_symbiont_group;uncultured_cyanobacterium	93.58	0.0011083	0	0.9915132	0.00102209	0.00098733
OTU2441	JX983992.1.1255	Bacteria;Dependentiae;Babeliae;Babeliales;uncultured_bacterium	90.87	0	0	0	0.00102209	0
OTU2442	AM991164.1.1470	Bacteria;Proteobacteria;Gammaproteobacteria;Xanthomonadales;Xanthomonadaceae;Lysobacter;uncultured_bacterium	99.06	0.0011083	0.00105612	0.00138673	0.06745776	0
OTU2443	AB930587.1.1463	Bacteria;Nitrospirota;Nitrospiria;Nitrospirales;Nitrospiraceae;Nitrospira;uncultured_bacterium	100	0.0011083	0.00105612	0.00138673	0	0
OTU2444	HQ119627.1.1476	Bacteria;Planctomycetota;Planctomycetes;Gemmatales;Gemmataceae;Gemmata;uncultured_bacterium	94.43	0	0.00105612	0.00138673	0.00102209	0
OTU245	KF836147.1.1531	Bacteria;Nitrospirota;Leptospirillia;Leptospirillales;Leptospirillaceae;Leptospirillum;uncultured_bacterium	91.36	0	0.00105612	0.00138673	0.00102209	0.00098733
OTU2452	FJ208846.1.1386	Bacteria;Planctomycetota;vadinHA49;uncultured_bacterium	93.47	0	0	0.00138673	0	0
OTU2456	KX622733.1.1381	Bacteria;Proteobacteria;Alphaproteobacteria;Rhizobiales;Rhizobiales_Incertae_Sedis;Phreatobacter;Alphaproteobacteria_bacterium	98.01	0	0	0.00138673	0	0
OTU2458	HM187000.1.1345	Bacteria;Chloroflexi;Dehalococcoidia;SAR202_clade;uncultured_bacterium	99.5	0	0	0.00138673	0	0
OTU2459	CP011868.1476357.1477890	Bacteria;Actinobacteriota;Actinobacteria;Pseudonocardiales;Pseudonocardiaceae;Pseudonocardia;Pseudonocardia_sp._HH130629-09	99.02	0.0011083	0	0	0	0.00098733
OTU246	HM187432.1.1403	Bacteria;Nitrospirota;Nitrospiria;Nitrospirales;Nitrospiraceae;Nitrospira;uncultured_bacterium	86.45	0	0.00211224	0	0	0
OTU2460	JX105608.1.1495	Bacteria;Proteobacteria;Gammaproteobacteria;Burkholderiales;Neisseriaceae;uncultured;uncultured_bacterium	93.91	0	0	0	0.00102209	0
OTU2466	KT324339.1.1531	Bacteria;Sva0485;uncultured_delta_proteobacterium	85.75	0	0	0	0.00204417	0
OTU2469	AB262725.1.1463	Bacteria;Acidobacteriota;Acidobacteriae;Subgroup_2;uncultured_bacterium	91.41	0	0	0.00138673	0	0
OTU247	CP016769.680246.681768	Bacteria;Actinobacteriota;Actinobacteria;Frankiales;Sporichthyaceae;hgcI_clade;Candidatus_Planktophila_lacus	100	0.00221661	0.00211224	0.00277346	0	0.00098733
OTU2471	GT472705.1.1441	Bacteria;Actinobacteriota;Actinobacteria;Corynebacteriales;Mycobacteriaceae;Mycobacterium;uncultured_Mycobacterium_sp.	99.75	0	0	0.00138673	0	0
OTU2476	ABVR01000038.1362.2880	Bacteria;Firmicutes;Clostridia;Lachnospirales;Lachnospiraceae;Coprococcus;Coprococcus_comes_ATCC_27758	99.75	0	0	0.00138673	0	0
OTU248	GT305787.1.1485	Bacteria;Actinobacteriota;Actinobacteria;Micrococcales;Microbacteriaceae;Rhodoluna;uncultured_bacterium	100	0.00443321	0.00316837	0.00554693	0	0.00098733
OTU2483	AB551476.1.1303	Bacteria;Cyanobacteria;Cyanobacteriia;Cyanobacteriales;Nostocaceae;Dolichospermum_NIES41;Dolichospermum_spiroides_TAC551	100	0	0.00105612	0	0	0
OTU2486	FJ901820.1.1378	Bacteria;MBNT15;uncultured_delta_proteobacterium	81.59	0	0.00105612	0.00138673	0	0
OTU2488	AB100738.1.1439	Bacteria;Bacteroidota;Bacteroidia;Sphingobacteriales;Sphingobacteriaceae;Sphingobacterium;Sphingobacterium_multivorum	100	0	0	0.00138673	0	0
OTU2494	FJ710698.1.1431	Bacteria;Patescibacteria;Parcubacteria;Candidatus_Vogelbacteria;uncultured_bacterium	83.69	0.0011083	0	0	0	0
OTU2497	FPLS01008338.8.1471	Bacteria;Proteobacteria;Alphaproteobacteria;Rhizobiales;Hyphomicrobiaceae;Pedomicrobium;metagenome	99.5	0.0011083	0.00105612	0	0	0
OTU2499	FJ484483.1.1366	Bacteria;Proteobacteria;Gammaproteobacteria;Beggiatoales;Beggiatoaceae;uncultured;uncultured_proteobacterium	97.42	0	0	0	0.00102209	0
OTU25	AB722254.1.1352	Bacteria;Bacteroidota;Bacteroidia;Sphingobacteriales;env.OPS_17;uncultured_bacterium	98.58	0.00554152	0.02217857	0.01525405	0.01430922	0.00789866
OTU250	CP029482.347290.348829	Bacteria;Proteobacteria;Gammaproteobacteria;Pseudomonadales;Pseudomonadaceae;Pseudomonas;Pseudomonas_sp._31-12	100	0.04322383	0.00316837	0.47287553	0.00408835	0.5282229

OTU2500	ET266843.1.1528	Bacteria;MBNT15;uncultured_Syntrophaceae_bacterium	98.59	0	0.00211224	0	0	0
OTU2501	ET937952.1.1478	Bacteria;Dependentiae;Babeliae;Babeliales;uncultured_bacterium	92.04	0	0	0.00138673	0	0
OTU2504	HM129252.1.1436	Bacteria;Bacteroidota;Bacteroidia;Flavobacteriales;Flavobacteriaceae;Flavobacterium;uncultured_bacterium	100	0	0	0.00138673	0	0
OTU2507	FJ482182.1.1476	Bacteria;Patescibacteria;Parcubacteria;Candidatus_Nomurabacteria;uncultured_Parcubacteria_group_bacterium	98.53	0	0.00105612	0	0	0
OTU251	CP002876.632833.634358	Bacteria;Proteobacteria;Gammaproteobacteria;Burkholderiales;Nitrosomonadaceae;Nitrosomonas;Nitrosomonas_sp._Is79A3	99.3	0.00221661	0.00211224	0.0041602	0.00306626	0
OTU2514	AM179866.1.1323	Bacteria;Bacteroidota;Bacteroidia;Flavobacteriales;Flavobacteriaceae;Flavobacterium;Sporocytophaga_sp._A61	100	0.0011083	0.0095051	0.0041602	0.00204417	0
OTU2515	HE603186.1.1453	Bacteria;Verrucomicrobiota;Omnitrophia;Omnitrophales;Omnitrophaceae;Candidatus_Omnitrophus;uncultured_Firmicutes_bacterium	89.51	0	0	0.00138673	0	0
OTU2519	KY287410.1.1494	Bacteria;Bacteroidota;Bacteroidia;Chitinophagales;Chitinophagaceae;uncultured;uncultured_bacterium	99.29	0.0011083	0	0	0	0
OTU252	ET117805.1.1508	Bacteria;Actinobacteriota;Actinobacteria;Frankiales;Sporichthyaceae;Candidatus_Planktophila;uncultured_actinobacterium	100	0.00332491	0.00211224	0.01664078	0	0.00197467
OTU2520	HM128975.1.1408	Bacteria;Proteobacteria;Alphaproteobacteria;Acetobacterales;Acetobacteraceae;Roseomonas;uncultured_bacterium	99.75	0.0011083	0	0	0	0
OTU2524	HM187090.1.1449	Bacteria;Planctomycetota;Pla4_lineage;uncultured_bacterium	92.8	0	0	0.00138673	0	0
OTU2525	AY625139.1.1427	Bacteria;Proteobacteria;Alphaproteobacteria;Caulobacterales;Parvularculaceae;Amphiplicatus;uncultured_bacterium	99.5	0	0.00105612	0	0	0
OTU2526	HQ904237.1.1498	Bacteria;Proteobacteria;Alphaproteobacteria;Rhodobacterales;Rhodobacteraceae;Rhodobacter;uncultured_bacterium	99.01	0	0	0.00138673	0	0.00098733
OTU2529	CP016889.1470665.1472216	Bacteria;Proteobacteria;Gammaproteobacteria;Enterobacterales;Erwiniaceae;Pantoea;Pantoea_agglomerans	99.3	0.00221661	0.11194897	0.00138673	0	0
OTU253	KC424686.1.1518	Bacteria;Bacteroidota;Bacteroidia;Sphingobacteriales;env.OPS_17;uncultured_bacterium	100	0	0	0.00138673	0	0
OTU2530	AJ605292.1.1510	Bacteria;Firmicutes;Bacilli;Paenibacillales;Paenibacillaceae;Paenibacillus;Paenibacillus_antarcticus	96.95	0.12745489	0.00211224	0.69475261	0.22485921	0.14118855
OTU2537	AB245382.1.1495	Bacteria;Firmicutes;Bacilli;Paenibacillales;Paenibacillaceae;Paenibacillus;Paenibacillus_anaericanus	99.06	0	0	0.07765698	0.11651795	0
OTU254	MHJF01000011.24156.25754	Bacteria;Patescibacteria;Parcubacteria;Candidatus_Colwellbacteria;Candidatus_Harrisonbacteria_bacterium_RIFCSPHIGHO2_02_FTLL_40_20	84.62	0.0011083	0	0	0	0
OTU2540	FR749930.1.1533	Bacteria;Spirochaetota;Spirochaetia;Spirochaetales;Spirochaetaceae;Spirochaeta_2;Spirochaeta_isovalerica	87.59	0.0011083	0	0	0	0
OTU2547	FJ902622.1.1328	Bacteria;Cyanobacteria;Cyanobacteriia;Cyanobacteriales;Geitlerinemaceae;Geitlerinema_PCC-7105;uncultured_cyanobacterium	99.75	0	0	0.00138673	0	0
OTU2548	DQ128629.1.1410	Bacteria;Patescibacteria;Parcubacteria;Candidatus_Nomurabacteria;uncultured_soil_bacterium	91.63	0	0	0.00277346	0	0
OTU255	CP012156.636959.638430	Bacteria;Proteobacteria;Alphaproteobacteria;Caulobacterales;Hyphomonadaceae;TKL13-1;Hyphomonadaceae_bacterium_TKL13-1	100	0.0011083	0.00211224	0	0.00306626	0
OTU2550	LCOX01000043.1193.4545	Bacteria;Patescibacteria;Parcubacteria;Candidatus_Jorgensenbacteria;Parcubacteria_group_bacterium_GW2011_GWA2_47_9	80.05	0	0	0	0	0.00098733
OTU2558	KX123464.1.1579	Bacteria;Patescibacteria;Parcubacteria;Candidatus_Jorgensenbacteria;Candidatus_Adlerbacteria_bacterium_GW2011_GWC1_50_9	88.5	0	0	0	0	0.00098733
OTU2559	HM187255.1.1471	Bacteria;Proteobacteria;Gammaproteobacteria;Burkholderiales;Nitrosomonadaceae;MND1;uncultured_bacterium	98.59	0	0	0	0.00102209	0
OTU256	FPLK01001426.10.1481	Bacteria;Proteobacteria;Alphaproteobacteria;Rhizobiales;Rhizobiales_Incertae_Sedis;uncultured;metagenome	100	0.0011083	0.00528061	0.00277346	0.00204417	0.00197467
OTU2560	MG603752.1.1383	Bacteria;Bacteroidota;Bacteroidia;Flavobacteriales;Flavobacteriaceae;Flavobacterium;Flavobacterium_sp._TSK36	96.68	0	0	0	0.00102209	0
OTU2564	FPLK01002810.11.1518	Bacteria;Bacteroidota;Bacteroidia;Flavobacteriales;Crocinitomicaceae;Fluviicola;metagenome	99.53	0	0	0	0.00102209	0
OTU2565	AJ863179.1.1477	Bacteria;Firmicutes;Bacilli;Bacillales;Planococcaceae;Paenisporosarcina;uncultured_bacterium	100	0.0011083	0.00633673	0.00693366	0.15229101	0.00197467
OTU2569	AY509446.1.1463	Bacteria;Proteobacteria;Gammaproteobacteria;Burkholderiales;Oxalobacteraceae;uncultured;uncultured_beta_proteobacterium	99.53	0.0011083	0	0.00277346	0	0.00098733
OTU257	ET134909.1.1431	Bacteria;Verrucomicrobiota;Omnitrophia;Omnitrophales;Omnitrophaceae;Candidatus_Omnitrophus;uncultured_bacterium	89.98	0	0.00105612	0	0	0.00197467
OTU2578	AY454638.1.1047	Archaea;Nanoarchaeota;Nanoarchaeia;Woesearchaeales;uncultured_crenarchaeote	81.92	0	0.00211224	0	0	0
OTU258	MGTY01000053.17208.18715	Bacteria;Elusimicrobiota;Elusimicrobia;Lineage_IV;Elusimicrobia_bacterium_GWA2_69_24	91.11	0.00221661	0.00211224	0.00138673	0.00102209	0.00098733
OTU2583	HM445498.1.1382	Bacteria;Verrucomicrobiota;Omnitrophia;Omnitrophales;Omnitrophaceae;Candidatus_Omnitrophus;uncultured_bacterium	87.88	0	0	0	0	0.00098733
OTU2584	EF190151.1.1478	Bacteria;Bacteroidota;Bacteroidia;Flavobacteriales;Flavobacteriaceae;Flavobacterium;uncultured_bacterium	98.58	0	0	0	0	0.00098733
OTU2586	MFTV01000001.3080.4539	Bacteria;Patescibacteria;Parcubacteria;TBA9983;Candidatus_Nomurabacteria_bacterium_RIFCSPHIGHO2_02_40_30	92.89	0.0011083	0	0	0	0
OTU2588	KF616728.1.1486	Bacteria;Myxococcota;bacteriap25;uncultured_bacterium	93.94	0	0	0	0	0.00098733
OTU259	LCQK01000003.64973.66448	Bacteria;Patescibacteria;Parcubacteria;Candidatus_Jorgensenbacteria;Candidatus_Jorgensenbacteria_bacterium_GW2011_GWB1_50_10	88.86	0	0.00211224	0	0.00102209	0.00197467
OTU2594	CXWJ01006813.2949.4408	Bacteria;Nitrospirota;Nitrospiria;Nitrospirales;Nitrospiraceae;Nitrospira;wastewater_metagenome	100	0	0	0	0	0.00098733
OTU2595	CXWL01054502.320.1767	Bacteria;Elusimicrobiota;Elusimicrobia;Lineage_IV;groundwater_metagenome	95.17	0	0	0.00138673	0	0
OTU2598	MF942642.1.1390	Bacteria;Verrucomicrobiota;Omnitrophia;Omnitrophales;Omnitrophaceae;Candidatus_Omnitrophus;uncultured_bacterium	93.24	0.0011083	0	0	0	0
OTU26	ARBA01000001.2818.4335	Bacteria;Proteobacteria;Gammaproteobacteria;Burkholderiales;Methylophilaceae;Methylotenera;Methylotenera_mobilis_13	100	0.03546571	0.02640306	0.01386732	0.01737548	0.01974665
OTU260	JF235891.1.1295	Bacteria;Patescibacteria;Parcubacteria;Candidatus_Adlerbacteria;uncultured_bacterium	90.45	0	0.00105612	0	0.00204417	0
OTU2600	MHJF01000011.24156.25754	Bacteria;Patescibacteria;Parcubacteria;Candidatus_Colwellbacteria;Candidatus_Harrisonbacteria_bacterium_RIFCSPHIGHO2_02_FTLL_40_20	83.13	0.0011083	0	0	0	0
OTU2602	MF942639.1.1600	Bacteria;Verrucomicrobiota;Omnitrophia;Omnitrophales;uncultured_bacterium	93.69	0	0.00105612	0	0	0
OTU2604	GQ389172.1.1503	Bacteria;Proteobacteria;Gammaproteobacteria;Burkholderiales;Comamonadaceae;Rhodoferax;uncultured_bacterium	99.77	0	0.00105612	0	0	0.06220195
OTU2608	JN038245.1.1475	Bacteria;Chloroflexi;Dehalococcoidia;S085;uncultured_bacterium	92.33	0.0011083	0	0	0	0
OTU261	KY356869.1.921	Archaea;Nanoarchaeota;Nanoarchaeia;Woesearchaeales;GW2011_GWC1_47_15;uncultured_archaeon	86.88	0	0.00105612	0	0	0
OTU2611	DQ129259.1.1486	Bacteria;Proteobacteria;Gammaproteobacteria;Burkholderiales;Neisseriaceae;uncultured;uncultured_bacterium	95.55	0.00221661	0	0	0	0.00098733
OTU2613	KP196827.1.1401	Bacteria;Proteobacteria;Gammaproteobacteria;Burkholderiales;Oxalobacteraceae;Tndibacterium;Tndibacterium_parvum	98.36	0.0011083	0	0	0	0
OTU2614	JF833844.1.1522	Bacteria;Entotheonellaeota;Entotheonellia;Entotheonellales;Entotheonellaceae;uncultured_delta_proteobacterium	97.2	0	0	0	0	0.00098733
OTU2617	JX227646.1.1538	Bacteria;Verrucomicrobiota;Omnitrophia;Omnitrophales;Omnitrophaceae;Candidatus_Omnitrophus;uncultured_bacterium	87.21	0	0.00316837	0	0	0
OTU2619	LBVI01000009.76614.78085	Bacteria;Patescibacteria;Parcubacteria;Candidatus_Nomurabacteria;Parcubacteria_group_bacterium_GW2011_GWF2_38_76	88.4	0.0011083	0	0	0	0
OTU262	AF188300.1.1463	Bacteria;Proteobacteria;Gammaproteobacteria;Pseudomonadales;Moraxellaceae;Acinetobacter;Acinetobacter_johnsonii	100	0.00886643	0.00211224	0.0041602	0.00102209	0.002962
OTU2622	KC253284.1.1489	Bacteria;Bacteroidota;Bacteroidia;Flavobacteriales;Crocinitomicaceae;Fluviicola;uncultured_bacterium	97.87	0.00221661	0	0	0	0
OTU2623	AB680535.1.1473	Bacteria;Firmicutes;Bacilli;Lactobacillales;Streptococcaceae;Streptococcus;Streptococcus_salivarius_subsp._thermophilus	100	0	0	0.00138673	0	0
OTU263	ET117582.1.1509	Bacteria;Actinobacteriota;Actinobacteria;Frankiales;Sporichthyaceae;Candidatus_Planktophila;uncultured_actinobacterium	99.75	0.0011083	0.00105612	0.04853561	0.00102209	0
OTU2630	HM187267.1.1481	Bacteria;Acidobacteriota;Subgroup_22;uncultured_bacterium	98.13	0	0	0.00138673	0	0
OTU2633	JX971550.1.1370	Bacteria;Bacteroidota;Bacteroidia;Sphingobacteriales;Sphingobacteriaceae;Pedobacter;Pedobacter_boryungensis	100	0.0011083	0	0	0.00102209	0.07306261
OTU2635	MHJF01000011.24156.25754	Bacteria;Patescibacteria;Parcubacteria;Candidatus_Colwellbacteria;Candidatus_Harrisonbacteria_bacterium_RIFCSPHIGHO2_02_FTLL_40_20	76.4	0.0011083	0	0	0	0
OTU2638	AB179508.1.1524	Bacteria;Methylomirabilota;Methylomirabilia;Methylomirabilales;Methylomirabilaceae;MIZ17;uncultured_bacterium	99.3	0	0	0	0.00102209	0.00098733
OTU264	AB680959.1.1462	Bacteria;Proteobacteria;Gammaproteobacteria;Pseudomonadales;Pseudomonadaceae;Pseudomonas;Agrobacterium_agile	99.53	0.0011083	0	0.00138673	0.00102209	0.70001876
OTU2640	JX096997.1.1467	Bacteria;Actinobacteriota;Actinobacteria;Micrococcales;Micrococcaceae;Arthrobacter;Arthrobacter_sp._C0803	99.76	0	0.00211224	0	0.00204417	0
OTU2641	AY454678.1.1067	Archaea;Nanoarchaeota;Nanoarchaeia;Woesearchaeales;uncultured_crenarchaeote	83.59	0.0011083	0	0	0	0
OTU2644	AACY020170993.10.1544	Bacteria;Bdellovibrionota;Oligoflexia;0319-6G20;marine_metagenome	93.22	0.0011083	0	0	0	0
OTU2645	FJ479363.1.1442	Bacteria;Proteobacteria;Alphaproteobacteria;Caulobacterales;Caulobacteraceae;Phenylobacterium;uncultured_bacterium	100	0	0	0	0	0.00098733
OTU2647	FPLL01003665.15.1315	Bacteria;Bacteroidota;Bacteroidia;Cytophagales;Microscillaceae;Hassallia;metagenome	99.52	0	0	0.04437542	0	0

OTU2649	ET512011.1.1361	Bacteria;Proteobacteria;Gammaproteobacteria;Pseudomonadales;Pseudomonadaceae;Pseudomonas;uncultured_bacterium	94.85	0.3247329	0.2080561	0.00138673	0.01124296	0.00098733
OTU265	LCQK01000003.64973.66448	Bacteria;Patescibacteria;Parcubacteria;Candidatus_Jorgensenbacteria;Candidatus_Jorgensenbacteria_bacterium_GW2011_GWB1_50_10	84.49	0	0	0.0041602	0.00102209	0
OTU2651	MFTT01000025.14894.16462	Bacteria;Patescibacteria;Parcubacteria;Candidatus_Nomurabacteria;Candidatus_Nomurabacteria_bacterium_RIFCSPHIGHO2_01_FTLL_42_16	78.19	0	0	0	0	0.00098733
OTU2655	DQ532236.1.1432	Bacteria;Proteobacteria;Alphaproteobacteria;Rhodobacterales;Rhodobacteraceae;Rubellimicrobium;uncultured_bacterium	100	0.0011083	0	0	0.00102209	0
OTU2657	AB769168.1.1477	Bacteria;Firmicutes;Bacilli;Paenibacillales;Paenibacillaceae;Paenibacillus;Paenibacillus_shirakamiensis	99.06	0.0011083	0	0.14976703	0.12469465	0
OTU266	KX163953.1.1419	Bacteria;Nitrospirota;Nitrospiria;Nitrospirales;Nitrospiraceae;Nitrospira;uncultured_bacterium	99.3	0	0.00211224	0	0	0
OTU2667	AM997330.1.1642	Bacteria;Patescibacteria;Parcubacteria;Candidatus_Portnoybacteria;uncultured_deep-sea_bacterium	94.35	0	0	0	0.00102209	0
OTU2668	EF032777.1.1476	Bacteria;Verrucomicrobiota;Omnitrophia;Omnitrophales;Omnitrophaceae;Candidatus_Omnitrophus;uncultured_bacterium	82.86	0.0011083	0	0	0	0
OTU267	MHEO01000007.6346.7903	Bacteria;Nitrospirota;Leptospirillia;Leptospirillales;Leptospirillaceae;Leptospirillum;Nitrospirae_bacterium_RIFCSPHIGHO2_01_FTLL_66_17	98.83	0	0.00422449	0	0	0.00098733
OTU2674	GT455302.1.1426	Bacteria;Proteobacteria;Alphaproteobacteria;Rhodobacterales;Rhodobacteraceae;Rhodobacter;uncultured_bacterium	100	0	0	0	0.00204417	0
OTU2675	AM991220.1.1428	Bacteria;Proteobacteria;Alphaproteobacteria;Paracaedibacterales;Paracaedibacteraceae;Candidatus_Finniella;uncultured_bacterium	100	0	0	0	0.00102209	0
OTU2677	ET512011.1.1361	Bacteria;Proteobacteria;Gammaproteobacteria;Pseudomonadales;Pseudomonadaceae;Pseudomonas;uncultured_bacterium	95.32	0.00221661	0.12462244	0	0.00204417	0
OTU268	AF351227.1.1410	Bacteria;Proteobacteria;Gammaproteobacteria;Tenderiales;Tenderiaceae;Candidatus_Tenderia;uncultured_beta_proteobacterium	100	0	0.00105612	0.00138673	0.00408835	0.00098733
OTU2681	HM278749.1.1359	Bacteria;Bacteroidota;Bacteroidia;Flavobacteriales;Flavobacteriaceae;Pricia;uncultured_bacterium	92.64	0.00332491	0	0	0.00102209	0
OTU2684	LQBF01000029.1.1315	Bacteria;Desulfobacterota;Dissulfuribacteria;Dissulfuribacterales;Dissulfuribacteraceae;uncultured;delta_proteobacterium_ML8_D	83.45	0	0	0	0	0.00098733
OTU2687	CP017150.2637311.2638787	Bacteria;Actinobacteriota;Actinobacteria;Micrococcales;Brevibacteriaceae;Brevibacterium;Brevibacterium_aurantiacum	100	0.0011083	0.00105612	0	0.00102209	0
OTU2689	FPLS01053249.17.1497	Bacteria;Proteobacteria;Alphaproteobacteria;Rickettsiales;SM2D12;metagenome	90.05	0.0011083	0	0	0	0
OTU269	JN656876.1.1485	Bacteria;Bacteroidota;Bacteroidia;Sphingobacteriales;env.OPS_17;uncultured_Bacteroidetes_bacterium	100	0	0	0	0.00102209	0.00197467
OTU2693	AB637285.1.1482	Bacteria;Actinobacteriota;Actinobacteria;Micrococcales;Cellulomonadaceae;Cellulomonas;uncultured_bacterium	100	0.0011083	0	0	0	0
OTU2694	AB468957.1.1487	Bacteria;Proteobacteria;Gammaproteobacteria;Thioprofundaceae;Thioprofundum;Thioprofundum_lithotrophicum	92.04	0	0.00105612	0	0	0
OTU2697	FQ659000.2.1372	Bacteria;Myxococcota;Polyangia;Polyangiales;Sandaracinaceae;uncultured;uncultured_soil_bacterium	82.94	0	0	0.00138673	0	0
OTU2698	FJ373023.1.1444	Bacteria;Proteobacteria;Gammaproteobacteria;Pseudomonadales;Pseudomonadaceae;Pseudomonas;Pseudomonas_sp._W1-1	99.06	0	0	0.00138673	0.00102209	0.10663191
OTU27	HM129538.1.1438	Bacteria;Bacteroidota;Bacteroidia;Flavobacteriales;Flavobacteriaceae;Flavobacterium;uncultured_bacterium	100	0.01884116	0.0190102	0.16779454	0.0163534	0.01579732
OTU270	KX363701.1.1459	Bacteria;Proteobacteria;Gammaproteobacteria;Pseudomonadales;Pseudomonadaceae;Pseudomonas;uncultured_Pseudomonas_sp.	99.3	0.00997473	0.00211224	1.39505214	0.00306626	0.00197467
OTU2700	HM187000.1.1345	Bacteria;Chloroflexi;Dehalococcoidia;SAR202_clade;uncultured_bacterium	99.25	0.0011083	0	0	0	0
OTU2702	HM270655.1.1327	Bacteria;Cyanobacteria;Sericytochromatia;uncultured_bacterium	95.31	0	0.00105612	0	0	0
OTU2706	MF942642.1.1390	Bacteria;Verrucomicrobiota;Omnitrophia;Omnitrophales;Omnitrophaceae;Candidatus_Omnitrophus;uncultured_bacterium	89.74	0	0	0	0	0.00098733
OTU2708	MHGG01000050.25339.26910	Bacteria;Verrucomicrobiota;Omnitrophia;Omnitrophales;Omnitrophaceae;Candidatus_Omnitrophus;Omnitrophica_WOR_2_bacterium_GWF2_63_9	91.75	0	0	0	0	0.00098733
OTU271	FPLK01001355.1.1471	Bacteria;Proteobacteria;Gammaproteobacteria;Pseudomonadales;Pseudohongiellaceae;Pseudohongiella;metagenome	98.36	0	0.00211224	0.00138673	0.00204417	0.00394933
OTU2710	HM187047.1.1427	Bacteria;Verrucomicrobiota;Omnitrophia;Omnitrophales;Omnitrophaceae;Candidatus_Omnitrophus;uncultured_bacterium	94.64	0	0	0	0	0.00098733
OTU2712	MGFA01000012.36590.38091	Bacteria;Patescibacteria;ABY1;Candidatus_Thrbacteria;Candidatus_Thrbacteria_bacterium_RIFOXYB12_FTLL_58_10	95.05	0	0	0	0.00102209	0
OTU2715	AB506399.1.1495	Bacteria;Firmicutes;Clostridia;Peptostreptococcales-Tissierellales;Peptostreptococcaceae;Romboutsia;uncultured_bacterium	100	0	0	0	0.00102209	0
OTU2716	MHJF01000011.24156.25754	Bacteria;Patescibacteria;Parcubacteria;Candidatus_Colwellbacteria;Candidatus_Harrisonbacteria_bacterium_RIFCSPHIGHO2_02_FTLL_40_20	85.28	0	0.00105612	0.00138673	0	0
OTU2719	DQ395977.1.1441	Bacteria;Proteobacteria;Alphaproteobacteria;Reyranellales;Reyranellaceae;Reyranella;uncultured_organism	99.75	0	0	0	0.00102209	0
OTU272	FM873903.1.1478	Bacteria;Firmicutes;Clostridia;Peptostreptococcales-Tissierellales;Family_XI;Anaerococcus;uncultured_bacterium	99.75	0.0011083	0.00211224	0.00277346	0.00102209	0
OTU2720	AB517708.1.1442	Bacteria;Bacteroidota;Bacteroidia;Flavobacteriales;Weeksellaceae;Chryseobacterium;Chryseobacterium_indologenes	99.76	0.0011083	0	0	0	0
OTU2721	MFTV01000001.3080.4539	Bacteria;Patescibacteria;Parcubacteria;TBA9983;Candidatus_Nomurabacteria_bacterium_RIFCSPHIGHO2_02_40_30	94.48	0	0.00105612	0	0	0
OTU2722	HM129278.1.1438	Bacteria;Bacteroidota;Bacteroidia;Chitinophagales;Chitinophagaceae;Rurimicrobium;uncultured_bacterium	99.76	0	0	0	0.00102209	0
OTU2723	FJ676386.1.1395	Bacteria;Bacteroidota;Bacteroidia;Bacteroidales;Bacteroidaceae;Bacteroides;uncultured_bacterium	100	0.0011083	0	0	0	0
OTU2727	ET924256.1.1397	Bacteria;Patescibacteria;Parcubacteria;GW2011-GWA2-46-7;uncultured_bacterium	88.5	0	0	0	0	0.00098733
OTU273	CP009281.5845090.5846653	Bacteria;Firmicutes;Bacilli;Paenibacillales;Paenibacillaceae;Paenibacillus;Paenibacillus_sp._FSL_R5-0345	100	0.0011083	0	0.84867983	0.39248153	0.002962
OTU2735	LCOT01000026.4898.6395	Bacteria;Patescibacteria;Parcubacteria;Candidatus_Jorgensenbacteria;Parcubacteria_group_bacterium_GW2011_GWA2_47_8b	90.86	0	0	0	0	0.00098733
OTU2740	HE574354.1.1344	Bacteria;Bacteroidota;Bacteroidia;Flavobacteriales;Flavobacteriaceae;Flavobacterium;uncultured_bacterium	98.58	0	0	0.00138673	0	0
OTU2741	AFYF01000036.1.1391	Bacteria;Actinobacteriota;Actinobacteria;Micrococcales;Intrasporangiaceae;Serinicoccus;Serinicoccus_profundi_MCCC_1A05965	99.75	0	0	0.00138673	0.00102209	0
OTU2742	ET979061.1.1453	Bacteria;Proteobacteria;Alphaproteobacteria;Sphingomonadales;Sphingomonadaceae;Sphingomonas;uncultured_Alphaproteobacteria_bacterium	100	0	0.00105612	0	0.00102209	0
OTU2743	AY493985.1.1418	Bacteria;Patescibacteria;ABY1;Candidatus_Thrbacteria;uncultured_soil_bacterium	97.52	0	0.00105612	0	0.00102209	0
OTU2749	EF019262.1.1417	Bacteria;Verrucomicrobiota;Omnitrophia;Omnitrophales;Omnitrophaceae;Candidatus_Omnitrophus;uncultured_bacterium	97.9	0	0.00105612	0	0	0
OTU275	JX403027.1.1498	Bacteria;Campylobacterota;Campylobacteria;Campylobacterales;Sulfurovaceae;Sulfurovum;uncultured_bacterium	100	0.0011083	0	0.00277346	0.00102209	0
OTU2750	AM991202.1.1450	Bacteria;Elusimicrobiota;Elusimicrobia;Lineage_IV;uncultured_bacterium	95.41	0.0011083	0	0	0	0
OTU2754	FJ444659.1.1438	Bacteria;Proteobacteria;Gammaproteobacteria;Burkholderiales;Nitrosomonadaceae;MND1;uncultured_bacterium	100	0.0011083	0	0	0.00102209	0
OTU2757	MG214551.1.1436	Bacteria;Bacteroidota;Bacteroidia;Flavobacteriales;Flavobacteriaceae;Marixanthomonas;Marinirhabdus_sp.	99.05	0	0.00105612	0	0	0
OTU2758	ET800706.1.1447	Bacteria;Proteobacteria;Alphaproteobacteria;Rickettsiales;Rickettsiaceae;uncultured;uncultured_bacterium	92.29	0.0011083	0	0	0	0
OTU2759	JF809778.1.1414	Bacteria;Myxococcota;bacteriap25;uncultured_bacterium	94.86	0.0011083	0	0	0	0
OTU276	ET801889.1.1478	Bacteria;Bacteroidota;Bacteroidia;Flavobacteriales;Flavobacteriaceae;Flavobacterium;uncultured_bacterium	100	0.00221661	0.00105612	0.02080098	0.00204417	0.00493666
OTU2760	KC886756.1.1485	Bacteria;Bacteroidota;Bacteroidia;Flavobacteriales;Crocinitomicaceae;Fluviicola;uncultured_Cryomorphaceae_bacterium	99.05	0	0	0.0041602	0.00102209	0
OTU2766	ET134909.1.1431	Bacteria;Verrucomicrobiota;Omnitrophia;Omnitrophales;Omnitrophaceae;Candidatus_Omnitrophus;uncultured_bacterium	91.61	0	0	0.00138673	0	0
OTU2767	KY194666.1.1289	Bacteria;Chloroflexi;Dehalococcoidia;SAR202_clade;uncultured_bacterium	88.83	0	0.00105612	0	0	0
OTU277	FJ484483.1.1366	Bacteria;Proteobacteria;Gammaproteobacteria;Beggiatoales;Beggiatoaceae;uncultured;uncultured_proteobacterium	97.42	0	0.00211224	0.00138673	0.00102209	0.002962
OTU2770	AB681203.1.1455	Bacteria;Bacteroidota;Bacteroidia;Flavobacteriales;Flavobacteriaceae;Salegentibacter;Salegentibacter_mishustinae	99.05	0	0.00105612	0	0	0
OTU2773	AY168740.1.1424	Bacteria;Proteobacteria;Alphaproteobacteria;Rhodobacterales;Rhodobacteraceae;uncultured;uncultured_bacterium	100	0	0	0.00138673	0	0
OTU278	AB920569.1.1431	Bacteria;Actinobacteriota;Actinobacteria;Corynebacteriales;Nocardiaceae;Rhodococcus;Rhodococcus_kyotonensis	100	0.0011083	0	0.00138673	0.00204417	0.00098733
OTU2784	HM124374.1.1400	Bacteria;Proteobacteria;Gammaproteobacteria;Burkholderiales;Rhodocyclaceae;Ferribacterium;Ferribacterium_sp._24-19	98.13	0	0	0	0.00102209	0
OTU279	DQ453810.1.1496	Bacteria;Proteobacteria;Gammaproteobacteria;Pseudomonadales;Pseudomonadaceae;Pseudomonas;Pseudomonas_sp._m1(2006)	99.77	0.00554152	0.00316837	1.3756379	0.00511044	0.00197467
OTU2790	AY913248.1.1463	Bacteria;Acidobacteriota;Acidobacteriae;Subgroup_2;uncultured_forest_soil_bacterium	96.63	0.0011083	0	0	0	0
OTU2793	KC424704.1.1396	Bacteria;Patescibacteria;WWE3;uncultured_bacterium	90.32	0	0	0	0	0.00098733
OTU2797	KM251061.1.1371	Bacteria;Patescibacteria;Berkelbacteria;uncultured_bacterium	93.8	0	0	0.00138673	0	0
OTU28	ET802021.1.1480	Bacteria;Bacteroidota;Bacteroidia;Cytophagales;Spirosomaceae;Pseudarcicella;uncultured_bacterium	100	0.00886643	0.01689796	0.20662303	0.01226505	0.00789866

OTU280	JF703363.1.1461	Bacteria;Nitrospirota;Leptospirillia;Leptospirillales;Leptospirillaceae;Leptospirillum;uncultured_bacterium	87.85	0	0.00211224	0.00138673	0.00102209	0.00197467
OTU2801	HM186750.1.1348	Bacteria;Chloroflexi;Dehalococcoidia;SAR202_clade;uncultured_bacterium	98.76	0	0	0	0.00102209	0
OTU2804	FM873335.1.1476	Bacteria;Proteobacteria;Gammaproteobacteria;Burkholderiales;Comamonadaceae;Leptothrix;uncultured_bacterium	99.53	0.0011083	0	0.00138673	0	0.00098733
OTU2807	AM936280.1.1283	Bacteria;Proteobacteria;Alphaproteobacteria;Caulobacterales;Parvularculaceae;Amphiplicatus;uncultured_Rhizobiales_bacterium	100	0	0.00211224	0	0	0
OTU281	HQ120546.1.1496	Bacteria;Bacteroidota;Bacteroidia;Flavobacteriales;Flavobacteriaceae;Flavobacterium;uncultured_bacterium	99.53	0.00443321	0.00633673	0.00693366	0.00613252	0.62399415
OTU2811	FN668074.1.1480	Bacteria;Bacteroidota;Bacteroidia;Sphingobacteriales;NS11-12_marine_group;uncultured_Sphingobacterium_sp.	99.53	0.0011083	0	0	0	0
OTU2813	FJ478641.1.1456	Bacteria;Proteobacteria;Alphaproteobacteria;Dongiales;Dongiaceae;Dongia;uncultured_bacterium	99.5	0	0.00105612	0	0	0.00197467
OTU2814	JN417588.1.1505	Bacteria;Gemmatimonadota;Gemmatimonadetes;Gemmatimonadales;Gemmatimonadaceae;uncultured;uncultured_soil_bacterium	98.02	0	0	0	0	0.00197467
OTU2815	JQ278816.1.1534	Bacteria;Verrucomicrobiota;Omnitrophia;Omnitrophales;Omnitrophaceae;Candidatus_Omnitrophus;uncultured_bacterium	91.06	0	0.00105612	0	0	0
OTU282	KY356865.1.937	Archaea;Nanoarchaeota;Nanoarchaeia;Woesearchaeales;GW2011_GWC1_47_15;uncultured_archaeon	88.89	0	0.00211224	0	0	0
OTU2821	GT127062.1.1228	Bacteria;Verrucomicrobiota;Omnitrophia;Omnitrophales;Omnitrophaceae;Candidatus_Omnitrophus;uncultured_Verrucomicrobia_bacterium	85.05	0.0011083	0.00105612	0	0	0
OTU2823	ET801192.1.1495	Bacteria;Proteobacteria;Gammaproteobacteria;Burkholderiales;Comamonadaceae;Polaromonas;uncultured_bacterium	100	0.0011083	0	0	0	0.00789866
OTU2826	JX080239.1.1485	Bacteria;Acidobacteriota;Subgroup_22;uncultured_Acidobacteriales_bacterium	98.13	0.0011083	0	0	0	0.00098733
OTU2829	JF830233.1.1460	Bacteria;Proteobacteria;Alphaproteobacteria;Acetobacterales;Acetobacteraceae;Roseomonas;bacterium_enrichment_culture_clone_B197(2011)	99.75	0.0011083	0	0	0	0
OTU283	GQ500769.1.1502	Bacteria;Nitrospirota;Nitrospiria;Nitrospirales;Nitrospiraceae;Nitrospira;uncultured_bacterium	99.52	0	0.00211224	0	0	0.00098733
OTU2833	ET981270.1.1410	Bacteria;Desulfobacterota;Desulfomonilia;Desulfomonilales;Desulfomonilaceae;Desulfomonile;uncultured_bacterium	80	0	0	0	0	0.00098733
OTU2836	AB426197.1.1426	Bacteria;Latescibacterota;uncultured_bacterium	95.1	0	0.00105612	0	0	0
OTU2837	MF942639.1.1600	Bacteria;Verrucomicrobiota;Omnitrophia;Omnitrophales;uncultured_bacterium	93.22	0.00221661	0	0	0	0
OTU284	DQ520173.1.1439	Bacteria;Actinobacteriota;Actinobacteria;Frankiales;Sporichthyaceae;hgcI_clade;uncultured_bacterium	98.28	0.0011083	0.00316837	0.00138673	0	0
OTU2844	EF516273.1.1477	Bacteria;Latescibacterota;uncultured_bacterium	98.13	0	0	0	0	0.00098733
OTU285	AP014630.20654.22147	Bacteria;Proteobacteria;Gammaproteobacteria;Pseudomonadales;Moraxellaceae;Acinetobacter;Acinetobacter_guillouiae	100	0.00332491	0.00316837	0.00693366	0.00306626	0.00098733
OTU2851	JF266266.1.1362	Bacteria;Acidobacteriota;Acidobacteriae;Subgroup_2;uncultured_bacterium	96.88	0	0	0.00138673	0	0.00098733
OTU2856	MFVN01000036.3183.4707	Archaea;Nanoarchaeota;Nanoarchaeia;Woesearchaeales;Candidatus_Nomurabacteria_bacterium_RIFCSPLOWO2_02_FTLL_42_17	80.87	0	0.00316837	0	0	0
OTU286	HM128757.1.1437	Bacteria;Actinobacteriota;Actinobacteria;Frankiales;Sporichthyaceae;Candidatus_Planktophila;uncultured_bacterium	100	0	0.00105612	0.00138673	0.00102209	0
OTU2860	JN672018.1.1292	Bacteria;Elusimicrobiota;Elusimicrobia;Lineage_IV;uncultured_bacterium	95.89	0	0.00105612	0	0	0
OTU2861	AB360345.1.1443	Bacteria;Actinobacteriota;Acidimicrobiia;Microtrichales;Ilumatobacteraceae;Ilumatobacter;Ilumatobacter_nonamiensis_YM16-303	100	0.00332491	0.00211224	0	0.00613252	0
OTU2862	LN570914.1.1386	Bacteria;Bdellovibrionota;Oligoflexia;0319-6G20;uncultured_bacterium	93.93	0.0011083	0	0	0	0
OTU2869	AY584572.1.1405	Bacteria;Proteobacteria;Alphaproteobacteria;Sphingomonadales;Sphingomonadaceae;Sphingorhabdus;Sphingomonas_sp._HTCC503	99.5	0	0	0.0041602	0	0
OTU2870	DQ450182.1.1483	Bacteria;Bacteroidota;Bacteroidia;Flavobacteriales;Crocinitomicaceae;Fluviicola;uncultured_proteobacterium	99.76	0	0	0	0	0.00098733
OTU2871	LCOT01000026.4898.6395	Bacteria;Patescibacteria;Parcubacteria;Candidatus_Jorgensenbacteria;Parcubacteria_group_bacterium_GW2011_GWA2_47_8b	86.11	0	0.00105612	0	0	0
OTU2873	KX123607.1.1467	Bacteria;Patescibacteria;Parcubacteria;Candidatus_Yanofskybacteria;Candidatus_Yanofskybacteria_bacterium_GW2011_GWF1_44_227	91.07	0	0	0	0	0.00098733
OTU2875	LN560171.1.1322	Bacteria;Proteobacteria;Alphaproteobacteria;Rhodospirillales;uncultured;uncultured_bacterium	99	0	0.00105612	0	0	0
OTU2876	DQ515962.1.1424	Bacteria;Bacteroidota;Bacteroidia;Flavobacteriales;Flavobacteriaceae;Flavobacterium;Flavobacterium_glaciei	98.58	0	0	0.00138673	0	0
OTU288	JQ624268.1.1492	Bacteria;Bacteroidota;Bacteroidia;Sphingobacteriales;Sphingobacteriaceae;Pedobacter;uncultured_Pedobacter_sp.	99.76	0	0.00105612	0	0.00102209	0
OTU2880	JQ278807.1.1512	Bacteria;Acetothermia;Acetothermiia;uncultured_bacterium	97.2	0	0	0	0.00102209	0
OTU2882	FPLK01002744.11.1480	Bacteria;Proteobacteria;Alphaproteobacteria;Rhizobiales;Rhizobiales_Incertae_Sedis;Nordella;metagenome	99.75	0.00221661	0	0.00138673	0	0.00098733
OTU2884	CP002876.632833.634358	Bacteria;Proteobacteria;Gammaproteobacteria;Burkholderiales;Nitrosomonadaceae;Nitrosomonas;Nitrosomonas_sp._Is79A3	96.96	0.0011083	0	0	0	0
OTU2885	JQ278979.1.1316	Bacteria;Proteobacteria;Gammaproteobacteria;Burkholderiales;Gallionellaceae;Gallionella;uncultured_beta_proteobacterium	100	0	0.00211224	0	0	0.00098733
OTU2886	JF266340.1.1380	Bacteria;Nitrospirota;Leptospirillia;Leptospirillales;Leptospirillaceae;Leptospirillum;uncultured_bacterium	100	0	0	0.00138673	0.00102209	0
OTU2887	KX097513.1.1442	Bacteria;Patescibacteria;ABY1;Candidatus_Kerfeldbacteria;uncultured_bacterium	81.28	0	0	0	0	0.00098733
OTU2888	GQ448468.1.1366	Bacteria;Fusobacteriota;Fusobacteriia;Fusobacteriales;Fusobacteriaceae;Fusobacterium;uncultured_bacterium	99.75	0	0.00105612	0	0	0
OTU289	AZHS01000013.11871.13367	Bacteria;Actinobacteriota;Actinobacteria;Frankiales;Sporichthyaceae;hgcI_clade;actinobacterium_SCGC_AAA041-L13	100	0.0011083	0.00105612	0.00138673	0	0.00098733
OTU2891	LJHW01000064.3654.5190	Bacteria;Proteobacteria;Gammaproteobacteria;Burkholderiales;Comamonadaceae;Leptothrix;beta_proteobacterium_AAP65	98.59	0	0.00105612	0	0	0
OTU2892	KF697406.1.1227	Bacteria;Patescibacteria;Saccharimonadia;Saccharimonadales;uncultured_bacterium	98.01	0	0	0	0.00102209	0
OTU2893	AB630531.1.1467	Bacteria;Firmicutes;Clostridia;Clostridiales;Clostridiaceae;Clostridium_sensu_stricto_13;uncultured_bacterium	100	0	0	0.08875083	0.00204417	0
OTU29	AB552875.1.1385	Bacteria;Bacteroidota;Bacteroidia;Cytophagales;Spirosomaceae;Emticicia;Emticicia_sp._C2b	99.53	0.00886643	0.0095051	0.00832039	0.00715461	0.00493666
OTU290	AB681044.1.1446	Bacteria;Bacteroidota;Bacteroidia;Cytophagales;Cytophagaceae;Cytophaga;Cytophaga_aurantiaca	100	0.0011083	0.00105612	0.00138673	0	0.00098733
OTU2900	JQ712434.1.1375	Bacteria;Campylobacterota;Campylobacteria;Campylobacterales;Sulfurimonadaceae;Thiovulum;uncultured_Epsilonproteobacteria_bacterium	94.53	0.0011083	0	0	0	0
OTU2902	AFQE01000146.4587.6115	Bacteria;Proteobacteria;Gammaproteobacteria;Burkholderiales;Neisseriaceae;Neisseria;Neisseria_macacae_ATCC_33926	99.77	0	0	0	0	0.00098733
OTU291	AACY023318740.240.1489	Bacteria;Actinobacteriota;Acidimicrobiia;Microtrichales;Ilumatobacteraceae;CL500-29_marine_group;marine_metagenome	100	0.00221661	0	0	0	0
OTU2911	KX123508.1.1526	Bacteria;Patescibacteria;Parcubacteria;Candidatus_Liptonbacteria;Parcubacteria_group_bacterium_GW2011_GWA1_60_11	81.98	0	0.00105612	0	0	0
OTU2914	AJ309940.1.1428	Bacteria;Proteobacteria;Gammaproteobacteria;Pseudomonadales;Moraxellaceae;Psychrobacter;Psychrobacter_submarinus	100	0.0011083	0	0	0	0
OTU2917	ET385751.1.1486	Bacteria;Zixibacteria;uncultured_bacterium	94.38	0.0011083	0	0	0	0
OTU2919	FPLK01001222.1.1318	Bacteria;Proteobacteria;Alphaproteobacteria;Rhodobacterales;Rhodobacteraceae;Tabrizicola;metagenome	100	0	0	0	0	0.00098733
OTU2926	KX172614.1.1403	Bacteria;Patescibacteria;Parcubacteria;Candidatus_Yanofskybacteria;uncultured_bacterium	89.04	0.0011083	0	0	0	0
OTU293	JQ278934.1.1504	Bacteria;Proteobacteria;Gammaproteobacteria;Legionellales;Legionellaceae;Legionella;uncultured_gamma_proteobacterium	97.43	0.00221661	0	0	0.00102209	0.00098733
OTU2932	FJ744803.1.1357	Bacteria;Firmicutes;Bacilli;Erysipelotrichales;Erysipelatoclostridiaceae;TCG-004;uncultured_bacterium	95.77	0	0.00105612	0	0	0
OTU294	AB490786.1.1488	Bacteria;Firmicutes;Bacilli;Bacillales;Planococcaceae;Paenisporosarcina;Sporosarcina_sp._MB6	100	0	0.10561223	0.02218771	0.70626233	0.08984726
OTU2944	KF287743.1.1503	Bacteria;Proteobacteria;Gammaproteobacteria;Burkholderiales;Rhodocyclaceae;Denitratisoma;uncultured_Denitratisoma_sp.	99.77	0	0	0	0.00102209	0
OTU295	AB681724.1.1464	Bacteria;Proteobacteria;Gammaproteobacteria;Pseudomonadales;Moraxellaceae;Acinetobacter;Acinetobacter_johnsonii	100	0.00332491	0.00211224	0.00138673	0.00102209	0.002962
OTU2951	AB630765.1.1482	Bacteria;MBNT15;uncultured_bacterium	98.6	0	0	0	0	0.00098733
OTU2953	KC358393.1.1301	Bacteria;Verrucomicrobiota;Omnitrophia;Omnitrophales;Omnitrophaceae;Candidatus_Omnitrophus;uncultured_bacterium	91.38	0	0	0	0.00102209	0
OTU2955	KY356865.1.937	Archaea;Nanoarchaeota;Nanoarchaeia;Woesearchaeales;GW2011_GWC1_47_15;uncultured_archaeon	87.44	0	0.00105612	0	0	0
OTU2956	CP000949.5254580.5256108	Bacteria;Proteobacteria;Gammaproteobacteria;Pseudomonadales;Pseudomonadaceae;Pseudomonas;Pseudomonas_putida_W619	99.53	0.0011083	0	0.14560683	0.00102209	0.00098733
OTU296	FPLP01003869.16.1484	Bacteria;Proteobacteria;Alphaproteobacteria;Micavibrionales;uncultured;metagenome	100	0.00443321	0	0	0.00102209	0
OTU2962	KP308745.1.1066	Archaea;Iainarchaeota;Iainarchaeia;Iainarchaeales;Candidatus_Iainarchaeum;uncultured_archaeon	86.42	0	0	0	0	0.00098733
OTU2963	HM187047.1.1427	Bacteria;Verrucomicrobiota;Omnitrophia;Omnitrophales;Omnitrophaceae;Candidatus_Omnitrophus;uncultured_bacterium	91.84	0	0.00105612	0	0	0.00098733

OTU2964	DQ375559.1.1484	Bacteria;Firmicutes;Bacilli;Bacillales;Planococcaceae;Chryseomicrobium;Planococcus_sp._KRPC10y	100	0	0	0	0.00102209	0
OTU2968	HM187468.1.1330	Archaea;Crenarchaeota;Nitrososphaeria;uncultured;uncultured_archaeon	98.7	0	0	0	0.00102209	0
OTU2969	JF703557.1.1428	Bacteria;Bacteroidota;Bacteroidia;Cytophagales;Microscillaceae;Chryseolinea;uncultured_Sphingobacteriales_bacterium	97.62	0.00221661	0	0.07488351	0	0
OTU2970	JZEL01000013.42573.44024	Bacteria;Patescibacteria;Parcubacteria;Candidatus_Kaiserbacteria;Parcubacteria_bacterium_OLB19	99.51	0	0.00105612	0	0	0
OTU2973	LCAH01000003.23730.25224	Bacteria;Patescibacteria;ABY1;Candidatus_Thrbacteria;Candidatus_Thrbacteria_bacterium_GW2011_GWC2_41_11	87.38	0	0	0.00138673	0	0
OTU2974	JQ906007.1.1394	Bacteria;Bdellovibrionota;Oligoflexia;Oligoflexales;Oligoflexaceae;uncultured;uncultured_bacterium	90.31	0.0011083	0	0	0	0
OTU2978	CP014525.1459320.1460808	Bacteria;Proteobacteria;Alphaproteobacteria;Rhodospirillales;Rhodospirillaceae;Haematospirillum;Haematospirillum_jordaniae	99.75	0	0	0	0.00102209	0
OTU298	AY212613.1.1510	Bacteria;Actinobacteriota;Actinobacteria;Micrococcales;Dermabacteraceae;Brachybacterium;uncultured_bacterium	100	0.0011083	0.00316837	0	0.00102209	0
OTU2980	DQ404607.1.1517	Bacteria;Elusimicrobiota;Lineage_IIc;uncultured_bacterium	95.5	0	0	0	0.00102209	0
OTU2982	KF851141.1.1500	Bacteria;Proteobacteria;Gammaproteobacteria;Pseudomonadales;Pseudomonadaceae;Pseudomonas;uncultured_Pseudomonas_sp.	99.3	0.00775812	0.00422449	0	0.00102209	0.22313715
OTU299	HQ114161.1.1483	Bacteria;Bacteroidota;Bacteroidia;Cytophagales;Microscillaceae;Hassallia;uncultured_bacterium	99.29	0.0011083	0.00105612	0.00138673	0	0.00098733
OTU2991	AB363738.1.1476	Bacteria;Firmicutes;Bacilli;Bacillales;Bacillaceae;Bacillus;Bacillus_simplex	100	0	0.0285153	0	0.03577306	0.005924
OTU2992	CP009428.12031.13594	Bacteria;Firmicutes;Bacilli;Paenibacillales;Paenibacillaceae;Paenibacillus;Paenibacillus_odorifer	100	0.0011083	0	0	0.09096577	0
OTU2994	EF018687.1.1337	Bacteria;Proteobacteria;Alphaproteobacteria;Rhodospirillales;uncultured;uncultured_bacterium	96.77	0	0	0	0.00102209	0
OTU2996	JQ278911.1.1519	Bacteria;Methylomirabilota;Methylomirabilia;Rokubacteriales;uncultured_bacterium	96.96	0	0	0	0.00102209	0
OTU2998	MHJF01000011.24156.25754	Bacteria;Patescibacteria;Parcubacteria;Candidatus_Colwellbacteria;Candidatus_Harrisonbacteria_bacterium_RIFCSPHIGHO2_02_FTLL_40_20	83.87	0.0011083	0	0	0	0
OTU2999	KJ650724.1.1502	Bacteria;Proteobacteria;Gammaproteobacteria;Xanthomonadales;Xanthomonadaceae;Thermomonas;uncultured_gamma_proteobacterium	100	0.0011083	0	0	0	0
OTU3	AM293565.1.1492	Bacteria;Proteobacteria;Gammaproteobacteria;Pseudomonadales;Pseudomonadaceae;Pseudomonas;Pseudomonas_reinekei	100	0.36463182	0.1700357	1.4588418	0.17579902	48.6883287
OTU30	JN674641.1.1390	Bacteria;Bacteroidota;Bacteroidia;Chitinophagales;Chitinophagaceae;Sediminibacterium;Sediminibacterium_goheungense	100	0.00886643	0.00528061	0.00693366	0.00613252	0.01086066
OTU300	CT920940.1.1306	Bacteria;Proteobacteria;Alphaproteobacteria;Caulobacterales;Caulobacteraceae;Phenylobacterium;uncultured_bacterium	100	0.0011083	0	0.00554693	0	0.00098733
OTU3003	FPLS01056201.18.1520	Bacteria;Bacteroidota;Bacteroidia;Sphingobacteriales;KD3-93;metagenome	99.29	0	0.00105612	0	0	0
OTU3007	AB008514.1.1214	Bacteria;Proteobacteria;Alphaproteobacteria;Caulobacterales;Caulobacteraceae;Brevundimonas;Brevundimonas_bacteroides	100	0	0	0.00138673	0	0
OTU301	ET801021.1.1499	Bacteria;Proteobacteria;Gammaproteobacteria;Burkholderiales;Comamonadaceae;Limnohabitans;uncultured_bacterium	100	0	0.00422449	0.02496117	0.00204417	0
OTU3010	JQ195940.1.1350	Bacteria;Bacteroidota;Bacteroidia;Flavobacteriales;Crocinitomicaceae;Fluviicola;uncultured_bacterium	99.76	0.0011083	0	0	0	0
OTU3018	FPLS01041741.9.1413	Bacteria;Patescibacteria;ABY1;Candidatus_Magasanikbacteria;metagenome	90.35	0.0011083	0	0	0	0
OTU302	AB045898.1.1373	Bacteria;Cyanobacteria;Cyanobacteriia;Cyanobacteriales;Phormidiaceae;Planktothrix_NIVA-CYA_15;Planktothrix_agardhii_CCAP_1459/15	100	0	0.00105612	0.00554693	0.00204417	0.00197467
OTU3020	HM187518.1.1319	Archaea;Crenarchaeota;Nitrososphaeria;Nitrosotaleales;Nitrosotaleaceae;uncultured_archaeon	99.74	0.0011083	0	0	0	0
OTU3026	FPLS01023405.20.1499	Bacteria;Chloroflexi;KD4-96;metagenome	99.75	0	0.00105612	0	0	0
OTU3028	MHJF01000011.24156.25754	Bacteria;Patescibacteria;Parcubacteria;Candidatus_Colwellbacteria;Candidatus_Harrisonbacteria_bacterium_RIFCSPHIGHO2_02_FTLL_40_20	82.38	0	0.00105612	0	0	0
OTU303	FPLK01002673.23.1523	Bacteria;Bacteroidota;Bacteroidia;Chitinophagales;Chitinophagaceae;Sediminibacterium;metagenome	99.76	0	0	0.01664078	0	0
OTU3034	HM186652.1.1360	Bacteria;Actinobacteriota;MB-A2-108;uncultured_bacterium	99.51	0.0011083	0	0	0	0
OTU3035	GQ402722.1.1440	Bacteria;Elusimicrobiota;Elusimicrobia;Lineage_IV;uncultured_bacterium	96.62	0	0	0.00138673	0	0
OTU304	JQ675522.1.1363	Bacteria;Bdellovibrionota;Oligoflexia;0319-6G20;uncultured_bacterium	98.36	0.0011083	0	0.00138673	0.00204417	0.00098733
OTU3044	LCDF01000001.64365.66805	Bacteria;Patescibacteria;Parcubacteria;Candidatus_Giovannonibacteria;Candidatus_Giovannonibacteria_bacterium_GW2011_GWF2_42_19	85.4	0	0	0	0	0.00098733
OTU3045	ET134909.1.1431	Bacteria;Verrucomicrobiota;Omnitrophia;Omnitrophales;Omnitrophaceae;Candidatus_Omnitrophus;uncultured_bacterium	92.07	0	0.00105612	0	0	0
OTU3051	DQ906866.1.1489	Bacteria;Methylomirabilota;Methylomirabilia;Rokubacteriales;uncultured_bacterium	99.77	0	0.00105612	0	0	0
OTU3057	KC541017.1.1484	Bacteria;Proteobacteria;Alphaproteobacteria;Reyranellales;Reyranellaceae;Reyranella;uncultured_bacterium	99.5	0	0.00422449	0	0	0
OTU306	FJ390473.1.1459	Bacteria;Firmicutes;Bacilli;Bacillales;Bacillaceae;Bacillus;Bacillus_sp._HX11	100	0.00221661	0.00105612	0.00138673	0.87388465	0
OTU3061	GT305829.1.1490	Bacteria;Bacteroidota;Bacteroidia;Flavobacteriales;Crocinitomicaceae;Fluviicola;uncultured_bacterium	100	0	0.00105612	0	0	0
OTU3063	HQ120546.1.1496	Bacteria;Bacteroidota;Bacteroidia;Flavobacteriales;Flavobacteriaceae;Flavobacterium;uncultured_bacterium	99.76	0.0011083	0	0.00554693	0.00102209	0.30706041
OTU3067	KF836295.1.1480	Bacteria;Elusimicrobiota;Elusimicrobia;Lineage_IV;uncultured_bacterium	91.06	0	0.00105612	0	0	0
OTU307	HM128531.1.1448	Bacteria;Bacteroidota;Bacteroidia;Sphingobacteriales;NS11-12_marine_group;uncultured_bacterium	99.76	0	0.00105612	0.00138673	0.00204417	0.00098733
OTU3070	AM900775.1.1542	Bacteria;Firmicutes;Bacilli;Bacillales;Bacillaceae;Bacillus;Bacillus_sp._PA27	99.77	0	0	0.00138673	0.31275872	0.00098733
OTU3072	HM187450.1.1355	Bacteria;Verrucomicrobiota;Omnitrophia;Omnitrophales;Omnitrophaceae;Candidatus_Omnitrophus;uncultured_bacterium	96.28	0	0	0	0.00102209	0
OTU3077	HE574357.1.1362	Bacteria;Bacteroidota;Bacteroidia;Flavobacteriales;Flavobacteriaceae;Flavobacterium;uncultured_bacterium	99.76	0.0011083	0	0	0	0.00098733
OTU308	FJ936783.1.1449	Bacteria;Planctomycetota;Phycisphaerae;Phycisphaerales;Phycisphaeraceae;SM1A02;uncultured_bacterium	93.28	0.0011083	0.00211224	0	0	0
OTU3080	MF942642.1.1390	Bacteria;Verrucomicrobiota;Omnitrophia;Omnitrophales;Omnitrophaceae;Candidatus_Omnitrophus;uncultured_bacterium	92.77	0	0.00105612	0	0	0
OTU3081	FJ834325.1.1448	Bacteria;Proteobacteria;Alphaproteobacteria;Sphingomonadales;Sphingomonadaceae;Sphingomonas;Sphingomonas_rubra	99.75	0	0	0	0.00102209	0
OTU3084	JQ072423.1.1363	Bacteria;Proteobacteria;Alphaproteobacteria;Holosporales;Holosporaceae;uncultured;uncultured_bacterium	98.01	0.0011083	0.00211224	0	0	0
OTU3085	FN550143.1.1476	Bacteria;Actinobacteriota;Actinobacteria;Corynebacteriales;Mycobacteriaceae;Mycobacterium;Mycobacteriaceae_bacterium_MI-6.3_T19	99.51	0	0	0.00138673	0	0
OTU3089	DQ128511.1.1416	Bacteria;Elusimicrobiota;Lineage_IIa;uncultured_soil_bacterium	98.58	0.0011083	0.00105612	0	0	0
OTU309	CAVK010000090.144.1648	Bacteria;Proteobacteria;Alphaproteobacteria;Sphingomonadales;Sphingomonadaceae;Sphingobium;Sphingobium_japonicum_BiD32	100	0	0	0	0	0.00098733
OTU3092	AF534216.1.1462	Bacteria;Proteobacteria;Gammaproteobacteria;Pseudomonadales;Pseudomonadaceae;Pseudomonas;uncultured_bacterium	96.49	0.07647294	0.17214794	0.00138673	0.00102209	0
OTU3093	EF612978.1.1365	Bacteria;Proteobacteria;Gammaproteobacteria;Legionellales;Legionellaceae;Legionella;uncultured_bacterium	97.66	0	0	0	0.00102209	0
OTU3095	JQ278816.1.1534	Bacteria;Verrucomicrobiota;Omnitrophia;Omnitrophales;Omnitrophaceae;Candidatus_Omnitrophus;uncultured_bacterium	92.33	0	0	0	0	0.00098733
OTU31	AB680483.1.1462	Bacteria;Proteobacteria;Gammaproteobacteria;Pseudomonadales;Pseudomonadaceae;Pseudomonas;Pseudomonas_putida	99.77	0.02105776	0.02957143	10.5377746	0.02861844	0.02073398
OTU310	AY822000.1.1279	Archaea;Nanoarchaeota;Nanoarchaeia;Woesearchaeales;uncultured_euryarchaeote	81.87	0	0.00211224	0	0.00102209	0
OTU3108	AY126452.1.1520	Bacteria;Proteobacteria;Gammaproteobacteria;Burkholderiales;Rhodocyclaceae;Dechloromonas;Dechloromonas_sp._PC1	100	0.0011083	0	0	0.00102209	0
OTU3109	AB369333.1.1275	Bacteria;Proteobacteria;Gammaproteobacteria;Pseudomonadales;Pseudomonadaceae;Pseudomonas;Pseudomonas_sp._GmFRB018	99.53	0.0011083	0	0.04298868	0	0.05232862
OTU311	FPLK01001862.10.1479	Bacteria;Bacteroidota;Kapabacteria;Kapabacteriales;metagenome	99.52	0.0011083	0	0.00277346	0	0.002962
OTU3113	AJ863290.1.1477	Bacteria;Bdellovibrionota;Oligoflexia;0319-6G20;uncultured_bacterium	94.16	0.00221661	0	0	0	0
OTU3116	AB021379.1.1511	Bacteria;Proteobacteria;Gammaproteobacteria;Pseudomonadales;Pseudomonadaceae;Pseudomonas;Pseudomonas_oleovorans	100	0.0011083	0	0.00277346	0.00306626	0.00197467
OTU3118	DQ499288.1.1481	Bacteria;Bdellovibrionota;Bdellovibrionia;Bdellovibrionales;Bdellovibrionaceae;OM27_clade;uncultured_bacterium	87.47	0	0.00105612	0	0	0
OTU3119	AP014879.2384902.2386420	Bacteria;Proteobacteria;Gammaproteobacteria;Acidiferrobacterales;Acidiferrobacteraceae;Sulfurifustis;Sulfuricaulis_limicola	98.36	0.0011083	0	0	0	0
OTU312	HM187074.1.1485	Bacteria;Nitrospirota;Leptospirillia;Leptospirillales;Leptospirillaceae;Leptospirillum;uncultured_bacterium	99.53	0	0.00316837	0.00138673	0.00102209	0.00197467
OTU3121	FQ659415.1.1402	Bacteria;Patescibacteria;Parcubacteria;Candidatus_Nomurabacteria;uncultured_soil_bacterium	97.05	0	0	0.0041602	0	0
OTU3122	DQ676386.1.1467	Bacteria;Verrucomicrobiota;Omnitrophia;Omnitrophales;Omnitrophaceae;Candidatus_Omnitrophus;uncultured_Verrucomicrobia_bacterium	95.79	0	0	0	0.00102209	0

OTU3126	ET266808.1.1492	Bacteria;Proteobacteria;Gammaproteobacteria;Acidiferrobacterales;Acidiferrobacteraceae;Sulfurifustis;uncultured_Thiotrichaceae_bacterium	99.53	0.0011083	0	0	0	0
OTU3129	HM069083.1.1504	Bacteria;Nitrospirota;Nitrospiria;Nitrospirales;Nitrospiraceae;Nitrospira;uncultured_bacterium	99.76	0.00554152	0.04541326	0.0263479	0	0.00098733
OTU313	JQ278884.1.1533	Bacteria;Verrucomicrobiota;Omnitrophia;Omnitrophales;Omnitrophaceae;Candidatus_Omnitrophus;uncultured_bacterium	91.86	0.00221661	0.00211224	0.00138673	0	0
OTU3132	FJ602408.1.1407	Bacteria;Proteobacteria;Alphaproteobacteria;Rickettsiales;Mitochondria;uncultured_bacterium	97.51	0	0	0.00138673	0	0
OTU3133	HM187047.1.1427	Bacteria;Verrucomicrobiota;Omnitrophia;Omnitrophales;Omnitrophaceae;Candidatus_Omnitrophus;uncultured_bacterium	91.38	0	0	0.00138673	0	0
OTU3134	AJ252833.1.1512	Bacteria;Actinobacteriota;Actinobacteria;Pseudonocardiales;Pseudonocardiaceae;Pseudonocardia;Pseudonocardia_kongjuensis	99.51	0.0011083	0	0	0	0
OTU3139	AB795498.1.1475	Bacteria;Bacteroidota;Bacteroidia;Bacteroidales;Prolixibacteraceae;uncultured;uncultured_bacterium	97.87	0	0	0	0.00102209	0
OTU314	ET937856.1.1388	Bacteria;Bdellovibrionota;Bdellovibrionia;Bdellovibrionales;Bdellovibrionaceae;Bdellovibrio;uncultured_bacterium	99.75	0	0.00316837	0.00138673	0	0
OTU3146	HM186750.1.1348	Bacteria;Chloroflexi;Dehalococcoidia;SAR202_clade;uncultured_bacterium	98.76	0	0	0	0	0.00098733
OTU3147	JQ278816.1.1534	Bacteria;Verrucomicrobiota;Omnitrophia;Omnitrophales;Omnitrophaceae;Candidatus_Omnitrophus;uncultured_bacterium	91.86	0	0	0	0.00102209	0
OTU3148	ET134497.1.1336	Bacteria;Bdellovibrionota;Bdellovibrionia;Bdellovibrionales;Bdellovibrionaceae;OM27_clade;uncultured_bacterium	95.12	0	0.00105612	0	0.00102209	0
OTU3151	CP001619.4003859.4005353	Bacteria;Bacteroidota;Bacteroidia;Cytophagales;Spirosomaceae;Dyadobacter;Dyadobacter_fermentans_DSM_18053	99.05	0	0.00105612	0	0	0
OTU3153	GQ500851.1.1516	Bacteria;Acidobacteriota;Blastocatellia;11-24;uncultured_bacterium	94.34	0	0	0.00138673	0	0
OTU3157	KX123508.1.1526	Bacteria;Patescibacteria;Parcubacteria;Candidatus_Liptonbacteria;Parcubacteria_group_bacterium_GW2011_GWA1_60_11	86.42	0	0.00105612	0	0	0
OTU3158	HE603186.1.1453	Bacteria;Verrucomicrobiota;Omnitrophia;Omnitrophales;Omnitrophaceae;Candidatus_Omnitrophus;uncultured_Firmicutes_bacterium	97.67	0	0.00105612	0	0	0
OTU316	AB095126.1.1130	Archaea;Nanoarchaeota;Nanoarchaeia;Woesearchaeales;uncultured_archaeon	82.08	0.0011083	0	0	0	0
OTU3165	AM997563.1.1519	Bacteria;Patescibacteria;Parcubacteria;Candidatus_Terrybacteria;uncultured_deep-sea_bacterium	87.43	0	0	0	0	0.00098733
OTU3166	KC990421.1.1249	Bacteria;Patescibacteria;Parcubacteria;Candidatus_Azambacteria;uncultured_Parcubacteria_group_bacterium	91.73	0	0.00105612	0	0.00102209	0
OTU3167	ET134927.1.1286	Bacteria;Patescibacteria;Gracilibacteria;Candidatus_Peribacteria;uncultured_bacterium	88.09	0	0	0	0	0.00098733
OTU317	EF020104.1.1375	Bacteria;Planctomycetota;Pla4_lineage;uncultured_bacterium	85.07	0	0.00316837	0	0	0
OTU3171	AY660701.1.1475	Bacteria;Firmicutes;Bacilli;Bacillales;Planococcaceae;Psychrobacillus;Bacillus_sp._cryopeg_9	99.77	0	0	0.00277346	0.09198786	0.00098733
OTU3176	AB045094.1.1500	Bacteria;Firmicutes;Bacilli;Paenibacillales;Paenibacillaceae;Paenibacillus;Paenibacillus_pabuli	99.77	0	0	0	0.00306626	0.00098733
OTU3177	FM207922.1.1499	Bacteria;Proteobacteria;Gammaproteobacteria;Burkholderiales;Rhodocyclaceae;Sulfuritalea;uncultured_Rhodocyclaceae_bacterium	99.77	0	0	0	0.00102209	0
OTU3179	HQ118896.1.1480	Bacteria;Bdellovibrionota;Oligoflexia;0319-6G20;uncultured_bacterium	89.95	0	0.00105612	0.00138673	0	0
OTU318	KY356869.1.921	Archaea;Nanoarchaeota;Nanoarchaeia;Woesearchaeales;GW2011_GWC1_47_15;uncultured_archaeon	86.3	0	0.00316837	0	0	0
OTU3181	KY194666.1.1289	Bacteria;Chloroflexi;Dehalococcoidia;SAR202_clade;uncultured_bacterium	93.3	0	0.00105612	0	0	0
OTU3187	KC358519.1.1286	Bacteria;Bdellovibrionota;Oligoflexia;0319-6G20;uncultured_bacterium	93.46	0.0011083	0	0	0	0
OTU319	FPLS01054955.17.1552	Bacteria;Bdellovibrionota;Oligoflexia;0319-6G20;metagenome	93.22	0	0	0	0.00204417	0.00098733
OTU3197	JX222555.1.1393	Bacteria;Bacteroidota;Bacteroidia;Flavobacteriales;Flavobacteriaceae;Lutibacter;uncultured_bacterium	96.21	0.0011083	0	0	0	0
OTU3198	GQ132489.1.1397	Bacteria;Firmicutes;Clostridia;Oscillospirales;Oscillospiraceae;NK4A214_group;uncultured_bacterium	96.27	0	0	0	0	0.00098733
OTU32	AY863090.1.1355	Bacteria;Verrucomicrobiota;Verrucomicrobiae;uncultured;uncultured_bacterium	100	0.00997473	0.0095051	0.21771688	0.00408835	0.00394933
OTU320	KF836147.1.1531	Bacteria;Nitrospirota;Leptospirillia;Leptospirillales;Leptospirillaceae;Leptospirillum;uncultured_bacterium	91.59	0.0011083	0	0	0	0.00098733
OTU3205	AB637152.1.1508	Bacteria;Firmicutes;Bacilli;Bacillales;Bacillaceae;Bacillus;uncultured_bacterium	99.77	0	0	0.00138673	0	0
OTU3208	KY356876.1.911	Archaea;Nanoarchaeota;Nanoarchaeia;Woesearchaeales;GW2011_GWC1_47_15;uncultured_archaeon	98.19	0	0.00105612	0	0	0
OTU321	KF836147.1.1531	Bacteria;Nitrospirota;Leptospirillia;Leptospirillales;Leptospirillaceae;Leptospirillum;uncultured_bacterium	91.82	0	0	0	0.00204417	0.00098733
OTU3215	AY305310.1.1456	Bacteria;Firmicutes;Clostridia;Lachnospirales;Lachnospiraceae;Roseburia;Roseburia_faecis_M72/1	100	0	0.00105612	0	0	0
OTU3220	AB626633.1.1528	Bacteria;Firmicutes;Negativicutes;Veillonellales-Selenomonadales;Veillonellaceae;Dialister;Dialister_micraerophilus	100	0.0011083	0	0	0	0.00197467
OTU3223	HM237107.1.1268	Bacteria;Proteobacteria;Gammaproteobacteria;Burkholderiales;Oxalobacteraceae;Tndibacterium;uncultured_bacterium	99.77	0	0	0	0.00102209	0.01184799
OTU3229	LCOT01000026.4898.6395	Bacteria;Patescibacteria;Parcubacteria;Candidatus_Jorgensenbacteria;Parcubacteria_group_bacterium_GW2011_GWA2_47_8b	82.72	0.0011083	0	0	0	0
OTU323	CXWL01003141.114212.115751	Bacteria;Verrucomicrobiota;Verrucomicrobiae;Opitutales;Opitutaceae;Lacunisphaera;groundwater_metagenome	99.77	0.0011083	0.00105612	0.0041602	0	0
OTU3238	HM856568.1.1440	Bacteria;Actinobacteriota;Actinobacteria;Micrococcales;Microbacteriaceae;Rhodoluna;uncultured_Microbacteriaceae_bacterium	99.75	0	0	0.00138673	0	0
OTU3239	LT629741.1332352.1333885	Bacteria;Bacteroidota;Bacteroidia;Flavobacteriales;Flavobacteriaceae;Gramella;Gramella_sp._MAR_2010_147	100	0	0	0	0.00102209	0
OTU324	ET800953.1.1493	Bacteria;Bacteroidota;Bacteroidia;Chitinophagales;Chitinophagaceae;Sediminibacterium;uncultured_bacterium	100	0.00221661	0.00211224	0.00554693	0.00102209	0
OTU3241	HM129400.1.1417	Bacteria;Proteobacteria;Alphaproteobacteria;Rickettsiales;Mitochondria;uncultured_bacterium	96.52	0	0	0.00138673	0	0
OTU3244	ET803844.1.1507	Bacteria;Bdellovibrionota;Oligoflexia;0319-6G20;uncultured_bacterium	92.06	0	0	0	0	0.00098733
OTU3246	JX521044.1.1482	Bacteria;Bacteroidota;Bacteroidia;Flavobacteriales;Flavobacteriaceae;Flavobacterium;uncultured_bacterium	98.58	0	0	0.00138673	0	0
OTU325	KT514865.1.1450	Bacteria;Proteobacteria;Alphaproteobacteria;Rhizobiales;Rhizobiaceae;Allorhizobium-Neorhizobium-Pararhizobium-Rhizobium;uncultured_bacterium	100	0.00221661	0.00422449	0.0041602	0.00204417	0.06318928
OTU3250	JF265850.1.1359	Bacteria;Proteobacteria;Gammaproteobacteria;Gammaproteobacteria_Incertae_Sedis;Tnknown_Family;Acidibacter;uncultured_bacterium	100	0	0.00105612	0	0	0
OTU3251	FJ901639.1.1387	Bacteria;Verrucomicrobiota;Omnitrophia;Omnitrophales;Omnitrophaceae;Candidatus_Omnitrophus;uncultured_Omnitrophica_bacterium	96.26	0	0	0	0.00102209	0
OTU3256	JQ278884.1.1533	Bacteria;Verrucomicrobiota;Omnitrophia;Omnitrophales;Omnitrophaceae;Candidatus_Omnitrophus;uncultured_bacterium	92.09	0	0	0	0.00102209	0
OTU3259	DQ499327.1.1492	Bacteria;Proteobacteria;Gammaproteobacteria;Acidiferrobacterales;Acidiferrobacteraceae;Sulfurifustis;uncultured_bacterium	92.02	0	0.00105612	0	0	0
OTU326	KX123338.1.3317	Archaea;Thermoplasmatota;Thermoplasmata;uncultured;Candidatus_Amesbacteria_bacterium_GW2011_GWC1_47_15	86.83	0	0.00316837	0	0	0
OTU3267	AY736250.1.1248	Bacteria;Actinobacteriota;Actinobacteria;Corynebacteriales;Mycobacteriaceae;Mycobacterium;uncultured_actinobacterium	100	0	0	0	0.00102209	0
OTU3268	JN409122.1.1409	Bacteria;Proteobacteria;Alphaproteobacteria;Sphingomonadales;Sphingomonadaceae;uncultured;uncultured_Alphaproteobacteria_bacterium	100	0	0.00105612	0	0	0
OTU327	AY922093.1.1372	Bacteria;Patescibacteria;Parcubacteria;uncultured_Parcubacteria_group_bacterium	91.13	0	0.00105612	0	0	0.00098733
OTU3277	GQ182301.1.1421	Bacteria;FCPT426;uncultured_bacterium	82.44	0	0	0	0.00102209	0
OTU3279	ET132257.1.1353	Bacteria;Acidobacteriota;Acidobacteriae;Elev-16S-1166;uncultured_bacterium	97.26	0.0011083	0	0.00138673	0	0
OTU328	JF313067.1.1512	Bacteria;Actinobacteriota;Actinobacteria;Micrococcales;Micrococcaceae;Arthrobacter;Arthrobacter_sp._K11	100	0.0011083	0.00211224	0.05130907	0.00102209	0.58746285
OTU329	JN656834.1.1496	Bacteria;Proteobacteria;Gammaproteobacteria;Burkholderiales;Comamonadaceae;Rhodoferax;uncultured_beta_proteobacterium	100	0.00221661	0	0.00138673	0.00102209	0.00098733
OTU3292	FJ471648.1.1428	Bacteria;Fusobacteriota;Fusobacteriia;Fusobacteriales;Fusobacteriaceae;Fusobacterium;Fusobacterium_nucleatum	99.75	0.0011083	0	0	0	0
OTU3297	KC358393.1.1301	Bacteria;Verrucomicrobiota;Omnitrophia;Omnitrophales;Omnitrophaceae;Candidatus_Omnitrophus;uncultured_bacterium	84.62	0.0011083	0	0	0	0
OTU3298	MF942642.1.1390	Bacteria;Verrucomicrobiota;Omnitrophia;Omnitrophales;Omnitrophaceae;Candidatus_Omnitrophus;uncultured_bacterium	93.24	0.0011083	0	0.00138673	0	0
OTU33	AY838526.1.1522	Bacteria;Proteobacteria;Gammaproteobacteria;Xanthomonadales;Xanthomonadaceae;Stenotrophomonas;uncultured_bacterium	100	0.01551625	0.01372959	0.02218771	0.01839757	0.00888599
OTU330	KP686608.1.1447	Bacteria;Bacteroidota;Bacteroidia;Flavobacteriales;Crocinitomicaceae;Fluviicola;uncultured_bacterium	99.53	0	0.00105612	0.01664078	0.00102209	0.00098733
OTU3300	HM186310.1.1469	Bacteria;Gemmatimonadota;Gemmatimonadetes;Gemmatimonadales;Gemmatimonadaceae;uncultured;uncultured_bacterium	98.6	0	0.00105612	0	0	0
OTU3304	MG601270.1.1385	Bacteria;Verrucomicrobiota;Omnitrophia;Omnitrophales;Omnitrophaceae;Candidatus_Omnitrophus;uncultured_bacterium	88.65	0	0	0.00138673	0	0
OTU3306	JQ195940.1.1350	Bacteria;Bacteroidota;Bacteroidia;Flavobacteriales;Crocinitomicaceae;Fluviicola;uncultured_bacterium	100	0	0	0	0.00102209	0

OTU331	GT305831.1.1494	Bacteria;Bacteroidota;Bacteroidia;Chitinophagales;Chitinophagaceae;Sediminibacterium;uncultured_bacterium	99.05	0.00332491	0.00105612	0.00554693	0	0.00098733
OTU3323	JQ428022.1.1509	Bacteria;Myxococcota;Polyangia;Haliangiales;Haliangiaceae;Haliangium;uncultured_bacterium	98.83	0.0011083	0	0	0	0
OTU333	HM187000.1.1345	Bacteria;Chloroflexi;Dehalococcoidia;SAR202_clade;uncultured_bacterium	99.75	0	0.00211224	0	0.00102209	0.00098733
OTU3332	AB681338.1.1470	Bacteria;Proteobacteria;Gammaproteobacteria;Xanthomonadales;Xanthomonadaceae;Pseudoxanthomonas;Pseudoxanthomonas_mexicana	100	0.0011083	0	0	0	0
OTU3334	JF703568.1.1458	Bacteria;Bdellovibrionota;Bdellovibrionia;Bacteriovoracales;Bacteriovoracaceae;Bacteriovorax;uncultured_Bacteriovorax_sp.	97.9	0	0	0	0.00102209	0
OTU3335	HQ120546.1.1496	Bacteria;Bacteroidota;Bacteroidia;Flavobacteriales;Flavobacteriaceae;Flavobacterium;uncultured_bacterium	99.05	0	0	0.00138673	0	0.42159099
OTU3337	KC734149.1.1437	Bacteria;Proteobacteria;Gammaproteobacteria;Pseudomonadales;Moraxellaceae;Acinetobacter;bacterium_14W122	100	0	0	0	0	0.00197467
OTU3341	HM241048.1.1310	Bacteria;Proteobacteria;Alphaproteobacteria;Sphingomonadales;Sphingomonadaceae;Ellin6055;uncultured_bacterium	99.5	0.00332491	0	0	0	0
OTU3343	AF127023.1.1481	Bacteria;Firmicutes;Clostridia;Clostridiales;Clostridiaceae;Clostridium_sensu_stricto_5;Clostridium_algidicarnis	100	0	0	0.08181717	0.00102209	0
OTU3348	GT305740.1.1454	Bacteria;Proteobacteria;Alphaproteobacteria;Rhizobiales;Rhizobiales_Incertae_Sedis;uncultured;uncultured_bacterium	99.25	0	0.00105612	0.00138673	0	0
OTU335	AB240317.1.1489	Bacteria;Proteobacteria;Gammaproteobacteria;Burkholderiales;Comamonadaceae;Ideonella;uncultured_bacterium	99.3	0.0011083	0	0	0	0.00197467
OTU3358	JF809687.1.1323	Bacteria;Patescibacteria;Berkelbacteria;uncultured_bacterium	88.56	0	0	0	0.00102209	0
OTU336	FJ535179.1.1478	Bacteria;Bacteroidota;Bacteroidia;Flavobacteriales;Flavobacteriaceae;Flavobacterium;uncultured_Flavobacteriia_bacterium	99.53	0.0011083	0	0.56717329	0.00613252	0.00394933
OTU3360	ET800091.1.1317	Bacteria;Proteobacteria;Gammaproteobacteria;Burkholderiales;Comamonadaceae;Polaromonas;uncultured_bacterium	99.77	0.0011083	0.00105612	0	0.00102209	0
OTU3362	AM991246.1.1419	Bacteria;Proteobacteria;Alphaproteobacteria;Rickettsiales;Mitochondria;uncultured_bacterium	95.52	0	0	0.00277346	0	0
OTU3363	JQ278980.1.1483	Bacteria;Bacteroidota;Bacteroidia;Sphingobacteriales;env.OPS_17;uncultured_Bacteroidetes_bacterium	96.23	0	0	0	0	0.00098733
OTU3365	AF253509.1.1489	Bacteria;Actinobacteriota;Actinobacteria;Propionibacteriales;Nocardioidaceae;Nocardioides;Nocardioides_sp._C157	100	0	0	0	0	0.00098733
OTU3366	JQ675522.1.1363	Bacteria;Bdellovibrionota;Oligoflexia;0319-6G20;uncultured_bacterium	91.59	0	0.00211224	0	0	0
OTU338	JF231450.1.1376	Bacteria;Firmicutes;Bacilli;Lactobacillales;Aerococcaceae;Abiotrophia;uncultured_bacterium	100	0	0.00211224	0	0	0.00098733
OTU3381	EF111210.1.1218	Bacteria;Proteobacteria;Alphaproteobacteria;Caulobacterales;Caulobacteraceae;Brevundimonas;uncultured_Alphaproteobacteria_bacterium	100	0	0	0	0	0.00098733
OTU3384	GQ355039.1.1452	Bacteria;Campylobacterota;Campylobacteria;Campylobacterales;Sulfurovaceae;Sulfurovum;uncultured_bacterium	97.76	0	0.00105612	0	0	0
OTU3387	ASMR01000001.18974.20405	Archaea;Iainarchaeota;Iainarchaeia;Iainarchaeales;Candidatus_Iainarchaeum;Diapherotrites_archaeon_SCGC_AAA011-K09	81.1	0	0	0	0	0.00098733
OTU3391	DQ395891.1.1449	Bacteria;Proteobacteria;Alphaproteobacteria;Caulobacterales;Hyphomonadaceae;Henriciella;uncultured_organism	99	0	0	0.00138673	0	0
OTU3394	ATVO01000004.91274.92741	Bacteria;Proteobacteria;Alphaproteobacteria;Sphingomonadales;Sphingomonadaceae;Sandarakinorhabdus;Sandarakinorhabdus_limnophila_DSM_17366	100	0	0	0.00138673	0	0
OTU3396	KC588525.1.1446	Archaea;Crenarchaeota;Nitrososphaeria;uncultured;uncultured_archaeon	98.7	0	0.00105612	0	0	0
OTU3399	AM180484.1.1401	Bacteria;Proteobacteria;Alphaproteobacteria;Rhizobiales;Rhizobiaceae;Pseudochrobactrum;Pseudochrobactrum_saccharolyticum	100	0	0	0	0	0.00098733
OTU34	AY294222.1.1443	Bacteria;Bdellovibrionota;Bdellovibrionia;Bacteriovoracales;Bacteriovoracaceae;Peredibacter;Bacteriovorax_sp._EPC3	96.73	0.00664982	0.00211224	0.00970712	0.01226505	0.005924
OTU340	AB858570.1.1334	Bacteria;Patescibacteria;Parcubacteria;TBA9983;uncultured_bacterium	95.09	0	0.00105612	0	0	0
OTU3402	MF942642.1.1390	Bacteria;Verrucomicrobiota;Omnitrophia;Omnitrophales;Omnitrophaceae;Candidatus_Omnitrophus;uncultured_bacterium	90.21	0	0	0.00138673	0.00102209	0
OTU3403	JN038912.1.1518	Bacteria;Acidobacteriota;Subgroup_22;uncultured_proteobacterium	97.66	0.0011083	0	0	0	0
OTU3406	KP866212.1.1451	Bacteria;Bacteroidota;Bacteroidia;Flavobacteriales;Flavobacteriaceae;Flavobacterium;Flavobacterium_sp._HMF3121	100	0.0011083	0	0	0	0
OTU3407	LCOT01000026.4898.6395	Bacteria;Patescibacteria;Parcubacteria;Candidatus_Jorgensenbacteria;Parcubacteria_group_bacterium_GW2011_GWA2_47_8b	83.21	0	0.00105612	0	0	0
OTU341	JQ675500.1.1252	Bacteria;Proteobacteria;Alphaproteobacteria;Sphingomonadales;Sphingomonadaceae;Novosphingobium;uncultured_bacterium	100	0.0011083	0.00105612	0	0	0
OTU3412	FM207940.1.1448	Bacteria;Patescibacteria;Gracilibacteria;uncultured_Microgenomates_group_bacterium	91.58	0	0	0	0	0.00098733
OTU3414	FJ748799.1.1484	Bacteria;Gemmatimonadota;Gemmatimonadetes;Gemmatimonadales;Gemmatimonadaceae;uncultured;uncultured_bacterium	97.77	0	0	0	0.00102209	0
OTU3418	MF942642.1.1390	Bacteria;Verrucomicrobiota;Omnitrophia;Omnitrophales;Omnitrophaceae;Candidatus_Omnitrophus;uncultured_bacterium	90.91	0.0011083	0	0	0	0
OTU3419	KC682569.1.1445	Bacteria;Chloroflexi;Anaerolineae;Ardenticatenales;Ardenticatenaceae;uncultured;uncultured_bacterium	96.77	0	0	0	0	0.00098733
OTU342	AY970951.1.1520	Bacteria;Proteobacteria;Gammaproteobacteria;Pseudomonadales;Pseudomonadaceae;Pseudomonas;Pseudomonas_vranovensis	100	0.0011083	0.00211224	0.0041602	0.02146383	0.54895688
OTU3420	JQ977079.1.1423	Bacteria;Actinobacteriota;Actinobacteria;Micrococcales;Intrasporangiaceae;Knoellia;Knoellia_sp._Bra22	100	0	0.00105612	0	0.00102209	0.00098733
OTU3423	JX505033.1.1422	Bacteria;Chloroflexi;Anaerolineae;RBG-13-54-9;uncultured_Anaerolineaceae_bacterium	95.04	0	0	0.00138673	0	0
OTU3424	HQ857716.1.1431	Bacteria;Proteobacteria;Alphaproteobacteria;Rhodobacterales;Rhodobacteraceae;Hwanghaeicola;uncultured_Rhodospirillaceae_bacterium	97.76	0.0011083	0	0	0	0
OTU3426	FPLS01055849.17.1484	Bacteria;Proteobacteria;Alphaproteobacteria;Rhodospirillales;uncultured;metagenome	96.27	0	0	0	0.00102209	0
OTU3431	ET134907.1.1350	Bacteria;Verrucomicrobiota;Omnitrophia;Omnitrophales;Omnitrophaceae;Candidatus_Omnitrophus;uncultured_bacterium	96.5	0	0	0.00138673	0	0
OTU3433	CP016460.3086814.3088288	Bacteria;Proteobacteria;Alphaproteobacteria;Sphingomonadales;Sphingomonadaceae;Blastomonas;Blastomonas_sp._RAC04	99	0	0	0.00138673	0.00102209	0
OTU3436	KF733329.1.1428	Bacteria;Proteobacteria;Gammaproteobacteria;Pseudomonadales;Pseudomonadaceae;Pseudomonas;Pseudomonas_sp._B12(2014)	99.77	0	0	0	0	0.08885993
OTU3437	HM445333.1.1499	Bacteria;Acidobacteriota;Acidobacteriae;Subgroup_2;uncultured_bacterium	94.7	0	0.00105612	0.00138673	0	0
OTU3440	DQ856515.1.1511	Bacteria;Proteobacteria;Gammaproteobacteria;Xanthomonadales;Xanthomonadaceae;Thermomonas;uncultured_bacterium	100	0.0011083	0	0.00138673	0	0
OTU3442	AB551445.1.1301	Bacteria;Cyanobacteria;Cyanobacteriia;Cyanobacteriales;Nostocaceae;Dolichospermum_NIES41;Dolichospermum_affine_TAC439	99.51	0.0011083	0	0	0	0
OTU345	ET466699.1.1406	Bacteria;Firmicutes;Bacilli;Bacillales;Planococcaceae;uncultured;uncultured_bacterium	99.53	0.00221661	0.00211224	0.18443532	0.4108791	0.25176979
OTU3450	KF616728.1.1486	Bacteria;Myxococcota;bacteriap25;uncultured_bacterium	90.91	0.0011083	0	0	0	0
OTU3452	LCCV01000010.74269.78529	Bacteria;Patescibacteria;Parcubacteria;Candidatus_Brennerbacteria;Parcubacteria_group_bacterium_GW2011_GWA2_42_14	85.96	0	0	0.00138673	0	0
OTU346	AF005008.1.1490	Bacteria;Actinobacteriota;Actinobacteria;Propionibacteriales;Nocardioidaceae;Nocardioides;Nocardioides_plantarum	99.76	0.0011083	0	0.0041602	0.00102209	0.00098733
OTU3461	LCOT01000026.4898.6395	Bacteria;Patescibacteria;Parcubacteria;Candidatus_Jorgensenbacteria;Parcubacteria_group_bacterium_GW2011_GWA2_47_8b	79.94	0	0	0.00138673	0	0
OTU3462	KF836339.1.1492	Bacteria;SAR324_clade(Marine_group_B);uncultured_bacterium	98.76	0.0011083	0	0	0	0
OTU3463	JF915320.1.1384	Bacteria;Bacteroidota;Bacteroidia;Flavobacteriales;Flavobacteriaceae;Flavobacterium;Flavobacterium_psychrolimnae	100	0	0	0	0	0.00098733
OTU3466	AJ583207.1.1384	Bacteria;Elusimicrobiota;Elusimicrobia;Lineage_IV;uncultured_bacterium	95.41	0	0	0.00138673	0	0
OTU3467	HQ730653.1.1464	Bacteria;Acidobacteriota;Acidobacteriae;Acidobacteriales;Acidobacteriaceae_(Subgroup_1);uncultured;uncultured_Acidobacterium_sp.	85.01	0	0	0	0	0.00098733
OTU3469	JX521228.1.1455	Bacteria;Proteobacteria;Alphaproteobacteria;Sphingomonadales;Sphingomonadaceae;Rhizorhapis;uncultured_bacterium	99.75	0.0011083	0	0	0	0
OTU347	MFTV01000001.3080.4539	Bacteria;Patescibacteria;Parcubacteria;TBA9983;Candidatus_Nomurabacteria_bacterium_RIFCSPHIGHO2_02_40_30	96.16	0	0.00105612	0	0.00102209	0
OTU3470	JQ278831.1.1499	Bacteria;Proteobacteria;Gammaproteobacteria;Burkholderiales;Gallionellaceae;Gallionella;uncultured_beta_proteobacterium	99.77	0	0	0	0	0.00098733
OTU3472	HQ120546.1.1496	Bacteria;Bacteroidota;Bacteroidia;Flavobacteriales;Flavobacteriaceae;Flavobacterium;uncultured_bacterium	98.82	0.0011083	0.00105612	0	0.00306626	0.07404994
OTU3473	EF019116.1.1389	Bacteria;Acidobacteriota;Acidobacteriae;Subgroup_2;uncultured_bacterium	96.15	0	0	0	0.00102209	0
OTU3474	LCCV01000010.74269.78529	Bacteria;Patescibacteria;Parcubacteria;Candidatus_Brennerbacteria;Parcubacteria_group_bacterium_GW2011_GWA2_42_14	89.37	0	0	0.00138673	0	0
OTU3475	AJ871433.1.1454	Bacteria;Proteobacteria;Alphaproteobacteria;Rhizobiales;Beijerinckiaceae;Microvirga;Chelatococcus_asaccharovorans	99.75	0	0	0.00138673	0	0
OTU3476	EF516218.1.1461	Bacteria;Bdellovibrionota;Bdellovibrionia;Bdellovibrionales;Bdellovibrionaceae;Bdellovibrio;uncultured_bacterium	88.76	0.0011083	0.00105612	0	0	0
OTU3477	JF265912.1.1363	Bacteria;Nitrospirota;Nitrospiria;Nitrospirales;Nitrospiraceae;Nitrospira;uncultured_bacterium	98.33	0	0.00105612	0.00138673	0	0.00098733
OTU348	KF836147.1.1531	Bacteria;Nitrospirota;Leptospirillia;Leptospirillales;Leptospirillaceae;Leptospirillum;uncultured_bacterium	91.36	0	0.00105612	0	0.00102209	0

OTU3480	ASMR01000001.18974.20405	Archaea;Iainarchaeota;Iainarchaeia;Iainarchaeales;Candidatus_Iainarchaeum;Diapherotrites_archaeon_SCGC_AAA011-K09	81.63	0.0011083	0	0	0	0
OTU3483	FPLM01005745.11.1472	Bacteria;Proteobacteria;Alphaproteobacteria;Rhizobiales;Hyphomicrobiaceae;Hyphomicrobium;metagenome	99.5	0	0.00316837	0	0	0.00197467
OTU349	ET283598.1.1355	Bacteria;Patescibacteria;Gracilibacteria;uncultured_bacterium	87.62	0.0011083	0.00105612	0	0.00102209	0.00098733
OTU3495	HM445391.1.1288	Bacteria;Proteobacteria;Alphaproteobacteria;Caulobacterales;Hyphomonadaceae;SWB02;uncultured_bacterium	98.76	0.00221661	0	0	0	0
OTU35	AB042288.1.1486	Bacteria;Actinobacteriota;Actinobacteria;Propionibacteriales;Propionibacteriaceae;Cutibacterium;Cutibacterium_acnes	100	0.01773285	0.01689796	0.02080098	0.01226505	0.01086066
OTU350	JN232909.1.1507	Bacteria;Proteobacteria;Gammaproteobacteria;Burkholderiales;Methylophilaceae;uncultured;uncultured_bacterium	99.3	0.00221661	0.00316837	0.00277346	0.00102209	0.00098733
OTU3504	ET335173.1.1507	Bacteria;Acidobacteriota;Acidobacteriae;Subgroup_2;uncultured_bacterium	92.36	0	0	0	0.00102209	0
OTU351	HM186378.1.1303	Bacteria;Patescibacteria;Parcubacteria;Candidatus_Yanofskybacteria;uncultured_bacterium	91.81	0	0.00105612	0	0	0
OTU3510	JQ278816.1.1534	Bacteria;Verrucomicrobiota;Omnitrophia;Omnitrophales;Omnitrophaceae;Candidatus_Omnitrophus;uncultured_bacterium	97.18	0	0	0	0.00102209	0
OTU3511	ET135280.1.1361	Bacteria;Planctomycetota;Phycisphaerae;CCM11a;uncultured_bacterium	97.01	0	0.00105612	0	0	0
OTU3513	FJ809934.1.1530	Bacteria;Firmicutes;Bacilli;Bacillales;Planococcaceae;Domibacillus;Bacillus_sp._AER315-13	100	0	0	0	0	0.00098733
OTU3514	FPLM01002935.1.1311	Bacteria;Proteobacteria;Alphaproteobacteria;Rhodospirillales;uncultured;metagenome	95.77	0	0	0.00138673	0	0
OTU3517	KF836147.1.1531	Bacteria;Nitrospirota;Leptospirillia;Leptospirillales;Leptospirillaceae;Leptospirillum;uncultured_bacterium	95.1	0	0	0	0.00102209	0
OTU3519	FJ611855.1.1366	Bacteria;Proteobacteria;Gammaproteobacteria;Enterobacterales;Erwiniaceae;Pantoea;Pantoea_stewartii	99.77	0.07314803	0	0	0	0.00098733
OTU3522	KF616728.1.1486	Bacteria;Myxococcota;bacteriap25;uncultured_bacterium	90.68	0.0011083	0	0	0	0
OTU3524	DQ499301.1.1524	Bacteria;Methylomirabilota;Methylomirabilia;Rokubacteriales;uncultured_bacterium	99.53	0	0	0.00138673	0	0
OTU3526	HM128505.1.1387	Bacteria;Proteobacteria;Alphaproteobacteria;Rhodobacterales;Rhodobacteraceae;Tabrizicola;uncultured_bacterium	100	0	0	0.00277346	0	0
OTU353	ET240202.1.1337	Bacteria;Proteobacteria;Gammaproteobacteria;Pseudomonadales;Moraxellaceae;Acinetobacter;Acinetobacter_sp._B1(2015)	100	0	0.00211224	0.00138673	0	0.00197467
OTU3531	AB006125.1.1294	Bacteria;Firmicutes;Bacilli;Lactobacillales;Streptococcaceae;Streptococcus;Streptococcus_parasanguinis	100	0.0011083	0	0	0	0
OTU3532	KX123508.1.1526	Bacteria;Patescibacteria;Parcubacteria;Candidatus_Liptonbacteria;Parcubacteria_group_bacterium_GW2011_GWA1_60_11	81.48	0	0	0	0	0.00098733
OTU3538	AY608605.1.1505	Bacteria;Firmicutes;Bacilli;Bacillales;Bacillaceae;Bacillus;Bacillus_litoralis	100	0	0	0	0.00306626	0.00098733
OTU3539	KC569964.1.1362	Bacteria;Proteobacteria;Gammaproteobacteria;PLTA13;uncultured_bacterium	99.3	0	0.00105612	0	0.00102209	0
OTU3542	FJ946627.1.1290	Bacteria;Acidobacteriota;Blastocatellia;Blastocatellales;Blastocatellaceae;Stenotrophobacter;uncultured_bacterium_gp4	99.75	0	0	0.09013756	0	0
OTU3543	FJ719059.1.1478	Bacteria;Verrucomicrobiota;Omnitrophia;Omnitrophales;Omnitrophaceae;Candidatus_Omnitrophus;uncultured_bacterium	88.26	0	0	0	0.00204417	0
OTU3545	LC140851.1.1514	Bacteria;Proteobacteria;Gammaproteobacteria;Enterobacterales;Vibrionaceae;Vibrio;uncultured_Vibrio_sp.	100	0.0011083	0	0	0.00102209	0
OTU3552	AM691112.1.1456	Bacteria;Proteobacteria;Gammaproteobacteria;Burkholderiales;Oxalobacteraceae;Massilia;beta_proteobacterium_JV3	100	0.0011083	0.00105612	0	0	0.00098733
OTU3556	MGSO01000007.47053.48554	Bacteria;Myxococcota;bacteriap25;Deltaproteobacteria_bacterium_RIFCSPLOWO2_12_FTLL_44_12	89.98	0	0	0	0.00102209	0
OTU356	KJ578067.1.1490	Bacteria;Bacteroidota;Kryptonia;Kryptoniales;BSV26;uncultured_bacterium	96.42	0	0	0	0.00102209	0
OTU3560	JX221875.1.1507	Bacteria;Proteobacteria;Gammaproteobacteria;Burkholderiales;TRA3-20;uncultured_bacterium	98.83	0	0	0.00138673	0.00102209	0
OTU3564	KX932590.1.1393	Bacteria;Proteobacteria;Gammaproteobacteria;Piscirickettsiales;Piscirickettsiaceae;Candidatus_Endoecteinascidia;uncultured_marine_bacterium	89.93	0.0011083	0	0.00138673	0	0
OTU3566	AJ564859.1.1495	Bacteria;Actinobacteriota;Actinobacteria;Micrococcales;Brevibacteriaceae;Brevibacterium;Brevibacterium_sanguinis	100	0	0	0.00277346	0.00102209	0
OTU3569	HM187569.1.1333	Archaea;Thermoplasmatota;Thermoplasmata;Methanomassiliicoccales;uncultured;uncultured_archaeon	98.44	0	0.00105612	0	0	0
OTU357	DQ453810.1.1496	Bacteria;Proteobacteria;Gammaproteobacteria;Pseudomonadales;Pseudomonadaceae;Pseudomonas;Pseudomonas_sp._m1(2006)	99.53	0.00443321	0.00211224	1.00954071	0.00204417	0.00098733
OTU3571	AB071382.1.1400	Bacteria;Bacteroidota;Bacteroidia;Flavobacteriales;Flavobacteriaceae;Vitellibacter;Aequorivita_vladivostokensis	93.85	0	0.00105612	0	0.0725682	0
OTU3573	JN446047.1.1377	Bacteria;Bdellovibrionota;Bdellovibrionia;Bdellovibrionales;Bdellovibrionaceae;Bdellovibrio;uncultured_organism	88.31	0	0	0	0	0.00098733
OTU3575	ET134909.1.1431	Bacteria;Verrucomicrobiota;Omnitrophia;Omnitrophales;Omnitrophaceae;Candidatus_Omnitrophus;uncultured_bacterium	92.56	0	0.00105612	0	0	0
OTU3576	JF265705.1.1359	Bacteria;Bacteroidota;Bacteroidia;Sphingobacteriales;AKYH767;uncultured_bacterium	99.29	0.0011083	0	0	0	0
OTU3584	ET286996.1.1484	Bacteria;Chloroflexi;Dehalococcoidia;SAR202_clade;uncultured_bacterium	95.53	0	0	0	0	0.00098733
OTU359	HG969252.1.1474	Bacteria;Proteobacteria;Gammaproteobacteria;Pseudomonadales;Marinobacteraceae;Marinobacter;Tamilnaduibacter_salinus	94.38	0.0011083	0.00422449	0.00138673	0.00102209	0.00098733
OTU3590	HE648194.1.1418	Bacteria;Patescibacteria;Gracilibacteria;Candidatus_Peregrinibacteria;uncultured_bacterium	82.59	0	0	0	0.00102209	0
OTU3593	JQ278884.1.1533	Bacteria;Verrucomicrobiota;Omnitrophia;Omnitrophales;Omnitrophaceae;Candidatus_Omnitrophus;uncultured_bacterium	98.35	0	0.00105612	0	0	0
OTU3598	DQ297957.1.1483	Bacteria;Bacteroidota;Bacteroidia;Sphingobacteriales;Sphingobacteriaceae;Pedobacter;uncultured_soil_bacterium	99.53	0	0	0	0	0.00098733
OTU3599	AB008209.1.1566	Bacteria;Firmicutes;Bacilli;Lactobacillales;Lactobacillaceae;Lactobacillus;Lactobacillus_gasseri	100	0	0	0	0	0.00098733
OTU36	HQ120546.1.1496	Bacteria;Bacteroidota;Bacteroidia;Flavobacteriales;Flavobacteriaceae;Flavobacterium;uncultured_bacterium	100	0.01108303	0.00422449	0.10539161	0.00613252	6.73459514
OTU360	KY356869.1.921	Archaea;Nanoarchaeota;Nanoarchaeia;Woesearchaeales;GW2011_GWC1_47_15;uncultured_archaeon	85.22	0.0011083	0.00211224	0	0	0
OTU3602	KT569325.1.1455	Bacteria;Cyanobacteria;Cyanobacteriia;Phormidesmiales;Nodosilineaceae;Nodosilinea_PCC-7104;Nodosilinea_nodulosa_LEGE_06104	99.26	0	0	0.00277346	0	0
OTU3603	MFRI01000031.4327.5856	Bacteria;Margulisbacteria;Candidatus_Margulisbacteria_bacterium_GWF2_38_17	84.04	0	0	0	0	0.00098733
OTU361	DQ058677.1.1546	Bacteria;Nitrospirota;Leptospirillia;Leptospirillales;Leptospirillaceae;Leptospirillum;uncultured_bacterium	99.77	0	0.00105612	0	0	0.00098733
OTU3611	JF219465.1.1310	Bacteria;Cyanobacteria;Cyanobacteriia;Cyanobacteriales;Chroococcidiopsaceae;uncultured;uncultured_bacterium	100	0	0	0.00138673	0	0
OTU3615	AM176873.1.1455	Bacteria;Proteobacteria;Gammaproteobacteria;Pseudomonadales;Halieaceae;Halioglobus;uncultured_bacterium	99.53	0.0011083	0	0	0	0
OTU362	MFTT01000025.14894.16462	Bacteria;Patescibacteria;Parcubacteria;Candidatus_Nomurabacteria;Candidatus_Nomurabacteria_bacterium_RIFCSPHIGHO2_01_FTLL_42_16	79.31	0	0	0	0	0.00098733
OTU3628	AB753963.1.1449	Bacteria;Bacteroidota;Bacteroidia;Flavobacteriales;Crocinitomicaceae;Fluviicola;uncultured_bacterium	100	0	0	0.00138673	0	0
OTU363	JX426859.1.954	Archaea;Nanoarchaeota;Nanoarchaeia;Woesearchaeales;uncultured_archaeon	85.26	0	0.00211224	0.00138673	0	0
OTU3638	AJ293462.7.1536	Bacteria;Proteobacteria;Gammaproteobacteria;Xanthomonadales;Xanthomonadaceae;Stenotrophomonas;Stenotrophomonas_rhizophila	100	0	0	0	0.04088349	0
OTU364	AB175629.1.1435	Bacteria;Proteobacteria;Alphaproteobacteria;Rhizobiales;Beijerinckiaceae;Methylobacterium-Methylorubrum;Methylorubrum_aminovorans	100	0	0	0.00554693	0	0.00098733
OTU3642	MHST01000019.19787.21369	Bacteria;Patescibacteria;Parcubacteria;Candidatus_Terrybacteria;Candidatus_Terrybacteria_bacterium_RIFCSPHIGHO2_01_FTLL_58_15	89.66	0	0	0	0	0.00098733
OTU3644	KP686648.1.1428	Bacteria;Patescibacteria;Parcubacteria;uncultured_bacterium	81.82	0.0011083	0	0	0	0
OTU3645	KJ566470.1.959	Archaea;Nanoarchaeota;Nanoarchaeia;Woesearchaeales;uncultured_euryarchaeote	90.82	0.0011083	0.00105612	0	0	0
OTU3646	LCOT01000026.4898.6395	Bacteria;Patescibacteria;Parcubacteria;Candidatus_Jorgensenbacteria;Parcubacteria_group_bacterium_GW2011_GWA2_47_8b	81.92	0	0.00105612	0	0	0
OTU365	HM129181.1.1450	Bacteria;Proteobacteria;Gammaproteobacteria;Burkholderiales;Comamonadaceae;Limnohabitans;uncultured_bacterium	100	0.00221661	0.00105612	0.00277346	0.00204417	0.00197467
OTU3651	MHJF01000011.24156.25754	Bacteria;Patescibacteria;Parcubacteria;Candidatus_Colwellbacteria;Candidatus_Harrisonbacteria_bacterium_RIFCSPHIGHO2_02_FTLL_40_20	84.12	0	0	0	0.00102209	0
OTU3659	KX033858.1.1518	Bacteria;Proteobacteria;Gammaproteobacteria;Enterobacterales;Alteromonadaceae;Rheinheimera;Rheinheimera_sp._SA_1	99.53	0.0011083	0.00211224	0.00138673	0.00102209	0.07306261
OTU366	FM875664.1.1470	Bacteria;Actinobacteriota;Actinobacteria;Corynebacteriales;Corynebacteriaceae;Corynebacterium;uncultured_bacterium	100	0	0.00316837	0	0	0.00098733
OTU3664	KF877720.1.1450	Bacteria;Proteobacteria;Alphaproteobacteria;Sphingomonadales;Sphingomonadaceae;uncultured;Sphingosinicella_sp._Z7	100	0	0.00105612	0	0	0.00098733
OTU367	GT305816.1.1450	Bacteria;Cyanobacteria;Cyanobacteriia;Synechococcales;Cyanobiaceae;Cyanobium_PCC-6307;uncultured_bacterium	99.5	0.00221661	0	0	0	0
OTU3674	HQ385626.1.1374	Bacteria;Bacteroidota;Bacteroidia;Chitinophagales;Saprospiraceae;uncultured;uncultured_bacterium	95.04	0	0	0.00138673	0	0
OTU3675	FJ902074.1.1313	Bacteria;Verrucomicrobiota;Omnitrophia;Omnitrophales;Omnitrophaceae;Candidatus_Omnitrophus;uncultured_Omnitrophica_bacterium	84.78	0.0011083	0	0	0	0

OTU368	CP023277.27833.29345	Bacteria;Proteobacteria;Gammaproteobacteria;Burkholderiales;Burkholderiaceae;Polynucleobacter;Polynucleobacter_acidiphobus	100	0.00443321	0.00633673	0.00277346	0	0.00098733
OTU3684	MGEH01000004.22459.23970	Bacteria;Patescibacteria;ABY1;Candidatus_Magasanikbacteria;Candidatus_Thrbacteria_bacterium_RIFCSPHIGHO2_12_FTLL_60_25	90.32	0.0011083	0	0	0	0.00098733
OTU369	KF428110.1.1484	Bacteria;Actinobacteriota;Actinobacteria;Corynebacteriales;Mycobacteriaceae;Mycobacterium;uncultured_bacterium	100	0.00443321	0	0.0041602	0.00204417	0
OTU37	CP016768.598628.600152	Bacteria;Actinobacteriota;Actinobacteria;Frankiales;Sporichthyaceae;hgcI_clade;Candidatus_Nanopelagicus_limnes	100	0.00775812	0.01056122	0.07211005	0.0081767	0.00493666
OTU3700	JN033190.1.1435	Bacteria;Fusobacteriota;Fusobacteriia;Fusobacteriales;Fusobacteriaceae;Cetobacterium;uncultured_bacterium	98.77	0	0	0.00138673	0	0
OTU3705	MHGG01000050.25339.26910	Bacteria;Verrucomicrobiota;Omnitrophia;Omnitrophales;Omnitrophaceae;Candidatus_Omnitrophus;Omnitrophica_WOR_2_bacterium_GWF2_63_9	91.75	0	0	0.00138673	0	0
OTU3708	JN397783.1.1446	Bacteria;Bacteroidota;Bacteroidia;Bacteroidales;SB-5;uncultured_bacterium	90.52	0.0011083	0	0	0	0
OTU371	ET133342.1.1247	Bacteria;Proteobacteria;Alphaproteobacteria;Caulobacterales;Hyphomonadaceae;SWB02;uncultured_bacterium	100	0.00221661	0.00844898	0.00138673	0	0.00098733
OTU3712	HQ692033.1.1354	Bacteria;Bacteroidota;Bacteroidia;Sphingobacteriales;ST-12K33;uncultured_Cytophagales_bacterium	95.26	0	0	0.00138673	0	0
OTU3714	LN562844.1.1336	Bacteria;Chloroflexi;Chloroflexia;Thermomicrobiales;JG30-KF-CM45;uncultured_bacterium	99.75	0.0011083	0	0	0	0
OTU3716	ET937845.1.1519	Bacteria;Myxococcota;bacteriap25;uncultured_bacterium	90.93	0	0	0.00138673	0	0
OTU372	FPLM01008234.16.1533	Bacteria;Proteobacteria;Gammaproteobacteria;Burkholderiales;Rhodocyclaceae;Candidatus_Accumulibacter;metagenome	99.53	0	0.00105612	0.0041602	0	0.00098733
OTU3720	JX221965.1.1519	Bacteria;Myxococcota;bacteriap25;uncultured_bacterium	94.63	0	0.00105612	0	0	0
OTU3721	AY928239.1.1291	Bacteria;Proteobacteria;Alphaproteobacteria;Rhizobiales;Beijerinckiaceae;Bosea;bacterium_11RO3	100	0.0011083	0	0	0	0.00098733
OTU3722	HM187241.1.1433	Bacteria;Chloroflexi;Dehalococcoidia;SAR202_clade;uncultured_bacterium	98.01	0	0	0	0.00102209	0
OTU3724	MNDM01000022.26933.28470	Bacteria;Nitrospirota;Nitrospiria;Nitrospirales;Nitrospiraceae;Nitrospira;Nitrospirae_bacterium_13_2_20CM_2_62_8	99.76	0.0011083	0	0	0	0.00098733
OTU3726	HM186624.1.1368	Bacteria;Bacteroidota;Kryptonia;Kryptoniales;BSV26;uncultured_bacterium	94.3	0	0.00105612	0	0	0
OTU373	JX948487.1.1485	Bacteria;Bacteroidota;Bacteroidia;Flavobacteriales;Crocinitomicaceae;Fluviicola;uncultured_bacterium	99.05	0.00554152	0.00211224	0.00554693	0.00102209	0
OTU3732	DQ906827.1.1473	Bacteria;Myxococcota;Myxococcia;Myxococcales;Myxococcaceae;uncultured;uncultured_bacterium	95.31	0	0	0	0	0.00098733
OTU3735	FPLL01006513.12.1474	Bacteria;Proteobacteria;Alphaproteobacteria;Rickettsiales;Mitochondria;metagenome	95.77	0	0	0.00138673	0	0
OTU374	JN868758.1.1506	Bacteria;Bacteroidota;Bacteroidia;Flavobacteriales;Flavobacteriaceae;Flavobacterium;uncultured_bacterium	100	0	0.00105612	0.0041602	0.00102209	0.00098733
OTU3740	AB023428.1.1395	Bacteria;Proteobacteria;Alphaproteobacteria;Caulobacterales;Caulobacteraceae;Brevundimonas;Brevundimonas_bullata	100	0	0	0.00138673	0	0.00197467
OTU375	FPLS01007037.11.1343	Bacteria;Verrucomicrobiota;Verrucomicrobiae;Pedosphaerales;Pedosphaeraceae;metagenome	99.06	0.00332491	0	0	0.00204417	0
OTU3753	GQ302574.1.1531	Bacteria;Latescibacterota;uncultured_Acidobacterium_sp.	99.77	0.0011083	0	0	0	0
OTU3762	JQ278980.1.1483	Bacteria;Bacteroidota;Bacteroidia;Sphingobacteriales;env.OPS_17;uncultured_Bacteroidetes_bacterium	95.99	0	0	0	0.00102209	0
OTU3766	AF216948.1.1407	Bacteria;Cyanobacteria;Cyanobacteriia;Synechococcales;Cyanobiaceae;Cyanobium_PCC-6307;Synechococcus_sp._PCC_7920	100	0	0	0.00277346	0	0
OTU3769	MF942642.1.1390	Bacteria;Verrucomicrobiota;Omnitrophia;Omnitrophales;Omnitrophaceae;Candidatus_Omnitrophus;uncultured_bacterium	92.07	0	0.00105612	0	0	0
OTU377	HAFO01014186.601.2065	Bacteria;Cyanobacteria;Cyanobacteriia;Synechococcales;Cyanobiaceae;Cyanobium_PCC-6307;Troglena_sp._WA34KE	100	0	0.00105612	0.00138673	0	0.00197467
OTU3773	MF942642.1.1390	Bacteria;Verrucomicrobiota;Omnitrophia;Omnitrophales;Omnitrophaceae;Candidatus_Omnitrophus;uncultured_bacterium	91.61	0	0	0.00138673	0	0
OTU3776	CP001120.4390086.4391619	Bacteria;Proteobacteria;Gammaproteobacteria;Enterobacterales;Enterobacteriaceae;Salmonella;Salmonella_enterica_subsp._enterica_serovar_Heidelberg_str._SL476	99.53	0	0	0.00138673	0	0.00098733
OTU3778	HE602793.1.1386	Bacteria;Bacteroidota;Bacteroidia;Cytophagales;Microscillaceae;uncultured;uncultured_bacterium	95.72	0	0.00105612	0	0	0.00098733
OTU378	HQ203977.1.1485	Bacteria;Campylobacterota;Campylobacteria;Campylobacterales;Arcobacteraceae;uncultured;uncultured_bacterium	100	0.00443321	0	0.00138673	0	0
OTU3785	HE602802.1.1436	Bacteria;Elusimicrobiota;Lineage_IIb;uncultured_bacterium	93.85	0	0	0	0	0.00098733
OTU3789	LCOT01000026.4898.6395	Bacteria;Patescibacteria;Parcubacteria;Candidatus_Jorgensenbacteria;Parcubacteria_group_bacterium_GW2011_GWA2_47_8b	91	0.0011083	0	0	0	0.00098733
OTU379	JF703349.1.1490	Bacteria;Proteobacteria;Alphaproteobacteria;Rhizobiales;A0839;uncultured_Alphaproteobacteria_bacterium	100	0.00332491	0.00105612	0	0.00102209	0.00493666
OTU3790	KY356876.1.911	Archaea;Nanoarchaeota;Nanoarchaeia;Woesearchaeales;GW2011_GWC1_47_15;uncultured_archaeon	87.34	0.0011083	0	0.00138673	0	0
OTU38	KF063291.1.1360	Bacteria;Proteobacteria;Gammaproteobacteria;Burkholderiales;Neisseriaceae;uncultured;uncultured_bacterium	100	0.01329964	0.01056122	0.01248059	0.01022087	0.00987333
OTU380	AF513089.1.1453	Bacteria;Proteobacteria;Gammaproteobacteria;Burkholderiales;Comamonadaceae;Aquabacterium;uncultured_bacterium	100	0	0.00211224	0	0.00102209	0
OTU381	AF373196.1.1490	Bacteria;Proteobacteria;Gammaproteobacteria;Enterobacterales;Erwiniaceae;Pantoea;Pantoea_agglomerans	100	0.02216607	0.4256173	0.00138673	0	0
OTU3811	MHJF01000011.24156.25754	Bacteria;Patescibacteria;Parcubacteria;Candidatus_Colwellbacteria;Candidatus_Harrisonbacteria_bacterium_RIFCSPHIGHO2_02_FTLL_40_20	84.77	0	0.00105612	0	0	0
OTU3817	KP308718.482.1864	Archaea;Nanoarchaeota;Nanoarchaeia;Woesearchaeales;uncultured_archaeon	91.19	0.0011083	0	0	0	0
OTU3821	ET375072.1.1209	Bacteria;Proteobacteria;Alphaproteobacteria;Sphingomonadales;Sphingomonadaceae;Altererythrobacter;uncultured_Sphingomonadales_bacterium	100	0	0	0	0.00102209	0
OTU3825	ET294005.1.1445	Bacteria;Proteobacteria;Gammaproteobacteria;Pseudomonadales;Halomonadaceae;Salinicola;Halomonas_sp._ny1-3	100	0	0	0.00277346	0	0.00197467
OTU3826	KY241528.1.1521	Bacteria;Marinimicrobia_(SAR406_clade);uncultured_bacterium	96.96	0	0	0	0.00102209	0
OTU3828	LCQK01000003.64973.66448	Bacteria;Patescibacteria;Parcubacteria;Candidatus_Jorgensenbacteria;Candidatus_Jorgensenbacteria_bacterium_GW2011_GWB1_50_10	83.16	0.0011083	0.00105612	0	0	0
OTU383	FLOH01001871.338.1831	Bacteria;Campylobacterota;Campylobacteria;Campylobacterales;Sulfurimonadaceae;Sulfurimonas;marine_metagenome	99.25	0.0011083	0	0	0.00102209	0
OTU3833	AQSZ01000059.14608.16151	Bacteria;Verrucomicrobiota;Omnitrophia;Omnitrophales;Omnitrophaceae;Candidatus_Omnitrophus;Omnitrophica_bacterium_SCGC_AAA257-O07	95.56	0	0.00105612	0	0	0
OTU3834	HG315619.1.1624	Bacteria;Proteobacteria;Alphaproteobacteria;Rickettsiales;Rickettsiaceae;Candidatus_Trichorickettsia;Candidatus_Trichorickettsia_mobilis	96.52	0.0011083	0	0	0	0
OTU3836	MHGG01000050.25339.26910	Bacteria;Verrucomicrobiota;Omnitrophia;Omnitrophales;Omnitrophaceae;Candidatus_Omnitrophus;Omnitrophica_WOR_2_bacterium_GWF2_63_9	93.71	0.0011083	0	0	0	0
OTU3839	JQ278884.1.1533	Bacteria;Verrucomicrobiota;Omnitrophia;Omnitrophales;Omnitrophaceae;Candidatus_Omnitrophus;uncultured_bacterium	90.82	0.0011083	0	0	0	0
OTU384	HQ857761.1.1442	Bacteria;Proteobacteria;Gammaproteobacteria;Pseudomonadales;Moraxellaceae;Acinetobacter;Acinetobacter_ursingii	100	0	0.00211224	0.00138673	0.00102209	0.00098733
OTU3844	AB045094.1.1500	Bacteria;Firmicutes;Bacilli;Paenibacillales;Paenibacillaceae;Paenibacillus;Paenibacillus_pabuli	99.06	0.00775812	0	0.04160195	0.13593761	0.00197467
OTU3845	JF116235.1.1374	Bacteria;Bdellovibrionota;Oligoflexia;0319-6G20;uncultured_bacterium	93.46	0	0	0	0.00102209	0
OTU3856	FM177504.1.1465	Bacteria;Cyanobacteria;Cyanobacteriia;Cyanobacteriales;Microcystaceae;Microcystis_PCC-7914;Synechocystis_sp._2LT05S06	93.09	0	0	0.00138673	0	0
OTU3859	AY821999.1.1276	Archaea;Nanoarchaeota;Nanoarchaeia;Woesearchaeales;uncultured_euryarchaeote	87.43	0	0.00105612	0	0	0
OTU3860	KC331508.1.1507	Bacteria;Patescibacteria;Parcubacteria;Candidatus_Nomurabacteria;uncultured_bacterium	97.59	0	0.00105612	0	0	0
OTU3861	CP025544.661010.662555	Bacteria;Dependentiae;Babeliae;Babeliales;TBA12409;Candidatus_Dependentiae_bacterium_(ex_Spumella_elongata_CCAP_955/1)	97.42	0.0011083	0	0	0.00102209	0
OTU3868	KC358051.1.1266	Bacteria;Fibrobacterota;Fibrobacteria;Fibrobacterales;Fibrobacteraceae;uncultured;uncultured_bacterium	99.28	0	0	0.00138673	0	0
OTU387	ET703456.1.1490	Bacteria;Bacteroidota;Bacteroidia;Flavobacteriales;Crocinitomicaceae;Fluviicola;uncultured_Fluviicola_sp.	99.53	0.0011083	0.00105612	0.00693366	0.00102209	0
OTU3871	CT921716.1.1311	Bacteria;Proteobacteria;Alphaproteobacteria;Rhizobiales;Rhizobiales_Incertae_Sedis;uncultured;uncultured_bacterium	100	0	0	0.00277346	0	0.00098733
OTU3873	JF266316.1.1341	Bacteria;Actinobacteriota;Actinobacteria;Pseudonocardiales;Pseudonocardiaceae;Crossiella;uncultured_bacterium	100	0	0	0	0.00102209	0
OTU388	AB626125.1.1460	Bacteria;Proteobacteria;Gammaproteobacteria;Pseudomonadales;Moraxellaceae;Acinetobacter;Acinetobacter_lwoffii	100	0.0011083	0	0.00138673	0.01022087	0.00197467
OTU3882	HM187298.1.1433	Bacteria;Proteobacteria;Alphaproteobacteria;uncultured;uncultured_bacterium	99.5	0.0011083	0	0	0	0
OTU389	AY922047.1.1353	Bacteria;Chloroflexi;KD4-96;uncultured_Chloroflexi_bacterium	100	0.0011083	0	0	0	0
OTU3892	MF942642.1.1390	Bacteria;Verrucomicrobiota;Omnitrophia;Omnitrophales;Omnitrophaceae;Candidatus_Omnitrophus;uncultured_bacterium	91.61	0	0.00105612	0	0	0
OTU3895	JQ278816.1.1534	Bacteria;Verrucomicrobiota;Omnitrophia;Omnitrophales;Omnitrophaceae;Candidatus_Omnitrophus;uncultured_bacterium	91.53	0	0	0	0.00102209	0
OTU3898	AB365795.1.1281	Bacteria;Proteobacteria;Alphaproteobacteria;Rhizobiales;Rhizobiaceae;Phyllobacterium;Phyllobacterium_sp._WR140	97.26	0	0.00105612	0	0	0

OTU39	KC432419.1.1293	Bacteria;Patescibacteria;Parcubacteria;Candidatus_Kaiserbacteria;uncultured_bacterium	95.06	0.00332491	0.0095051	0.00138673	0.00204417	0.00098733
OTU390	MG601368.1.1536	Bacteria;Verrucomicrobiota;Chlamydiae;LD1-PA32;uncultured_bacterium	83.22	0.0011083	0	0	0	0.00098733
OTU391	KX172280.1.1419	Bacteria;Patescibacteria;ABY1;Candidatus_Thrbacteria;uncultured_bacterium	88.12	0.0011083	0	0	0	0
OTU3916	JQ977389.1.1446	Bacteria;Proteobacteria;Gammaproteobacteria;Burkholderiales;Comamonadaceae;Hydrogenophaga;Hydrogenophaga_sp._Ama29	100	0	0	0.01525405	0.00102209	0.02764531
OTU392	KT826351.1.1469	Bacteria;Proteobacteria;Alphaproteobacteria;Elsterales;Elsteraceae;Elstera;Elstera_sp._TH171	100	0.0011083	0.00105612	0	0	0.00098733
OTU3921	GQ500755.1.1501	Bacteria;Proteobacteria;Gammaproteobacteria;Burkholderiales;Nitrosomonadaceae;mle1-7;uncultured_bacterium	99.06	0.0011083	0	0	0	0
OTU3923	AB193905.1.1466	Bacteria;Patescibacteria;Parcubacteria;Candidatus_Colwellbacteria;uncultured_bacterium	81.4	0.0011083	0	0	0	0
OTU3926	MFTV01000001.3080.4539	Bacteria;Patescibacteria;Parcubacteria;TBA9983;Candidatus_Nomurabacteria_bacterium_RIFCSPHIGHO2_02_40_30	82.21	0	0	0	0.00102209	0
OTU393	GQ487908.1.1451	Bacteria;Proteobacteria;Alphaproteobacteria;Sphingomonadales;Sphingomonadaceae;Sphingobium;uncultured_bacterium	100	0.0011083	0	0.00138673	0.00102209	0
OTU394	ET540131.1.1380	Bacteria;Actinobacteriota;Actinobacteria;Corynebacteriales;Corynebacteriaceae;Lawsonella;uncultured_bacterium	100	0	0.00211224	0.00277346	0.00102209	0
OTU3941	GQ389066.1.1520	Bacteria;Planctomycetota;OM190;uncultured_bacterium	98.12	0.0011083	0	0	0	0
OTU3942	AJ583207.1.1384	Bacteria;Elusimicrobiota;Elusimicrobia;Lineage_IV;uncultured_bacterium	96.14	0	0	0	0.00102209	0
OTU3947	JN397814.1.1475	Bacteria;Patescibacteria;Parcubacteria;uncultured_bacterium	96.06	0	0	0.00277346	0	0
OTU3949	ET731480.1.969	Archaea;Nanoarchaeota;Nanoarchaeia;Woesearchaeales;SCGC_AAA011-D5;uncultured_euryarchaeote	88.17	0	0.00211224	0	0	0
OTU3955	MF040398.1.1476	Bacteria;Bacteroidota;Bacteroidia;Flavobacteriales;Cryomorphaceae;uncultured;uncultured_bacterium	99.76	0.0011083	0	0	0	0
OTU396	DQ264427.1.1508	Bacteria;Proteobacteria;Gammaproteobacteria;Burkholderiales;Comamonadaceae;Acidovorax;uncultured_bacterium	100	0	0	0.00554693	0	0.00197467
OTU3961	HM128690.1.1453	Bacteria;Verrucomicrobiota;Verrucomicrobiae;Chthoniobacterales;Terrimicrobiaceae;FukuN18_freshwater_group;uncultured_bacterium	100	0	0.00105612	0.00138673	0	0
OTU3967	MF942666.1.1354	Bacteria;Planctomycetota;Pla4_lineage;uncultured_bacterium	95.55	0	0	0	0.00102209	0
OTU3969	FPLK01001318.15.1474	Bacteria;Bacteroidota;Kapabacteria;Kapabacteriales;metagenome	100	0	0	0.00138673	0.00102209	0
OTU397	MHOL01000019.20791.21763	Archaea;Nanoarchaeota;Nanoarchaeia;Woesearchaeales;SCGC_AAA011-D5;Candidatus_Staskawiczbacteria_bacterium_RIFCSPHIGHO2_01_FTLL_34_27	87.66	0.0011083	0	0	0	0
OTU3970	LN870758.1.1414	Bacteria;Verrucomicrobiota;Omnitrophia;Omnitrophales;Omnitrophaceae;Candidatus_Omnitrophus;uncultured_bacterium	89.67	0	0	0	0	0.00098733
OTU3973	AB861981.1.1489	Bacteria;Bacteroidota;Bacteroidia;Bacteroidales;Bacteroidaceae;Bacteroides;Bacteroides_caecigallinarum	98.58	0.0011083	0	0	0	0.002962
OTU3976	HM318949.1.1375	Bacteria;Bdellovibrionota;Oligoflexia;0319-6G20;uncultured_bacterium	96.5	0.0011083	0	0	0	0
OTU398	CP010350.1666491.1668035	Bacteria;Proteobacteria;Gammaproteobacteria;Pseudomonadales;Moraxellaceae;Acinetobacter;Acinetobacter_johnsonii_XBB1	100	0.0011083	0.00211224	0	0	0.00098733
OTU3980	DQ532168.1.1495	Bacteria;Proteobacteria;Gammaproteobacteria;Burkholderiales;Comamonadaceae;Pelomonas;uncultured_bacterium	100	0	0	0.00138673	0	0
OTU3982	MF942642.1.1390	Bacteria;Verrucomicrobiota;Omnitrophia;Omnitrophales;Omnitrophaceae;Candidatus_Omnitrophus;uncultured_bacterium	93.71	0	0	0.00138673	0	0
OTU3983	HM187450.1.1355	Bacteria;Verrucomicrobiota;Omnitrophia;Omnitrophales;Omnitrophaceae;Candidatus_Omnitrophus;uncultured_bacterium	93.72	0	0	0.00138673	0	0
OTU3986	ET803575.1.1493	Bacteria;Verrucomicrobiota;Verrucomicrobiae;uncultured;uncultured_bacterium	96.68	0.0011083	0	0	0.00102209	0
OTU399	DQ058674.1.1514	Bacteria;Nitrospirota;Nitrospiria;Nitrospirales;Nitrospiraceae;Nitrospira;uncultured_bacterium	98.81	0	0	0	0.00204417	0
OTU3991	AJ786666.1.1490	Bacteria;Actinobacteriota;Actinobacteria;Corynebacteriales;Nocardiaceae;Rhodococcus;Rhodococcus_wratislaviensis	100	0	0	0.00138673	0.00102209	0
OTU3996	KF836184.1.1496	Bacteria;Elusimicrobiota;Elusimicrobia;MVP-88;uncultured_bacterium	94.08	0	0.00105612	0	0	0
OTU4	CP024034.172000.173465	Bacteria;Proteobacteria;Alphaproteobacteria;SAR11_clade;Clade_III;Candidatus_Fonsibacter_ubiquis	100	0.04876535	0.05491836	0.04714888	0.02964053	0.03258197
OTU40	DQ058674.1.1514	Bacteria;Nitrospirota;Nitrospiria;Nitrospirales;Nitrospiraceae;Nitrospira;uncultured_bacterium	99.52	0.01108303	0.11511734	0.01248059	0.00919879	0.00888599
OTU400	KX123607.1.1467	Bacteria;Patescibacteria;Parcubacteria;Candidatus_Yanofskybacteria;Candidatus_Yanofskybacteria_bacterium_GW2011_GWF1_44_227	80.15	0.0011083	0	0	0	0
OTU4003	KF616728.1.1486	Bacteria;Myxococcota;bacteriap25;uncultured_bacterium	91.14	0.0011083	0	0	0	0
OTU401	AM934647.1.1486	Bacteria;Bacteroidota;Bacteroidia;Flavobacteriales;Flavobacteriaceae;Flavobacterium;Flavobacterium_sp._WB2.3-46	99.76	0.00221661	0.00844898	0	0.00204417	0.35938904
OTU4013	MHGG01000050.25339.26910	Bacteria;Verrucomicrobiota;Omnitrophia;Omnitrophales;Omnitrophaceae;Candidatus_Omnitrophus;Omnitrophica_WOR_2_bacterium_GWF2_63_9	91.61	0	0	0	0	0.00098733
OTU4014	AB042093.1.1510	Bacteria;Actinobacteriota;Actinobacteria;Micrococcales;Microbacteriaceae;Herbiconiux;Curtobacterium_sp._VKM_Ac-2058	100	0	0.00211224	0.00277346	0.00102209	0.00098733
OTU4015	ET700164.1.1497	Bacteria;Proteobacteria;Gammaproteobacteria;Burkholderiales;Rhodocyclaceae;C39;uncultured_bacterium	99.3	0	0	0.00277346	0	0
OTU4016	KT166990.1.1341	Bacteria;Proteobacteria;Alphaproteobacteria;uncultured;uncultured_bacterium	97.01	0	0.00105612	0	0	0
OTU402	ET244058.1.1476	Bacteria;Bacteroidota;Bacteroidia;Flavobacteriales;Flavobacteriaceae;Flavobacterium;uncultured_bacterium	100	0	0	0.02496117	0	0.002962
OTU4023	JF266152.1.1349	Bacteria;Planctomycetota;Pla4_lineage;uncultured_bacterium	88.31	0.0011083	0	0	0	0
OTU403	ET703456.1.1490	Bacteria;Bacteroidota;Bacteroidia;Flavobacteriales;Crocinitomicaceae;Fluviicola;uncultured_Fluviicola_sp.	99.76	0	0.00105612	0.0041602	0.00102209	0
OTU4039	ET134062.1.1319	Bacteria;Chloroflexi;Dehalococcoidia;S085;uncultured_bacterium	97.76	0	0	0	0	0.00098733
OTU404	ET431712.1.1474	Bacteria;Bdellovibrionota;Bdellovibrionia;Bdellovibrionales;Bdellovibrionaceae;Bdellovibrio;uncultured_bacterium	99.26	0	0	0	0	0.00098733
OTU4042	ET134603.1.1373	Bacteria;Elusimicrobiota;Lineage_IIc;uncultured_bacterium	97.39	0	0	0.00138673	0	0
OTU405	AB680487.1.1410	Bacteria;Proteobacteria;Alphaproteobacteria;Rhizobiales;Rhizobiaceae;Allorhizobium-Neorhizobium-Pararhizobium-Rhizobium;Rhizobium_sp._NBRC_13714	100	0.0011083	0	0.00693366	0	0.00197467
OTU4059	MHOL01000019.20791.21763	Archaea;Nanoarchaeota;Nanoarchaeia;Woesearchaeales;SCGC_AAA011-D5;Candidatus_Staskawiczbacteria_bacterium_RIFCSPHIGHO2_01_FTLL_34_27	81.57	0.0011083	0	0	0	0
OTU406	CP020892.1158451.1160002	Bacteria;Proteobacteria;Gammaproteobacteria;Pseudomonadales;Pseudomonadaceae;Pseudomonas;Pseudomonas_sp._M30-35	100	0.13742962	0.00211224	0.00693366	0.38226065	0.002962
OTU4061	JQ278816.1.1534	Bacteria;Verrucomicrobiota;Omnitrophia;Omnitrophales;Omnitrophaceae;Candidatus_Omnitrophus;uncultured_bacterium	90.35	0	0.00105612	0	0	0
OTU4066	MHGG01000050.25339.26910	Bacteria;Verrucomicrobiota;Omnitrophia;Omnitrophales;Omnitrophaceae;Candidatus_Omnitrophus;Omnitrophica_WOR_2_bacterium_GWF2_63_9	92.07	0	0	0	0.00102209	0
OTU407	AB722124.1.1353	Bacteria;Patescibacteria;Parcubacteria;TBA9983;uncultured_bacterium	83.59	0	0.00211224	0	0.00102209	0.00098733
OTU408	KF836295.1.1480	Bacteria;Elusimicrobiota;Elusimicrobia;Lineage_IV;uncultured_bacterium	95.17	0	0	0	0	0.00098733
OTU4087	EF190149.1.1478	Bacteria;Bacteroidota;Bacteroidia;Flavobacteriales;Flavobacteriaceae;Flavobacterium;uncultured_bacterium	99.53	0	0	0	0.00102209	0.06516395
OTU409	ET703456.1.1490	Bacteria;Bacteroidota;Bacteroidia;Flavobacteriales;Crocinitomicaceae;Fluviicola;uncultured_Fluviicola_sp.	99.29	0.0011083	0	0.00554693	0.00204417	0.00197467
OTU4094	FPLS01060071.8.1519	Bacteria;Proteobacteria;Gammaproteobacteria;Pseudomonadales;Pseudomonadaceae;Pseudomonas;metagenome	99.77	0	0	0.03605503	0.00102209	0
OTU4097	LJNF01000007.41371.42892	Bacteria;Patescibacteria;Parcubacteria;Candidatus_Yanofskybacteria;Parcubacteria_bacterium_DG_74_2	81.53	0	0	0	0	0.00098733
OTU41	FJ820479.1.1477	Bacteria;Bacteroidota;Bacteroidia;Flavobacteriales;Flavobacteriaceae;Flavobacterium;uncultured_bacterium	100	0.01108303	0.01689796	0.16918127	0.00306626	0.00888599
OTU410	GT559788.1.1398	Bacteria;Proteobacteria;Gammaproteobacteria;Xanthomonadales;Xanthomonadaceae;Silanimonas;uncultured_bacterium	100	0.00221661	0.00105612	0	0.00204417	0.00098733
OTU411	AB360546.1.1550	Bacteria;Firmicutes;Bacilli;Paenibacillales;Paenibacillaceae;Paenibacillus;Paenibacillus_macquariensis_subsp._defensor	99.3	0	0	0.52557133	0.25041139	0.02863264
OTU4113	JF417820.1.1499	Bacteria;Elusimicrobiota;Lineage_IIc;uncultured_bacterium	95.73	0	0.00105612	0	0	0
OTU4114	JQ278816.1.1534	Bacteria;Verrucomicrobiota;Omnitrophia;Omnitrophales;Omnitrophaceae;Candidatus_Omnitrophus;uncultured_bacterium	91.63	0	0.00105612	0	0	0
OTU4115	KJ615120.1.1318	Bacteria;Proteobacteria;Gammaproteobacteria;Beggiatoales;Beggiatoaceae;uncultured;uncultured_bacterium	100	0	0	0	0	0.00098733
OTU4119	LN870718.1.1401	Bacteria;Myxococcota;Polyangia;Blfdi19;uncultured_bacterium	93.13	0.0011083	0	0.00138673	0	0
OTU412	AJ867912.1.1482	Bacteria;Proteobacteria;Gammaproteobacteria;Burkholderiales;Comamonadaceae;Limnohabitans;uncultured_beta_proteobacterium	100	0	0	0.00138673	0.00204417	0
OTU4122	FPLS01051976.2.1398	Bacteria;Proteobacteria;Gammaproteobacteria;PLTA13;metagenome	99.77	0	0.00211224	0.0041602	0	0
OTU413	AJ440981.1.1455	Bacteria;Bacteroidota;Bacteroidia;Flavobacteriales;Flavobacteriaceae;Flavobacterium;Antarctic_bacterium_R-7579	99.53	0.00221661	0.00105612	0.00970712	0.00204417	0.00098733

OTU4133	HM270126.1.1360	Bacteria;Actinobacteriota;Actinobacteria;Propionibacteriales;Nocardioidaceae;Nocardioides;uncultured_bacterium	100	0	0	0.00138673	0	0.00098733
OTU4137	JN656823.1.1488	Bacteria;Bacteroidota;Bacteroidia;Chitinophagales;Chitinophagaceae;Ferruginibacter;uncultured_Bacteroidetes_bacterium	99.76	0	0	0.00138673	0	0
OTU414	AB750589.1.1467	Bacteria;Proteobacteria;Alphaproteobacteria;Sphingomonadales;Sphingomonadaceae;Sphingopyxis;uncultured_bacterium	100	0.00332491	0.00105612	0.00138673	0.00306626	0.08984726
OTU4141	KX172841.1.1419	Bacteria;Proteobacteria;Alphaproteobacteria;Rhodobacterales;Rhodobacteraceae;Limibaculum;uncultured_bacterium	96.52	0	0.00105612	0	0	0
OTU4142	MHGG01000050.25339.26910	Bacteria;Verrucomicrobiota;Omnitrophia;Omnitrophales;Omnitrophaceae;Candidatus_Omnitrophus;Omnitrophica_WOR_2_bacterium_GWF2_63_9	94.87	0	0	0.00138673	0	0
OTU4147	AM501446.1.1382	Bacteria;Proteobacteria;Gammaproteobacteria;Burkholderiales;Comamonadaceae;Roseateles;Roseateles_aquatilis	100	0	0.00105612	0	0.00102209	0
OTU415	MHJF01000011.24156.25754	Bacteria;Patescibacteria;Parcubacteria;Candidatus_Colwellbacteria;Candidatus_Harrisonbacteria_bacterium_RIFCSPHIGHO2_02_FTLL_40_20	87.5	0	0.00211224	0.00138673	0.00204417	0.00197467
OTU4150	AY887010.1.1360	Bacteria;Proteobacteria;Gammaproteobacteria;Enterobacterales;Alteromonadaceae;Rheinheimera;uncultured_gamma_proteobacterium	100	0	0.00211224	0.00138673	0	0
OTU4154	FPLL01006943.13.1527	Bacteria;Proteobacteria;Gammaproteobacteria;Pseudomonadales;Cellvibrionaceae;Cellvibrio;metagenome	99.53	0.0011083	0	0.00138673	0	0.00098733
OTU4159	JQ278884.1.1533	Bacteria;Verrucomicrobiota;Omnitrophia;Omnitrophales;Omnitrophaceae;Candidatus_Omnitrophus;uncultured_bacterium	91.63	0	0	0.00138673	0	0
OTU416	AB022925.1.1450	Bacteria;Firmicutes;Bacilli;Lactobacillales;Lactobacillaceae;Leuconostoc;Leuconostoc_carnosum	100	0	0	0	0.00102209	0
OTU4165	KJ782244.1.1479	Bacteria;Gemmatimonadota;Gemmatimonadetes;Gemmatimonadales;Gemmatimonadaceae;uncultured;uncultured_bacterium	99.75	0	0	0	0	0.00098733
OTU4168	FJ755773.1.1481	Bacteria;Verrucomicrobiota;Omnitrophia;Omnitrophales;Omnitrophaceae;Candidatus_Omnitrophus;uncultured_bacterium	87.82	0	0.00105612	0	0	0
OTU4169	AJ314919.1.1470	Bacteria;Actinobacteriota;Actinobacteria;Corynebacteriales;Corynebacteriaceae;Corynebacterium;Corynebacterium_appendicis	100	0.0011083	0.00211224	0	0	0
OTU417	CP000949.5254580.5256108	Bacteria;Proteobacteria;Gammaproteobacteria;Pseudomonadales;Pseudomonadaceae;Pseudomonas;Pseudomonas_putida_W619	100	0.00332491	0.00105612	0.80014422	0	0.002962
OTU4170	AB022027.1.1407	Bacteria;Firmicutes;Bacilli;Lactobacillales;Carnobacteriaceae;Granulicatella;Abiotrophia_para-adiacens	99.77	0	0	0	0	0.00098733
OTU4178	AF468435.1.1462	Bacteria;Bacteroidota;Bacteroidia;Flavobacteriales;Flavobacteriaceae;Salegentibacter;Arctic_sea_ice_bacterium_ARK9985	100	0	0	0.00138673	0	0
OTU4179	KX172614.1.1403	Bacteria;Patescibacteria;Parcubacteria;Candidatus_Yanofskybacteria;uncultured_bacterium	92.53	0.0011083	0	0	0	0
OTU418	AF493635.1.1300	Bacteria;Bacteroidota;Bacteroidia;Flavobacteriales;Flavobacteriaceae;Flavobacterium;Flavobacterium_sp._EP030	99.76	0.0011083	0	0.51863767	0	0
OTU4180	HE603186.1.1453	Bacteria;Verrucomicrobiota;Omnitrophia;Omnitrophales;Omnitrophaceae;Candidatus_Omnitrophus;uncultured_Firmicutes_bacterium	90.44	0	0	0	0	0.00098733
OTU4181	AB735546.1.1462	Bacteria;Proteobacteria;Gammaproteobacteria;Salinisphaerales;Salinisphaeraceae;Salinisphaera;Salinisphaera_japonica_YTM-1	100	0	0.00105612	0	0	0
OTU419	KX123508.1.1526	Bacteria;Patescibacteria;Parcubacteria;Candidatus_Liptonbacteria;Parcubacteria_group_bacterium_GW2011_GWA1_60_11	87.41	0	0.00105612	0.00138673	0	0.00098733
OTU4194	ET467938.1.1380	Bacteria;Actinobacteriota;Actinobacteria;Micrococcales;Cellulomonadaceae;Oerskovia;uncultured_bacterium	100	0.00221661	0	0.00554693	0.00102209	0.00987333
OTU4195	KY356869.1.921	Archaea;Nanoarchaeota;Nanoarchaeia;Woesearchaeales;GW2011_GWC1_47_15;uncultured_archaeon	87.76	0	0.00105612	0	0	0
OTU4199	JF703465.1.1445	Bacteria;Proteobacteria;Gammaproteobacteria;Burkholderiales;Nitrosomonadaceae;MND1;uncultured_beta_proteobacterium	98.83	0	0	0	0	0.00098733
OTU42	KF091174.1.1346	Bacteria;Actinobacteriota;Actinobacteria;Corynebacteriales;Corynebacteriaceae;Corynebacterium;uncultured_bacterium	99.76	0.01219134	0.01478571	0.00970712	0.01328713	0.00493666
OTU420	CP023276.28082.29594	Bacteria;Proteobacteria;Gammaproteobacteria;Burkholderiales;Burkholderiaceae;Polynucleobacter;Polynucleobacter_difficilis	100	0.00332491	0.00105612	0.01386732	0	0.00098733
OTU4201	AF414584.1.1434	Bacteria;Proteobacteria;Gammaproteobacteria;Burkholderiales;Leeiaceae;Leeia;uncultured_bacterium	98.36	0	0	0	0.00102209	0
OTU4204	KX172614.1.1403	Bacteria;Patescibacteria;Parcubacteria;Candidatus_Yanofskybacteria;uncultured_bacterium	93.54	0.0011083	0	0	0	0
OTU421	AB024288.1.1477	Bacteria;Proteobacteria;Alphaproteobacteria;Sphingomonadales;Sphingomonadaceae;Blastomonas;Blastomonas_natatoria	100	0.0011083	0.00105612	0.02357444	0.00204417	0.00098733
OTU4210	FPLK01002409.9.1522	Bacteria;Proteobacteria;Gammaproteobacteria;Methylococcales;Methylomonadaceae;uncultured;metagenome	99.53	0	0.00105612	0	0	0
OTU4211	KT514538.1.1483	Bacteria;Bacteroidota;Bacteroidia;Sphingobacteriales;Sphingobacteriaceae;Pedobacter;uncultured_bacterium	100	0	0	0	0	0.02172132
OTU4219	MFTV01000001.3080.4539	Bacteria;Patescibacteria;Parcubacteria;TBA9983;Candidatus_Nomurabacteria_bacterium_RIFCSPHIGHO2_02_40_30	96.35	0	0	0.00138673	0	0
OTU422	AY957923.1.1520	Bacteria;Proteobacteria;Gammaproteobacteria;Burkholderiales;Comamonadaceae;Curvibacter;uncultured_bacterium	100	0	0	0	0.00204417	0.00098733
OTU4225	ET216734.1.1344	Bacteria;Proteobacteria;Gammaproteobacteria;Enterobacterales;Erwiniaceae;Pantoea;Pantoea_septica	99.77	0.05541517	0	0	0	0
OTU4226	FJ535179.1.1478	Bacteria;Bacteroidota;Bacteroidia;Flavobacteriales;Flavobacteriaceae;Flavobacterium;uncultured_Flavobacteriia_bacterium	99.52	0.0011083	0	0.20384957	0	0.00098733
OTU4228	KC358393.1.1301	Bacteria;Verrucomicrobiota;Omnitrophia;Omnitrophales;Omnitrophaceae;Candidatus_Omnitrophus;uncultured_bacterium	91.14	0	0	0	0	0.00098733
OTU423	ET621913.1.1477	Bacteria;Firmicutes;Bacilli;Bacillales;Planococcaceae;Psychrobacillus;Paenibacillus_sp._CCBAT_51494	100	0.0011083	0.00211224	0.04021522	0.56317011	0
OTU4230	KC358524.1.1248	Bacteria;Planctomycetota;Pla4_lineage;uncultured_bacterium	93.55	0	0	0	0	0.00098733
OTU4235	JF231993.1.1342	Bacteria;Bacteroidota;Bacteroidia;Flavobacteriales;Flavobacteriaceae;Flavobacterium;uncultured_bacterium	97.39	0	0	0	0	0.00493666
OTU4237	HQ315827.1.1466	Bacteria;Proteobacteria;Gammaproteobacteria;Xanthomonadales;Xanthomonadaceae;Lysobacter;Lysobacter_arseniciresistens_ZS79	98.13	0	0	0	0.06030315	0
OTU424	FJ535179.1.1478	Bacteria;Bacteroidota;Bacteroidia;Flavobacteriales;Flavobacteriaceae;Flavobacterium;uncultured_Flavobacteriia_bacterium	99.29	0	0.00211224	0.52279787	0.00204417	0.002962
OTU4241	MG571573.1.1362	Bacteria;Proteobacteria;Alphaproteobacteria;Rhodobacterales;Rhodobacteraceae;Maribius;Palleronia_sp.	99.75	0	0.00105612	0	0	0
OTU4242	MHGG01000050.25339.26910	Bacteria;Verrucomicrobiota;Omnitrophia;Omnitrophales;Omnitrophaceae;Candidatus_Omnitrophus;Omnitrophica_WOR_2_bacterium_GWF2_63_9	91.75	0	0.00211224	0	0	0
OTU425	AB511013.1.1469	Bacteria;Bdellovibrionota;Oligoflexia;0319-6G20;uncultured_bacterium	100	0.0011083	0	0.00138673	0	0
OTU4250	AB035549.1.1457	Bacteria;Cyanobacteria;Cyanobacteriia;Cyanobacteriales;Microcystaceae;Microcystis_PCC-7914;Microcystis_aeruginosa	98.27	0.0011083	0	0.00138673	0	0
OTU4251	ET335173.1.1507	Bacteria;Acidobacteriota;Acidobacteriae;Subgroup_2;uncultured_bacterium	92.6	0	0.00105612	0.00138673	0	0
OTU4252	KC555001.1.1542	Bacteria;Actinobacteriota;Thermoleophilia;Gaiellales;uncultured;uncultured_bacterium	100	0	0.00105612	0	0	0
OTU4254	MHGG01000050.25339.26910	Bacteria;Verrucomicrobiota;Omnitrophia;Omnitrophales;Omnitrophaceae;Candidatus_Omnitrophus;Omnitrophica_WOR_2_bacterium_GWF2_63_9	92.54	0	0	0	0	0.00098733
OTU4256	JF168675.1.1382	Bacteria;Myxococcota;Polyangia;Haliangiales;Haliangiaceae;Haliangium;uncultured_bacterium	87.15	0	0.00105612	0	0	0
OTU426	CP015604.1047190.1048682	Bacteria;Actinobacteriota;Actinobacteria;Frankiales;Sporichthyaceae;hgcI_clade;Actinobacteria_bacterium_IMCC26103	100	0.00221661	0.00316837	0.01386732	0	0.00098733
OTU4260	DQ450755.1.1317	Bacteria;Proteobacteria;Alphaproteobacteria;Reyranellales;Reyranellaceae;Reyranella;uncultured_Alphaproteobacteria_bacterium	99.75	0	0.00105612	0	0	0
OTU4265	KY999930.1.1441	Bacteria;Proteobacteria;Gammaproteobacteria;Pseudomonadales;Halomonadaceae;Salinicola;Salinicola_sp.	98.36	0	0.00105612	0	0.00102209	0
OTU4266	JF766443.1.1504	Bacteria;Proteobacteria;Gammaproteobacteria;Burkholderiales;Oxalobacteraceae;Janthinobacterium;uncultured_bacterium	98.83	0	0	0	0.00102209	0.01974665
OTU427	HM856434.1.1437	Bacteria;Actinobacteriota;Actinobacteria;Frankiales;Sporichthyaceae;hgcI_clade;uncultured_Actinomycetales_bacterium	100	0	0.00105612	0	0.00102209	0
OTU4270	MF942653.1.1445	Bacteria;Verrucomicrobiota;Omnitrophia;Omnitrophales;Omnitrophaceae;Candidatus_Omnitrophus;uncultured_bacterium	90.42	0	0	0	0.00102209	0
OTU428	FPLK01002537.2.1547	Bacteria;Verrucomicrobiota;Verrucomicrobiae;Pedosphaerales;Pedosphaeraceae;SH3-11;metagenome	99.53	0.0011083	0	0	0	0.00197467
OTU4281	JQ675477.1.1273	Bacteria;SAR324_clade(Marine_group_B);uncultured_bacterium	90.3	0	0.00105612	0	0	0
OTU4283	HM598263.1.1538	Bacteria;Verrucomicrobiota;Omnitrophia;Omnitrophales;Omnitrophaceae;Candidatus_Omnitrophus;uncultured_bacterium	83.18	0.0011083	0	0	0	0
OTU4285	HQ856352.1.1448	Bacteria;Bacteroidota;Kapabacteria;Kapabacteriales;uncultured_bacterium	99.05	0.0011083	0	0	0	0
OTU4287	DQ337066.1.1462	Bacteria;Proteobacteria;Alphaproteobacteria;Paracaedibacterales;Paracaedibacteraceae;uncultured;uncultured_bacterium	99.25	0	0	0.00138673	0	0
OTU429	CP012945.1523999.1525460	Bacteria;Proteobacteria;Alphaproteobacteria;Rhizobiales;Devosiaceae;Devosia;Devosia_sp._A16	100	0	0	0	0.00102209	0.00987333
OTU4297	KP686656.1.1459	Bacteria;Proteobacteria;Gammaproteobacteria;Burkholderiales;Comamonadaceae;Limnohabitans;uncultured_bacterium	99.53	0	0	0.00277346	0	0
OTU43	KF836147.1.1531	Bacteria;Nitrospirota;Leptospirillia;Leptospirillales;Leptospirillaceae;Leptospirillum;uncultured_bacterium	93.22	0.01108303	0.00844898	0.00693366	0.00408835	0.00493666
OTU430	AY922093.1.1372	Bacteria;Patescibacteria;Parcubacteria;uncultured_Parcubacteria_group_bacterium	90.89	0	0.00105612	0	0	0
OTU4302	JF274938.1.1492	Bacteria;Bacteroidota;Bacteroidia;Sphingobacteriales;Sphingobacteriaceae;Pedobacter;Pedobacter_sp._PX8b_S1	100	0.0011083	0	0	0	0
OTU4308	ET800906.1.1499	Bacteria;Proteobacteria;Gammaproteobacteria;Burkholderiales;Sutterellaceae;AAP99;uncultured_bacterium	99.3	0	0	0	0	0.00098733

OTU431	LN870812.1.1394	Bacteria;Proteobacteria;Gammaproteobacteria;Burkholderiales;Nitrosomonadaceae;MND1;uncultured_bacterium	99.06	0.00221661	0.00105612	0	0.00102209	0.00197467
OTU4315	MFVN01000036.3183.4707	Archaea;Nanoarchaeota;Nanoarchaeia;Woesearchaeales;Candidatus_Nomurabacteria_bacterium_RIFCSPLOWO2_02_FTLL_42_17	91.45	0	0	0.00138673	0	0
OTU432	CALI02000005.143.1657	Bacteria;Firmicutes;Clostridia;Peptostreptococcales-Tissierellales;Family_XI;Fenollaria;Fenollaria_massiliensis	100	0.0011083	0	0.00138673	0	0
OTU4324	FPLK01001309.1.1434	Bacteria;Bacteroidota;Bacteroidia;Sphingobacteriales;env.OPS_17;metagenome	100	0.0011083	0	0	0	0
OTU4325	MHGG01000050.25339.26910	Bacteria;Verrucomicrobiota;Omnitrophia;Omnitrophales;Omnitrophaceae;Candidatus_Omnitrophus;Omnitrophica_WOR_2_bacterium_GWF2_63_9	91.14	0	0	0	0.00102209	0
OTU433	HQ703822.1.1430	Bacteria;Bacteroidota;Bacteroidia;Bacteroidales;Prolixibacteraceae;Draconibacterium;uncultured_bacterium	98.34	0.00221661	0	0.00138673	0	0
OTU434	FJ502260.1.1488	Bacteria;Bacteroidota;Bacteroidia;Flavobacteriales;Flavobacteriaceae;Flavobacterium;uncultured_bacterium	100	0.0011083	0	0.00277346	0	0.00098733
OTU4340	AAJM01000140.1744.3228	Bacteria;Firmicutes;Bacilli;Bacillales;Bacillaceae;Bacillus;Bacillus_thuringiensis_serovar_israelensis_ATCC_35646	100	0	0	0.02912137	0.00306626	0.00098733
OTU4341	AB184058.1.1465	Bacteria;Actinobacteriota;Actinobacteria;Streptomycetales;Streptomycetaceae;Streptomyces;Streptomyces_gangtokensis	99.75	0.00443321	0.06864795	0.00277346	0	0
OTU4343	DQ066985.1.1377	Bacteria;Bacteroidota;Bacteroidia;Sphingobacteriales;Lentimicrobiaceae;uncultured_bacterium	92.2	0.0011083	0	0	0	0
OTU4345	MF942639.1.1600	Bacteria;Verrucomicrobiota;Omnitrophia;Omnitrophales;uncultured_bacterium	92.52	0.0011083	0	0	0	0
OTU4349	JQ278884.1.1533	Bacteria;Verrucomicrobiota;Omnitrophia;Omnitrophales;Omnitrophaceae;Candidatus_Omnitrophus;uncultured_bacterium	91.4	0	0.00105612	0.00138673	0	0
OTU4350	HM186999.1.1341	Bacteria;Chloroflexi;Dehalococcoidia;SAR202_clade;uncultured_bacterium	91.32	0	0	0.00138673	0	0
OTU4353	AY676115.1.1389	Bacteria;Proteobacteria;Alphaproteobacteria;Sphingomonadales;Sphingomonadaceae;Altererythrobacter;Altererythrobacter_troitsensis	100	0	0.00105612	0	0	0
OTU4354	JX025753.1.1517	Bacteria;Myxococcota;Polyangia;Haliangiales;Haliangiaceae;Haliangium;uncultured_Myxococcales_bacterium	96.02	0	0	0	0.00102209	0
OTU4364	AJ639827.1.1489	Bacteria;Actinobacteriota;Actinobacteria;Micrococcales;Micrococcaceae;Arthrobacter;Arthrobacter_pigmenti	100	0	0	0	0.00102209	0
OTU4366	KP686877.1.1447	Bacteria;Actinobacteriota;Actinobacteria;PeM15;uncultured_bacterium	100	0.0011083	0.00105612	0.00138673	0	0
OTU437	MF942653.1.1445	Bacteria;Verrucomicrobiota;Omnitrophia;Omnitrophales;Omnitrophaceae;Candidatus_Omnitrophus;uncultured_bacterium	90.19	0	0	0	0.00102209	0
OTU4371	EF659433.1.1519	Bacteria;Actinobacteriota;Actinobacteria;Micrococcales;Microbacteriaceae;Candidatus_Aquiluna;uncultured_bacterium	99.75	0	0	0.00138673	0	0
OTU4377	FJ516974.1.1503	Bacteria;Desulfobacterota;Desulfobulbia;Desulfobulbales;Desulfocapsaceae;uncultured;uncultured_Desulfobulbaceae_bacterium	98.83	0	0.00105612	0	0	0
OTU438	KP686648.1.1428	Bacteria;Patescibacteria;Parcubacteria;uncultured_bacterium	81.82	0	0.00105612	0	0	0
OTU4388	EF373534.1.1420	Bacteria;Actinobacteriota;Actinobacteria;Micrococcales;Microbacteriaceae;Chryseoglobus;Chryseoglobus_frigidaquae	100	0.0011083	0.00105612	0.00138673	0	0
OTU439	HM128588.1.1439	Bacteria;Actinobacteriota;Actinobacteria;Frankiales;Sporichthyaceae;hgcI_clade;uncultured_bacterium	100	0.0011083	0	0	0	0.00098733
OTU4392	LN870758.1.1414	Bacteria;Verrucomicrobiota;Omnitrophia;Omnitrophales;Omnitrophaceae;Candidatus_Omnitrophus;uncultured_bacterium	87.79	0	0	0.00138673	0	0
OTU4394	AB517711.1.1439	Bacteria;Bacteroidota;Bacteroidia;Flavobacteriales;Flavobacteriaceae;Flavobacterium;Flavobacterium_aquatile	100	0	0	0	0	0.05923995
OTU4396	MHGG01000050.25339.26910	Bacteria;Verrucomicrobiota;Omnitrophia;Omnitrophales;Omnitrophaceae;Candidatus_Omnitrophus;Omnitrophica_WOR_2_bacterium_GWF2_63_9	91.86	0.0011083	0	0	0	0
OTU44	AM935276.1.1391	Bacteria;Patescibacteria;Saccharimonadia;Saccharimonadales;uncultured_Candidatus_Saccharibacteria_bacterium	95.04	0.00332491	0.00422449	0.00138673	0.00511044	0
OTU440	KR868710.1.1452	Bacteria;Bacteroidota;Bacteroidia;Flavobacteriales;Flavobacteriaceae;Gramella;Gramella_aquimixticola	95.73	0	0.00105612	0	0	0.00098733
OTU4405	KC358393.1.1301	Bacteria;Verrucomicrobiota;Omnitrophia;Omnitrophales;Omnitrophaceae;Candidatus_Omnitrophus;uncultured_bacterium	91.38	0	0	0	0	0.00098733
OTU441	AM939566.1.1457	Bacteria;Actinobacteriota;Actinobacteria;Micrococcales;Microbacteriaceae;Candidatus_Planktoluna;Candidatus_Planktoluna_difficilis	100	0	0.00105612	0.01109385	0.00204417	0.00098733
OTU4414	HQ224601.1.1426	Bacteria;Proteobacteria;Gammaproteobacteria;Pseudomonadales;Pseudomonadaceae;Pseudomonas;Pseudomonas_sp._SGb186	99.77	0	0	0.07211005	0	0.00098733
OTU442	AKJR01000012.66484.67881	Bacteria;Proteobacteria;Gammaproteobacteria;Pseudomonadales;Pseudomonadaceae;Pseudomonas;Pseudomonas_sp._GM24	100	0.00997473	0.00105612	0.53805192	0.00306626	0.07010061
OTU4423	KY356865.1.937	Archaea;Nanoarchaeota;Nanoarchaeia;Woesearchaeales;GW2011_GWC1_47_15;uncultured_archaeon	83.81	0	0	0.0041602	0	0
OTU4428	JQ195940.1.1350	Bacteria;Bacteroidota;Bacteroidia;Flavobacteriales;Crocinitomicaceae;Fluviicola;uncultured_bacterium	99.29	0	0	0.00693366	0	0
OTU443	HQ436493.1.1435	Bacteria;Proteobacteria;Alphaproteobacteria;Sphingomonadales;Sphingomonadaceae;Sphingorhabdus;Sphingorhabdus_wooponensis	100	0.0011083	0.00211224	0.01802751	0.00408835	0
OTU4432	KF836295.1.1480	Bacteria;Elusimicrobiota;Elusimicrobia;Lineage_IV;uncultured_bacterium	94.44	0	0.00105612	0	0	0
OTU4437	KF975551.1.1519	Bacteria;Acidobacteriota;Blastocatellia;Blastocatellales;Blastocatellaceae;Blastocatella;uncultured_bacterium	99	0	0	0.00138673	0	0
OTU444	AF493635.1.1300	Bacteria;Bacteroidota;Bacteroidia;Flavobacteriales;Flavobacteriaceae;Flavobacterium;Flavobacterium_sp._EP030	99.53	0.00221661	0.00105612	0.50199689	0.00408835	0.00493666
OTU4440	MG710486.1.1505	Bacteria;Cyanobacteria;Cyanobacteriia;uncultured;cyanobacterium_I_35_MF_cl1	99.51	0.00886643	0	0	0	0
OTU445	JQ994351.1.1479	Bacteria;Actinobacteriota;Acidimicrobiia;Microtrichales;Ilumatobacteraceae;CL500-29_marine_group;uncultured_Acidimicrobiales_bacterium	100	0.0011083	0.00105612	0.00138673	0.00204417	0
OTU4455	KC424697.1.1525	Bacteria;Bacteroidota;Kryptonia;Kryptoniales;BSV26;uncultured_bacterium	91.69	0.0011083	0	0	0	0
OTU4459	DQ407745.1.1462	Bacteria;Proteobacteria;Alphaproteobacteria;Rickettsiales;Rickettsiaceae;Rickettsia;primary_endosymbiont_of_Liposcelis_bostrychophila_(host_pop._CQXM)	100	0	0.00105612	0	0	0
OTU446	FPLS01017925.13.1507	Bacteria;Elusimicrobiota;Elusimicrobia;Lineage_IV;metagenome	98.31	0	0	0	0.00102209	0
OTU4462	KT283080.1.1499	Bacteria;Proteobacteria;Gammaproteobacteria;Burkholderiales;Nitrosomonadaceae;MND1;uncultured_bacterium	98.83	0	0.00105612	0	0	0
OTU4466	KJ013397.1.1514	Bacteria;Actinobacteriota;Actinobacteria;Propionibacteriales;Propionibacteriaceae;Microlunatus;uncultured_bacterium	100	0	0	0	0.00102209	0
OTU4468	FJ484653.1.1356	Bacteria;Verrucomicrobiota;Omnitrophia;Omnitrophales;Omnitrophaceae;Candidatus_Omnitrophus;uncultured_Omnitrophica_bacterium	80	0	0.00105612	0	0	0
OTU447	AB362299.1.1469	Bacteria;Proteobacteria;Gammaproteobacteria;Pseudomonadales;Moraxellaceae;Acinetobacter;Acinetobacter_sp._JAM-GA0301	100	0.0011083	0.00211224	0.00277346	0	0
OTU4471	DQ404728.1.1548	Bacteria;Verrucomicrobiota;Omnitrophia;Omnitrophales;Omnitrophaceae;Candidatus_Omnitrophus;uncultured_bacterium	98.82	0	0	0	0	0.00098733
OTU4473	FPLS01056716.36.1538	Bacteria;Actinobacteriota;Thermoleophilia;Solirubrobacterales;Solirubrobacteraceae;Conexibacter;metagenome	98.36	0.0011083	0	0	0	0
OTU4476	EF018499.1.1391	Bacteria;Verrucomicrobiota;Omnitrophia;Omnitrophales;uncultured_bacterium	95.54	0	0	0	0.00102209	0
OTU4480	ET512011.1.1361	Bacteria;Proteobacteria;Gammaproteobacteria;Pseudomonadales;Pseudomonadaceae;Pseudomonas;uncultured_bacterium	95.08	0.0011083	0.04118877	0	0.00102209	0
OTU4485	LN555033.1.1358	Bacteria;Elusimicrobiota;Elusimicrobia;MVP-88;uncultured_delta_proteobacterium	94.08	0.0011083	0	0	0	0
OTU4489	EF020157.1.1362	Bacteria;Patescibacteria;Parcubacteria;uncultured_bacterium	86.45	0	0.00105612	0	0	0
OTU449	JN674641.1.1390	Bacteria;Bacteroidota;Bacteroidia;Chitinophagales;Chitinophagaceae;Sediminibacterium;Sediminibacterium_goheungense	99.53	0.00332491	0	0	0	0
OTU45	JQ923566.1.1499	Bacteria;Nitrospirota;Nitrospiria;Nitrospirales;Nitrospiraceae;Nitrospira;uncultured_bacterium	100	0.0011083	0.00739286	0.00970712	0.00715461	0.00197467
OTU4506	FR667461.1.1428	Bacteria;NB1-j;uncultured_bacterium	99.53	0	0	0.00138673	0	0.00098733
OTU4510	GEGG01003054.119.1620	Bacteria;Actinobacteriota;Actinobacteria;Streptomycetales;Streptomycetaceae;E1B-B3-114;Rhacophorus_dennysi	98.28	0.00664982	0.01372959	0.02912137	0.26472061	0
OTU4517	KX123556.1.1493	Bacteria;Patescibacteria;ABY1;Candidatus_Thrbacteria;Candidatus_Thrbacteria_bacterium_GW2011_GWE2_46_68	87.25	0	0	0	0	0.00098733
OTU452	HQ674906.1.1487	Bacteria;Planctomycetota;Pla4_lineage;uncultured_planctomycete	98.26	0.0011083	0	0	0.00102209	0.00098733
OTU453	AB537169.1.1458	Bacteria;Actinobacteriota;Actinobacteria;Micrococcales;Dermabacteraceae;Brachybacterium;Brachybacterium_conglomeratum	100	0.00221661	0	0	0.00102209	0
OTU4538	KX123391.1.2305	Bacteria;Patescibacteria;Parcubacteria;Candidatus_Jorgensenbacteria;Candidatus_Azambacteria_bacterium_GW2011_GWB1_42_17	86.86	0	0.00211224	0	0	0
OTU4539	JF266405.1.1299	Bacteria;Patescibacteria;Parcubacteria;Candidatus_Kaiserbacteria;uncultured_bacterium	86.75	0	0.00105612	0	0	0
OTU454	FJ827859.1.1493	Bacteria;Actinobacteriota;Acidimicrobiia;Microtrichales;Ilumatobacteraceae;CL500-29_marine_group;uncultured_actinobacterium	99.5	0	0.00105612	0	0.00102209	0
OTU4544	EF032777.1.1476	Bacteria;Verrucomicrobiota;Omnitrophia;Omnitrophales;Omnitrophaceae;Candidatus_Omnitrophus;uncultured_bacterium	85.92	0	0	0	0.00102209	0
OTU4549	JQ278784.1.1466	Bacteria;Elusimicrobiota;Elusimicrobia;Lineage_IV;uncultured_bacterium	89.08	0.0011083	0	0	0	0
OTU455	JF697461.1.1494	Bacteria;Proteobacteria;Gammaproteobacteria;Burkholderiales;Comamonadaceae;Limnohabitans;uncultured_bacterium	100	0	0.00105612	0.00138673	0	0
OTU4551	JQ278884.1.1533	Bacteria;Verrucomicrobiota;Omnitrophia;Omnitrophales;Omnitrophaceae;Candidatus_Omnitrophus;uncultured_bacterium	89.3	0	0.00105612	0	0	0

OTU4557	AB045094.1.1500	Bacteria;Firmicutes;Bacilli;Paenibacillales;Paenibacillaceae;Paenibacillus;Paenibacillus_pabuli	99.53	0	0	0.04992234	0.09709829	0
OTU456	AY904033.1.1437	Bacteria;Firmicutes;Bacilli;Bacillales;Bacillaceae;Bacillus;Bacillus_idriensis	100	0	0	0.00138673	0.00204417	0.002962
OTU4561	KT868099.1.1468	Bacteria;Patescibacteria;Parcubacteria;Candidatus_Yanofskybacteria;uncultured_bacterium	93.89	0	0.00105612	0	0	0
OTU4568	KF836295.1.1480	Bacteria;Elusimicrobiota;Elusimicrobia;Lineage_IV;uncultured_bacterium	94.93	0	0.00105612	0	0	0
OTU457	FPLP01003961.16.1460	Bacteria;Proteobacteria;Alphaproteobacteria;Rhodobacterales;Rhodobacteraceae;uncultured;metagenome	99.5	0	0	0.00138673	0	0
OTU4573	ET133858.1.1327	Bacteria;Proteobacteria;Gammaproteobacteria;Burkholderiales;Nitrosomonadaceae;MND1;uncultured_bacterium	99.06	0	0.00105612	0.00138673	0	0.00098733
OTU4574	HQ120666.1.1506	Bacteria;Bdellovibrionota;Oligoflexia;Oligoflexales;Oligoflexaceae;uncultured;uncultured_bacterium	94.39	0.0011083	0	0	0	0
OTU4582	KC831438.1.1452	Bacteria;Proteobacteria;Alphaproteobacteria;Acetobacterales;Acetobacteraceae;Rhodovarius;uncultured_bacterium	99	0	0	0.00277346	0	0
OTU459	HM856568.1.1440	Bacteria;Actinobacteriota;Actinobacteria;Micrococcales;Microbacteriaceae;Rhodoluna;uncultured_Microbacteriaceae_bacterium	100	0.0011083	0.00105612	0.01386732	0.00102209	0
OTU4596	HM187224.1.1501	Bacteria;Myxococcota;Polyangia;Haliangiales;Haliangiaceae;Haliangium;uncultured_bacterium	98.12	0	0.00105612	0	0	0
OTU4598	AAOH01000015.31873.33412	Bacteria;Proteobacteria;Gammaproteobacteria;Enterobacterales;Pseudoalteromonadaceae;Pseudoalteromonas;Pseudoalteromonas_tunicata_D2	100	0	0	0	0.00102209	0
OTU46	FJ037619.1.1470	Bacteria;Campylobacterota;Campylobacteria;Campylobacterales;Sulfurimonadaceae;Sulfurimonas;uncultured_bacterium	98.01	0.01773285	0.00316837	0.0041602	0.00715461	0.00691133
OTU460	KY356869.1.921	Archaea;Nanoarchaeota;Nanoarchaeia;Woesearchaeales;GW2011_GWC1_47_15;uncultured_archaeon	85.8	0	0	0.00138673	0	0
OTU4606	AB245348.1.1434	Bacteria;Proteobacteria;Alphaproteobacteria;Sphingomonadales;Sphingomonadaceae;uncultured;Sphingomonadaceae_bacterium_Gsoil_690	100	0.00664982	0	0	0.00204417	0
OTU4607	EF190149.1.1478	Bacteria;Bacteroidota;Bacteroidia;Flavobacteriales;Flavobacteriaceae;Flavobacterium;uncultured_bacterium	99.29	0.00221661	0	0	0	0.08688526
OTU4608	FJ900575.1.1514	Bacteria;Chloroflexi;Dehalococcoidia;SAR202_clade;uncultured_bacterium	87.97	0.0011083	0.00105612	0	0	0
OTU461	KP151153.1.1498	Bacteria;Proteobacteria;Gammaproteobacteria;Beggiatoales;Beggiatoaceae;uncultured;uncultured_bacterium	93.68	0.00221661	0	0	0	0
OTU4617	GQ060239.1.1340	Bacteria;Actinobacteriota;Actinobacteria;Corynebacteriales;Corynebacteriaceae;Corynebacterium;uncultured_bacterium	99.76	0	0	0	0	0.00098733
OTU462	ET801876.1.1485	Bacteria;Actinobacteriota;Acidimicrobiia;Microtrichales;Ilumatobacteraceae;CL500-29_marine_group;uncultured_bacterium	100	0.0011083	0	0	0	0.002962
OTU4620	AHAE01000085.45441.46976	Bacteria;Actinobacteriota;Actinobacteria;Corynebacteriales;Corynebacteriaceae;Turicella;Turicella_otitidis_ATCC_51513	99.52	0.0011083	0.00105612	0	0	0
OTU4623	JQ770028.1.1463	Bacteria;Proteobacteria;Alphaproteobacteria;Paracaedibacterales;Paracaedibacteraceae;Candidatus_Captivus;uncultured_bacterium	99.25	0	0	0	0	0.00098733
OTU4626	FJ592705.1.1414	Bacteria;Verrucomicrobiota;Verrucomicrobiae;Opitutales;Opitutaceae;Diplosphaera;uncultured_bacterium	99.53	0.00221661	0	0.00138673	0	0
OTU463	MHGG01000050.25339.26910	Bacteria;Verrucomicrobiota;Omnitrophia;Omnitrophales;Omnitrophaceae;Candidatus_Omnitrophus;Omnitrophica_WOR_2_bacterium_GWF2_63_9	92.07	0	0	0	0	0.00098733
OTU4631	KX173051.1.1479	Bacteria;Planctomycetota;BD7-11;uncultured_bacterium	88.65	0	0	0	0.00102209	0
OTU464	CP010350.612046.613590	Bacteria;Proteobacteria;Gammaproteobacteria;Pseudomonadales;Moraxellaceae;Acinetobacter;Acinetobacter_johnsonii_XBB1	100	0.00221661	0.00316837	0.00277346	0	0.00098733
OTU4645	MF942642.1.1390	Bacteria;Verrucomicrobiota;Omnitrophia;Omnitrophales;Omnitrophaceae;Candidatus_Omnitrophus;uncultured_bacterium	89.51	0	0	0.00138673	0	0
OTU465	LCOT01000026.4898.6395	Bacteria;Patescibacteria;Parcubacteria;Candidatus_Jorgensenbacteria;Parcubacteria_group_bacterium_GW2011_GWA2_47_8b	83.46	0	0	0	0.00102209	0.002962
OTU4655	AJ609630.1.1468	Bacteria;Actinobacteriota;Actinobacteria;Micrococcales;Micrococcaceae;Glutamicibacter;Glutamicibacter_bergerei	98.77	0.00221661	0.00844898	0	0.36386308	0
OTU4658	FR847879.1.1498	Bacteria;Proteobacteria;Gammaproteobacteria;Beggiatoales;Beggiatoaceae;uncultured;uncultured_bacterium	95.08	0.0011083	0.00105612	0	0	0
OTU466	AM991204.1.1392	Bacteria;Patescibacteria;Parcubacteria;Candidatus_Giovannonibacteria;uncultured_bacterium	78.87	0	0.00105612	0	0	0
OTU4660	MHGG01000050.25339.26910	Bacteria;Verrucomicrobiota;Omnitrophia;Omnitrophales;Omnitrophaceae;Candidatus_Omnitrophus;Omnitrophica_WOR_2_bacterium_GWF2_63_9	92.07	0	0	0	0.00102209	0
OTU4664	DQ375559.1.1484	Bacteria;Firmicutes;Bacilli;Bacillales;Planococcaceae;Chryseomicrobium;Planococcus_sp._KRPC10y	99.77	0	0	0	0.00102209	0
OTU4665	AJ440975.1.1492	Bacteria;Proteobacteria;Gammaproteobacteria;Enterobacterales;Alteromonadaceae;Paraglaciecola;Antarctic_bacterium_R-11381	97.66	0.0011083	0	0	0	0
OTU4669	AF394173.1.1542	Bacteria;Firmicutes;Bacilli;Bacillales;Planococcaceae;Planococcus;blackwater_bioreactor_bacterium_BW21	100	0	0	0	0.07359029	0.00098733
OTU467	JN392913.1.1480	Bacteria;Bacteroidota;Bacteroidia;Flavobacteriales;Flavobacteriaceae;Flavobacterium;uncultured_bacterium	96.45	0.00221661	0.00105612	0	0	0
OTU4673	AJ519620.1.1294	Bacteria;Nitrospirota;Nitrospiria;Nitrospirales;Nitrospiraceae;Nitrospira;uncultured_bacterium	99.28	0	0	0	0.00102209	0
OTU4675	AY180102.1.1430	Bacteria;Proteobacteria;Alphaproteobacteria;Rhodobacterales;Rhodobacteraceae;Sulfitobacter;Sulfitobacter_dubius	100	0.0011083	0	0	0.00102209	0
OTU4677	AJ518792.1.1313	Bacteria;Myxococcota;Polyangia;Haliangiales;Haliangiaceae;Haliangium;uncultured_delta_proteobacterium	98.36	0	0	0	0.00102209	0
OTU468	KM410840.1.1391	Bacteria;Campylobacterota;Campylobacteria;Campylobacterales;Sulfurovaceae;Sulfurovum;uncultured_prokaryote	100	0.0011083	0.00105612	0	0	0
OTU4680	JF178037.1.1312	Bacteria;Proteobacteria;Alphaproteobacteria;Acetobacterales;Acetobacteraceae;Craurococcus-Caldovatus;uncultured_bacterium	99.75	0	0	0	0.00102209	0
OTU4684	KJ566431.1.984	Archaea;Nanoarchaeota;Nanoarchaeia;Woesearchaeales;GW2011;AR20;uncultured_euryarchaeote	84.14	0	0	0	0	0.00098733
OTU4686	HM129740.1.1447	Bacteria;Gemmatimonadota;Gemmatimonadetes;Gemmatimonadales;Gemmatimonadaceae;Gemmatimonas;uncultured_bacterium	99.52	0	0	0.00277346	0	0
OTU469	KR537251.1.1498	Bacteria;Bacteroidota;Rhodothermia;Balneolales;Balneolaceae;CK06-06-Mud-MAS4B-21;uncultured_bacterium	93.13	0	0	0	0.00102209	0
OTU4690	KX172614.1.1403	Bacteria;Patescibacteria;Parcubacteria;Candidatus_Yanofskybacteria;uncultured_bacterium	91.56	0.0011083	0	0	0	0
OTU47	HQ178923.1.1438	Bacteria;Bacteroidota;Bacteroidia;Flavobacteriales;Flavobacteriaceae;Flavobacterium;uncultured_bacterium	100	0.00775812	0.01056122	0.06240293	0.00511044	0.01184799
OTU470	FN870313.1.1549	Bacteria;Verrucomicrobiota;Omnitrophia;Omnitrophales;Omnitrophaceae;Candidatus_Omnitrophus;uncultured_bacterium	95.98	0.0011083	0	0	0	0
OTU4701	KP145919.1.1262	Bacteria;Actinobacteriota;Actinobacteria;Frankiales;Geodermatophilaceae;Geodermatophilus;Geodermatophilus_sp._SBT_350	100	0	0.00105612	0	0	0.00098733
OTU4707	AJ316319.1.1485	Bacteria;Actinobacteriota;Actinobacteria;Micrococcales;Intrasporangiaceae;uncultured;uncultured_bacterium	100	0	0	0.00277346	0	0
OTU471	JN613471.1.1470	Bacteria;Firmicutes;Bacilli;Bacillales;Planococcaceae;Psychrobacillus;Bacillus_sp._O-NR6	100	0	0	0.00832039	0.45380676	0
OTU472	KY356869.1.921	Archaea;Nanoarchaeota;Nanoarchaeia;Woesearchaeales;GW2011_GWC1_47_15;uncultured_archaeon	87.17	0	0.00316837	0	0	0
OTU4721	KC990424.1.1439	Bacteria;Patescibacteria;Parcubacteria;TBA9983;uncultured_Parcubacteria_group_bacterium	80.99	0	0.00105612	0	0	0
OTU4722	KC255280.1.1485	Bacteria;Bacteroidota;Bacteroidia;Cytophagales;Microscillaceae;Hassallia;uncultured_bacterium	100	0	0.00211224	0.00138673	0	0
OTU4728	AB930763.1.1448	Bacteria;Bacteroidota;Bacteroidia;Flavobacteriales;Crocinitomicaceae;Fluviicola;uncultured_bacterium	98.1	0	0	0.00277346	0	0
OTU473	FPLP01010844.16.1501	Bacteria;WPS-2;metagenome	99.75	0	0	0.00138673	0.00102209	0
OTU4732	LC124634.1.1453	Bacteria;Proteobacteria;Gammaproteobacteria;Acidiferrobacterales;Acidiferrobacteraceae;Sulfurifustis;uncultured_bacterium	99.53	0.0011083	0	0	0	0
OTU4735	KC604492.1.913	Archaea;Aenigmarchaeota;Aenigmarchaeia;Aenigmarchaeales;uncultured_archaeon	88.29	0.0011083	0	0	0	0
OTU4738	FQ658775.1.1285	Bacteria;Proteobacteria;Alphaproteobacteria;Caulobacterales;Hyphomonadaceae;SWB02;uncultured_soil_bacterium	100	0	0.00316837	0.00277346	0	0.002962
OTU474	KX123338.1.3317	Archaea;Thermoplasmatota;Thermoplasmata;uncultured;Candidatus_Amesbacteria_bacterium_GW2011_GWC1_47_15	87.11	0	0.00105612	0	0	0
OTU4747	AF507700.1.1456	Bacteria;Chloroflexi;Dehalococcoidia;SAR202_clade;uncultured_soil_bacterium	94.04	0	0.00105612	0	0	0
OTU475	JN119227.1.1459	Bacteria;Planctomycetota;Phycisphaerae;Phycisphaerales;Phycisphaeraceae;CL500-3;uncultured_marine_bacterium	91.58	0.0011083	0	0	0	0.00098733
OTU4751	AJ697701.1.1474	Bacteria;Bacteroidota;Bacteroidia;Flavobacteriales;Crocinitomicaceae;Fluviicola;uncultured_Sphingobacteriales_bacterium	99.53	0	0	0.0041602	0.00102209	0.00098733
OTU4754	ET234319.1.1490	Bacteria;Actinobacteriota;Actinobacteria;Corynebacteriales;Mycobacteriaceae;Mycobacterium;uncultured_bacterium	100	0	0.00211224	0.00138673	0	0
OTU4756	KY194098.1.1393	Bacteria;Chloroflexi;Dehalococcoidia;SAR202_clade;uncultured_bacterium	94.42	0	0.00105612	0	0	0
OTU4764	JQ278884.1.1533	Bacteria;Verrucomicrobiota;Omnitrophia;Omnitrophales;Omnitrophaceae;Candidatus_Omnitrophus;uncultured_bacterium	97.21	0	0	0.00138673	0	0
OTU4767	KC247342.1.1435	Bacteria;Bacteroidota;Bacteroidia;Flavobacteriales;Flavobacteriaceae;Flavobacterium;Flavobacterium_ahnfeltiae	100	0	0	0.00277346	0	0
OTU477	ET801403.1.1483	Bacteria;Bacteroidota;Bacteroidia;Flavobacteriales;Cryomorphaceae;uncultured;uncultured_bacterium	100	0.0011083	0	0	0	0
OTU4775	FJ750462.1.1407	Bacteria;Proteobacteria;Gammaproteobacteria;Xanthomonadales;Xanthomonadaceae;Luteimonas;Luteimonas_sp._EM0590	99.77	0.00332491	0	0.00138673	0	0

OTU478	KF836147.1.1531	Bacteria;Nitrospirota;Leptospirillia;Leptospirillales;Leptospirillaceae;Leptospirillum;uncultured_bacterium	92.06	0	0.00211224	0.00277346	0.00102209	0.00098733
OTU4784	JF189188.1.1374	Bacteria;Bdellovibrionota;Bdellovibrionia;Bdellovibrionales;Bdellovibrionaceae;Bdellovibrio;uncultured_bacterium	91.12	0	0	0.00138673	0	0
OTU4788	KY356876.1.911	Archaea;Nanoarchaeota;Nanoarchaeia;Woesearchaeales;GW2011_GWC1_47_15;uncultured_archaeon	87.6	0	0.00105612	0	0	0
OTU4789	FPLK01002459.43.1533	Bacteria;Bacteroidota;Bacteroidia;Chitinophagales;Chitinophagaceae;Dinghuibacter;metagenome	99.76	0.0011083	0	0	0	0
OTU4799	AB546308.1.1482	Bacteria;Actinobacteriota;Actinobacteria;Micrococcales;Microbacteriaceae;Agromyces;Agromyces_iriomotensis	100	0.0011083	0	0	0	0
OTU48	JF830152.1.1482	Bacteria;Actinobacteriota;Actinobacteria;Micrococcales;Microbacteriaceae;Rhodoluna;uncultured_bacterium	99.75	0.01329964	0.01372959	0.00970712	0.01533131	0.00493666
OTU480	MEPS01000173.1634.3193	Bacteria;Bdellovibrionota;Oligoflexia;0319-6G20;Bdellovibrionales_bacterium_GWC1_52_8	95.09	0.00221661	0.00105612	0	0	0
OTU4800	AB475013.1.1459	Bacteria;Proteobacteria;Alphaproteobacteria;Rhodobacterales;Rhodobacteraceae;Rhodobacter;uncultured_Alphaproteobacteria_bacterium	99.5	0	0	0	0	0.00098733
OTU4806	MGSW01000083.11045.12608	Bacteria;Myxococcota;bacteriap25;Deltaproteobacteria_bacterium_RIFOXYA2_FTLL_55_11	96.26	0.0011083	0	0	0	0
OTU481	JX566623.1.1532	Bacteria;Proteobacteria;Gammaproteobacteria;Xanthomonadales;Xanthomonadaceae;Stenotrophomonas;Stenotrophomonas_sp._5099	100	0	0.00105612	0	0	0.00098733
OTU4817	ET834800.1.1445	Bacteria;Bacteroidota;Bacteroidia;Cytophagales;Microscillaceae;OLB12;uncultured_bacterium	100	0	0	0.00138673	0	0
OTU4818	KF851141.1.1500	Bacteria;Proteobacteria;Gammaproteobacteria;Pseudomonadales;Pseudomonadaceae;Pseudomonas;uncultured_Pseudomonas_sp.	99.77	0.00332491	0	0	0.00204417	0.09675859
OTU482	AB600460.1.1408	Archaea;Aenigmarchaeota;Aenigmarchaeia;Aenigmarchaeales;uncultured_archaeon	83.51	0	0	0	0	0.00098733
OTU4822	ET375199.1.1201	Bacteria;Actinobacteriota;Acidimicrobiia;Microtrichales;Ilumatobacteraceae;Ilumatobacter;uncultured_actinobacterium	100	0	0	0	0.00102209	0
OTU4826	HM217066.1.1396	Bacteria;Cyanobacteria;Cyanobacteriia;Phormidesmiales;Nodosilineaceae;Nodosilinea_PCC-7104;Leptolyngbya_sp._LEGE_07312	99.5	0	0	0.0041602	0	0
OTU4829	ET134248.1.1391	Bacteria;Chloroflexi;Dehalococcoidia;SAR202_clade;uncultured_bacterium	97.27	0	0	0	0.00102209	0
OTU483	ET801391.1.1417	Bacteria;Proteobacteria;Gammaproteobacteria;Burkholderiales;Methylophilaceae;OM43_clade;uncultured_bacterium	99.77	0.00221661	0.00211224	0.02080098	0	0
OTU4835	JN409061.1.1445	Bacteria;Bacteroidota;Bacteroidia;Cytophagales;Microscillaceae;Ohtaekwangia;uncultured_Bacteroidetes_bacterium	97.86	0.00221661	0	0	0	0
OTU4836	AF493648.1.1297	Bacteria;Bacteroidota;Bacteroidia;Flavobacteriales;Flavobacteriaceae;Flavobacterium;Flavobacterium_sp._EP100	100	0	0	0	0	0.05430329
OTU484	EF471696.1.1463	Bacteria;Actinobacteriota;Acidimicrobiia;Microtrichales;Ilumatobacteraceae;CL500-29_marine_group;uncultured_actinobacterium	100	0	0.00105612	0	0	0.00098733
OTU4840	JN983564.1.1450	Bacteria;Proteobacteria;Gammaproteobacteria;Thiotrichales;Thiotrichaceae;Thiothrix;uncultured_Thiothrix_sp.	100	0	0.00105612	0	0	0
OTU4842	AM935472.1.1379	Bacteria;Acidobacteriota;Subgroup_22;uncultured_Acidobacteria_bacterium	98.36	0	0.00105612	0	0	0.00098733
OTU4849	KJ566445.1.975	Archaea;Nanoarchaeota;Nanoarchaeia;Woesearchaeales;uncultured_euryarchaeote	80.85	0	0.00105612	0	0	0
OTU485	KY356865.1.937	Archaea;Nanoarchaeota;Nanoarchaeia;Woesearchaeales;GW2011_GWC1_47_15;uncultured_archaeon	89.42	0	0.00211224	0	0	0
OTU4850	GT208448.1.1503	Bacteria;Proteobacteria;Gammaproteobacteria;Burkholderiales;Gallionellaceae;Gallionella;uncultured_prokaryote	99.77	0	0.00105612	0	0	0
OTU4851	FPLS01005173.1.1333	Bacteria;Bacteroidota;Bacteroidia;Sphingobacteriales;Sphingobacteriaceae;Pedobacter;metagenome	100	0.04322383	0	0	0	0
OTU4854	HM129231.1.1461	Bacteria;Verrucomicrobiota;Verrucomicrobiae;Verrucomicrobiales;Rubritaleaceae;Luteolibacter;uncultured_bacterium	99.53	0	0.00105612	0	0	0
OTU4858	AF320989.1.1452	Bacteria;Proteobacteria;Gammaproteobacteria;Pseudomonadales;Pseudomonadaceae;Pseudomonas;Pseudomonas_tolaasii	98.36	0.00886643	0.06653571	0	0.00306626	0
OTU486	KC886746.1.1470	Bacteria;Bacteroidota;Bacteroidia;Flavobacteriales;Flavobacteriaceae;Flavobacterium;uncultured_Flavobacterium_sp.	100	0.0011083	0	0.00277346	0	0.00197467
OTU487	KT841576.1.1400	Bacteria;Cyanobacteria;Cyanobacteriia;Synechococcales;Cyanobiaceae;Cyanobium_PCC-6307;uncultured_Synechococcus_sp.	99.75	0	0	0.00138673	0.00102209	0
OTU4871	AB252933.1.1456	Bacteria;Proteobacteria;Alphaproteobacteria;Rhizobiales;Rhizobiales_Incertae_Sedis;uncultured;uncultured_Alphaproteobacteria_bacterium	100	0	0.00105612	0.00138673	0.00102209	0.00098733
OTU4872	KC331491.1.1519	Bacteria;Myxococcota;Myxococcia;Myxococcales;Myxococcaceae;P3OB-42;uncultured_bacterium	90.89	0.0011083	0	0	0	0
OTU4876	FPLS01034413.21.1485	Bacteria;Proteobacteria;Alphaproteobacteria;Rhizobiales;Rhizobiaceae;Allorhizobium-Neorhizobium-Pararhizobium-Rhizobium;metagenome	99.5	0	0	0	0.00102209	0
OTU488	KF964419.1.931	Archaea;Nanoarchaeota;Nanoarchaeia;Woesearchaeales;uncultured_archaeon	91.38	0	0.00105612	0	0	0
OTU4881	HM066571.1.1533	Bacteria;Bdellovibrionota;Oligoflexia;0319-6G20;uncultured_bacterium	92.74	0	0	0	0	0.00098733
OTU4891	JQ716273.1.1493	Bacteria;Bacteroidota;Bacteroidia;Flavobacteriales;Flavobacteriaceae;Marixanthomonas;uncultured_bacterium	100	0	0.00105612	0	0	0
OTU4895	KY356869.1.921	Archaea;Nanoarchaeota;Nanoarchaeia;Woesearchaeales;GW2011_GWC1_47_15;uncultured_archaeon	86.88	0	0	0	0.00102209	0
OTU4899	FPLL01005049.15.1467	Bacteria;Proteobacteria;Alphaproteobacteria;Rickettsiales;SM2D12;metagenome	97.26	0	0	0.00277346	0	0
OTU49	AB578881.1.1391	Bacteria;Proteobacteria;Alphaproteobacteria;Rhodobacterales;Rhodobacteraceae;Rhodobacter;alpha_proteobacterium_S-18	100	0.01440794	0.00844898	0.01941424	0.01022087	0.00394933
OTU490	MHGG01000050.25339.26910	Bacteria;Verrucomicrobiota;Omnitrophia;Omnitrophales;Omnitrophaceae;Candidatus_Omnitrophus;Omnitrophica_WOR_2_bacterium_GWF2_63_9	91.84	0	0.00105612	0	0	0
OTU4900	KC358519.1.1286	Bacteria;Bdellovibrionota;Oligoflexia;0319-6G20;uncultured_bacterium	93.69	0	0.00105612	0	0	0
OTU4902	KX123515.1.1510	Bacteria;Patescibacteria;Parcubacteria;Candidatus_Wolfebacteria;Candidatus_Wolfebacteria_bacterium_GW2011_GWA2_42_10	83.69	0	0	0.00138673	0	0
OTU4907	FLOH01001871.338.1831	Bacteria;Campylobacterota;Campylobacteria;Campylobacterales;Sulfurimonadaceae;Sulfurimonas;marine_metagenome	98.76	0	0	0	0.00102209	0
OTU4908	HE819389.1.1362	Bacteria;Proteobacteria;Gammaproteobacteria;Burkholderiales;Chromobacteriaceae;Vogesella;Vogesella_alkaliphila	93.68	0	0	0	0.00102209	0
OTU491	KC886756.1.1485	Bacteria;Bacteroidota;Bacteroidia;Flavobacteriales;Crocinitomicaceae;Fluviicola;uncultured_Cryomorphaceae_bacterium	99.53	0	0.00105612	0	0	0
OTU4913	HM186410.1.1344	Bacteria;Planctomycetota;Pla4_lineage;uncultured_bacterium	99.75	0	0	0	0	0.00098733
OTU492	JF697410.1.1489	Bacteria;Bacteroidota;Bacteroidia;Flavobacteriales;Crocinitomicaceae;Fluviicola;uncultured_bacterium	100	0	0.00105612	0.00277346	0	0.00197467
OTU4921	AB769482.1.1491	Bacteria;Firmicutes;Bacilli;Bacillales;Marinococcaceae;Marinococcus;Marinococcus_halotolerans	100	0	0.00105612	0	0	0
OTU493	JQ278914.1.1501	Bacteria;Acidobacteriota;Holophagae;Holophagales;Holophagaceae;Geothrix;uncultured_Geothrix_sp.	99.3	0.0011083	0	0.00138673	0.00102209	0
OTU4934	LN870758.1.1414	Bacteria;Verrucomicrobiota;Omnitrophia;Omnitrophales;Omnitrophaceae;Candidatus_Omnitrophus;uncultured_bacterium	87.56	0	0.00105612	0	0	0
OTU494	LCQK01000003.64973.66448	Bacteria;Patescibacteria;Parcubacteria;Candidatus_Jorgensenbacteria;Candidatus_Jorgensenbacteria_bacterium_GW2011_GWB1_50_10	86.52	0	0.00105612	0	0	0
OTU4940	AB694977.1.1470	Bacteria;Proteobacteria;Gammaproteobacteria;Xanthomonadales;Xanthomonadaceae;Lysobacter;Lysobacter_oligotrophicus	99.77	0.0011083	0	0.01386732	0	0
OTU4943	ET881253.1.1526	Bacteria;Myxococcota;Polyangia;TASB-TL25;uncultured_bacterium	91.38	0.0011083	0	0	0	0
OTU4945	HQ120813.1.1510	Bacteria;Proteobacteria;Gammaproteobacteria;Xanthomonadales;Xanthomonadaceae;Lysobacter;uncultured_bacterium	99.77	0	0	0.00138673	0.00102209	0.00098733
OTU4948	JQ278772.1.1501	Bacteria;Proteobacteria;Gammaproteobacteria;Burkholderiales;Gallionellaceae;Candidatus_Nitrotoga;uncultured_Rhodocyclaceae_bacterium	98.6	0	0	0	0	0.00098733
OTU495	JN868881.1.1534	Bacteria;Proteobacteria;Gammaproteobacteria;Burkholderiales;Burkholderiaceae;Polynucleobacter;uncultured_bacterium	100	0	0.00316837	0.00138673	0.00102209	0.00098733
OTU4951	CP015506.11217.12771	Bacteria;Firmicutes;Bacilli;Bacillales;Bacillaceae;Bacillus;Bacillus_oceanisediminis_2691	100	0	0.00105612	0.00138673	0.00204417	0.00197467
OTU4952	FPLS01022762.17.1453	Bacteria;Proteobacteria;Alphaproteobacteria;Rhodobacterales;Rhodobacteraceae;Flavimaricola;metagenome	99.25	0.0011083	0	0.00277346	0	0.00098733
OTU4955	ET937864.1.1499	Bacteria;Proteobacteria;Gammaproteobacteria;Burkholderiales;Nitrosomonadaceae;Nitrosomonas;uncultured_bacterium	99.06	0	0	0	0	0.00098733
OTU4958	FJ529934.1.1484	Bacteria;Bacteroidota;Bacteroidia;Cytophagales;Cytophagaceae;Cytophaga;uncultured_bacterium	97.39	0	0	0	0	0.00098733
OTU496	FJ263017.1.1461	Bacteria;Firmicutes;Bacilli;Bacillales;Bacillaceae;Bacillus;Bacillus_sp._XA8	100	0	0.00211224	0	0.00511044	0.00098733
OTU497	JQ675517.1.1377	Bacteria;Myxococcota;bacteriap25;uncultured_bacterium	84.54	0	0.00211224	0	0	0
OTU498	FPLK01000264.2.1373	Bacteria;Bacteroidota;Bacteroidia;Sphingobacteriales;env.OPS_17;metagenome	99.76	0	0	0	0	0.00098733
OTU4981	HQ190277.1.1493	Bacteria;Bacteroidota;Bacteroidia;Sphingobacteriales;env.OPS_17;uncultured_bacterium	92.18	0	0.00105612	0	0	0
OTU4985	HM444888.1.1352	Bacteria;Nitrospirota;Nitrospiria;Nitrospirales;Nitrospiraceae;Nitrospira;uncultured_bacterium	100	0.0011083	0	0.00138673	0	0.00098733
OTU4993	LN870812.1.1394	Bacteria;Proteobacteria;Gammaproteobacteria;Burkholderiales;Nitrosomonadaceae;MND1;uncultured_bacterium	100	0.0011083	0	0.00138673	0	0
OTU4997	KM265469.1.1365	Bacteria;Proteobacteria;Alphaproteobacteria;Rhodobacterales;Rhodobacteraceae;Paracoccus;Paracoccus_sp._JL7	100	0	0	0	0.00102209	0.00098733

OTU5	AB680170.1.1462	Bacteria;Proteobacteria;Gammaproteobacteria;Pseudomonadales;Pseudomonadaceae;Pseudomonas;Pseudomonas_synxantha	100	24.3383429	0.06759183	0.15947415	0.07563446	0.23498514
OTU50	KF911258.1.1474	Bacteria;Firmicutes;Bacilli;Bacillales;Planococcaceae;Sporosarcina;uncultured_bacterium	99.77	0.00443321	0.00422449	0.00832039	0.01022087	5.37800026
OTU5002	HF548406.1.1395	Bacteria;Proteobacteria;Alphaproteobacteria;Sphingomonadales;Sphingomonadaceae;Sphingomonas;Sphingomonas_sp._Bg12ra	100	0	0.00105612	0.00138673	0.00102209	0
OTU5008	JX521618.1.1526	Bacteria;Patescibacteria;Parcubacteria;Candidatus_Nomurabacteria;uncultured_bacterium	88.58	0	0	0.00138673	0	0
OTU5009	AB472942.1.1414	Bacteria;Proteobacteria;Gammaproteobacteria;Enterobacterales;Aeromonadaceae;Aeromonas;Aeromonas_sobria	100	0	0	0.00277346	0	0
OTU501	KX033858.1.1518	Bacteria;Proteobacteria;Gammaproteobacteria;Enterobacterales;Alteromonadaceae;Rheinheimera;Rheinheimera_sp._SA_1	99.77	0	0.00316837	0	0	0.32878173
OTU5011	HM187412.1.1331	Bacteria;Chloroflexi;TK10;uncultured_bacterium	99.26	0	0	0	0.00102209	0
OTU5012	HM187129.1.1480	Bacteria;Bdellovibrionota;Oligoflexia;0319-6G20;uncultured_bacterium	96.26	0	0	0	0	0.00098733
OTU5016	AB286463.1.1374	Bacteria;Proteobacteria;Alphaproteobacteria;Caulobacterales;Hyphomonadaceae;Hirschia;uncultured_bacterium	99	0.00443321	0.00105612	0	0.00306626	0
OTU5017	AB696323.1.1448	Bacteria;Actinobacteriota;Actinobacteria;Pseudonocardiales;Pseudonocardiaceae;Pseudonocardia;uncultured_bacterium	99.76	0.00775812	0.03485204	0.00138673	0.00102209	0.002962
OTU5019	ET134201.1.1319	Bacteria;Chloroflexi;JG30-KF-CM66;uncultured_bacterium	92.71	0	0	0	0	0.00098733
OTU5023	JQ278940.1.1499	Bacteria;Nitrospirota;Nitrospiria;Nitrospirales;Nitrospiraceae;Nitrospira;uncultured_Nitrospira_sp.	99.28	0	0	0	0	0.00098733
OTU5025	AB981193.1.1453	Bacteria;Proteobacteria;Gammaproteobacteria;Xanthomonadales;Xanthomonadaceae;Stenotrophomonas;Stenotrophomonas_rhizophila	100	0	0	0	0.00102209	0
OTU503	FN668074.1.1480	Bacteria;Bacteroidota;Bacteroidia;Sphingobacteriales;NS11-12_marine_group;uncultured_Sphingobacterium_sp.	99.29	0	0.00211224	0	0	0
OTU5037	KF616728.1.1486	Bacteria;Myxococcota;bacteriap25;uncultured_bacterium	89.51	0	0.00105612	0	0	0
OTU504	HQ672167.1.1483	Bacteria;Bacteroidota;Bacteroidia;Flavobacteriales;NS9_marine_group;uncultured_bacterium	99.29	0.0011083	0	0	0.00102209	0.00098733
OTU5042	AB021409.1.1503	Bacteria;Proteobacteria;Gammaproteobacteria;Pseudomonadales;Pseudomonadaceae;Pseudomonas;Pseudomonas_monteilii	100	0	0	0.0041602	0	0
OTU5044	MHGG01000050.25339.26910	Bacteria;Verrucomicrobiota;Omnitrophia;Omnitrophales;Omnitrophaceae;Candidatus_Omnitrophus;Omnitrophica_WOR_2_bacterium_GWF2_63_9	92.31	0.0011083	0	0	0	0
OTU505	MHGG01000050.25339.26910	Bacteria;Verrucomicrobiota;Omnitrophia;Omnitrophales;Omnitrophaceae;Candidatus_Omnitrophus;Omnitrophica_WOR_2_bacterium_GWF2_63_9	92.31	0.0011083	0	0	0	0.00098733
OTU5050	MF942642.1.1390	Bacteria;Verrucomicrobiota;Omnitrophia;Omnitrophales;Omnitrophaceae;Candidatus_Omnitrophus;uncultured_bacterium	91.38	0	0	0	0	0.00098733
OTU506	EF190149.1.1478	Bacteria;Bacteroidota;Bacteroidia;Flavobacteriales;Flavobacteriaceae;Flavobacterium;uncultured_bacterium	99.76	0.0011083	0.00105612	0.00138673	0	0.50650158
OTU5062	GQ072498.1.1339	Bacteria;Actinobacteriota;Actinobacteria;Corynebacteriales;Mycobacteriaceae;Mycobacterium;uncultured_bacterium	99.02	0	0	0.00277346	0	0
OTU507	AM934647.1.1486	Bacteria;Bacteroidota;Bacteroidia;Flavobacteriales;Flavobacteriaceae;Flavobacterium;Flavobacterium_sp._WB2.3-46	99.53	0.0011083	0.01795408	0.00277346	0.00511044	0.33273106
OTU5070	KF758585.1.1502	Bacteria;Spirochaetota;Spirochaetia;Spirochaetales;Spirochaetaceae;Spirochaeta_2;uncultured_Spirochaeta_sp.	98.83	0.0011083	0	0	0	0
OTU5086	HM066571.1.1533	Bacteria;Bdellovibrionota;Oligoflexia;0319-6G20;uncultured_bacterium	98.36	0	0.00105612	0	0	0
OTU5088	HG969252.1.1474	Bacteria;Proteobacteria;Gammaproteobacteria;Pseudomonadales;Marinobacteraceae;Marinobacter;Tamilnaduibacter_salinus	94.38	0	0	0	0.00102209	0.00098733
OTU509	EF520602.1.1486	Bacteria;Bacteroidota;Bacteroidia;Chitinophagales;uncultured;uncultured_Sphingobacteriia_bacterium	95.26	0	0.00316837	0	0	0
OTU5091	AM084887.1.1296	Bacteria;Proteobacteria;Gammaproteobacteria;Burkholderiales;Nitrosomonadaceae;MND1;uncultured_beta_proteobacterium	98.36	0	0	0	0.00102209	0
OTU5095	ET134906.1.1312	Bacteria;Verrucomicrobiota;Omnitrophia;Omnitrophales;Omnitrophaceae;Candidatus_Omnitrophus;uncultured_bacterium	90.19	0	0	0	0	0.00197467
OTU51	KT122361.1.1505	Bacteria;Nitrospirota;Nitrospiria;Nitrospirales;Nitrospiraceae;Nitrospira;uncultured_Nitrospira_sp.	99.04	0.00664982	0.00422449	0.00832039	0.00408835	0.00493666
OTU510	FPLK01000324.10.1502	Bacteria;Actinobacteriota;Actinobacteria;Frankiales;Sporichthyaceae;hgcI_clade;metagenome	99.51	0.0011083	0.00105612	0.02218771	0.00102209	0
OTU5101	JN628304.1.1500	Bacteria;Proteobacteria;Gammaproteobacteria;Burkholderiales;Nitrosomonadaceae;Nitrosomonas;uncultured_bacterium	100	0	0	0	0	0.00098733
OTU511	AB286421.1.1403	Bacteria;Planctomycetota;Phycisphaerae;Phycisphaerales;Phycisphaeraceae;SM1A02;uncultured_bacterium	95.98	0	0	0.00138673	0	0.00098733
OTU5112	ASPP01042319.1.1433	Bacteria;Proteobacteria;Alphaproteobacteria;Rhodobacterales;Rhodobacteraceae;Rhodobacter;Reticulomyxa_filosa	100	0	0	0.00554693	0.00102209	0
OTU5115	ATSA02000014.12085.13545	Bacteria;Proteobacteria;Alphaproteobacteria;Rhizobiales;Rhizobiaceae;Allorhizobium-Neorhizobium-Pararhizobium-Rhizobium;Rhizobiales_bacterium_JGI_001012-O08	98.51	0	0	0.00277346	0	0
OTU5118	AB021407.1.1502	Bacteria;Proteobacteria;Gammaproteobacteria;Burkholderiales;Comamonadaceae;Pelomonas;Pelomonas_saccharophila	100	0	0	0	0	0.00098733
OTU512	JQ278784.1.1466	Bacteria;Elusimicrobiota;Elusimicrobia;Lineage_IV;uncultured_bacterium	95.53	0	0.00105612	0	0	0
OTU5129	HQ445757.1.1486	Bacteria;Planctomycetota;Pla4_lineage;uncultured_bacterium	97.52	0	0.00105612	0	0	0
OTU513	JN626511.1.1354	Bacteria;Bacteroidota;Bacteroidia;Sphingobacteriales;env.OPS_17;uncultured_bacterium	99.53	0	0	0	0	0.00098733
OTU5131	GT295969.1.1408	Bacteria;Bacteroidota;Bacteroidia;Flavobacteriales;Flavobacteriaceae;Flavobacterium;Flavobacterium_anseonense	99.53	0	0	0.00277346	0	0.002962
OTU5133	KM462133.1.1519	Bacteria;Bacteroidota;Bacteroidia;Bacteroidales;Prevotellaceae;Alloprevotella;Alloprevotella_sp._feline_oral_taxon_167	100	0.0011083	0	0	0	0
OTU514	AY734239.1.1379	Bacteria;Nitrospirota;Leptospirillia;Leptospirillales;Leptospirillaceae;Leptospirillum;uncultured_bacterium	99.07	0.00221661	0.00211224	0.00138673	0.00102209	0
OTU5144	JQ278816.1.1534	Bacteria;Verrucomicrobiota;Omnitrophia;Omnitrophales;Omnitrophaceae;Candidatus_Omnitrophus;uncultured_bacterium	90.93	0	0	0	0	0.00098733
OTU515	KC358393.1.1301	Bacteria;Verrucomicrobiota;Omnitrophia;Omnitrophales;Omnitrophaceae;Candidatus_Omnitrophus;uncultured_bacterium	90.68	0	0	0	0	0.00098733
OTU5152	JN868748.1.1554	Bacteria;Verrucomicrobiota;Verrucomicrobiae;Chthoniobacterales;Chthoniobacteraceae;LD29;uncultured_bacterium	99.3	0	0	0.00277346	0	0
OTU5153	EF680204.1.943	Archaea;Hadarchaeota;Hadarchaeia;Hadarchaeales;uncultured_archaeon	72.54	0.0011083	0	0	0	0
OTU5155	MF942653.1.1445	Bacteria;Verrucomicrobiota;Omnitrophia;Omnitrophales;Omnitrophaceae;Candidatus_Omnitrophus;uncultured_bacterium	91.82	0	0	0	0.00102209	0
OTU5156	KF576216.1.1489	Bacteria;Bacteroidota;Bacteroidia;Flavobacteriales;Flavobacteriaceae;Hoppeia;Hoppeia_youngheungensis	98.1	0	0.00105612	0	0	0
OTU5159	GQ402722.1.1440	Bacteria;Elusimicrobiota;Elusimicrobia;Lineage_IV;uncultured_bacterium	89.64	0.0011083	0	0	0	0
OTU516	KX348539.1.1502	Bacteria;Proteobacteria;Gammaproteobacteria;Xanthomonadales;Rhodanobacteraceae;Ahniella;uncultured_bacterium	98.83	0	0.00211224	0.00138673	0.00204417	0
OTU5161	AJ318134.1.1441	Bacteria;Proteobacteria;Alphaproteobacteria;Rhizobiales;Devosiaceae;Devosia;uncultured_Alphaproteobacteria_bacterium	99.75	0.00332491	0	0	0.00204417	0.002962
OTU5164	KX504534.1.1485	Bacteria;Bdellovibrionota;Oligoflexia;Silvanigrellales;Silvanigrellaceae;uncultured;uncultured_bacterium	89.49	0	0	0.00277346	0	0
OTU5167	MFTT01000025.14894.16462	Bacteria;Patescibacteria;Parcubacteria;Candidatus_Nomurabacteria;Candidatus_Nomurabacteria_bacterium_RIFCSPHIGHO2_01_FTLL_42_16	79.09	0	0	0.00138673	0	0
OTU517	AM696895.1.1479	Bacteria;Firmicutes;Clostridia;Peptostreptococcales-Tissierellales;Family_XI;Anaerococcus;uncultured_bacterium	100	0	0.00105612	0.00138673	0	0
OTU518	ET134909.1.1431	Bacteria;Verrucomicrobiota;Omnitrophia;Omnitrophales;Omnitrophaceae;Candidatus_Omnitrophus;uncultured_bacterium	90.21	0.00332491	0	0	0	0
OTU5185	MNDM01000022.26933.28470	Bacteria;Nitrospirota;Nitrospiria;Nitrospirales;Nitrospiraceae;Nitrospira;Nitrospirae_bacterium_13_2_20CM_2_62_8	99.76	0	0.00105612	0	0	0
OTU5187	LN680449.1.1351	Bacteria;Proteobacteria;Alphaproteobacteria;uncultured;uncultured_Rhodospirillaceae_bacterium	99	0	0.00105612	0	0	0
OTU519	GQ045521.1.1350	Bacteria;Firmicutes;Clostridia;Peptostreptococcales-Tissierellales;Family_XI;Anaerococcus;uncultured_bacterium	100	0	0	0.00138673	0	0
OTU5195	GQ472458.1.1490	Bacteria;Proteobacteria;Gammaproteobacteria;Burkholderiales;Comamonadaceae;uncultured;uncultured_bacterium	99.06	0	0.00105612	0	0	0
OTU5196	JF747934.1.1505	Bacteria;Elusimicrobiota;Endomicrobia;Endomicrobiales;Endomicrobiaceae;Endomicrobium;uncultured_bacterium	85.48	0	0	0.00138673	0	0
OTU5197	AY326583.1.1489	Bacteria;Myxococcota;bacteriap25;uncultured_soil_bacterium	85.81	0	0	0	0	0.00098733
OTU52	ET703353.1.1401	Bacteria;Bacteroidota;Bacteroidia;Cytophagales;Cyclobacteriaceae;Algoriphagus;uncultured_Hongiella_sp.	100	0.00664982	0.01161735	0.01525405	0.00408835	0.01086066
OTU5201	JF166617.1.1326	Bacteria;Proteobacteria;Alphaproteobacteria;Acetobacterales;Acetobacteraceae;Craurococcus-Caldovatus;uncultured_bacterium	99.25	0	0	0	0.00102209	0
OTU5204	JN032895.1.1442	Bacteria;Actinobacteriota;Acidimicrobiia;Microtrichales;Microtrichaceae;IMCC26207;uncultured_bacterium	99.75	0	0.00211224	0	0	0
OTU521	AB012061.1.1381	Bacteria;Proteobacteria;Alphaproteobacteria;Sphingomonadales;Sphingomonadaceae;Erythrobacter;alpha_proteobacterium_MBIC2351	100	0	0	0	0	0.00197467
OTU5216	JQ818208.1.1210	Bacteria;Proteobacteria;Gammaproteobacteria;Enterobacterales;Morganellaceae;Candidatus_Hamiltonella;uncultured_Enterobacteriaceae_bacterium	95.32	0.0011083	0	0	0	0.04936663
OTU5219	FPLK01001565.9.1505	Bacteria;Bdellovibrionota;Bdellovibrionia;Bacteriovoracales;Bacteriovoracaceae;Peredibacter;metagenome	99.53	0	0	0.00138673	0.00204417	0

OTU522	ET512011.1.1361	Bacteria;Proteobacteria;Gammaproteobacteria;Pseudomonadales;Pseudomonadaceae;Pseudomonas;uncultured_bacterium	94.15	1.16704349	0.00316837	0.00277346	0.00102209	0.02270865
OTU5225	GT208400.1.1441	Bacteria;Proteobacteria;Gammaproteobacteria;Xanthomonadales;Xanthomonadaceae;Arenimonas;uncultured_prokaryote	96.49	0.0011083	0	0	0	0.00098733
OTU5227	ET134909.1.1431	Bacteria;Verrucomicrobiota;Omnitrophia;Omnitrophales;Omnitrophaceae;Candidatus_Omnitrophus;uncultured_bacterium	95.57	0	0	0	0	0.00098733
OTU523	FJ976603.1.1389	Bacteria;Proteobacteria;Gammaproteobacteria;Pseudomonadales;Moraxellaceae;Acinetobacter;Acinetobacter_johnsonii	100	0.0011083	0.00105612	0	0.00102209	0.00098733
OTU5233	AB180234.1.1396	Bacteria;Actinobacteriota;Actinobacteria;Micrococcales;Intrasporangiaceae;Janibacter;Janibacter_sp._DFA10	99.75	0	0.00211224	0	0	0
OTU524	KC886756.1.1485	Bacteria;Bacteroidota;Bacteroidia;Flavobacteriales;Crocinitomicaceae;Fluviicola;uncultured_Cryomorphaceae_bacterium	99.76	0.00443321	0.00211224	0.00832039	0	0.00098733
OTU5249	KY356876.1.911	Archaea;Nanoarchaeota;Nanoarchaeia;Woesearchaeales;GW2011_GWC1_47_15;uncultured_archaeon	91.28	0	0	0.00277346	0	0
OTU525	KY356865.1.937	Archaea;Nanoarchaeota;Nanoarchaeia;Woesearchaeales;GW2011_GWC1_47_15;uncultured_archaeon	87.17	0	0	0	0	0.00098733
OTU5254	KC358393.1.1301	Bacteria;Verrucomicrobiota;Omnitrophia;Omnitrophales;Omnitrophaceae;Candidatus_Omnitrophus;uncultured_bacterium	90.91	0	0.00105612	0	0	0
OTU5255	JF168453.1.1343	Bacteria;Actinobacteriota;Acidimicrobiia;Microtrichales;Ilumatobacteraceae;uncultured;uncultured_bacterium	100	0.01662455	0.00211224	0.00277346	0.03168471	0
OTU5256	KX123338.1.3317	Archaea;Thermoplasmatota;Thermoplasmata;uncultured;Candidatus_Amesbacteria_bacterium_GW2011_GWC1_47_15	94.19	0	0	0	0.00102209	0
OTU5257	DQ223087.1.1346	Bacteria;Bdellovibrionota;Bdellovibrionia;Bdellovibrionales;Bdellovibrionaceae;OM27_clade;uncultured_bacterium	97.53	0.0011083	0	0	0	0
OTU526	FPLK01002510.10.1490	Bacteria;Actinobacteriota;Actinobacteria;Micrococcales;Microbacteriaceae;Rhodoluna;metagenome	99.51	0	0	0.02496117	0	0
OTU5264	JF222242.1.1303	Bacteria;Proteobacteria;Alphaproteobacteria;Rhizobiales;Hyphomicrobiaceae;Hyphomicrobium;uncultured_bacterium	100	0	0	0.00138673	0	0
OTU5269	DQ333897.1.1460	Bacteria;Firmicutes;Bacilli;Bacillales;Planococcaceae;Paenisporosarcina;Paenisporosarcina_quisquiliarum	100	0	0.00105612	0	0.02044175	0
OTU527	ET117725.1.1506	Bacteria;Actinobacteriota;Actinobacteria;Frankiales;Sporichthyaceae;hgcI_clade;uncultured_actinobacterium	99.51	0	0	0.01386732	0	0
OTU5274	AF293003.1.1492	Bacteria;Proteobacteria;Gammaproteobacteria;Burkholderiales;Comamonadaceae;Comamonas;uncultured_Green_Bay_ferromanganous_micronodule_bacterium_MNE12	98.36	0.0011083	0	0	0	0
OTU528	AF522998.1.1493	Bacteria;Proteobacteria;Gammaproteobacteria;Burkholderiales;Comamonadaceae;Aquabacterium;uncultured_Aquabacterium_sp.	99.77	0.00221661	0	0	0	0.00197467
OTU5284	HE603186.1.1453	Bacteria;Verrucomicrobiota;Omnitrophia;Omnitrophales;Omnitrophaceae;Candidatus_Omnitrophus;uncultured_Firmicutes_bacterium	91.84	0	0	0	0.00102209	0
OTU5285	FJ916089.1.1513	Bacteria;Actinobacteriota;Actinobacteria;Corynebacteriales;Mycobacteriaceae;Mycobacterium;uncultured_actinobacterium	99.75	0.0011083	0	0	0	0
OTU529	HM244223.1.906	Archaea;Aenigmarchaeota;Aenigmarchaeia;Aenigmarchaeales;uncultured_archaeon	89.14	0	0.00211224	0	0	0
OTU5296	MFVN01000036.3183.4707	Archaea;Nanoarchaeota;Nanoarchaeia;Woesearchaeales;Candidatus_Nomurabacteria_bacterium_RIFCSPLOWO2_02_FTLL_42_17	86.01	0	0.00105612	0	0	0
OTU5299	GQ406175.1.1484	Bacteria;Bacteroidota;Bacteroidia;Flavobacteriales;Flavobacteriaceae;Flavobacterium;uncultured_Bacteroidetes_bacterium	99.53	0	0.00105612	0.00277346	0	0.00098733
OTU53	AB680165.1.1462	Bacteria;Proteobacteria;Gammaproteobacteria;Pseudomonadales;Pseudomonadaceae;Pseudomonas;Pseudomonas_fluorescens	100	5.18242674	0.01795408	0.18720879	0.01022087	0.25275713
OTU530	HQ224601.1.1426	Bacteria;Proteobacteria;Gammaproteobacteria;Pseudomonadales;Pseudomonadaceae;Pseudomonas;Pseudomonas_sp._SGb186	100	0.0011083	0.00105612	0.78211671	0.00306626	0.00098733
OTU5306	AF373196.1.1490	Bacteria;Proteobacteria;Gammaproteobacteria;Enterobacterales;Erwiniaceae;Pantoea;Pantoea_agglomerans	99.06	0.00443321	0.07709693	0.00138673	0.00204417	0.00098733
OTU5308	MH362787.1.1265	Bacteria;Actinobacteriota;Actinobacteria;Micrococcales;Intrasporangiaceae;Knoellia;Actinobacteria_bacterium	100	0	0	0	0.00102209	0.00098733
OTU531	ET703171.1.1433	Bacteria;Chloroflexi;Chloroflexia;Chloroflexales;Roseiflexaceae;uncultured;uncultured_Roseiflexus_sp.	100	0.0011083	0	0	0	0.00098733
OTU5310	FPLL01002318.7.1543	Bacteria;Verrucomicrobiota;Verrucomicrobiae;Opitutales;Opitutaceae;Opitutus;metagenome	98.36	0	0.00105612	0	0	0
OTU5311	KF999725.1.1412	Bacteria;Proteobacteria;Gammaproteobacteria;Enterobacterales;Alteromonadaceae;Rheinheimera;Rheinheimera_sp._THG-LS118	99.06	0	0.00105612	0	0	0
OTU5313	AB004747.1.1438	Bacteria;Proteobacteria;Gammaproteobacteria;Enterobacterales;Enterobacteriaceae;Kluyvera;Kluyvera_intermedia	99.77	0.04322383	0	0	0	0
OTU532	LCOK01000044.9216.10662	Bacteria;Patescibacteria;Parcubacteria;Candidatus_Ryanbacteria;Candidatus_Giovannonibacteria_bacterium_GW2011_GWB1_47_6b	80.27	0	0.00316837	0	0	0
OTU5321	HE603186.1.1453	Bacteria;Verrucomicrobiota;Omnitrophia;Omnitrophales;Omnitrophaceae;Candidatus_Omnitrophus;uncultured_Firmicutes_bacterium	90.21	0	0	0.00138673	0	0
OTU534	GQ077208.1.1367	Bacteria;Bacteroidota;Bacteroidia;Bacteroidales;Porphyromonadaceae;Porphyromonas;uncultured_bacterium	100	0.0011083	0	0	0.00102209	0
OTU5343	FPLP01011586.10.1487	Bacteria;Proteobacteria;Alphaproteobacteria;Defluviicoccales;uncultured;metagenome	100	0	0	0	0	0.00098733
OTU5344	HQ597988.1.1458	Bacteria;Acidobacteriota;Acidobacteriae;Solibacterales;Solibacteraceae;Candidatus_Solibacter;uncultured_Acidobacteria_bacterium	100	0	0.00105612	0	0	0
OTU535	HM128588.1.1439	Bacteria;Actinobacteriota;Actinobacteria;Frankiales;Sporichthyaceae;hgcI_clade;uncultured_bacterium	99.75	0.0011083	0.00316837	0.00138673	0.00102209	0
OTU5351	JN615922.1.1320	Bacteria;Proteobacteria;Alphaproteobacteria;Dongiales;Dongiaceae;Dongia;uncultured_bacterium	100	0	0	0	0	0.00098733
OTU5352	AB257646.1.1479	Bacteria;Proteobacteria;Alphaproteobacteria;Sphingomonadales;Sphingomonadaceae;Qipengyuania;uncultured_Alphaproteobacteria_bacterium	100	0.00221661	0	0.00277346	0.00306626	0
OTU5353	EF580954.1.1514	Bacteria;Proteobacteria;Gammaproteobacteria;Arenicellales;Arenicellaceae;uncultured;uncultured_bacterium	99.07	0	0	0	0.00102209	0
OTU536	KC358031.1.1241	Bacteria;Chloroflexi;Dehalococcoidia;SAR202_clade;uncultured_bacterium	98.76	0.00221661	0	0.00138673	0.00102209	0
OTU5360	HM187047.1.1427	Bacteria;Verrucomicrobiota;Omnitrophia;Omnitrophales;Omnitrophaceae;Candidatus_Omnitrophus;uncultured_bacterium	93.01	0	0	0	0.00102209	0
OTU5364	FJ437928.1.1504	Bacteria;Verrucomicrobiota;Verrucomicrobiae;Verrucomicrobiales;Rubritaleaceae;Luteolibacter;uncultured_bacterium	98.36	0	0	0.00138673	0	0
OTU5369	FPLK01000417.20.1486	Bacteria;Proteobacteria;Alphaproteobacteria;Sphingomonadales;Sphingomonadaceae;Sandarakinorhabdus;metagenome	99.75	0	0	0.0041602	0	0
OTU537	KP636082.1.1496	Bacteria;Nitrospirota;Nitrospiria;Nitrospirales;Nitrospiraceae;Nitrospira;uncultured_bacterium	99.76	0	0	0.00138673	0	0
OTU5371	HW067158.1.1422	Bacteria;Bacteroidota;Bacteroidia;Sphingobacteriales;Sphingobacteriaceae;Pedobacter;unidentified	99.76	0.05541517	0	0.00138673	0	0.00493666
OTU5376	AB630392.1.1435	Bacteria;Proteobacteria;Alphaproteobacteria;Rhizobiales;A0839;uncultured_bacterium	100	0	0	0.00277346	0	0
OTU5378	ATLT01001521.394.1849	Bacteria;Patescibacteria;Gracilibacteria;JGI_0000069-P22;bioreactor_metagenome	96.98	0.0011083	0	0	0	0
OTU538	HM444841.1.1385	Bacteria;Planctomycetota;Pla4_lineage;uncultured_bacterium	97.43	0	0	0.00138673	0	0
OTU5388	JQ278816.1.1534	Bacteria;Verrucomicrobiota;Omnitrophia;Omnitrophales;Omnitrophaceae;Candidatus_Omnitrophus;uncultured_bacterium	97.88	0	0	0.00138673	0	0
OTU5389	LCOX01000043.1193.4545	Bacteria;Patescibacteria;Parcubacteria;Candidatus_Jorgensenbacteria;Parcubacteria_group_bacterium_GW2011_GWA2_47_9	82.97	0	0.00105612	0	0	0
OTU539	ET512011.1.1361	Bacteria;Proteobacteria;Gammaproteobacteria;Pseudomonadales;Pseudomonadaceae;Pseudomonas;uncultured_bacterium	95.32	0.00886643	0.36330609	0.00277346	0.00613252	0.00394933
OTU5392	ET135157.1.1386	Bacteria;Planctomycetota;Planctomycetes;Pirellulales;Pirellulaceae;Pir4_lineage;uncultured_bacterium	99.05	0.0011083	0	0.00138673	0	0
OTU5393	FPLS01039142.8.1552	Bacteria;Verrucomicrobiota;Verrucomicrobiae;Pedosphaerales;Pedosphaeraceae;SH3-11;metagenome	95.31	0	0	0	0	0.00098733
OTU5395	KY194068.1.1446	Bacteria;Chloroflexi;Dehalococcoidia;SAR202_clade;uncultured_bacterium	96.03	0.0011083	0	0	0	0
OTU5396	FPLS01023335.19.1548	Bacteria;Myxococcota;Myxococcia;Myxococcales;Myxococcaceae;P3OB-42;metagenome	98.12	0	0.00211224	0	0	0
OTU5399	ET037293.1.1477	Bacteria;Actinobacteriota;Actinobacteria;Micrococcales;Micrococcaceae;Citricoccus;Micrococcus_sp._G3DM-95	100	0	0	0	0.00102209	0
OTU54	AY168745.1.1490	Bacteria;Proteobacteria;Gammaproteobacteria;Burkholderiales;Comamonadaceae;uncultured;uncultured_bacterium	100	0.01219134	0.01689796	0.0041602	0.01022087	0.00098733
OTU540	EF659437.1.1523	Bacteria;Actinobacteriota;Actinobacteria;Micrococcales;Microbacteriaceae;Candidatus_Aquiluna;uncultured_bacterium	99.75	0.0011083	0.00105612	0.00693366	0	0
OTU5409	AF493635.1.1300	Bacteria;Bacteroidota;Bacteroidia;Flavobacteriales;Flavobacteriaceae;Flavobacterium;Flavobacterium_sp._EP030	99.52	0.0011083	0	0.06656312	0	0
OTU541	EF471696.1.1463	Bacteria;Actinobacteriota;Acidimicrobiia;Microtrichales;Ilumatobacteraceae;CL500-29_marine_group;uncultured_actinobacterium	99.5	0.0011083	0	0	0.00408835	0
OTU542	LN570848.1.1349	Bacteria;Bdellovibrionota;Bdellovibrionia;Bdellovibrionales;Bdellovibrionaceae;Bdellovibrio;uncultured_bacterium	95.31	0	0	0.00138673	0	0
OTU5420	KJ650731.1.1505	Bacteria;Firmicutes;Clostridia;Oscillospirales;Hungateiclostridiaceae;Anaerobacterium;uncultured_Clostridia_bacterium	99.01	0	0	0.00138673	0	0
OTU5425	KJ540651.1.1415	Bacteria;Patescibacteria;Parcubacteria;uncultured_Parcubacteria_group_bacterium	93.05	0	0	0	0.00102209	0
OTU5427	MF319205.1.1455	Bacteria;Proteobacteria;Gammaproteobacteria;Enterobacterales;Alteromonadaceae;Paraglaciecola;Paraglaciecola_aestuariivivens	99.3	0	0.00105612	0	0	0
OTU543	CXWJ01006813.2949.4408	Bacteria;Nitrospirota;Nitrospiria;Nitrospirales;Nitrospiraceae;Nitrospira;wastewater_metagenome	99.76	0.0011083	0.00105612	0	0	0.00098733
OTU544	FPLK01002673.23.1523	Bacteria;Bacteroidota;Bacteroidia;Chitinophagales;Chitinophagaceae;Sediminibacterium;metagenome	99.53	0.0011083	0.00211224	0.00138673	0	0

OTU5444	AY922093.1.1372	Bacteria;Patescibacteria;Parcubacteria;uncultured_Parcubacteria_group_bacterium	90.39	0	0.00105612	0	0	0
OTU5447	FM253668.1.1449	Bacteria;Proteobacteria;Alphaproteobacteria;Rhizobiales;Hyphomicrobiaceae;Pedomicrobium;uncultured_Alphaproteobacteria_bacterium	99.75	0	0	0.00138673	0	0
OTU5457	JQ278816.1.1534	Bacteria;Verrucomicrobiota;Omnitrophia;Omnitrophales;Omnitrophaceae;Candidatus_Omnitrophus;uncultured_bacterium	91.86	0	0	0	0	0.00098733
OTU5458	JF116235.1.1374	Bacteria;Bdellovibrionota;Oligoflexia;0319-6G20;uncultured_bacterium	94.86	0	0	0	0.00102209	0
OTU5459	MF942642.1.1390	Bacteria;Verrucomicrobiota;Omnitrophia;Omnitrophales;Omnitrophaceae;Candidatus_Omnitrophus;uncultured_bacterium	91.61	0	0.00105612	0	0	0
OTU5460	LCOT01000026.4898.6395	Bacteria;Patescibacteria;Parcubacteria;Candidatus_Jorgensenbacteria;Parcubacteria_group_bacterium_GW2011_GWA2_47_8b	89.72	0	0	0.00138673	0	0
OTU5466	JX170254.1.1399	Bacteria;Proteobacteria;Alphaproteobacteria;Rickettsiales;Fokiniaceae;MD3-55;uncultured_bacterium	100	0	0	0	0	0.00098733
OTU5468	MF040331.1.1492	Bacteria;Proteobacteria;Gammaproteobacteria;Burkholderiales;Neisseriaceae;uncultured;uncultured_bacterium	94.63	0	0.00105612	0	0	0
OTU547	DQ444978.1.1416	Bacteria;Firmicutes;Bacilli;Paenibacillales;Paenibacillaceae;Paenibacillus;Paenibacillus_sp._Eur1_9.9	99.3	0	0	0.34113601	0.08278907	0.05627795
OTU548	ET801739.1.1501	Bacteria;Proteobacteria;Gammaproteobacteria;Burkholderiales;Comamonadaceae;Limnohabitans;uncultured_bacterium	100	0.0011083	0.00316837	0.01386732	0	0
OTU5484	AB861981.1.1489	Bacteria;Bacteroidota;Bacteroidia;Bacteroidales;Bacteroidaceae;Bacteroides;Bacteroides_caecigallinarum	98.34	0.00221661	0	0.00138673	0	0
OTU5485	MF942653.1.1445	Bacteria;Verrucomicrobiota;Omnitrophia;Omnitrophales;Omnitrophaceae;Candidatus_Omnitrophus;uncultured_bacterium	89.95	0	0	0	0.00102209	0
OTU5488	AB750594.1.1449	Bacteria;Proteobacteria;Alphaproteobacteria;Sphingomonadales;Sphingomonadaceae;Sphingopyxis;uncultured_bacterium	99.75	0.0011083	0.00105612	0	0	0.002962
OTU55	JF178876.1.1345	Bacteria;Bacteroidota;Bacteroidia;Flavobacteriales;Flavobacteriaceae;Flavobacterium;uncultured_bacterium	97.63	0.00664982	0.00316837	0.0305081	0.00408835	0.00394933
OTU5504	ET790279.1.1202	Bacteria;Proteobacteria;Gammaproteobacteria;Gammaproteobacteria_Incertae_Sedis;Tnknown_Family;Candidatus_Ovatusbacter;uncultured_bacterium	76.62	0.00221661	0	0	0.02146383	0
OTU5507	HE648058.1.1411	Bacteria;Proteobacteria;Alphaproteobacteria;Sphingomonadales;Sphingomonadaceae;Sphingopyxis;Sphingopyxis_italica	100	0.0011083	0	0	0.00102209	0.00098733
OTU551	HQ532763.1.1516	Bacteria;Actinobacteriota;Actinobacteria;Micrococcales;Microbacteriaceae;MWH-Ta3;uncultured_actinobacterium	99.51	0.00221661	0.00105612	0.01248059	0	0
OTU5516	HQ224601.1.1426	Bacteria;Proteobacteria;Gammaproteobacteria;Pseudomonadales;Pseudomonadaceae;Pseudomonas;Pseudomonas_sp._SGb186	99.53	0	0	0.04437542	0	0.00197467
OTU552	HM244223.1.906	Archaea;Aenigmarchaeota;Aenigmarchaeia;Aenigmarchaeales;uncultured_archaeon	87.84	0	0.00105612	0	0	0
OTU5524	KT973508.1.1328	Bacteria;Proteobacteria;Alphaproteobacteria;Rhodobacterales;Rhodobacteraceae;Gemmobacter;Gemmobacter_sp._yp3	99.25	0	0	0	0.00102209	0
OTU5529	FJ535179.1.1478	Bacteria;Bacteroidota;Bacteroidia;Flavobacteriales;Flavobacteriaceae;Flavobacterium;uncultured_Flavobacteriia_bacterium	99.27	0	0	0.03189483	0	0.00098733
OTU553	AY133074.1.1424	Bacteria;Elusimicrobiota;Elusimicrobia;Lineage_IV;uncultured_Termite_group_1_bacterium	100	0	0.00105612	0.00138673	0.00102209	0
OTU5532	JQ279045.1.1494	Bacteria;Proteobacteria;Gammaproteobacteria;Burkholderiales;Gallionellaceae;Gallionella;uncultured_Rhodocyclaceae_bacterium	99.06	0	0.00105612	0	0	0
OTU5536	MK121890.1.1411	Bacteria;Proteobacteria;Alphaproteobacteria;Sphingomonadales;Sphingomonadaceae;Erythrobacter;Erythrobacter_sp._KEM-5	99.5	0	0.00105612	0	0.00306626	0
OTU554	MFWP01000008.61.1387	Archaea;Nanoarchaeota;Nanoarchaeia;Woesearchaeales;SCGC_AAA011-D5;Candidatus_Pacearchaeota_archaeon_RBG_19FT_COMBO_34_9	89.82	0	0	0	0.00102209	0
OTU5540	GQ263723.1.1454	Bacteria;Proteobacteria;Gammaproteobacteria;Burkholderiales;Nitrosomonadaceae;MND1;uncultured_bacterium	100	0.0011083	0	0	0	0
OTU5541	AF234747.1.1524	Bacteria;Myxococcota;Polyangia;Haliangiales;Haliangiaceae;Haliangium;uncultured_sludge_bacterium_A9	98.58	0.00221661	0	0	0	0
OTU5544	AB680060.1.1465	Bacteria;Proteobacteria;Gammaproteobacteria;Enterobacterales;Enterobacteriaceae;Klebsiella;Klebsiella_pneumoniae	100	0.0011083	0	0	0	0
OTU5546	EF516540.1.1491	Bacteria;Verrucomicrobiota;Verrucomicrobiae;Pedosphaerales;Pedosphaeraceae;uncultured_bacterium	96.01	0	0	0	0.00102209	0
OTU5548	FP929140.1090464.1091986	Bacteria;Proteobacteria;Gammaproteobacteria;Pseudomonadales;Halomonadaceae;HdN1;gamma_proteobacterium_HdN1	93.21	0	0	0.00138673	0	0.00098733
OTU555	MF942653.1.1445	Bacteria;Verrucomicrobiota;Omnitrophia;Omnitrophales;Omnitrophaceae;Candidatus_Omnitrophus;uncultured_bacterium	92.06	0	0	0	0.00102209	0
OTU556	GT814629.1.1332	Bacteria;Proteobacteria;Gammaproteobacteria;Pseudomonadales;Pseudomonadaceae;Pseudomonas;Pseudomonas_sp._AT12	100	0	0	0.62680275	0.00511044	0.02270865
OTU5563	HM187496.1.1362	Archaea;Crenarchaeota;Methanomethylicia;Methanomethyliales;Methanomethyliaceae;uncultured_archaeon	99.22	0.0011083	0	0	0	0
OTU5566	HQ462509.1.1249	Bacteria;Proteobacteria;Alphaproteobacteria;Rhizobiales;Xanthobacteraceae;uncultured;uncultured_Rhizobiales_bacterium	98.26	0	0	0.00138673	0	0
OTU557	LCOK01000044.9216.10662	Bacteria;Patescibacteria;Parcubacteria;Candidatus_Ryanbacteria;Candidatus_Giovannonibacteria_bacterium_GW2011_GWB1_47_6b	83.22	0	0	0	0.00102209	0
OTU5570	AB089841.1.1476	Bacteria;Actinobacteriota;Actinobacteria;Micrococcales;Micrococcaceae;Arthrobacter;Arthrobacter_globiformis	100	0.00775812	0.00105612	0.05962947	0.00613252	0.01184799
OTU5575	AM934647.1.1486	Bacteria;Bacteroidota;Bacteroidia;Flavobacteriales;Flavobacteriaceae;Flavobacterium;Flavobacterium_sp._WB2.3-46	99.29	0	0	0	0	0.02172132
OTU558	KF836147.1.1531	Bacteria;Nitrospirota;Leptospirillia;Leptospirillales;Leptospirillaceae;Leptospirillum;uncultured_bacterium	91.12	0	0	0	0.00102209	0
OTU5581	FPLS01007638.18.1486	Bacteria;Proteobacteria;Alphaproteobacteria;Rickettsiales;Mitochondria;metagenome	94.03	0.0011083	0	0	0	0
OTU5583	HE974798.1.1477	Bacteria;Chloroflexi;Anaerolineae;Anaerolineales;Anaerolineaceae;uncultured;uncultured_Anaerolineaceae_bacterium	97.77	0.0011083	0	0	0.00102209	0
OTU559	AY599655.1.1425	Bacteria;Bacteroidota;Bacteroidia;Flavobacteriales;Flavobacteriaceae;Flavobacterium;Chryseobacterium_sp._TB4-8-II	99.76	0	0	0.00277346	0	0.005924
OTU5596	FPLS01013203.15.1552	Bacteria;Verrucomicrobiota;Verrucomicrobiae;Opitutales;Opitutaceae;Cephaloticoccus;metagenome	99.3	0	0	0.00138673	0	0
OTU56	AB360546.1.1550	Bacteria;Firmicutes;Bacilli;Paenibacillales;Paenibacillaceae;Paenibacillus;Paenibacillus_macquariensis_subsp._defensor	100	0.00775812	0.02640306	4.37791214	0.81971402	2.46833131
OTU5601	HM749694.1.1368	Bacteria;Proteobacteria;Gammaproteobacteria;Burkholderiales;TRA3-20;uncultured_bacterium	100	0	0.00105612	0	0	0
OTU5603	MK038867.1.1438	Bacteria;Proteobacteria;Gammaproteobacteria;Pseudomonadales;Pseudomonadaceae;Pseudomonas;Pseudomonas_stutzeri	95.79	1.79766813	0.00211224	0.00277346	0.00102209	0.00197467
OTU5604	KT201591.1.1447	Bacteria;Bacteroidota;Bacteroidia;Chitinophagales;uncultured;uncultured_bacterium	95.5	0.0011083	0	0	0	0
OTU5605	HM243925.1.1524	Bacteria;Verrucomicrobiota;Omnitrophia;Omnitrophales;Omnitrophaceae;Candidatus_Omnitrophus;uncultured_bacterium	94.63	0.0011083	0	0	0	0
OTU561	KX504487.1.1440	Bacteria;Proteobacteria;Alphaproteobacteria;Rickettsiales;SM2D12;uncultured_bacterium	99.75	0.0011083	0.00105612	0	0.00204417	0.00098733
OTU5618	KX172614.1.1403	Bacteria;Patescibacteria;Parcubacteria;Candidatus_Yanofskybacteria;uncultured_bacterium	89.38	0	0	0.00138673	0	0
OTU562	DQ664243.1.1426	Bacteria;Proteobacteria;Alphaproteobacteria;Sphingomonadales;Sphingomonadaceae;Sphingopyxis;alpha_proteobacterium_IMCC1725	100	0.0011083	0.00211224	0.00277346	0	0
OTU5624	ET937847.1.1412	Bacteria;Bdellovibrionota;Bdellovibrionia;Bdellovibrionales;Bdellovibrionaceae;Bdellovibrio;uncultured_bacterium	100	0	0	0.00138673	0	0
OTU5628	ET360289.1.1450	Bacteria;Proteobacteria;Alphaproteobacteria;Sphingomonadales;Sphingomonadaceae;Erythrobacter;uncultured_Novosphingobium_sp.	100	0.0011083	0	0	0.00408835	0
OTU563	AJTL01000009.2868995.2870465	Bacteria;Proteobacteria;Gammaproteobacteria;Thiotrichales;Thiotrichaceae;Thiothrix;Thiothrix_nivea_DSM_5205	98.26	0.0011083	0.00105612	0.00138673	0.00102209	0
OTU5630	KX123607.1.1467	Bacteria;Patescibacteria;Parcubacteria;Candidatus_Yanofskybacteria;Candidatus_Yanofskybacteria_bacterium_GW2011_GWF1_44_227	87.02	0.0011083	0	0	0	0
OTU5635	HM187450.1.1355	Bacteria;Verrucomicrobiota;Omnitrophia;Omnitrophales;Omnitrophaceae;Candidatus_Omnitrophus;uncultured_bacterium	96.51	0	0	0.00138673	0	0
OTU564	HM185936.1.1403	Bacteria;Nitrospirota;Leptospirillia;Leptospirillales;Leptospirillaceae;Leptospirillum;uncultured_bacterium	99.06	0	0.00105612	0	0.00102209	0
OTU5641	KM454248.1.1411	Bacteria;Acidobacteriota;Subgroup_22;uncultured_bacterium	96.25	0	0.00105612	0	0.00102209	0
OTU5645	HM186580.1.1402	Bacteria;Nitrospirota;Leptospirillia;Leptospirillales;Leptospirillaceae;Leptospirillum;uncultured_bacterium	99.3	0	0.00105612	0	0	0
OTU565	ET117725.1.1506	Bacteria;Actinobacteriota;Actinobacteria;Frankiales;Sporichthyaceae;hgcI_clade;uncultured_actinobacterium	100	0.00221661	0	0.0041602	0	0
OTU5658	KC358393.1.1301	Bacteria;Verrucomicrobiota;Omnitrophia;Omnitrophales;Omnitrophaceae;Candidatus_Omnitrophus;uncultured_bacterium	91.84	0.0011083	0	0	0	0
OTU566	AB009939.1.1494	Bacteria;Firmicutes;Bacilli;Staphylococcales;Staphylococcaceae;Staphylococcus;Staphylococcus_equorum	100	0	0.00211224	0	0.00102209	0.00098733
OTU567	AB045094.1.1500	Bacteria;Firmicutes;Bacilli;Paenibacillales;Paenibacillaceae;Paenibacillus;Paenibacillus_pabuli	100	0	0	0.08597737	0.38328274	0.00098733
OTU5670	AB681203.1.1455	Bacteria;Bacteroidota;Bacteroidia;Flavobacteriales;Flavobacteriaceae;Salegentibacter;Salegentibacter_mishustinae	99.76	0.0011083	0.00105612	0.00138673	0.00102209	0
OTU5672	DQ297939.1.1477	Bacteria;Bacteroidota;Bacteroidia;Flavobacteriales;Flavobacteriaceae;Flavobacterium;uncultured_soil_bacterium	100	0	0	0	0	0.02567065
OTU5675	JQ426216.1.1512	Bacteria;Myxococcota;Myxococcia;Myxococcales;Myxococcaceae;P3OB-42;uncultured_bacterium	87.59	0	0	0	0	0.00098733
OTU5686	JF236022.1.1361	Bacteria;Verrucomicrobiota;Verrucomicrobiae;Verrucomicrobiales;Rubritaleaceae;Luteolibacter;uncultured_bacterium	100	0	0	0	0	0.00789866
OTU5689	JX521775.1.1447	Bacteria;Patescibacteria;Gracilibacteria;JGI_0000069-P22;uncultured_bacterium	99.5	0.0011083	0	0	0	0

OTU5690	FPLK01000747.9.1521	Bacteria;Proteobacteria;Gammaproteobacteria;Burkholderiales;Oxalobacteraceae;Noviherbaspirillum;metagenome	100	0	0	0	0	0.00098733
OTU5691	AEEH01000006.40.1343	Bacteria;Firmicutes;Clostridia;Peptostreptococcales-Tissierellales;Family_XI;Peptoniphilus;Peptoniphilus_duerdenii_ATCC_BAA-1640	100	0	0.00105612	0	0	0
OTU5699	JN592656.1.1362	Bacteria;Nitrospirota;Nitrospiria;Nitrospirales;Nitrospiraceae;Nitrospira;uncultured_bacterium	97.84	0.0011083	0	0	0	0
OTU57	FPLP01001833.16.1477	Bacteria;Planctomycetota;Phycisphaerae;Phycisphaerales;Phycisphaeraceae;SM1A02;metagenome	99.5	0.0011083	0.00528061	0.0041602	0.00306626	0
OTU570	FPLK01002750.13.1504	Bacteria;Bacteroidota;Bacteroidia;Chitinophagales;Chitinophagaceae;Sediminibacterium;metagenome	99.76	0	0.00105612	0.00138673	0.00102209	0
OTU5705	HM266863.1.1344	Bacteria;Bacteroidota;Bacteroidia;Flavobacteriales;Flavobacteriaceae;Flavobacterium;uncultured_bacterium	98.34	0	0	0	0	0.00098733
OTU571	JQ278816.1.1534	Bacteria;Verrucomicrobiota;Omnitrophia;Omnitrophales;Omnitrophaceae;Candidatus_Omnitrophus;uncultured_bacterium	90.93	0.0011083	0.00105612	0	0	0
OTU5712	ET134561.1.1381	Bacteria;Myxococcota;Polyangia;Polyangiales;Sandaracinaceae;uncultured;uncultured_bacterium	92.06	0	0	0	0	0.00098733
OTU5713	AB294336.1.1439	Bacteria;Campylobacterota;Campylobacteria;Campylobacterales;Arcobacteraceae;uncultured;uncultured_bacterium	100	0	0	0.00138673	0	0
OTU5715	FJ217185.1.1436	Bacteria;Actinobacteriota;Actinobacteria;Micrococcales;Micrococcaceae;Micrococcus;Micrococcus_sp._BBAPs-01d	100	0	0	0.00138673	0	0
OTU5717	AMRV01000034.30.1498	Bacteria;Proteobacteria;Alphaproteobacteria;Sphingomonadales;Sphingomonadaceae;uncultured;alpha_proteobacterium_JLT2015	99.75	0	0	0	0.00204417	0
OTU5719	JQ977256.1.1467	Bacteria;Firmicutes;Bacilli;Bacillales;Planococcaceae;Psychrobacillus;Bacillus_sp._Cza19	95.08	0.0664982	0.00211224	0.29814733	0.56828054	0.60029817
OTU572	CP017150.4047203.4048679	Bacteria;Actinobacteriota;Actinobacteria;Micrococcales;Brevibacteriaceae;Brevibacterium;Brevibacterium_aurantiacum	100	0	0	0	0	0.00098733
OTU573	AJ626712.1.1374	Bacteria;Proteobacteria;Gammaproteobacteria;Pseudomonadales;Moraxellaceae;Acinetobacter;Acinetobacter_beijerinckii	100	0.0011083	0.00211224	0.00138673	0	0.00098733
OTU5731	AB001445.1.1538	Bacteria;Proteobacteria;Gammaproteobacteria;Pseudomonadales;Pseudomonadaceae;Pseudomonas;Pseudomonas_amygdali_pv._morsprunorum	99.06	0	0.00105612	0.07211005	0	0
OTU5732	JF703350.1.1419	Bacteria;Bacteroidota;Bacteroidia;Cytophagales;Microscillaceae;uncultured;uncultured_Bacteroidetes_bacterium	99.29	0	0	0.00138673	0	0
OTU5733	AF320989.1.1452	Bacteria;Proteobacteria;Gammaproteobacteria;Pseudomonadales;Pseudomonadaceae;Pseudomonas;Pseudomonas_tolaasii	98.59	0.09974731	0.00316837	0.00138673	0.00511044	0
OTU5737	KJ566441.1.1088	Archaea;Nanoarchaeota;Nanoarchaeia;Woesearchaeales;uncultured_euryarchaeote	92.51	0	0.00105612	0	0	0
OTU5741	AY454678.1.1067	Archaea;Nanoarchaeota;Nanoarchaeia;Woesearchaeales;uncultured_crenarchaeote	82.41	0	0	0.00138673	0	0
OTU5742	JX490007.1.1506	Bacteria;Firmicutes;Bacilli;Bacillales;Planococcaceae;Sporosarcina;uncultured_soil_bacterium	100	0.01219134	0	0.03466829	0.0643915	0.0424553
OTU5744	LCOK01000044.9216.10662	Bacteria;Patescibacteria;Parcubacteria;Candidatus_Ryanbacteria;Candidatus_Giovannonibacteria_bacterium_GW2011_GWB1_47_6b	88.52	0	0.00105612	0	0	0
OTU5745	MF942653.1.1445	Bacteria;Verrucomicrobiota;Omnitrophia;Omnitrophales;Omnitrophaceae;Candidatus_Omnitrophus;uncultured_bacterium	90.44	0	0.00105612	0	0	0
OTU5753	GQ500773.1.1450	Bacteria;Proteobacteria;Alphaproteobacteria;Rhizobiales;A0839;uncultured_bacterium	100	0	0.00105612	0	0	0
OTU5756	KX828259.1.1276	Bacteria;Proteobacteria;Gammaproteobacteria;Pseudomonadales;Pseudomonadaceae;Pseudomonas;Pseudomonas_sp.	88.5	0.0011083	0.00528061	0.10123142	0.15229101	0.00098733
OTU5759	JQ278816.1.1534	Bacteria;Verrucomicrobiota;Omnitrophia;Omnitrophales;Omnitrophaceae;Candidatus_Omnitrophus;uncultured_bacterium	92.33	0	0.00105612	0	0	0
OTU576	AB680701.1.1388	Bacteria;Proteobacteria;Alphaproteobacteria;Rhodobacterales;Rhodobacteraceae;Paracoccus;Paracoccus_sp._NBRC_14911	100	0	0	0	0.00102209	0
OTU5760	ET374059.1.1516	Bacteria;Elusimicrobiota;Lineage_IIa;uncultured_bacterium	86.17	0	0.00105612	0	0	0
OTU5761	JQ675520.1.1287	Bacteria;Cyanobacteria;Vampirivibrionia;Caenarcaniphilales;uncultured_bacterium	92.59	0	0.00105612	0	0	0.00098733
OTU5764	KX097573.1.1501	Bacteria;Elusimicrobiota;Lineage_IIc;uncultured_bacterium	88.39	0.0011083	0	0	0	0
OTU577	AF286868.1.1461	Bacteria;Proteobacteria;Gammaproteobacteria;Enterobacterales;Yersiniaceae;Serratia;Serratia_grimesii	100	0.00221661	0	0.0041602	0	0.00197467
OTU5770	FPLS01035572.19.1525	Bacteria;Proteobacteria;Gammaproteobacteria;Burkholderiales;Comamonadaceae;uncultured;metagenome	99.3	0	0	0	0.00102209	0
OTU5773	KC527400.1.1227	Bacteria;Bacteroidota;Bacteroidia;Sphingobacteriales;Lentimicrobiaceae;uncultured_bacterium	91	0	0	0	0.00102209	0
OTU5774	AY454636.1.1056	Archaea;Nanoarchaeota;Nanoarchaeia;Woesearchaeales;uncultured_crenarchaeote	80.38	0	0.00105612	0	0	0
OTU5777	JF809781.1.1421	Bacteria;Verrucomicrobiota;Omnitrophia;Omnitrophales;Omnitrophaceae;Candidatus_Omnitrophus;uncultured_bacterium	93.46	0.0011083	0	0	0	0
OTU5784	KX123475.1.1558	Bacteria;Patescibacteria;Parcubacteria;Candidatus_Azambacteria;Parcubacteria_group_bacterium_GW2011_GWC2_36_17	93.99	0	0.00105612	0	0	0
OTU5786	KY194068.1.1446	Bacteria;Chloroflexi;Dehalococcoidia;SAR202_clade;uncultured_bacterium	94.04	0	0.00105612	0	0.00102209	0
OTU579	ET512011.1.1361	Bacteria;Proteobacteria;Gammaproteobacteria;Pseudomonadales;Pseudomonadaceae;Pseudomonas;uncultured_bacterium	95.55	0.53420224	0.00633673	0.00970712	0.00919879	0.00197467
OTU5793	AB025188.1.1406	Bacteria;Proteobacteria;Alphaproteobacteria;Rhodobacterales;Rhodobacteraceae;Paracoccus;Paracoccus_sp._MBIC4017	100	0	0	0.00138673	0	0
OTU5795	MHGG01000050.25339.26910	Bacteria;Verrucomicrobiota;Omnitrophia;Omnitrophales;Omnitrophaceae;Candidatus_Omnitrophus;Omnitrophica_WOR_2_bacterium_GWF2_63_9	92.92	0	0	0.00138673	0	0
OTU5799	ET703375.1.1476	Bacteria;Bacteroidota;Bacteroidia;Flavobacteriales;Flavobacteriaceae;Flavobacterium;uncultured_Flavobacterium_sp.	99.53	0	0	0.00277346	0	0
OTU58	ET800627.1.1498	Bacteria;Proteobacteria;Gammaproteobacteria;Burkholderiales;Comamonadaceae;Limnohabitans;uncultured_bacterium	100	0.00664982	0.00739286	0.0873641	0.00715461	0.00987333
OTU5802	LAZR01000136.31686.33154	Bacteria;Patescibacteria;Parcubacteria;Candidatus_Yanofskybacteria;marine_sediment_metagenome	84.45	0	0.00105612	0	0	0
OTU5803	FJ482182.1.1476	Bacteria;Patescibacteria;Parcubacteria;Candidatus_Nomurabacteria;uncultured_Parcubacteria_group_bacterium	98.77	0	0	0.00554693	0	0
OTU5809	JQ627417.1.1349	Bacteria;Deinococcota;Deinococci;Deinococcales;Trueperaceae;Truepera;uncultured_Deinococcales_bacterium	100	0.0011083	0	0	0	0
OTU581	AY454636.1.1056	Archaea;Nanoarchaeota;Nanoarchaeia;Woesearchaeales;uncultured_crenarchaeote	83.92	0	0	0	0.00102209	0
OTU5812	GT208422.1.1442	Bacteria;Proteobacteria;Gammaproteobacteria;Burkholderiales;Nitrosomonadaceae;DSSD61;uncultured_prokaryote	99.53	0	0	0	0.00102209	0
OTU5815	DQ841234.1.1284	Archaea;Nanoarchaeota;Nanoarchaeia;Woesearchaeales;uncultured_archaeon	86.63	0.0011083	0	0	0	0
OTU5816	KC854904.1.1468	Bacteria;Bacteroidota;Bacteroidia;Cytophagales;Hymenobacteraceae;Pontibacter;Pontibacter_sp._LX8	98.34	0.0011083	0	0	0	0
OTU5819	JF149183.1.1342	Bacteria;Actinobacteriota;Actinobacteria;Corynebacteriales;Corynebacteriaceae;Corynebacterium;uncultured_bacterium	100	0	0.00105612	0	0	0.00098733
OTU5820	MF942688.1.1523	Archaea;Nanoarchaeota;Nanoarchaeia;Woesearchaeales;uncultured_archaeon	81.12	0	0	0.00277346	0	0
OTU5821	KC358393.1.1301	Bacteria;Verrucomicrobiota;Omnitrophia;Omnitrophales;Omnitrophaceae;Candidatus_Omnitrophus;uncultured_bacterium	91.38	0	0	0	0.00102209	0
OTU5823	CP024915.487207.488746	Bacteria;Actinobacteriota;Actinobacteria;Micrococcales;Micrococcaceae;Arthrobacter;Arthrobacter_agilis	99.52	0	0	0	0	0.00098733
OTU583	AB722124.1.1353	Bacteria;Patescibacteria;Parcubacteria;TBA9983;uncultured_bacterium	83.59	0	0	0	0	0.00098733
OTU5839	JQ627584.1.1290	Bacteria;Actinobacteriota;Actinobacteria;Pseudonocardiales;Pseudonocardiaceae;Pseudonocardia;uncultured_actinobacterium	100	0.0011083	0.00105612	0	0	0
OTU585	AB045098.1.1504	Bacteria;Firmicutes;Bacilli;Paenibacillales;Paenibacillaceae;Paenibacillus;Bacillus_sp._HSCC_1651	100	0	0	0.00138673	0.39043735	0.00098733
OTU5850	FJ653966.1.1421	Bacteria;Proteobacteria;Gammaproteobacteria;Enterobacterales;Vibrionaceae;Thaumasiovibrio;uncultured_bacterium	93.91	0.0011083	0.00105612	0	0	0
OTU5854	ET134906.1.1312	Bacteria;Verrucomicrobiota;Omnitrophia;Omnitrophales;Omnitrophaceae;Candidatus_Omnitrophus;uncultured_bacterium	89.98	0	0	0	0	0.00098733
OTU586	AB722124.1.1353	Bacteria;Patescibacteria;Parcubacteria;TBA9983;uncultured_bacterium	85.12	0	0.00105612	0	0	0
OTU5860	KC358393.1.1301	Bacteria;Verrucomicrobiota;Omnitrophia;Omnitrophales;Omnitrophaceae;Candidatus_Omnitrophus;uncultured_bacterium	91.84	0	0	0	0.00102209	0
OTU5870	ET488452.1.1442	Bacteria;Patescibacteria;Parcubacteria;Paceibacterales;Paceibacteraceae;Candidatus_Paceibacter;uncultured_bacterium	83.51	0	0	0	0	0.00098733
OTU5873	HQ753767.1.1417	Bacteria;Actinobacteriota;Actinobacteria;Frankiales;uncultured;uncultured_organism	99.75	0.0011083	0	0.00138673	0	0
OTU5879	HQ597127.1.1505	Bacteria;Acidobacteriota;Vicinamibacteria;Subgroup_17;uncultured_Acidobacteria_bacterium	81.88	0.0011083	0	0	0	0
OTU588	HQ166700.1.1493	Bacteria;Patescibacteria;Parcubacteria;Candidatus_Moranbacteria;uncultured_bacterium	96.55	0.0011083	0	0	0.00102209	0
OTU5882	JN038245.1.1475	Bacteria;Chloroflexi;Dehalococcoidia;S085;uncultured_bacterium	91.83	0	0	0	0	0.00098733
OTU5884	FJ612339.1.1469	Bacteria;Desulfobacterota;Desulfuromonadia;Bradymonadales;uncultured_bacterium	99.26	0.0011083	0	0	0.00102209	0.00098733
OTU589	AF293012.1.1505	Bacteria;Nitrospirota;Nitrospiria;Nitrospirales;Nitrospiraceae;Nitrospira;uncultured_Green_Bay_ferromanganous_micronodule_bacterium_MNF8	99.52	0	0	0.00554693	0	0.00098733
OTU5894	GQ302572.1.1493	Bacteria;Acidobacteriota;Blastocatellia;Blastocatellales;Blastocatellaceae;uncultured;uncultured_Acidobacterium_sp.	99.75	0	0	0.05824273	0	0.00197467

OTU59	AF035052.1.1416	Bacteria;Proteobacteria;Gammaproteobacteria;Burkholderiales;Comamonadaceae;Aquabacterium;Aquabacterium_parvum	100	0.01329964	0.01478571	0.00693366	0.00613252	0.01283532
OTU590	LCOT01000026.4898.6395	Bacteria;Patescibacteria;Parcubacteria;Candidatus_Jorgensenbacteria;Parcubacteria_group_bacterium_GW2011_GWA2_47_8b	90.67	0	0.00211224	0	0.00102209	0
OTU5902	HM237097.1.1234	Bacteria;Acidobacteriota;Acidobacteriae;Bryobacterales;Bryobacteraceae;Bryobacter;uncultured_bacterium	94.53	0	0.02957143	0	0	0
OTU5907	KP866212.1.1451	Bacteria;Bacteroidota;Bacteroidia;Flavobacteriales;Flavobacteriaceae;Flavobacterium;Flavobacterium_sp._HMF3121	99.29	0	0	0.00138673	0	0
OTU5909	ET881253.1.1526	Bacteria;Myxococcota;Polyangia;TASB-TL25;uncultured_bacterium	91.38	0	0.00105612	0.00138673	0	0
OTU591	LJNF01000007.41371.42892	Bacteria;Patescibacteria;Parcubacteria;Candidatus_Yanofskybacteria;Parcubacteria_bacterium_DG_74_2	81.08	0	0	0	0	0.00098733
OTU5913	CP016460.3086814.3088288	Bacteria;Proteobacteria;Alphaproteobacteria;Sphingomonadales;Sphingomonadaceae;Blastomonas;Blastomonas_sp._RAC04	100	0.0011083	0	0	0	0
OTU5918	FJ592565.1.1343	Bacteria;Proteobacteria;Alphaproteobacteria;Sphingomonadales;Sphingomonadaceae;Sphingomonas;uncultured_bacterium	100	0	0	0.00138673	0	0
OTU592	JQ278816.1.1534	Bacteria;Verrucomicrobiota;Omnitrophia;Omnitrophales;Omnitrophaceae;Candidatus_Omnitrophus;uncultured_bacterium	96.24	0	0	0	0.00102209	0
OTU5921	HM187255.1.1471	Bacteria;Proteobacteria;Gammaproteobacteria;Burkholderiales;Nitrosomonadaceae;MND1;uncultured_bacterium	98.59	0	0.00105612	0	0	0
OTU5922	AF324539.1.1486	Bacteria;Campylobacterota;Campylobacteria;Campylobacterales;Arcobacteraceae;uncultured;uncultured_epsilon_proteobacterium_BioIuz_K34	100	0	0	0	0.00102209	0
OTU5924	KF830684.1.1402	Bacteria;Actinobacteriota;Actinobacteria;Pseudonocardiales;Pseudonocardiaceae;Actinomycetospora;Actinomycetospora_sp._FXJ1.556	99.05	0	0.00211224	0	0	0
OTU5937	FJ820414.1.1478	Bacteria;Bacteroidota;Bacteroidia;Flavobacteriales;Crocinitomicaceae;Fluviicola;uncultured_bacterium	99.53	0.00332491	0	0	0	0
OTU594	AY948070.1.1447	Bacteria;Bacteroidota;Bacteroidia;Flavobacteriales;Crocinitomicaceae;Fluviicola;uncultured_Bacteroidetes_bacterium	99.53	0	0.00211224	0	0	0.00098733
OTU5945	FN555708.1.1502	Bacteria;Firmicutes;Bacilli;Bacillales;Planococcaceae;Chryseomicrobium_palamuruense	100	0	0.00105612	0	0.01328713	0.00098733
OTU5950	AB630759.1.1466	Bacteria;Proteobacteria;Gammaproteobacteria;Legionellales;Legionellaceae;Legionella;uncultured_bacterium	91.57	0	0.00105612	0	0	0
OTU5956	FJ543059.1.1427	Bacteria;Proteobacteria;Alphaproteobacteria;Rhodobacterales;Rhodobacteraceae;uncultured;uncultured_Amaricoccus_sp.	97.51	0.00221661	0	0	0.00408835	0
OTU5957	ET134927.1.1286	Bacteria;Patescibacteria;Gracilibacteria;Candidatus_Peribacteria;uncultured_bacterium	88.09	0	0	0.00138673	0	0
OTU596	KY356869.1.921	Archaea;Nanoarchaeota;Nanoarchaeia;Woesearchaeales;GW2011_GWC1_47_15;uncultured_archaeon	85.14	0	0.00105612	0	0.00102209	0
OTU5960	HQ166785.1.1286	Bacteria;Proteobacteria;Alphaproteobacteria;Rhodobacterales;Rhodobacteraceae;uncultured;uncultured_bacterium	98.51	0.0011083	0	0	0	0
OTU5964	FJ801182.1.1453	Bacteria;Proteobacteria;Alphaproteobacteria;Rhizobiales;A0839;uncultured_bacterium	100	0	0	0	0.00102209	0
OTU5966	AB769168.1.1477	Bacteria;Firmicutes;Bacilli;Paenibacillales;Paenibacillaceae;Paenibacillus;Paenibacillus_shirakamiensis	98.83	0	0	0	0.07665655	0
OTU5969	AB045094.1.1500	Bacteria;Firmicutes;Bacilli;Paenibacillales;Paenibacillaceae;Paenibacillus;Paenibacillus_pabuli	99.3	0	0	0.00277346	0.0643915	0
OTU5974	JF266320.1.1358	Bacteria;Proteobacteria;Gammaproteobacteria;Burkholderiales;TRA3-20;uncultured_bacterium	99.06	0	0	0.00138673	0	0
OTU5976	ATXG01000026.18916.20423	Bacteria;Actinobacteriota;Actinobacteria;Micrococcales;Microbacteriaceae;Agromyces;Agromyces_subbeticus_DSM_16689	99.28	0	0.00105612	0	0	0
OTU598	JF432053.1.1493	Bacteria;Proteobacteria;Gammaproteobacteria;Pseudomonadales;Pseudomonadaceae;Pseudomonas;Pseudomonas_formosensis	100	0.00221661	0.00422449	0.00138673	0.00204417	0.00197467
OTU5981	DQ330672.1.1380	Bacteria;Patescibacteria;WWE3;uncultured_Parcubacteria_group_bacterium	77.57	0.0011083	0	0	0.00102209	0
OTU5986	GQ231372.1.1327	Bacteria;Proteobacteria;Gammaproteobacteria;Burkholderiales;Comamonadaceae;Candidatus_Symbiobacter;uncultured_bacterium	100	0	0	0	0	0.00098733
OTU5988	JX526467.1.1435	Bacteria;Proteobacteria;Gammaproteobacteria;Pseudomonadales;Spongiibacteraceae;Oceanicoccus;uncultured_BD1-7_clade_bacterium	96.02	0	0	0.00138673	0	0
OTU5995	FN870313.1.1549	Bacteria;Verrucomicrobiota;Omnitrophia;Omnitrophales;Omnitrophaceae;Candidatus_Omnitrophus;uncultured_bacterium	95.98	0	0.00105612	0	0	0
OTU5997	JF703350.1.1419	Bacteria;Bacteroidota;Bacteroidia;Cytophagales;Microscillaceae;uncultured;uncultured_Bacteroidetes_bacterium	99.29	0	0	0	0	0.00098733
OTU6	HM243774.1.1457	Bacteria;Patescibacteria;Parcubacteria;Candidatus_Moranbacteria;uncultured_bacterium	88.67	0.01219134	0.0095051	0.01109385	0.00715461	0.00888599
OTU60	DQ981457.1.1418	Bacteria;Proteobacteria;Gammaproteobacteria;Pseudomonadales;Pseudomonadaceae;Pseudomonas;Pseudomonas_fluorescens	100	0.01440794	0.01161735	1.76530952	0.02044175	3.40827187
OTU600	MHST01000019.19787.21369	Bacteria;Patescibacteria;Parcubacteria;Candidatus_Terrybacteria;Candidatus_Terrybacteria_bacterium_RIFCSPHIGHO2_01_FTLL_58_15	89.41	0	0	0	0	0.00098733
OTU6006	JQ278805.1.1402	Bacteria;Myxococcota;Polyangia;TASB-TL25;uncultured_Nannocystineae_bacterium	89.51	0	0	0.00138673	0	0
OTU601	EF018971.1.1370	Bacteria;Patescibacteria;Parcubacteria;Candidatus_Yanofskybacteria;uncultured_bacterium	87.59	0	0.00105612	0	0	0
OTU602	FM209350.1.1508	Bacteria;Bdellovibrionota;Oligoflexia;0319-6G20;uncultured_bacterium	94.16	0	0.00105612	0	0	0
OTU6023	JF139787.1.1342	Bacteria;Acidobacteriota;Blastocatellia;Blastocatellales;Blastocatellaceae;Aridibacter;uncultured_bacterium	99.25	0	0	0.0263479	0	0.00197467
OTU6025	CP019947.1956676.1958192	Bacteria;Proteobacteria;Gammaproteobacteria;Pseudomonadales;Pseudomonadaceae;Pseudomonas;Pseudomonas_sp._CC6-YY-74	99.06	0	0	0.08043044	0.00408835	0
OTU6027	KM356249.1.1471	Bacteria;Patescibacteria;Parcubacteria;Candidatus_Nealsonbacteria;uncultured_bacterium	80.2	0.0011083	0	0	0	0
OTU6030	AM882590.1.1521	Bacteria;Desulfobacterota;Desulfobacteria;Desulfobacterales;Desulfosarcinaceae;uncultured;uncultured_delta_proteobacterium	100	0.0011083	0	0	0	0
OTU6036	AF094743.1.1494	Bacteria;Proteobacteria;Gammaproteobacteria;Pseudomonadales;Pseudomonadaceae;Pseudomonas;Pseudomonas_putida	99.77	0.0011083	0	0.04298868	0.00102209	0
OTU604	JX391257.1.1485	Bacteria;Bacteroidota;Bacteroidia;Bacteroidales;Bacteroidetes_BD2-2;uncultured_bacterium	93.6	0.00221661	0	0	0	0.00197467
OTU605	MHJF01000011.24156.25754	Bacteria;Patescibacteria;Parcubacteria;Candidatus_Colwellbacteria;Candidatus_Harrisonbacteria_bacterium_RIFCSPHIGHO2_02_FTLL_40_20	83.37	0.0011083	0	0	0.00102209	0
OTU6051	FJ482182.1.1476	Bacteria;Patescibacteria;Parcubacteria;Candidatus_Nomurabacteria;uncultured_Parcubacteria_group_bacterium	95.82	0	0.00105612	0	0	0
OTU606	AJ519621.1.1284	Bacteria;Nitrospirota;4-29-1;uncultured_bacterium	99.53	0	0	0	0.00102209	0
OTU6063	JQ278884.1.1533	Bacteria;Verrucomicrobiota;Omnitrophia;Omnitrophales;Omnitrophaceae;Candidatus_Omnitrophus;uncultured_bacterium	91.63	0.0011083	0	0	0	0
OTU6065	KC432111.1.1348	Bacteria;Bacteroidota;Bacteroidia;Flavobacteriales;Flavobacteriaceae;Flavobacterium;uncultured_bacterium	98.58	0	0	0	0	0.00098733
OTU6069	KC777199.1.1465	Bacteria;Firmicutes;Bacilli;Bacillales;Bacillaceae;Bacillus;uncultured_bacterium	100	0	0	0	0.00102209	0.00197467
OTU607	FPLS01036239.1.1232	Bacteria;Proteobacteria;Alphaproteobacteria;Rickettsiales;Mitochondria;metagenome	83.87	0	0	0	0.00102209	0
OTU6077	JN628304.1.1500	Bacteria;Proteobacteria;Gammaproteobacteria;Burkholderiales;Nitrosomonadaceae;Nitrosomonas;uncultured_bacterium	99.53	0	0	0	0.00102209	0
OTU6078	HM187361.1.1334	Bacteria;Chloroflexi;TK10;uncultured_bacterium	99.01	0.0011083	0	0	0	0
OTU6079	CAGT01000064.57.1563	Bacteria;Firmicutes;Clostridia;Peptostreptococcales-Tissierellales;Family_XI;Anaerococcus;Anaerococcus_sp._PH9	100	0	0	0	0	0.00098733
OTU608	ET642573.1.1404	Bacteria;Proteobacteria;Gammaproteobacteria;Thiotrichales;Thiotrichaceae;Thiothrix;Thiothrix_caldifontis	100	0	0.00105612	0	0.00102209	0.00197467
OTU6081	EF515953.1.1466	Bacteria;Myxococcota;bacteriap25;uncultured_bacterium	92.29	0	0	0	0	0.00098733
OTU6082	FPLS01041826.10.1476	Bacteria;Proteobacteria;Alphaproteobacteria;Rhizobiales;Xanthobacteraceae;Afipia;metagenome	100	0	0	0.00277346	0	0.00098733
OTU6083	KF836147.1.1531	Bacteria;Nitrospirota;Leptospirillia;Leptospirillales;Leptospirillaceae;Leptospirillum;uncultured_bacterium	94.63	0	0.00105612	0	0	0
OTU609	HM327751.1.1373	Bacteria;Firmicutes;Bacilli;Bacillales;Planococcaceae;Psychrobacillus;uncultured_bacterium	100	0	0	0.53250499	0.05212645	0.002962
OTU6091	AY328616.1.1511	Bacteria;Proteobacteria;Gammaproteobacteria;Salinisphaerales;Solimonadaceae;Nevskia;uncultured_bacterium	98.36	0.0011083	0	0.00138673	0	0
OTU6095	ET799624.1.1289	Bacteria;Proteobacteria;Gammaproteobacteria;Legionellales;Legionellaceae;Legionella;uncultured_bacterium	98.07	0	0	0	0.00102209	0
OTU6099	ET432426.1.1360	Bacteria;Bacteroidota;Bacteroidia;Bacteroidales;vadinHA21;uncultured_Bacteroidetes_bacterium	90.28	0	0	0	0.00102209	0
OTU610	MF942643.1.1452	Bacteria;Elusimicrobiota;Elusimicrobia;Lineage_IV;uncultured_bacterium	98.55	0.0011083	0	0	0.00102209	0
OTU6102	EF018499.1.1391	Bacteria;Verrucomicrobiota;Omnitrophia;Omnitrophales;uncultured_bacterium	88.5	0	0.00105612	0	0	0
OTU6105	HM187225.1.1504	Bacteria;Methylomirabilota;Methylomirabilia;Rokubacteriales;uncultured_bacterium	100	0	0	0	0.00102209	0
OTU6107	AM936510.1.1345	Bacteria;Actinobacteriota;Acidimicrobiia;IMCC26256;uncultured_Cellulomonadaceae_bacterium	99.5	0.0011083	0	0	0	0
OTU6109	JN672018.1.1292	Bacteria;Elusimicrobiota;Elusimicrobia;Lineage_IV;uncultured_bacterium	95.17	0	0.00105612	0	0	0
OTU611	JF163050.1.1358	Bacteria;Firmicutes;Clostridia;Peptostreptococcales-Tissierellales;Family_XI;Fenollaria;uncultured_bacterium	100	0	0	0	0.00102209	0

OTU6112	EF111264.1.1272	Bacteria;Proteobacteria;Gammaproteobacteria;Pseudomonadales;Pseudomonadaceae;Pseudomonas;gamma_proteobacterium_RBE2CD-136	99.06	0	0	0.05824273	0	0
OTU6116	ATYR01000063.8.1557	Bacteria;Firmicutes;Bacilli;Bacillales;Planococcaceae;Planomicrobium;Planomicrobium_glaciei_CHR43	100	0	0	0.00138673	0.03577306	0
OTU612	AB184058.1.1465	Bacteria;Actinobacteriota;Actinobacteria;Streptomycetales;Streptomycetaceae;Streptomyces;Streptomyces_gangtokensis	100	0.00664982	0.20488773	0.03189483	0.01226505	0
OTU6125	FPLS01032615.16.1530	Bacteria;Proteobacteria;Gammaproteobacteria;Burkholderiales;Chitinibacteraceae;Deefgea;metagenome	97.66	0.0011083	0	0	0	0
OTU6126	DQ129258.1.1414	Bacteria;Actinobacteriota;Thermoleophilia;Gaiellales;Gaiellaceae;Gaiella;uncultured_bacterium	98.83	0	0	0	0.00102209	0
OTU6128	ALWB01000102.7448.8921	Bacteria;Cyanobacteria;Cyanobacteriia;Pseudanabaenales;Pseudanabaenaceae;Pseudanabaena_PCC-7429;Pseudanabaena_biceps_PCC_7429	100	0	0	0.00138673	0	0
OTU613	MF942642.1.1390	Bacteria;Verrucomicrobiota;Omnitrophia;Omnitrophales;Omnitrophaceae;Candidatus_Omnitrophus;uncultured_bacterium	91.38	0	0	0.00138673	0	0
OTU614	AFQE01000146.4587.6115	Bacteria;Proteobacteria;Gammaproteobacteria;Burkholderiales;Neisseriaceae;Neisseria;Neisseria_macacae_ATCC_33926	100	0	0	0	0.00102209	0
OTU6146	AY038033.1.1427	Bacteria;Cyanobacteria;Cyanobacteriia;Cyanobacteriales;Nostocaceae;Nodularia_PCC-9350;Anabaenopsis_sp._PCC_9215	100	0.0011083	0	0	0	0
OTU6149	KF616728.1.1486	Bacteria;Myxococcota;bacteriap25;uncultured_bacterium	91.38	0	0	0.00138673	0	0
OTU615	JX080239.1.1485	Bacteria;Acidobacteriota;Subgroup_22;uncultured_Acidobacteriales_bacterium	96.03	0	0	0	0.00102209	0
OTU6155	ET809361.1.1344	Bacteria;Proteobacteria;Gammaproteobacteria;Burkholderiales;Rhodocyclaceae;Methyloversatilis;uncultured_bacterium	100	0	0	0	0	0.00098733
OTU6156	AB470238.1.1485	Bacteria;Firmicutes;Bacilli;Lactobacillales;Lactobacillaceae;Latilactobacillus;Lactobacillus_sakei	99.3	0.0011083	0	0	0	0
OTU6157	DQ450755.1.1317	Bacteria;Proteobacteria;Alphaproteobacteria;Reyranellales;Reyranellaceae;Reyranella;uncultured_Alphaproteobacteria_bacterium	99.5	0	0	0	0	0.00098733
OTU6161	HQ218576.1.1520	Bacteria;Verrucomicrobiota;Verrucomicrobiae;Opitutales;Opitutaceae;IMCC26134;uncultured_bacterium	97.18	0	0	0.00138673	0	0
OTU6162	JQ278816.1.1534	Bacteria;Verrucomicrobiota;Omnitrophia;Omnitrophales;Omnitrophaceae;Candidatus_Omnitrophus;uncultured_bacterium	91.86	0	0.00105612	0	0	0
OTU617	AB694470.1.1372	Bacteria;Fibrobacterota;Fibrobacteria;Fibrobacterales;B5-096;uncultured_bacterium	91.37	0.0011083	0	0.00138673	0	0
OTU6172	JQ278816.1.1534	Bacteria;Verrucomicrobiota;Omnitrophia;Omnitrophales;Omnitrophaceae;Candidatus_Omnitrophus;uncultured_bacterium	90.82	0.0011083	0	0	0	0
OTU618	KX123448.1.1621	Bacteria;Patescibacteria;Parcubacteria;Candidatus_Azambacteria;Parcubacteria_group_bacterium_GW2011_GWB1_40_14	75.91	0	0	0.00138673	0	0.00098733
OTU6184	HQ178748.1.1457	Bacteria;Proteobacteria;Gammaproteobacteria;Burkholderiales;Comamonadaceae;uncultured;uncultured_bacterium	100	0	0.00105612	0.00554693	0.00102209	0
OTU6185	ET937894.1.1532	Bacteria;Verrucomicrobiota;Omnitrophia;Omnitrophales;Omnitrophaceae;Candidatus_Omnitrophus;uncultured_bacterium	96.05	0	0	0	0.00102209	0
OTU619	CP009281.5845090.5846653	Bacteria;Firmicutes;Bacilli;Paenibacillales;Paenibacillaceae;Paenibacillus;Paenibacillus_sp._FSL_R5-0345	99.77	0	0	0.00138673	0.3464876	0
OTU6195	FPLS01051976.2.1398	Bacteria;Proteobacteria;Gammaproteobacteria;PLTA13;metagenome	99.3	0	0	0.0041602	0	0
OTU6198	KY770594.1.1378	Bacteria;Proteobacteria;Alphaproteobacteria;Rhodobacterales;Rhodobacteraceae;Roseovarius;Roseovarius_sp.	98.01	0	0	0	0.00102209	0
OTU62	KF827263.1.1400	Bacteria;Proteobacteria;Alphaproteobacteria;Sphingomonadales;Sphingomonadaceae;Novosphingobium;uncultured_Alphaproteobacteria_bacterium	100	0.00886643	0.01056122	0.00693366	0.01022087	0.00987333
OTU620	ET135137.1.1400	Bacteria;Planctomycetota;vadinHA49;uncultured_bacterium	99.77	0	0	0.00138673	0	0.00098733
OTU6203	FJ444640.1.1446	Bacteria;Proteobacteria;Alphaproteobacteria;Rhizobiales;Rhizobiaceae;Shinella;uncultured_bacterium	100	0	0	0	0	0.00888599
OTU6207	FN553456.1.1494	Bacteria;Bdellovibrionota;Oligoflexia;Oligoflexales;Oligoflexaceae;uncultured;uncultured_sediment_bacterium	92.99	0	0	0	0.00102209	0
OTU6208	AB769168.1.1477	Bacteria;Firmicutes;Bacilli;Paenibacillales;Paenibacillaceae;Paenibacillus;Paenibacillus_shirakamiensis	99.06	0	0	0	0.0398614	0
OTU621	FN668191.1.1477	Bacteria;Bacteroidota;Bacteroidia;Flavobacteriales;Crocinitomicaceae;Fluviicola;uncultured_Flexibacter_sp.	100	0.00221661	0.00105612	0.00277346	0	0
OTU6214	CP009428.12031.13594	Bacteria;Firmicutes;Bacilli;Paenibacillales;Paenibacillaceae;Paenibacillus;Paenibacillus_odorifer	99.53	0	0	0.01248059	0.12776091	0
OTU6215	AB769168.1.1477	Bacteria;Firmicutes;Bacilli;Paenibacillales;Paenibacillaceae;Paenibacillus;Paenibacillus_shirakamiensis	99.06	0	0	0.056856	0.03781723	0
OTU6216	AB672242.1.1426	Bacteria;Patescibacteria;ABY1;Candidatus_Magasanikbacteria;uncultured_bacterium	92.06	0	0.00105612	0	0	0
OTU6225	JQ278801.1.1501	Bacteria;Nitrospirota;Nitrospiria;Nitrospirales;Nitrospiraceae;Nitrospira;uncultured_Nitrospira_sp.	98.8	0.0011083	0	0	0	0
OTU624	CP016769.680246.681768	Bacteria;Actinobacteriota;Actinobacteria;Frankiales;Sporichthyaceae;hgcI_clade;Candidatus_Planktophila_lacus	99.51	0	0	0.01802751	0	0
OTU6243	DQ453810.1.1496	Bacteria;Proteobacteria;Gammaproteobacteria;Pseudomonadales;Pseudomonadaceae;Pseudomonas;Pseudomonas_sp._m1(2006)	99.3	0	0.00211224	0.06378966	0	0
OTU6244	ET133244.1.1341	Bacteria;Actinobacteriota;Actinobacteria;Propionibacteriales;Nocardioidaceae;Nocardioides;uncultured_bacterium	99.26	0.0011083	0	0	0	0
OTU625	HM187432.1.1403	Bacteria;Nitrospirota;Nitrospiria;Nitrospirales;Nitrospiraceae;Nitrospira;uncultured_bacterium	86.45	0	0.00105612	0	0	0
OTU6260	AY923084.1.1237	Bacteria;Proteobacteria;Alphaproteobacteria;Acetobacterales;Acetobacteraceae;Craurococcus-Caldovatus;uncultured_bacterium	99.5	0	0	0	0.00102209	0
OTU6265	AM997330.1.1642	Bacteria;Patescibacteria;Parcubacteria;Candidatus_Portnoybacteria;uncultured_deep-sea_bacterium	91.15	0	0	0	0.00102209	0
OTU6266	JF198933.1.1362	Bacteria;Bacteroidota;Bacteroidia;Chitinophagales;Chitinophagaceae;Ferruginibacter;uncultured_bacterium	98.1	0	0	0.00138673	0	0
OTU6268	FPLS01017333.1.1416	Bacteria;Proteobacteria;Gammaproteobacteria;Gammaproteobacteria_Incertae_Sedis;Tnknown_Family;metagenome	98.36	0	0	0.00138673	0	0
OTU627	HQ692033.1.1354	Bacteria;Bacteroidota;Bacteroidia;Sphingobacteriales;ST-12K33;uncultured_Cytophagales_bacterium	89.81	0	0.00105612	0	0	0
OTU6272	ET771617.1.1415	Bacteria;Firmicutes;Bacilli;Bacillales;Planococcaceae;Lysinibacillus;uncultured_bacterium	100	0	0	0	0.00102209	0.00789866
OTU6276	DQ297980.1.1473	Bacteria;Proteobacteria;Gammaproteobacteria;Burkholderiales;Comamonadaceae;Piscinibacter;uncultured_soil_bacterium	99.53	0	0	0.00138673	0	0
OTU628	GQ500744.1.1502	Bacteria;Proteobacteria;Gammaproteobacteria;PLTA13;uncultured_bacterium	100	0.0011083	0.00105612	0.00277346	0.00102209	0
OTU6288	AM162295.1.1494	Bacteria;Firmicutes;Bacilli;Paenibacillales;Paenibacillaceae;Paenibacillus;Paenibacillus_sp._6M20	99.53	0	0	0.05962947	0.03883932	0
OTU629	KX172614.1.1403	Bacteria;Patescibacteria;Parcubacteria;Candidatus_Yanofskybacteria;uncultured_bacterium	92.52	0	0.00211224	0	0	0
OTU6292	AB614553.1.1493	Bacteria;Myxococcota;Polyangia;Haliangiales;Haliangiaceae;Haliangium;uncultured_bacterium	78.27	0.0011083	0	0	0	0
OTU6294	KJ638253.1.1340	Bacteria;Proteobacteria;Alphaproteobacteria;Rhodobacterales;Rhodobacteraceae;Palleronia-Pseudomaribius;Palleronia_abyssalis	99	0	0	0	0.00102209	0
OTU6296	FJ820373.1.1487	Bacteria;Bacteroidota;Bacteroidia;Sphingobacteriales;NS11-12_marine_group;uncultured_bacterium	100	0	0	0.00138673	0	0
OTU6299	FJ592546.1.1316	Bacteria;Proteobacteria;Alphaproteobacteria;Caulobacterales;Hyphomonadaceae;Hirschia;uncultured_bacterium	99.25	0.0011083	0	0	0	0
OTU630	FJ946566.1.1290	Bacteria;Proteobacteria;Gammaproteobacteria;Burkholderiales;Neisseriaceae;uncultured;uncultured_beta_proteobacterium	96.49	0.0011083	0.00211224	0	0	0.00098733
OTU6306	DQ448720.1.1484	Bacteria;Actinobacteriota;Actinobacteria;Propionibacteriales;Nocardioidaceae;Nocardioides;Marmoricola_sp._CNJ780_PL04	99.02	0.0011083	0	0	0	0
OTU631	AACY020058346.1121.2624	Bacteria;Bacteroidota;Bacteroidia;Chitinophagales;Chitinophagaceae;Sediminibacterium;marine_metagenome	99.76	0.00221661	0.00105612	0	0	0
OTU6310	FJ535025.1.1497	Bacteria;Proteobacteria;Gammaproteobacteria;Burkholderiales;Nitrosomonadaceae;MND1;uncultured_bacterium	98.83	0	0	0.00138673	0	0
OTU6313	FPLP01005193.1.1450	Bacteria;Proteobacteria;Gammaproteobacteria;CCD24;metagenome	99.3	0	0.00105612	0	0	0
OTU6315	KC990424.1.1439	Bacteria;Patescibacteria;Parcubacteria;TBA9983;uncultured_Parcubacteria_group_bacterium	85.89	0	0	0	0	0.00098733
OTU6321	AJ968697.1.1428	Bacteria;Proteobacteria;Alphaproteobacteria;Rhizobiales;Rhizobiaceae;Phyllobacterium;Phyllobacterium_sp._ORS_1420	100	0.01662455	0	0	0	0.00098733
OTU6323	CP021383.308567.310096	Bacteria;Actinobacteriota;Actinobacteria;Micrococcales;Promicromonosporaceae;Cellulosimicrobium;Cellulosimicrobium_cellulans	100	0	0	0	0.00102209	0
OTU6325	FPLS01034656.1.1458	Bacteria;Myxococcota;Polyangia;Polyangiales;Polyangiaceae;Polyangium;metagenome	99.06	0	0.00105612	0	0	0
OTU6327	JF697432.1.1492	Bacteria;Proteobacteria;Gammaproteobacteria;Burkholderiales;Comamonadaceae;Limnohabitans;uncultured_bacterium	99.06	0	0.00105612	0.00277346	0	0
OTU6328	AP012048.1097572.1099087	Bacteria;Campylobacterota;Campylobacteria;Campylobacterales;Arcobacteraceae;Pseudarcobacter;Arcobacter_sp._L	99.25	0	0	0	0.00102209	0
OTU6340	FPLS01023335.19.1548	Bacteria;Myxococcota;Myxococcia;Myxococcales;Myxococcaceae;P3OB-42;metagenome	97.89	0.0011083	0	0	0	0
OTU6349	MF942642.1.1390	Bacteria;Verrucomicrobiota;Omnitrophia;Omnitrophales;Omnitrophaceae;Candidatus_Omnitrophus;uncultured_bacterium	90.8	0	0.00105612	0	0	0
OTU635	DQ337063.1.1519	Bacteria;Patescibacteria;Parcubacteria;TBA9983;uncultured_bacterium	80.7	0.0011083	0	0	0	0.00098733
OTU6352	HM187069.1.1466	Bacteria;Verrucomicrobiota;Omnitrophia;Omnitrophales;uncultured_bacterium	93.68	0	0	0	0.00102209	0

OTU6355	JF830231.1.1457	Bacteria;Cyanobacteria;Cyanobacteriia;Synechococcales;Cyanobiaceae;Cyanobium_PCC-6307;bacterium_enrichment_culture_clone_188(2011)	99.5	0	0	0.00138673	0	0
OTU6356	HM822017.1.1287	Bacteria;Proteobacteria;Alphaproteobacteria;Rhodobacterales;Rhodobacteraceae;Rubellimicrobium;uncultured_bacterium	100	0	0	0.00138673	0	0
OTU636	AB022027.1.1407	Bacteria;Firmicutes;Bacilli;Lactobacillales;Carnobacteriaceae;Granulicatella;Abiotrophia_para-adiacens	100	0	0	0	0.00102209	0
OTU6367	FPLS01062885.1.1340	Bacteria;Proteobacteria;Gammaproteobacteria;Burkholderiales;Comamonadaceae;Leptothrix;metagenome	99.77	0.0011083	0.00316837	0	0	0.00098733
OTU637	ET134927.1.1286	Bacteria;Patescibacteria;Gracilibacteria;Candidatus_Peribacteria;uncultured_bacterium	88.53	0	0	0.00138673	0	0
OTU6370	KC294000.1.1428	Bacteria;Actinobacteriota;Actinobacteria;Micrococcales;Microbacteriaceae;Leifsonia;Leifsonia_sp._EB23	100	0	0	0	0.00102209	0
OTU638	JF265912.1.1363	Bacteria;Nitrospirota;Nitrospiria;Nitrospirales;Nitrospiraceae;Nitrospira;uncultured_bacterium	98.09	0	0.00211224	0	0	0
OTU6384	JNIK01000012.94.1594	Bacteria;Actinobacteriota;Actinobacteria;Frankiales;Geodermatophilaceae;Blastococcus;Blastococcus_sp._TRHD0036	100	0	0	0	0	0.00197467
OTU6396	AJ786361.1.1400	Bacteria;Proteobacteria;Alphaproteobacteria;Rhizobiales;Rhizobiaceae;Aurantimonas;Aurantimonas_coralicida	100	0.0011083	0	0	0	0
OTU64	DQ129623.1.1444	Bacteria;Proteobacteria;Alphaproteobacteria;Sphingomonadales;Sphingomonadaceae;Sphingomonas;uncultured_bacterium	100	0.01108303	0.01267347	0.01386732	0.00715461	0.00888599
OTU640	LCOT01000026.4898.6395	Bacteria;Patescibacteria;Parcubacteria;Candidatus_Jorgensenbacteria;Parcubacteria_group_bacterium_GW2011_GWA2_47_8b	83.05	0	0.00105612	0	0.00102209	0
OTU6416	JQ346769.1.1359	Bacteria;Acidobacteriota;Blastocatellia;Blastocatellales;Blastocatellaceae;Stenotrophobacter;uncultured_Acidobacteria_bacterium	99.25	0	0	0.04437542	0	0.00493666
OTU642	AP014723.1861552.1863040	Bacteria;Campylobacterota;Campylobacteria;Campylobacterales;Sulfurospirillaceae;Sulfurospirillum;Sulfurospirillum_sp._TCH001	100	0	0	0.00138673	0	0
OTU6420	HM187325.1.1504	Bacteria;Myxococcota;bacteriap25;uncultured_bacterium	96.5	0	0	0	0.00102209	0.00098733
OTU6421	JX222290.1.1490	Bacteria;Proteobacteria;Gammaproteobacteria;Burkholderiales;Oxalobacteraceae;Massilia;uncultured_bacterium	99.77	0	0	0	0	0.00098733
OTU6422	AB476277.1.1408	Bacteria;Proteobacteria;Alphaproteobacteria;Rhizobiales;Rhizobiaceae;uncultured;uncultured_bacterium	98.76	0	0.00105612	0	0	0
OTU6423	KM019985.1.1462	Bacteria;Cyanobacteria;Cyanobacteriia;Cyanobacteriales;Xenococcaceae;Pleurocapsa_PCC-7319;Stanieria_sp._PCC_7302	99.75	0.0011083	0	0	0	0
OTU643	AM997537.1.1494	Bacteria;Proteobacteria;Gammaproteobacteria;Pseudomonadales;Spongiibacteraceae;BD1-7_clade;uncultured_deep-sea_bacterium	83.14	0.0011083	0.00105612	0	0	0
OTU6433	ET512011.1.1361	Bacteria;Proteobacteria;Gammaproteobacteria;Pseudomonadales;Pseudomonadaceae;Pseudomonas;uncultured_bacterium	95.32	0.05984838	0.00105612	0	0	0
OTU6438	ET491175.1.1551	Bacteria;Verrucomicrobiota;Omnitrophia;Omnitrophales;uncultured_bacterium	92.76	0	0.00105612	0	0	0
OTU644	FPLK01000798.18.1475	Bacteria;Verrucomicrobiota;Verrucomicrobiae;uncultured;metagenome	99.76	0	0.00211224	0	0	0
OTU6443	AJ507468.1.1455	Bacteria;Actinobacteriota;Actinobacteria;Micrococcales;Microbacteriaceae;MWH-Ta3;Microbacteriaceae_bacterium_MWH-Ta3	98.77	0	0.00105612	0	0	0
OTU6447	FJ746252.1.1434	Bacteria;Chloroflexi;JG30-KF-CM66;uncultured_bacterium	98.26	0	0	0.00138673	0	0
OTU645	FPLS01047215.10.1471	Bacteria;Proteobacteria;Alphaproteobacteria;Caulobacterales;Caulobacteraceae;Phenylobacterium;metagenome	100	0.0011083	0.00105612	0	0.00102209	0.00098733
OTU6451	ET332825.1.1489	Bacteria;Actinobacteriota;Thermoleophilia;Solirubrobacterales;Solirubrobacteraceae;Solirubrobacter;Solirubrobacter_ginsenosidimutans	99.53	0.00221661	0	0	0.00204417	0
OTU6452	KY609354.1.1527	Bacteria;Myxococcota;bacteriap25;uncultured_bacterium	93.72	0.0011083	0	0	0	0
OTU6454	HQ462490.1.1255	Bacteria;Planctomycetota;Phycisphaerae;Phycisphaerales;Phycisphaeraceae;SM1A02;uncultured_Planctomycetaceae_bacterium	95.26	0.02327437	0	0	0	0
OTU6455	FPLS01035294.8.1469	Bacteria;Proteobacteria;Alphaproteobacteria;Rhizobiales;Devosiaceae;Devosia;metagenome	100	0.00332491	0	0	0	0
OTU6458	KY474374.1.1403	Bacteria;Proteobacteria;Gammaproteobacteria;Pseudomonadales;Halomonadaceae;Halomonas;Halomonas_sp.	100	0.0011083	0	0	0	0
OTU646	ET731480.1.969	Archaea;Nanoarchaeota;Nanoarchaeia;Woesearchaeales;SCGC_AAA011-D5;uncultured_euryarchaeote	88.44	0	0.00105612	0	0	0
OTU6470	HM241119.1.1412	Bacteria;Proteobacteria;Alphaproteobacteria;Sphingomonadales;Sphingomonadaceae;Sphingomonas;uncultured_Sphingomonas_sp.	99.75	0	0	0	0.00102209	0
OTU6471	JX393854.1.1480	Bacteria;Proteobacteria;Alphaproteobacteria;Sphingomonadales;Sphingomonadaceae;Sphingomicrobium;Sphingomicrobium_flavum	100	0	0	0	0	0.00098733
OTU6475	KC854830.1.1476	Bacteria;Proteobacteria;Gammaproteobacteria;Burkholderiales;Oxalobacteraceae;Massilia;Massilia_timonae	100	0	0	0	0	0.00098733
OTU6476	FJ682678.1.1396	Bacteria;Bacteroidota;Bacteroidia;Bacteroidales;Bacteroidaceae;Bacteroides;uncultured_bacterium	99.76	0	0	0.00138673	0	0
OTU6477	HQ692033.1.1354	Bacteria;Bacteroidota;Bacteroidia;Sphingobacteriales;ST-12K33;uncultured_Cytophagales_bacterium	98.1	0.0011083	0	0	0.00102209	0
OTU6478	HM186750.1.1348	Bacteria;Chloroflexi;Dehalococcoidia;SAR202_clade;uncultured_bacterium	98.51	0	0	0	0	0.00098733
OTU6479	CP000431.1001498.1003016	Bacteria;Actinobacteriota;Actinobacteria;Corynebacteriales;Nocardiaceae;Rhodococcus;Rhodococcus_jostii_RHA1	100	0	0	0.00138673	0.00102209	0.00098733
OTU648	JQ716273.1.1493	Bacteria;Bacteroidota;Bacteroidia;Flavobacteriales;Flavobacteriaceae;Marixanthomonas;uncultured_bacterium	98.1	0	0	0.00277346	0	0
OTU6481	DQ453810.1.1496	Bacteria;Proteobacteria;Gammaproteobacteria;Pseudomonadales;Pseudomonadaceae;Pseudomonas;Pseudomonas_sp._m1(2006)	99.53	0	0	0.08320391	0.00102209	0
OTU649	HG969252.1.1474	Bacteria;Proteobacteria;Gammaproteobacteria;Pseudomonadales;Marinobacteraceae;Marinobacter;Tamilnaduibacter_salinus	94.61	0.0011083	0.00105612	0	0.00102209	0.00098733
OTU6490	HQ828005.1.1458	Bacteria;Proteobacteria;Alphaproteobacteria;Rickettsiales;SM2D12;uncultured_bacterium	96.27	0.0011083	0	0	0	0
OTU6495	CT919220.1.1283	Bacteria;Proteobacteria;Alphaproteobacteria;Rhodobacterales;Rhodobacteraceae;Defluviimonas;uncultured_bacterium	100	0	0	0.00138673	0.00102209	0
OTU65	ET937837.1.1527	Bacteria;Nitrospirota;Nitrospiria;Nitrospirales;Nitrospiraceae;Nitrospira;uncultured_bacterium	100	0.00332491	0.00211224	0.00277346	0.00102209	0.002962
OTU650	KX123588.1.1480	Bacteria;Patescibacteria;Parcubacteria;Candidatus_Yanofskybacteria;Candidatus_Yanofskybacteria_bacterium_GW2011_GWC1_48_11	89	0.0011083	0.00105612	0	0	0
OTU6502	ET200680.1.1475	Bacteria;Actinobacteriota;Actinobacteria;Pseudonocardiales;Pseudonocardiaceae;Pseudonocardia;Pseudonocardia_callicarpae	100	0.0011083	0	0.00138673	0	0
OTU6504	HM186290.1.1383	Bacteria;GAL15;uncultured_bacterium	97.61	0	0.00105612	0	0	0
OTU6505	HM187047.1.1427	Bacteria;Verrucomicrobiota;Omnitrophia;Omnitrophales;Omnitrophaceae;Candidatus_Omnitrophus;uncultured_bacterium	93.24	0	0	0.00138673	0	0
OTU651	AF293012.1.1505	Bacteria;Nitrospirota;Nitrospiria;Nitrospirales;Nitrospiraceae;Nitrospira;uncultured_Green_Bay_ferromanganous_micronodule_bacterium_MNF8	99.28	0	0	0.00138673	0	0
OTU6518	MGSW01000083.11045.12608	Bacteria;Myxococcota;bacteriap25;Deltaproteobacteria_bacterium_RIFOXYA2_FTLL_55_11	96.5	0	0.00105612	0	0	0
OTU653	AY429720.1.1337	Bacteria;Proteobacteria;Gammaproteobacteria;Burkholderiales;Comamonadaceae;Methylibium;beta_proteobacterium_HTCC304	100	0	0.0095051	0.00970712	0.00102209	0.00098733
OTU6533	AB273732.1.1489	Bacteria;Proteobacteria;Gammaproteobacteria;Enterobacterales;Enterobacteriaceae;Escherichia-Shigella;Shigella_sonnei	99.77	0	0	0	0.00102209	0
OTU6539	EF444654.1.1254	Archaea;Nanoarchaeota;Nanoarchaeia;Woesearchaeales;uncultured_archaeon	77.09	0	0	0.00138673	0	0
OTU6542	EF111099.1.1270	Bacteria;Bacteroidota;Bacteroidia;Flavobacteriales;Flavobacteriaceae;Flavobacterium;uncultured_Bacteroidetes_bacterium	99.05	0	0	0	0	0.00098733
OTU6544	HQ132471.1.1573	Bacteria;Desulfobacterota;uncultured;uncultured_Desulfuromonadales_bacterium	99.77	0	0.00211224	0.00138673	0	0
OTU6545	AY734243.1.1346	Bacteria;Proteobacteria;Gammaproteobacteria;Coxiellales;Coxiellaceae;Coxiella;uncultured_bacterium	96.72	0	0	0	0.00102209	0
OTU6548	KM356614.1.1480	Bacteria;Verrucomicrobiota;Omnitrophia;Omnitrophales;uncultured_bacterium	87.35	0	0	0	0	0.00098733
OTU655	KC189739.1.1449	Bacteria;Proteobacteria;Alphaproteobacteria;Sphingomonadales;Sphingomonadaceae;Rhizorhapis;uncultured_bacterium	100	0.00443321	0	0	0.00102209	0
OTU6555	KX123338.1.3317	Archaea;Thermoplasmatota;Thermoplasmata;uncultured;Candidatus_Amesbacteria_bacterium_GW2011_GWC1_47_15	88.24	0	0	0	0.00102209	0
OTU656	AY454638.1.1047	Archaea;Nanoarchaeota;Nanoarchaeia;Woesearchaeales;uncultured_crenarchaeote	83.51	0	0.00211224	0	0	0
OTU6561	ET626638.1.1360	Bacteria;Bacteroidota;Bacteroidia;Bacteroidales;Marinifilaceae;uncultured;uncultured_Bacteroidetes_bacterium	98.58	0	0	0	0	0.00098733
OTU6567	HF968573.1.1413	Bacteria;Proteobacteria;Gammaproteobacteria;Burkholderiales;Comamonadaceae;Limnohabitans;uncultured_Limnohabitans_sp.	100	0.0011083	0	0	0	0
OTU6568	GT127207.1.1249	Bacteria;Proteobacteria;Gammaproteobacteria;Methylococcales;Methylococcaceae;uncultured;uncultured_bacterium	99.77	0	0.00105612	0	0	0
OTU6570	KC189705.1.1504	Bacteria;Proteobacteria;Gammaproteobacteria;Burkholderiales;Methylophilaceae;Methylotenera;uncultured_bacterium	99.77	0	0	0.00138673	0	0
OTU6576	JQ278778.1.1496	Bacteria;Planctomycetota;Pla4_lineage;uncultured_bacterium	82.22	0	0	0	0.00102209	0
OTU6579	AB022873.1.1516	Bacteria;Actinobacteriota;Actinobacteria;Streptomycetales;Streptomycetaceae;Streptomyces;Kitasatospora_paracochleata	100	0.02438268	0.00105612	0	0.00919879	0
OTU658	HQ864096.1.1515	Bacteria;Firmicutes;Bacilli;Bacillales;Bacillaceae;Bacillus;uncultured_bacterium	100	0	0	0.00138673	0.00511044	0.00098733
OTU6583	KT223251.1.1480	Bacteria;Proteobacteria;Gammaproteobacteria;JTB23;uncultured_bacterium	85.28	0	0.00105612	0	0	0

OTU6589	FQ659407.1.1359	Bacteria;Proteobacteria;Gammaproteobacteria;Burkholderiales;Gallionellaceae;Sideroxydans;uncultured_soil_bacterium	98.83	0.0011083	0	0	0	0.00098733
OTU659	DQ337063.1.1519	Bacteria;Patescibacteria;Parcubacteria;TBA9983;uncultured_bacterium	80.05	0.00221661	0	0	0	0
OTU6590	FPLS01031084.20.1521	Bacteria;Acidobacteriota;Blastocatellia;Blastocatellales;Blastocatellaceae;uncultured;metagenome	100	0.0011083	0.00211224	0.02912137	0.00511044	0
OTU6596	DQ158116.1.1503	Bacteria;Proteobacteria;Gammaproteobacteria;Burkholderiales;Comamonadaceae;Acidovorax;uncultured_bacterium	100	0	0.00105612	0	0	0.01974665
OTU66	FJ184385.1.1384	Bacteria;Cyanobacteria;Cyanobacteriia;Cyanobacteriales;Phormidiaceae;Tychonema_CCAP_1459-11B;Tychonema_bourrellyi_HAB663	99.75	0.0011083	0.00739286	0.18582205	0.00511044	0.00197467
OTU6600	AB369333.1.1275	Bacteria;Proteobacteria;Gammaproteobacteria;Pseudomonadales;Pseudomonadaceae;Pseudomonas;Pseudomonas_sp._GmFRB018	99.3	0.01219134	0.00422449	0.19136898	0.00102209	0.09280926
OTU6602	AY293856.1.1440	Bacteria;Spirochaetota;Leptospirae;Leptospirales;Leptospiraceae;Turneriella;Turneriella_parva	100	0.0011083	0	0	0	0
OTU661	CP013943.1831760.1833315	Bacteria;Firmicutes;Bacilli;Staphylococcales;Staphylococcaceae;Staphylococcus;Staphylococcus_epidermidis	100	0	0.00211224	0	0.00102209	0.00098733
OTU662	HM187111.1.1482	Bacteria;Myxococcota;bacteriap25;uncultured_bacterium	99.53	0.00221661	0.00105612	0.00277346	0	0.00098733
OTU6621	FPLP01001422.16.1529	Bacteria;Proteobacteria;Gammaproteobacteria;Burkholderiales;Comamonadaceae;Rhodoferax;metagenome	99.77	0	0.00105612	0	0.00102209	0.00098733
OTU6627	JN128637.1.1414	Bacteria;Proteobacteria;Gammaproteobacteria;Burkholderiales;Comamonadaceae;Ideonella;Aquincola_sp._THE-49	99.77	0	0.00105612	0.00138673	0	0
OTU663	MNVX01000029.4758.6202	Archaea;Nanoarchaeota;Nanoarchaeia;Woesearchaeales;GW2011_GWC1_47_15;Candidatus_Pacearchaeota_archaeon_CG1_02_32_21	82.8	0	0.00105612	0	0	0
OTU6630	AM295338.1.1398	Bacteria;Actinobacteriota;Actinobacteria;Propionibacteriales;Nocardioidaceae;Marmoricola;Marmoricola_aequoreus	99.26	0.0011083	0	0	0	0.00098733
OTU6631	CT926815.1.1310	Bacteria;Proteobacteria;Alphaproteobacteria;Sphingomonadales;Sphingomonadaceae;uncultured;uncultured_bacterium	100	0	0.00105612	0	0	0.00098733
OTU6632	FJ484483.1.1366	Bacteria;Proteobacteria;Gammaproteobacteria;Beggiatoales;Beggiatoaceae;uncultured;uncultured_proteobacterium	96.49	0	0	0.00138673	0	0
OTU6636	LCDI01000005.1.1381	Bacteria;Patescibacteria;Parcubacteria;Candidatus_Azambacteria;Candidatus_Azambacteria_bacterium_GW2011_GWF2_42_22	88.85	0	0.00105612	0	0	0
OTU664	KX172805.1.1397	Bacteria;Patescibacteria;Gracilibacteria;JGI_0000069-P22;uncultured_bacterium	97.01	0.0011083	0	0	0	0.00098733
OTU6641	MF942653.1.1445	Bacteria;Verrucomicrobiota;Omnitrophia;Omnitrophales;Omnitrophaceae;Candidatus_Omnitrophus;uncultured_bacterium	92.76	0	0.00105612	0	0	0
OTU6645	ET937860.1.1431	Bacteria;Patescibacteria;Gracilibacteria;uncultured_bacterium	98.01	0	0	0.00138673	0	0
OTU665	JX223750.1.1401	Bacteria;Bacteroidota;Bacteroidia;Bacteroidales;Rs-E47_termite_group;GWE2-42-42;uncultured_bacterium	90.28	0.0011083	0.00105612	0	0.00102209	0
OTU6651	HM459717.1.1449	Bacteria;Bacteroidota;Bacteroidia;Chitinophagales;Chitinophagaceae;Aurantisolimonas;uncultured_bacterium	99.76	0.0011083	0	0	0.00102209	0
OTU6654	AB257649.1.1487	Bacteria;Acidobacteriota;Acidobacteriae;Bryobacterales;Bryobacteraceae;Bryobacter;uncultured_Acidobacterium_sp.	99.75	0.01219134	0	0.01525405	0	0.00098733
OTU6659	FJ710714.1.1473	Bacteria;Armatimonadota;Fimbriimonadia;Fimbriimonadales;uncultured_bacterium	92.82	0	0.00105612	0	0	0
OTU666	GQ340146.1.1393	Bacteria;Bacteroidota;Bacteroidia;Flavobacteriales;Crocinitomicaceae;Fluviicola;uncultured_bacterium	100	0.0011083	0	0	0	0.00098733
OTU6660	JN038700.1.1516	Bacteria;RCP2-54;uncultured_delta_proteobacterium	98.83	0.00221661	0	0	0	0
OTU6664	AM991246.1.1419	Bacteria;Proteobacteria;Alphaproteobacteria;Rickettsiales;Mitochondria;uncultured_bacterium	95.77	0	0.00105612	0.00138673	0	0
OTU6672	KC424740.1.1520	Bacteria;Patescibacteria;Parcubacteria;Candidatus_Nomurabacteria;uncultured_bacterium	92.36	0	0	0.00138673	0.00102209	0
OTU668	HM267137.1.1387	Bacteria;Patescibacteria;Saccharimonadia;Saccharimonadales;LWQ8;uncultured_bacterium	98.76	0	0.00105612	0	0	0
OTU6687	KC331282.1.1529	Bacteria;Verrucomicrobiota;Omnitrophia;Omnitrophales;Omnitrophaceae;Candidatus_Omnitrophus;uncultured_bacterium	84.87	0	0.00105612	0	0	0
OTU6688	KY287384.1.1520	Bacteria;Myxococcota;Polyangia;Polyangiales;Sandaracinaceae;uncultured;uncultured_bacterium	82.29	0	0	0	0	0.00098733
OTU6690	KX504671.1.1497	Bacteria;Proteobacteria;Gammaproteobacteria;Burkholderiales;Comamonadaceae;Rubrivivax;uncultured_bacterium	99.3	0	0	0.00138673	0	0
OTU6697	JN003096.1.1297	Bacteria;Actinobacteriota;Actinobacteria;Propionibacteriales;Nocardioidaceae;uncultured;uncultured_actinobacterium	98.28	0.01551625	0	0	0.00511044	0
OTU6698	JQ977347.1.1428	Bacteria;Proteobacteria;Gammaproteobacteria;Burkholderiales;Oxalobacteraceae;Janthinobacterium;Janthinobacterium_sp._Ata9	99.77	0	0	0	0.00102209	0.00098733
OTU67	JX097018.1.1439	Bacteria;Proteobacteria;Gammaproteobacteria;Burkholderiales;Oxalobacteraceae;Massilia;Massilia_sp._C1804	99.77	0.01219134	0.01795408	0.00693366	0.01124296	0.00691133
OTU6710	HM126920.1.1446	Bacteria;Bacteroidota;Bacteroidia;Cytophagales;Cyclobacteriaceae;uncultured;uncultured_bacterium	100	0	0	0.00277346	0	0
OTU6713	HE603186.1.1453	Bacteria;Verrucomicrobiota;Omnitrophia;Omnitrophales;Omnitrophaceae;Candidatus_Omnitrophus;uncultured_Firmicutes_bacterium	92.54	0.0011083	0	0	0	0
OTU672	MEPS01000173.1634.3193	Bacteria;Bdellovibrionota;Oligoflexia;0319-6G20;Bdellovibrionales_bacterium_GWC1_52_8	93.93	0.00221661	0.00105612	0	0	0
OTU6720	KC003075.1.1352	Bacteria;Planctomycetota;Phycisphaerae;Phycisphaerales;Phycisphaeraceae;SM1A02;unidentified_marine_bacterioplankton	99.75	0	0.00105612	0	0	0
OTU675	FPLK01003065.12.1502	Bacteria;Bacteroidota;Bacteroidia;Chitinophagales;Chitinophagaceae;Dinghuibacter;metagenome	99.76	0	0.00211224	0	0	0
OTU678	KX123463.1.1595	Bacteria;Patescibacteria;Parcubacteria;Candidatus_Nomurabacteria;Candidatus_Nomurabacteria_bacterium_GW2011_GWE1_32_28	93.09	0	0	0.00138673	0	0
OTU68	AB102732.157.1651	Bacteria;Proteobacteria;Alphaproteobacteria;Rhizobiales;Rhizobiaceae;Allorhizobium-Neorhizobium-Pararhizobium-Rhizobium;Agrobacterium_radiobacter	100	0.00332491	0.00844898	0.0041602	0.00919879	0.00888599
OTU681	HM856418.1.1455	Bacteria;Proteobacteria;Gammaproteobacteria;Burkholderiales;Burkholderiaceae;Polynucleobacter;uncultured_Polynucleobacter_sp.	99.77	0.00221661	0.00105612	0.00277346	0.00204417	0.00098733
OTU682	AF132933.1.1409	Bacteria;Cyanobacteria;Cyanobacteriia;Cyanobacteriales;Phormidiaceae;Trichodesmium_IMS101;Oscillatoria_sancta_PCC_7515	99.5	0	0	0.01525405	0	0
OTU683	AF367482.1.1328	Bacteria;Patescibacteria;Gracilibacteria;JGI_0000069-P22;uncultured_Epsilonproteobacteria_bacterium	98.26	0	0	0	0	0.00098733
OTU686	KR868710.1.1452	Bacteria;Bacteroidota;Bacteroidia;Flavobacteriales;Flavobacteriaceae;Gramella;Gramella_aquimixticola	95.97	0	0.00211224	0	0	0
OTU687	CT927689.1.1322	Bacteria;Cyanobacteria;Vampirivibrionia;Obscuribacterales;Obscuribacteraceae;Candidatus_Obscuribacter;uncultured_bacterium	100	0	0	0.00138673	0	0
OTU688	DQ190078.1.919	Archaea;Crenarchaeota;Nitrososphaeria;Nitrosopumilales;Nitrosopumilaceae;uncultured_archaeon	99.74	0	0.00105612	0	0	0
OTU69	ARFT01000016.358.1875	Bacteria;Proteobacteria;Gammaproteobacteria;Pseudomonadales;Moraxellaceae;Acinetobacter;Acinetobacter_tjernbergiae_DSM_14971_=_CIP_107465	100	0.01108303	0.0095051	0.00832039	0.01124296	0.00987333
OTU690	KY356875.1.908	Archaea;Nanoarchaeota;Nanoarchaeia;Woesearchaeales;uncultured_archaeon	82.57	0	0	0	0	0.00098733
OTU691	HQ224601.1.1426	Bacteria;Proteobacteria;Gammaproteobacteria;Pseudomonadales;Pseudomonadaceae;Pseudomonas;Pseudomonas_sp._SGb186	99.77	0.00221661	0.00316837	0.56856002	0	0.00197467
OTU692	DQ640710.1.1339	Bacteria;Proteobacteria;Alphaproteobacteria;Rhodospirillales;uncultured;uncultured_Alphaproteobacteria_bacterium	99.5	0	0	0.00138673	0.00204417	0
OTU695	FJ612277.1.1480	Bacteria;Proteobacteria;Gammaproteobacteria;Burkholderiales;MWH-TniP1_aquatic_group;uncultured_bacterium	100	0.0011083	0.00211224	0.00277346	0.00102209	0
OTU696	AY193168.1.1312	Bacteria;Fusobacteriota;Fusobacteriia;Fusobacteriales;Leptotrichiaceae;Hypnocyclicus;uncultured_Fusobacteria_bacterium	98.77	0	0	0.00138673	0	0
OTU697	LC081118.1.1457	Bacteria;Patescibacteria;Parcubacteria;Candidatus_Portnoybacteria;uncultured_bacterium	87.97	0	0	0	0.00102209	0
OTU698	FJ192729.1.1480	Bacteria;Bacteroidota;Bacteroidia;Sphingobacteriales;Sphingobacteriaceae;Nubsella;uncultured_bacterium	100	0	0	0	0	0.00098733
OTU699	ET803844.1.1507	Bacteria;Bdellovibrionota;Oligoflexia;0319-6G20;uncultured_bacterium	93.69	0	0	0	0	0.00098733
OTU7	HM124374.1.1400	Bacteria;Proteobacteria;Gammaproteobacteria;Burkholderiales;Rhodocyclaceae;Ferribacterium;Ferribacterium_sp._24-19	100	0.04433214	0.04858163	0.04576215	0.04088349	0.04442996
OTU70	KT179367.1.1485	Bacteria;Proteobacteria;Gammaproteobacteria;Enterobacterales;Enterobacteriaceae;Buttiauxella;Buttiauxella_brennerae	99.77	4.5518021	0.01056122	0.00832039	0.00919879	0.00493666
OTU700	JX222018.1.1446	Bacteria;Proteobacteria;Alphaproteobacteria;Caulobacterales;Caulobacteraceae;Caulobacter;uncultured_bacterium	100	0.0011083	0	0	0.00102209	0
OTU703	AB294345.1.1480	Bacteria;Verrucomicrobiota;Omnitrophia;Omnitrophales;Omnitrophaceae;Candidatus_Omnitrophus;uncultured_bacterium	94.87	0	0	0	0.00102209	0
OTU708	HE603186.1.1453	Bacteria;Verrucomicrobiota;Omnitrophia;Omnitrophales;Omnitrophaceae;Candidatus_Omnitrophus;uncultured_Firmicutes_bacterium	90.21	0	0	0.00138673	0.00102209	0
OTU709	KY241517.1.1472	Bacteria;Chloroflexi;Dehalococcoidia;SAR202_clade;uncultured_bacterium	97.77	0.0011083	0	0	0	0
OTU71	AB286421.1.1403	Bacteria;Planctomycetota;Phycisphaerae;Phycisphaerales;Phycisphaeraceae;SM1A02;uncultured_bacterium	96.27	0	0.00105612	0.00138673	0	0.00098733
OTU710	GQ500746.1.1487	Bacteria;Nitrospirota;Nitrospiria;Nitrospirales;Nitrospiraceae;Nitrospira;uncultured_bacterium	99.52	0	0.00105612	0	0.00102209	0.00098733
OTU712	EF018971.1.1370	Bacteria;Patescibacteria;Parcubacteria;Candidatus_Yanofskybacteria;uncultured_bacterium	88.52	0	0	0.00277346	0	0
OTU713	FN668214.1.1474	Bacteria;Actinobacteriota;Actinobacteria;Micrococcales;Microbacteriaceae;Rhodoluna;uncultured_actinobacterium	100	0	0.00105612	0.01525405	0	0.002962
OTU717	KJ566499.1.944	Archaea;Nanoarchaeota;Nanoarchaeia;Woesearchaeales;uncultured_euryarchaeote	94.44	0	0	0	0	0.00098733

OTU719	JX105608.1.1495	Bacteria;Proteobacteria;Gammaproteobacteria;Burkholderiales;Neisseriaceae;uncultured;uncultured_bacterium	94.15	0	0.00105612	0	0.00102209	0.00098733
OTU72	AM934647.1.1486	Bacteria;Bacteroidota;Bacteroidia;Flavobacteriales;Flavobacteriaceae;Flavobacterium;Flavobacterium_sp._WB2.3-46	100	0.00221661	0.02534694	0.00554693	0.00204417	3.0538195
OTU721	MHGG01000050.25339.26910	Bacteria;Verrucomicrobiota;Omnitrophia;Omnitrophales;Omnitrophaceae;Candidatus_Omnitrophus;Omnitrophica_WOR_2_bacterium_GWF2_63_9	90.21	0.0011083	0	0	0	0.00098733
OTU722	HE603186.1.1453	Bacteria;Verrucomicrobiota;Omnitrophia;Omnitrophales;Omnitrophaceae;Candidatus_Omnitrophus;uncultured_Firmicutes_bacterium	91.67	0	0	0.00138673	0	0
OTU723	MHJF01000011.24156.25754	Bacteria;Patescibacteria;Parcubacteria;Candidatus_Colwellbacteria;Candidatus_Harrisonbacteria_bacterium_RIFCSPHIGHO2_02_FTLL_40_20	85.11	0.0011083	0	0.00138673	0.00102209	0
OTU724	AM261868.1.1483	Bacteria;Bacteroidota;Bacteroidia;Flavobacteriales;Weeksellaceae;Chryseobacterium;Chryseobacterium_hominis	100	0.0011083	0	0.00138673	0	0
OTU725	HM187432.1.1403	Bacteria;Nitrospirota;Nitrospiria;Nitrospirales;Nitrospiraceae;Nitrospira;uncultured_bacterium	86.45	0.0011083	0	0	0	0
OTU728	DQ223087.1.1346	Bacteria;Bdellovibrionota;Bdellovibrionia;Bdellovibrionales;Bdellovibrionaceae;OM27_clade;uncultured_bacterium	94.07	0	0	0	0.00102209	0
OTU73	FJ820478.1.1481	Bacteria;Actinobacteriota;Actinobacteria;Micrococcales;Microbacteriaceae;Candidatus_Limnoluna;uncultured_bacterium	99.75	0.01108303	0.01161735	0.04853561	0.00613252	0.005924
OTU730	ET731166.1.1223	Archaea;Nanoarchaeota;Nanoarchaeia;Woesearchaeales;uncultured_euryarchaeote	86.27	0.0011083	0	0	0	0
OTU731	AAJM01000140.1744.3228	Bacteria;Firmicutes;Bacilli;Bacillales;Bacillaceae;Bacillus;Bacillus_thuringiensis_serovar_israelensis_ATCC_35646	99.77	0.0011083	0.00211224	0	0	0
OTU732	AY454638.1.1047	Archaea;Nanoarchaeota;Nanoarchaeia;Woesearchaeales;uncultured_crenarchaeote	79.43	0	0.00105612	0	0	0
OTU733	GQ358806.1.1469	Bacteria;Actinobacteriota;Actinobacteria;Corynebacteriales;Dietziaceae;Dietzia;uncultured_bacterium	99.26	0	0	0.00138673	0.00102209	0
OTU734	KC990421.1.1249	Bacteria;Patescibacteria;Parcubacteria;Candidatus_Azambacteria;uncultured_Parcubacteria_group_bacterium	92.59	0.0011083	0.00105612	0	0	0
OTU736	GQ397003.1.1431	Bacteria;Proteobacteria;Alphaproteobacteria;Rhodobacterales;Rhodobacteraceae;uncultured;uncultured_bacterium	99.5	0	0.00105612	0	0	0
OTU738	LCDF01000001.64365.66805	Bacteria;Patescibacteria;Parcubacteria;Candidatus_Giovannonibacteria;Candidatus_Giovannonibacteria_bacterium_GW2011_GWF2_42_19	85.4	0	0.00211224	0	0.00102209	0
OTU74	CP015603.920030.921522	Bacteria;Actinobacteriota;Actinobacteria;Frankiales;Sporichthyaceae;hgcI_clade;Actinobacteria_bacterium_IMCC25003	100	0.00664982	0.00633673	0.09013756	0.00511044	0.002962
OTU740	KF836295.1.1480	Bacteria;Elusimicrobiota;Elusimicrobia;Lineage_IV;uncultured_bacterium	95.65	0	0.00105612	0.00138673	0	0
OTU742	MHGG01000050.25339.26910	Bacteria;Verrucomicrobiota;Omnitrophia;Omnitrophales;Omnitrophaceae;Candidatus_Omnitrophus;Omnitrophica_WOR_2_bacterium_GWF2_63_9	94.64	0	0.00105612	0.00138673	0	0.00098733
OTU743	KC295710.1.1399	Bacteria;Patescibacteria;Saccharimonadia;Saccharimonadales;uncultured_bacterium	97.03	0	0.00211224	0	0	0
OTU744	KJ782238.1.1518	Bacteria;Proteobacteria;Gammaproteobacteria;Burkholderiales;Nitrosomonadaceae;MND1;uncultured_bacterium	98.83	0	0.00105612	0	0	0.00197467
OTU745	KY356865.1.937	Archaea;Nanoarchaeota;Nanoarchaeia;Woesearchaeales;GW2011_GWC1_47_15;uncultured_archaeon	89.91	0	0.00105612	0	0.00102209	0
OTU748	HM187450.1.1355	Bacteria;Verrucomicrobiota;Omnitrophia;Omnitrophales;Omnitrophaceae;Candidatus_Omnitrophus;uncultured_bacterium	93.95	0	0	0	0	0.00098733
OTU75	FJ535179.1.1478	Bacteria;Bacteroidota;Bacteroidia;Flavobacteriales;Flavobacteriaceae;Flavobacterium;uncultured_Flavobacteriia_bacterium	99.76	0	0.00105612	3.67899933	0.00204417	0.02369598
OTU750	ET335170.1.1510	Bacteria;Actinobacteriota;Thermoleophilia;Gaiellales;Gaiellaceae;Gaiella;uncultured_bacterium	99.53	0.0011083	0	0	0	0.00098733
OTU752	JX000953.1.1397	Bacteria;Patescibacteria;Parcubacteria;Candidatus_Yanofskybacteria;uncultured_bacterium	78.91	0	0.00211224	0	0	0.00098733
OTU753	AB680997.1.1412	Bacteria;Proteobacteria;Alphaproteobacteria;Sphingomonadales;Sphingomonadaceae;Sphingomonas;Sphingomonas_sp._NBRC_15914	100	0.00332491	0.00105612	0.00277346	0.00102209	0.00098733
OTU756	JF703317.1.1434	Bacteria;Proteobacteria;Gammaproteobacteria;Acidiferrobacterales;Acidiferrobacteraceae;Sulfurifustis;uncultured_proteobacterium	99.53	0.00332491	0.00105612	0.00138673	0.00102209	0.00098733
OTU757	HQ190571.1.1494	Bacteria;Gemmatimonadota;Gemmatimonadetes;Gemmatimonadales;Gemmatimonadaceae;uncultured;uncultured_bacterium	98.09	0.0011083	0	0	0.00102209	0
OTU758	KC358031.1.1241	Bacteria;Chloroflexi;Dehalococcoidia;SAR202_clade;uncultured_bacterium	97.77	0.0011083	0.00105612	0	0	0
OTU76	JN474863.1.1220	Bacteria;Patescibacteria;Parcubacteria;Candidatus_Kaiserbacteria;uncultured_organism	95.07	0	0.00316837	0.00138673	0	0.00098733
OTU760	KP686648.1.1428	Bacteria;Patescibacteria;Parcubacteria;uncultured_bacterium	83.59	0.0011083	0.00105612	0.00138673	0	0
OTU761	AY947931.1.1449	Bacteria;Bacteroidota;Bacteroidia;Chitinophagales;Chitinophagaceae;Edaphobaculum;uncultured_Bacteroidetes_bacterium	100	0	0	0.00138673	0	0.00098733
OTU762	ET133981.1.1315	Bacteria;Chloroflexi;P2-11E;uncultured_bacterium	99.75	0.0011083	0	0	0	0.00098733
OTU763	JX521689.1.1518	Bacteria;Proteobacteria;Gammaproteobacteria;Thiomicrospirales;Thiomicrospiraceae;uncultured;uncultured_bacterium	99.3	0.00221661	0.00105612	0	0	0.00098733
OTU764	EF032738.1.1406	Bacteria;Proteobacteria;Alphaproteobacteria;Rickettsiales;SM2D12;uncultured_Alphaproteobacteria_bacterium	97.76	0	0	0.00138673	0	0.00098733
OTU765	KC331538.1.1517	Bacteria;Bdellovibrionota;Oligoflexia;0319-6G20;uncultured_bacterium	95.33	0	0.00105612	0	0	0
OTU767	AP014879.2384902.2386420	Bacteria;Proteobacteria;Gammaproteobacteria;Acidiferrobacterales;Acidiferrobacteraceae;Sulfurifustis;Sulfuricaulis_limicola	98.36	0	0	0.00277346	0	0.00098733
OTU77	ABYQ02000004.752.2257	Bacteria;Actinobacteriota;Actinobacteria;Corynebacteriales;Corynebacteriaceae;Corynebacterium;Corynebacterium_pseudogenitalium_ATCC_33035	100	0.00221661	0.00528061	0.00832039	0.00408835	0.002962
OTU770	KC424740.1.1520	Bacteria;Patescibacteria;Parcubacteria;Candidatus_Nomurabacteria;uncultured_bacterium	88.92	0	0	0	0.00102209	0
OTU772	CT920214.1.1285	Bacteria;Proteobacteria;Alphaproteobacteria;Rhodobacterales;Rhodobacteraceae;Tabrizicola;uncultured_bacterium	99.25	0	0	0.00138673	0	0.00197467
OTU773	AB255084.1.1423	Bacteria;Proteobacteria;Alphaproteobacteria;Rhodobacterales;Rhodobacteraceae;Pseudorhodobacter;uncultured_bacterium	100	0.0011083	0	0	0	0
OTU774	AB073364.1.1508	Bacteria;Firmicutes;Bacilli;Paenibacillales;Paenibacillaceae;Paenibacillus;Paenibacillus_borealis	99.77	0	0	0.19414245	0.2412126	0
OTU777	MF942639.1.1600	Bacteria;Verrucomicrobiota;Omnitrophia;Omnitrophales;uncultured_bacterium	93.69	0	0	0	0	0.00098733
OTU778	JX223750.1.1401	Bacteria;Bacteroidota;Bacteroidia;Bacteroidales;Rs-E47_termite_group;GWE2-42-42;uncultured_bacterium	91.23	0.0011083	0	0.00138673	0	0
OTU78	ET800157.1.1455	Bacteria;Actinobacteriota;Acidimicrobiia;Microtrichales;Ilumatobacteraceae;CL500-29_marine_group;uncultured_bacterium	100	0.00664982	0.01161735	0.00277346	0.00511044	0.002962
OTU780	AF480599.1.1458	Bacteria;Actinobacteriota;Actinobacteria;Corynebacteriales;Mycobacteriaceae;Mycobacterium;Mycobacterium_diernhoferi	100	0.00221661	0.00105612	0	0.00102209	0
OTU781	JF178876.1.1345	Bacteria;Bacteroidota;Bacteroidia;Flavobacteriales;Flavobacteriaceae;Flavobacterium;uncultured_bacterium	99.76	0	0.00316837	0.0041602	0	0.002962
OTU782	HQ782701.1.1430	Bacteria;Fusobacteriota;Fusobacteriia;Fusobacteriales;Fusobacteriaceae;Fusobacterium;uncultured_organism	89.36	0.0011083	0	0	0	0.00098733
OTU784	MF942640.1.1495	Bacteria;Verrucomicrobiota;Omnitrophia;Omnitrophales;Omnitrophaceae;Candidatus_Omnitrophus;uncultured_bacterium	91.38	0	0	0	0.00102209	0
OTU785	MF942642.1.1390	Bacteria;Verrucomicrobiota;Omnitrophia;Omnitrophales;Omnitrophaceae;Candidatus_Omnitrophus;uncultured_bacterium	92.54	0.00332491	0	0	0.00204417	0
OTU786	JX521689.1.1518	Bacteria;Proteobacteria;Gammaproteobacteria;Thiomicrospirales;Thiomicrospiraceae;uncultured;uncultured_bacterium	99.53	0.0011083	0.00105612	0	0	0
OTU787	MF942642.1.1390	Bacteria;Verrucomicrobiota;Omnitrophia;Omnitrophales;Omnitrophaceae;Candidatus_Omnitrophus;uncultured_bacterium	88.92	0	0	0	0	0.00098733
OTU789	AF361205.1.1491	Bacteria;Bacteroidota;Bacteroidia;Flavobacteriales;Crocinitomicaceae;Fluviicola;uncultured_Cytophagales_bacterium	100	0	0.00105612	0	0	0.00098733
OTU79	AF277202.1.1458	Bacteria;Actinobacteriota;Actinobacteria;Corynebacteriales;Nocardiaceae;Nocardia;Nocardia_salmonicida_subsp._cummidelens	100	0.00332491	0.01689796	0.00693366	0.00919879	0.00493666
OTU791	AB547639.1.1489	Bacteria;Firmicutes;Clostridia;Peptostreptococcales-Tissierellales;Family_XI;Ezakiella;[Bacteroides]_coagulans	100	0.0011083	0.00105612	0	0	0.00098733
OTU793	FPLS01052147.20.1490	Bacteria;Proteobacteria;Alphaproteobacteria;Rhodospirillales;Rhodospirillaceae;uncultured;metagenome	99	0.0011083	0.00105612	0	0.00204417	0
OTU796	JN481415.1.1467	Bacteria;Myxococcota;Myxococcia;Myxococcales;Myxococcaceae;P3OB-42;uncultured_organism	90.42	0	0	0.00138673	0	0
OTU797	ET703169.1.1476	Bacteria;Bacteroidota;Bacteroidia;Flavobacteriales;Flavobacteriaceae;Flavobacterium;uncultured_Flavobacterium_sp.	100	0	0	0.00138673	0	0
OTU798	ET803394.1.1462	Bacteria;Chloroflexi;SL56_marine_group;uncultured_bacterium	100	0.00332491	0	0	0	0
OTU80	AB051697.1.1445	Bacteria;Proteobacteria;Gammaproteobacteria;Pseudomonadales;Pseudomonadaceae;Pseudomonas;Pseudomonas_sp._LAB-20	100	0.12302168	0.17426019	4.83414688	0.12980509	0.01678465
OTU801	MF040478.1.1461	Bacteria;Proteobacteria;Alphaproteobacteria;Acetobacterales;Acetobacteraceae;Roseomonas;uncultured_bacterium	99.25	0.0011083	0	0	0	0
OTU802	FPLK01002034.11.1489	Bacteria;Proteobacteria;Alphaproteobacteria;NRL2;metagenome	99.75	0.0011083	0	0	0	0
OTU803	AY835924.1.1476	Bacteria;Actinobacteriota;Actinobacteria;Propionibacteriales;Nocardioidaceae;Nocardioides;Nocardioides_kribbensis	100	0	0.00105612	0	0	0.00098733
OTU804	KR052005.1.1385	Bacteria;Proteobacteria;Alphaproteobacteria;Rhodobacterales;Rhodobacteraceae;Paracoccus;Paracoccus_angustae	100	0	0.00105612	0	0	0.00098733
OTU805	HM187445.1.1351	Bacteria;Patescibacteria;Parcubacteria;Paceibacterales;Paceibacteraceae;Candidatus_Paceibacter;uncultured_bacterium	80.54	0.0011083	0	0	0	0.00098733
OTU81	KP686608.1.1447	Bacteria;Bacteroidota;Bacteroidia;Flavobacteriales;Crocinitomicaceae;Fluviicola;uncultured_bacterium	99.76	0.00886643	0.01161735	0.04714888	0.00511044	0.00394933

OTU810	AF351229.1.1460	Bacteria;Proteobacteria;Gammaproteobacteria;Burkholderiales;Gallionellaceae;Sideroxydans;uncultured_beta_proteobacterium	99.53	0.0011083	0.00105612	0	0	0
OTU811	HM187111.1.1482	Bacteria;Myxococcota;bacteriap25;uncultured_bacterium	99.07	0	0	0	0.00306626	0
OTU816	KC358393.1.1301	Bacteria;Verrucomicrobiota;Omnitrophia;Omnitrophales;Omnitrophaceae;Candidatus_Omnitrophus;uncultured_bacterium	92.62	0	0	0.00138673	0	0
OTU82	FJ612391.1.1490	Bacteria;Bacteroidota;Bacteroidia;Sphingobacteriales;NS11-12_marine_group;uncultured_bacterium	100	0.0011083	0.00211224	0.00554693	0.00306626	0.00493666
OTU820	MNWW01000056.4577.6048	Archaea;Nanoarchaeota;Nanoarchaeia;Woesearchaeales;CG1-02-57-44;Candidatus_Woesearchaeota_archaeon_CG1_02_57_44	87.11	0	0	0.00138673	0	0
OTU821	AJ631246.1.967	Archaea;Nanoarchaeota;Nanoarchaeia;Woesearchaeales;uncultured_archaeon	82.23	0	0.00105612	0	0	0
OTU822	JQ675522.1.1363	Bacteria;Bdellovibrionota;Oligoflexia;0319-6G20;uncultured_bacterium	92.76	0.0011083	0	0	0	0
OTU823	AB753878.1.1447	Bacteria;Actinobacteriota;Acidimicrobiia;Microtrichales;uncultured;uncultured_bacterium	100	0	0	0	0	0.00197467
OTU825	MF942653.1.1445	Bacteria;Verrucomicrobiota;Omnitrophia;Omnitrophales;Omnitrophaceae;Candidatus_Omnitrophus;uncultured_bacterium	93.22	0	0.00105612	0	0	0
OTU826	AB273624.1.1453	Bacteria;Actinobacteriota;Actinobacteria;Propionibacteriales;Nocardioidaceae;Nocardioides;Nocardioides_exalbidus	98.53	0	0.00105612	0	0	0.00098733
OTU828	KX123607.1.1467	Bacteria;Patescibacteria;Parcubacteria;Candidatus_Yanofskybacteria;Candidatus_Yanofskybacteria_bacterium_GW2011_GWF1_44_227	79.6	0	0	0	0	0.00098733
OTU829	FJ946537.1.1290	Bacteria;Bacteroidota;Bacteroidia;Flavobacteriales;Flavobacteriaceae;Lutibacter;uncultured_Flavobacterium_sp.	98.34	0.0011083	0.00211224	0.00138673	0	0
OTU83	JN626511.1.1354	Bacteria;Bacteroidota;Bacteroidia;Sphingobacteriales;env.OPS_17;uncultured_bacterium	98.82	0.00332491	0.00528061	0.13867318	0.00102209	0.00197467
OTU830	AB166956.1.1469	Bacteria;Actinobacteriota;Rubrobacteria;Rubrobacterales;Rubrobacteriaceae;Rubrobacter;Rubrobacter_sp._CBF_L56	99.3	0.0011083	0	0	0	0.00098733
OTU831	KY356865.1.937	Archaea;Nanoarchaeota;Nanoarchaeia;Woesearchaeales;GW2011_GWC1_47_15;uncultured_archaeon	87.3	0	0.00105612	0	0	0
OTU832	MHJF01000011.24156.25754	Bacteria;Patescibacteria;Parcubacteria;Candidatus_Colwellbacteria;Candidatus_Harrisonbacteria_bacterium_RIFCSPHIGHO2_02_FTLL_40_20	88.59	0.0011083	0.00105612	0	0	0
OTU835	JF204045.1.1361	Bacteria;Actinobacteriota;Actinobacteria;Actinomycetales;Actinomycetaceae;uncultured_bacterium	99.76	0.0011083	0	0	0	0.00098733
OTU836	MHIZ01000032.6820.8219	Bacteria;Patescibacteria;Parcubacteria;Candidatus_Colwellbacteria;Candidatus_Colwellbacteria_bacterium_RIFCSPLOWO2_02_FTLL_44_20b	78.73	0	0	0	0.00102209	0
OTU838	HM186577.1.1351	Bacteria;Planctomycetota;Pla4_lineage;uncultured_bacterium	98.76	0	0	0	0.00102209	0
OTU84	AF493635.1.1300	Bacteria;Bacteroidota;Bacteroidia;Flavobacteriales;Flavobacteriaceae;Flavobacterium;Flavobacterium_sp._EP030	100	0.00221661	0	3.6471045	0.00102209	0
OTU840	AM411932.1.1430	Bacteria;Proteobacteria;Gammaproteobacteria;Burkholderiales;Comamonadaceae;Ramlibacter;Variovorax_sp._P-9	99.06	0.0011083	0	0	0	0
OTU841	FJ719059.1.1478	Bacteria;Verrucomicrobiota;Omnitrophia;Omnitrophales;Omnitrophaceae;Candidatus_Omnitrophus;uncultured_bacterium	87.09	0	0	0.00138673	0	0
OTU843	FJ482182.1.1476	Bacteria;Patescibacteria;Parcubacteria;Candidatus_Nomurabacteria;uncultured_Parcubacteria_group_bacterium	96.31	0	0	0.00138673	0	0
OTU844	HM186577.1.1351	Bacteria;Planctomycetota;Pla4_lineage;uncultured_bacterium	99.5	0	0.00105612	0	0	0
OTU847	JX227655.1.1532	Bacteria;Verrucomicrobiota;Omnitrophia;Omnitrophales;uncultured_bacterium	92.92	0	0	0	0.00102209	0
OTU848	MFTT01000025.14894.16462	Bacteria;Patescibacteria;Parcubacteria;Candidatus_Nomurabacteria;Candidatus_Nomurabacteria_bacterium_RIFCSPHIGHO2_01_FTLL_42_16	79.35	0.0011083	0	0	0	0
OTU85	JN656775.1.1496	Bacteria;Verrucomicrobiota;Verrucomicrobiae;uncultured;uncultured_Verrucomicrobia_bacterium	100	0.0011083	0.00316837	0.16224761	0.00306626	0.00098733
OTU851	LCOK01000044.9216.10662	Bacteria;Patescibacteria;Parcubacteria;Candidatus_Ryanbacteria;Candidatus_Giovannonibacteria_bacterium_GW2011_GWB1_47_6b	83.22	0	0	0	0	0.00098733
OTU852	KP054233.1.1440	Bacteria;Spirochaetota;Leptospirae;Leptospirales;Leptospiraceae;Turneriella;uncultured_bacterium	99.76	0	0	0	0.00102209	0
OTU854	MHGG01000050.25339.26910	Bacteria;Verrucomicrobiota;Omnitrophia;Omnitrophales;Omnitrophaceae;Candidatus_Omnitrophus;Omnitrophica_WOR_2_bacterium_GWF2_63_9	91.84	0	0	0.00138673	0	0
OTU855	ET461958.1.1398	Bacteria;Bacteroidota;Bacteroidia;Bacteroidales;Muribaculaceae;uncultured_bacterium	100	0	0.00105612	0	0	0
OTU856	FM872861.1.1446	Bacteria;Proteobacteria;Alphaproteobacteria;Sphingomonadales;Sphingomonadaceae;Novosphingobium;uncultured_bacterium	99.5	0	0.00211224	0.00138673	0	0
OTU857	ASEP01000145.3956.5452	Bacteria;Actinobacteriota;Actinobacteria;Propionibacteriales;Nocardioidaceae;Nocardioides;Nocardioides_sp._CF8	100	0	0.00211224	0	0.00408835	0
OTU859	HM187366.1.1347	Bacteria;Chloroflexi;Dehalococcoidia;SAR202_clade;uncultured_bacterium	99.01	0	0.00105612	0	0	0
OTU86	ET594555.1.1445	Bacteria;Proteobacteria;Gammaproteobacteria;Pseudomonadales;Pseudomonadaceae;Pseudomonas;Pseudomonas_fulva	99.77	0.04322383	0.09399489	5.41657422	0.10629708	0.01382266
OTU860	KY356869.1.921	Archaea;Nanoarchaeota;Nanoarchaeia;Woesearchaeales;GW2011_GWC1_47_15;uncultured_archaeon	86.88	0.0011083	0	0	0	0
OTU862	LC026902.1.1415	Bacteria;Cyanobacteria;Cyanobacteriia;Cyanobacteriales;uncultured;uncultured_bacterium	99.75	0	0.00105612	0	0.00102209	0
OTU863	AJ227788.1.1416	Bacteria;Proteobacteria;Alphaproteobacteria;Caulobacterales;Caulobacteraceae;Brevundimonas;Caulobacter_sp.	100	0.0011083	0	0.00138673	0.00102209	0
OTU865	GQ340146.1.1393	Bacteria;Bacteroidota;Bacteroidia;Flavobacteriales;Crocinitomicaceae;Fluviicola;uncultured_bacterium	99.76	0.0011083	0	0.0041602	0	0
OTU866	KM823749.1.1521	Bacteria;Bacteroidota;Bacteroidia;Chitinophagales;Chitinophagaceae;Dinghuibacter;uncultured_bacterium	100	0	0	0.00138673	0	0
OTU868	AM997293.1.1472	Bacteria;Chloroflexi;Dehalococcoidia;SAR202_clade;uncultured_deep-sea_bacterium	96.77	0	0	0.00138673	0	0
OTU869	GT573992.1.1479	Bacteria;Actinobacteriota;Actinobacteria;Pseudonocardiales;Pseudonocardiaceae;Pseudonocardia;uncultured_bacterium	100	0.0011083	0.00211224	0	0	0
OTU87	JQ278985.1.1494	Bacteria;Proteobacteria;Gammaproteobacteria;Beggiatoales;Beggiatoaceae;uncultured;uncultured_gamma_proteobacterium	98.83	0.00997473	0.01161735	0.0041602	0.00613252	0.00394933
OTU871	LCOX01000043.1193.4545	Bacteria;Patescibacteria;Parcubacteria;Candidatus_Jorgensenbacteria;Parcubacteria_group_bacterium_GW2011_GWA2_47_9	83.94	0	0	0	0	0.00098733
OTU873	KC914427.1.1524	Bacteria;Proteobacteria;Gammaproteobacteria;Pseudomonadales;Spongiibacteraceae;BD1-7_clade;uncultured_proteobacterium	97.66	0	0	0	0.00102209	0
OTU874	AM183105.1.1436	Bacteria;Firmicutes;Clostridia;Clostridiales;Clostridiaceae;Clostridium_sensu_stricto_1;uncultured_bacterium	100	0.0011083	0.00105612	0	0	0
OTU875	AB753964.1.1456	Bacteria;Bacteroidota;Bacteroidia;Flavobacteriales;Crocinitomicaceae;Fluviicola;uncultured_bacterium	99.53	0.0011083	0	0.00138673	0	0
OTU878	CEPZ01014827.1.1192	Archaea;Nanoarchaeota;Nanoarchaeia;Woesearchaeales;marine_metagenome	85.9	0.0011083	0	0	0	0
OTU88	JQ278985.1.1494	Bacteria;Proteobacteria;Gammaproteobacteria;Beggiatoales;Beggiatoaceae;uncultured;uncultured_gamma_proteobacterium	99.3	0.00997473	0.00739286	0.00832039	0.01124296	0.00493666
OTU880	KC255345.1.1448	Bacteria;Proteobacteria;Alphaproteobacteria;Rhizobiales;A0839;uncultured_bacterium	99.26	0	0	0	0	0.00098733
OTU882	MHGG01000050.25339.26910	Bacteria;Verrucomicrobiota;Omnitrophia;Omnitrophales;Omnitrophaceae;Candidatus_Omnitrophus;Omnitrophica_WOR_2_bacterium_GWF2_63_9	94.41	0	0	0.00138673	0.00306626	0
OTU883	KX172614.1.1403	Bacteria;Patescibacteria;Parcubacteria;Candidatus_Yanofskybacteria;uncultured_bacterium	89.38	0.00221661	0	0	0	0
OTU884	JQ072424.1.1416	Bacteria;Proteobacteria;Gammaproteobacteria;Burkholderiales;Comamonadaceae;Inhella;uncultured_bacterium	99.53	0.0011083	0	0.00138673	0	0.00098733
OTU885	ET834774.1.1419	Bacteria;Proteobacteria;Alphaproteobacteria;Rhizobiales;Rhizobiales_Incertae_Sedis;Phreatobacter;uncultured_bacterium	99.25	0	0	0.00277346	0	0
OTU886	LCOK01000044.9216.10662	Bacteria;Patescibacteria;Parcubacteria;Candidatus_Ryanbacteria;Candidatus_Giovannonibacteria_bacterium_GW2011_GWB1_47_6b	83.85	0	0.00105612	0	0	0
OTU887	AB630583.1.1469	Bacteria;Actinobacteriota;Thermoleophilia;Solirubrobacterales;67-14;uncultured_bacterium	97.42	0	0.00105612	0	0	0
OTU888	HQ132472.1.1464	Bacteria;Patescibacteria;Parcubacteria;Candidatus_Kaiserbacteria;uncultured_bacterium	89.65	0	0	0	0.00102209	0
OTU89	HE602802.1.1436	Bacteria;Elusimicrobiota;Lineage_IIb;uncultured_bacterium	94.56	0.00221661	0.00739286	0.00138673	0.00408835	0.00691133
OTU890	AB753866.1.1385	Bacteria;Proteobacteria;Alphaproteobacteria;Rhodobacterales;Rhodobacteraceae;Cereibacter;uncultured_bacterium	100	0.0011083	0	0.00138673	0	0.00197467
OTU891	AJ605292.1.1510	Bacteria;Firmicutes;Bacilli;Paenibacillales;Paenibacillaceae;Paenibacillus;Paenibacillus_antarcticus	99.3	0	0	0.19552918	0.0398614	0
OTU893	AJ007870.1.1432	Bacteria;Bacteroidota;Bacteroidia;Chitinophagales;uncultured;uncultured_Cytophagales_bacterium	99.29	0	0.00105612	0	0.00102209	0.00098733
OTU894	ET731481.1.972	Archaea;Nanoarchaeota;Nanoarchaeia;Woesearchaeales;SCGC_AAA011-D5;uncultured_euryarchaeote	78.91	0	0	0	0	0.00098733
OTU895	KC189685.1.1451	Bacteria;Proteobacteria;Alphaproteobacteria;Sphingomonadales;Sphingomonadaceae;Rhizorhapis;uncultured_bacterium	100	0	0.00105612	0	0	0.00197467
OTU898	FJ664807.1.1422	Bacteria;Proteobacteria;Alphaproteobacteria;Sphingomonadales;Sphingomonadaceae;Sphingomonas;uncultured_Alphaproteobacteria_bacterium	100	0.00332491	0	0	0	0
OTU9	KT515071.1.1498	Bacteria;Proteobacteria;Gammaproteobacteria;Burkholderiales;Oxalobacteraceae;Massilia;uncultured_bacterium	100	0.04544044	0.05491836	0.04853561	0.03781723	0.0454173
OTU90	AB681347.1.1462	Bacteria;Proteobacteria;Gammaproteobacteria;Pseudomonadales;Pseudomonadaceae;Pseudomonas;Pseudomonas_gessardii	100	0.02881589	0.03062755	4.41674063	0.04599393	0.01382266
OTU901	CP019239.653029.654571	Bacteria;Proteobacteria;Gammaproteobacteria;Burkholderiales;Comamonadaceae;Rhodoferax;Rhodoferax_saidenbachensis	100	0.0011083	0.00105612	0	0.00102209	0.002962

OTU902	MHOD01000002.3577.5272	Bacteria;Patescibacteria;Parcubacteria;Candidatus_Portnoybacteria;Candidatus_Spechtbacteria_bacterium_RIFCSPHIGHO2_01_FTLL_43_30	89.18	0	0	0	0.00102209	0
OTU905	GT305831.1.1494	Bacteria;Bacteroidota;Bacteroidia;Chitinophagales;Chitinophagaceae;Sediminibacterium;uncultured_bacterium	99.53	0.00221661	0.00105612	0	0	0
OTU906	GQ085127.1.1344	Bacteria;Actinobacteriota;Actinobacteria;Corynebacteriales;Corynebacteriaceae;Corynebacterium;uncultured_bacterium	100	0	0.00105612	0	0.00204417	0.00098733
OTU907	DQ911241.1.1404	Bacteria;Firmicutes;Clostridia;Peptostreptococcales-Tissierellales;Family_XI;Peptoniphilus;Peptoniphilus_gorbachii	100	0	0	0.00138673	0	0
OTU908	MF942642.1.1390	Bacteria;Verrucomicrobiota;Omnitrophia;Omnitrophales;Omnitrophaceae;Candidatus_Omnitrophus;uncultured_bacterium	92.31	0.0011083	0	0	0	0
OTU91	AB008214.1.1540	Bacteria;Firmicutes;Bacilli;Lactobacillales;Streptococcaceae;Lactococcus;Lactococcus_lactis_subsp._cremoris	100	0.00332491	0.00211224	0.00554693	0.00613252	0.00197467
OTU910	GQ031186.1.1343	Bacteria;Actinobacteriota;Actinobacteria;Corynebacteriales;Corynebacteriaceae;Corynebacterium;uncultured_bacterium	100	0.0011083	0.00211224	0	0	0
OTU915	ET134560.1.1376	Bacteria;Myxococcota;Polyangia;Haliangiales;Haliangiaceae;Haliangium;uncultured_bacterium	94.63	0	0.00105612	0	0	0
OTU917	KX172614.1.1403	Bacteria;Patescibacteria;Parcubacteria;Candidatus_Yanofskybacteria;uncultured_bacterium	95.13	0	0.00105612	0	0	0
OTU918	AB805880.1.1415	Bacteria;Patescibacteria;Parcubacteria;Candidatus_Yanofskybacteria;uncultured_bacterium	77.64	0.0011083	0	0	0	0
OTU919	MNWJ01000041.315.1840	Bacteria;Patescibacteria;Parcubacteria;CG1-02-42-13;Parcubacteria_group_bacterium_CG1_02_42_13	83.7	0	0	0.00138673	0	0.00098733
OTU92	MF806552.1.1453	Bacteria;Proteobacteria;Gammaproteobacteria;Burkholderiales;Comamonadaceae;Hydrogenophaga;uncultured_bacterium	100	0.00664982	0.00633673	0.00832039	0.0081767	0.0454173
OTU920	KC886756.1.1485	Bacteria;Bacteroidota;Bacteroidia;Flavobacteriales;Crocinitomicaceae;Fluviicola;uncultured_Cryomorphaceae_bacterium	100	0.0011083	0	0.0041602	0	0
OTU922	JQ977347.1.1428	Bacteria;Proteobacteria;Gammaproteobacteria;Burkholderiales;Oxalobacteraceae;Janthinobacterium;Janthinobacterium_sp._Ata9	100	0	0	0	0	0.00493666
OTU925	KY356875.1.908	Archaea;Nanoarchaeota;Nanoarchaeia;Woesearchaeales;uncultured_archaeon	87.99	0	0.00105612	0	0	0
OTU929	MHGG01000050.25339.26910	Bacteria;Verrucomicrobiota;Omnitrophia;Omnitrophales;Omnitrophaceae;Candidatus_Omnitrophus;Omnitrophica_WOR_2_bacterium_GWF2_63_9	93.01	0	0.00105612	0	0.00102209	0
OTU93	ET937893.1.1500	Bacteria;Nitrospirota;Nitrospiria;Nitrospirales;Nitrospiraceae;Nitrospira;uncultured_bacterium	100	0.00554152	0.00528061	0.00277346	0.00715461	0.00098733
OTU933	KF733329.1.1428	Bacteria;Proteobacteria;Gammaproteobacteria;Pseudomonadales;Pseudomonadaceae;Pseudomonas;Pseudomonas_sp._B12(2014)	100	0.00221661	0	0.00554693	0.00102209	0.22609915
OTU934	AJ697701.1.1474	Bacteria;Bacteroidota;Bacteroidia;Flavobacteriales;Crocinitomicaceae;Fluviicola;uncultured_Sphingobacteriales_bacterium	99.76	0	0.00105612	0.00138673	0	0
OTU935	KP686648.1.1428	Bacteria;Patescibacteria;Parcubacteria;uncultured_bacterium	79.55	0	0.00211224	0	0	0
OTU937	AB002520.1.1377	Bacteria;Firmicutes;Bacilli;Lactobacillales;Streptococcaceae;Streptococcus;Streptococcus_mitis	100	0	0	0	0.00102209	0
OTU939	HQ119895.1.1474	Bacteria;Chloroflexi;Anaerolineae;Anaerolineales;Anaerolineaceae;uncultured;uncultured_bacterium	99.5	0	0.00105612	0	0	0
OTU94	ET803439.1.1482	Bacteria;Actinobacteriota;Actinobacteria;Frankiales;Sporichthyaceae;hgcI_clade;uncultured_bacterium	100	0.00332491	0.00739286	0.00693366	0.00511044	0.002962
OTU940	JQ794632.1.1352	Bacteria;Bacteroidota;Bacteroidia;Sphingobacteriales;env.OPS_17;uncultured_Bacteroidetes_bacterium	99.05	0	0	0.00138673	0	0
OTU947	AB240359.1.1516	Bacteria;Bdellovibrionota;Oligoflexia;0319-6G20;uncultured_bacterium	87.59	0	0.00211224	0	0	0
OTU949	AJ966883.1.1522	Bacteria;Verrucomicrobiota;Verrucomicrobiae;Verrucomicrobiales;Verrucomicrobiaceae;Prosthecobacter;Prosthecobacter_vanneervenii	99.3	0	0	0.00138673	0.00102209	0
OTU95	HF558545.1.1497	Bacteria;Nitrospirota;Nitrospiria;Nitrospirales;Nitrospiraceae;Nitrospira;uncultured_bacterium	100	0.00221661	0.00422449	0	0.00204417	0.005924
OTU951	KF836147.1.1531	Bacteria;Nitrospirota;Leptospirillia;Leptospirillales;Leptospirillaceae;Leptospirillum;uncultured_bacterium	91.12	0	0	0	0.00102209	0
OTU956	AB935927.1.1388	Bacteria;Cyanobacteria;Cyanobacteriia;Cyanobacteriales;Nostocaceae;Dolichospermum_NIES41;uncultured_cyanobacterium	100	0	0.00211224	0	0	0
OTU958	JN527220.1.1490	Bacteria;Desulfobacterota;Desulfobacteria;Desulfatiglandales;Desulfatiglandaceae;Desulfatiglans;uncultured_organism	78.27	0	0	0	0.00102209	0.00098733
OTU959	AB930763.1.1448	Bacteria;Bacteroidota;Bacteroidia;Flavobacteriales;Crocinitomicaceae;Fluviicola;uncultured_bacterium	99.53	0.0011083	0.00105612	0.00554693	0	0
OTU96	FPLS01014480.18.1399	Bacteria;Proteobacteria;Gammaproteobacteria;Burkholderiales;Comamonadaceae;Rhizobacter;metagenome	99.53	0.00554152	0.00211224	0.00693366	0.00204417	0.00493666
OTU961	AJ439343.1.1503	Bacteria;Actinobacteriota;Actinobacteria;Corynebacteriales;Corynebacteriaceae;Corynebacterium;Corynebacterium_pseudodiphtheriticum	100	0.0011083	0.00105612	0	0.00204417	0
OTU962	ET434595.1.1446	Bacteria;Actinobacteriota;Actinobacteria;Corynebacteriales;Corynebacteriaceae;Corynebacterium;Corynebacterium_coyleae	100	0	0	0.00138673	0	0.00098733
OTU963	MNDM01000022.26933.28470	Bacteria;Nitrospirota;Nitrospiria;Nitrospirales;Nitrospiraceae;Nitrospira;Nitrospirae_bacterium_13_2_20CM_2_62_8	99.52	0.0011083	0	0	0	0
OTU965	MHJF01000011.24156.25754	Bacteria;Patescibacteria;Parcubacteria;Candidatus_Colwellbacteria;Candidatus_Harrisonbacteria_bacterium_RIFCSPHIGHO2_02_FTLL_40_20	85.11	0.0011083	0.00105612	0.00138673	0	0
OTU967	HM186577.1.1351	Bacteria;Planctomycetota;Pla4_lineage;uncultured_bacterium	97.52	0	0.00105612	0	0	0
OTU969	ET801139.1.1499	Bacteria;Proteobacteria;Gammaproteobacteria;Burkholderiales;Comamonadaceae;Limnohabitans;uncultured_bacterium	99.3	0	0.00105612	0.00832039	0	0.00197467
OTU97	JX406201.1.1488	Bacteria;Proteobacteria;Gammaproteobacteria;Pseudomonadales;Moraxellaceae;Perlucidibaca;uncultured_bacterium	100	0.00775812	0.01372959	0.00554693	0.01022087	0.005924
OTU970	MF942640.1.1495	Bacteria;Verrucomicrobiota;Omnitrophia;Omnitrophales;Omnitrophaceae;Candidatus_Omnitrophus;uncultured_bacterium	88.58	0	0	0	0	0.00098733
OTU971	AM997348.1.1515	Bacteria;Myxococcota;bacteriap25;uncultured_deep-sea_bacterium	84.07	0	0	0.00138673	0	0
OTU974	MH091508.1.1423	Bacteria;Proteobacteria;Alphaproteobacteria;Sphingomonadales;Sphingomonadaceae;Sandaracinobacter;bacterium	100	0	0.00105612	0.00138673	0	0
OTU98	FPLP01007714.16.1529	Bacteria;Proteobacteria;Gammaproteobacteria;Burkholderiales;Comamonadaceae;Limnohabitans;metagenome	100	0.00554152	0.00739286	0.00693366	0.00408835	0.00493666
OTU980	AB672322.1.1383	Bacteria;Patescibacteria;Parcubacteria;Candidatus_Kaiserbacteria;uncultured_bacterium	95.56	0	0	0.00138673	0	0
OTU981	EF018687.1.1337	Bacteria;Proteobacteria;Alphaproteobacteria;Rhodospirillales;uncultured;uncultured_bacterium	98.51	0	0	0	0	0.00098733
OTU983	JQ278884.1.1533	Bacteria;Verrucomicrobiota;Omnitrophia;Omnitrophales;Omnitrophaceae;Candidatus_Omnitrophus;uncultured_bacterium	92.33	0.0011083	0.00105612	0	0	0
OTU984	FJ820442.1.1469	Bacteria;Bacteroidota;Bacteroidia;Flavobacteriales;Cryomorphaceae;uncultured;uncultured_bacterium	99.53	0	0	0.00138673	0	0
OTU987	FPLP01000459.14.1516	Bacteria;Bacteroidota;Bacteroidia;Sphingobacteriales;LiTT-11-161;metagenome	89.1	0	0	0.00138673	0	0
OTU99	FJ827871.1.1494	Bacteria;Actinobacteriota;Acidimicrobiia;Microtrichales;Ilumatobacteraceae;CL500-29_marine_group;uncultured_actinobacterium	100	0.00443321	0.00316837	0.0041602	0.00408835	0.002962
OTU992	AY821999.1.1276	Archaea;Nanoarchaeota;Nanoarchaeia;Woesearchaeales;uncultured_euryarchaeote	85.2	0	0.00211224	0.00138673	0	0
OTU994	AF358012.1.1450	Bacteria;Proteobacteria;Alphaproteobacteria;Rhizobiales;Xanthobacteraceae;uncultured;uncultured_bacterium	99.01	0	0.00105612	0.00554693	0	0
OTU996	HE574348.1.1420	Bacteria;Bacteroidota;Bacteroidia;Sphingobacteriales;NS11-12_marine_group;uncultured_bacterium	99.53	0	0	0.00277346	0	0.00098733
OTU998	FN553456.1.1494	Bacteria;Bdellovibrionota;Oligoflexia;Oligoflexales;Oligoflexaceae;uncultured;uncultured_sediment_bacterium	90.19	0	0.00105612	0	0	0
